# Mouse adaptation of human inflammatory bowel diseases microbiota enhances colonization efficiency and alters microbiome aggressiveness depending on the recipient colonic inflammatory environment

**DOI:** 10.1186/s40168-024-01857-2

**Published:** 2024-08-07

**Authors:** Simon M. Gray, Anh D. Moss, Jeremy W. Herzog, Saori Kashiwagi, Bo Liu, Jacqueline B. Young, Shan Sun, Aadra P. Bhatt, Anthony A. Fodor, R. Balfour Sartor

**Affiliations:** 1 Center for Gastrointestinal Biology and Disease, Department of Medicine, Division of Gastroenterology and Hepatology, University of North Carolina at Chapel Hill, Chapel Hill, NC USA; 2 Lineberger Comprehensive Cancer Center, University of North Carolina at Chapel Hill, Chapel Hill, NC USA; 3 Department of Bioinformatics and Genomics, University of North Carolina at Charlotte, Charlotte, NC USA; 4 Molecular Gastroenterology and Hepatology, Kyoto Prefectural University of Medicine, Kyoto, Japan; 5 Department of Microbiology and Immunology, University of North Carolina at Chapel Hill, Chapel Hill, NC USA; 6 National Gnotobiotic Rodent Resource Center, University of North Carolina at Chapel Hill, Chapel Hill, NC USA

**Keywords:** Inflammatory bowel diseases, Experimental colitis, Human microbiota associated mice, Fecal microbiota transplant, Microbiota transfer efficiency, Mouse-adapted, Interleukin-10 deficient

## Abstract

**Background:**

Understanding the cause vs consequence relationship of gut inflammation and microbial dysbiosis in inflammatory bowel diseases (IBD) requires a reproducible mouse model of human-microbiota-driven experimental colitis.

**Results:**

Our study demonstrated that human fecal microbiota transplant (FMT) transfer efficiency is an underappreciated source of experimental variability in human microbiota-associated (HMA) mice. Pooled human IBD patient fecal microbiota engrafted germ-free (GF) mice with low amplicon sequence variant (ASV)-level transfer efficiency, resulting in high recipient-to-recipient variation of microbiota composition and colitis severity in HMA *Il-10*^*−/−*^ mice. In contrast, mouse-to-mouse transfer of mouse-adapted human IBD patient microbiota transferred with high efficiency and low compositional variability resulting in highly consistent and reproducible colitis phenotypes in recipient *Il-10*^*−/−*^ mice. Engraftment of human-to-mouse FMT stochastically varied with individual transplantation events more than mouse-adapted FMT. Human-to-mouse FMT caused a population bottleneck with reassembly of microbiota composition that was host inflammatory environment specific. Mouse-adaptation in the inflamed *Il-10*^*−/−*^ host reassembled a more aggressive microbiota that induced more severe colitis in serial transplant to *Il-10*^*−/−*^ mice than the distinct microbiota reassembled in non-inflamed WT hosts.

**Conclusions:**

Our findings support a model of IBD pathogenesis in which host inflammation promotes aggressive resident bacteria, which further drives a feed-forward process of dysbiosis exacerbated by gut inflammation. This model implies that effective management of IBD requires treating both the dysregulated host immune response and aggressive inflammation-driven microbiota. We propose that our mouse-adapted human microbiota model is an optimized, reproducible, and rigorous system to study human microbiome-driven disease phenotypes, which may be generalized to mouse models of other human microbiota-modulated diseases, including metabolic syndrome/obesity, diabetes, autoimmune diseases, and cancer.

Video Abstract

**Supplementary Information:**

The online version contains supplementary material available at 10.1186/s40168-024-01857-2.

## Introduction

Human inflammatory bowel diseases (IBD) are heterogeneous chronic inflammatory conditions driven by microbial activation of dysregulated intestinal immune responses in genetically susceptible hosts [1]. Host genetic susceptibility loci, such as polymorphisms in *Nod2*, *Il23r*, *Il-10r*, and *Il-10*, explain < 20% of IBD variance [2–4] and disease incidence is rising globally [5], suggesting that environmental factors (diet, microbiome) are important drivers of IBD. IBD patients have altered intestinal microbiota composition (dysbiosis), functionally characterized by reduced diversity, unstable community structure over time and following perturbation, and expanded aggressive (*Gammaproteobacteria, Enterococcaceae*, sulfur-reducing bacteria) but reduced beneficial (short-chain fatty acid [SCFA]-producing *Clostridiales*, *Blautia*) resident bacteria [6–10]. Viable microbes are required to develop chronic T cell mediated intestinal inflammation in most experimental colitis models (i.e., *Il-10*^*−/−*^*, Il2*^*−/−*^*, Tcrab*^*−/−*^*,* Naïve CD4^+^ T cell transfer to *Rag1/2*^*−/−*^, *Tlr5*^*−/−*^*, Tnf*^*ΔARE*^ mice) in which GF mice have no inflammation but develop progressive intestinal inflammation after colonization with complex microbiota [11–16]. Aggressive resident bacteria (pathobionts) within the complex gut microbiota are the key drivers of intestinal inflammation [17–22]; however, whether the dysbiotic expansion of pathobionts is a cause or consequence of intestinal inflammation and how the host environment shapes microbial ecology in IBD remain poorly understood.

Colonization of GF animals with defined human bacterial consortia or human fecal microbiota transplant (FMT) is the gold-standard method to demonstrate causality and investigate mechanisms of human microbiome-driven disease phenotypes [23–29]. Defined consortia enable strict control of microbiota composition, which facilitates mechanistic studies using genetically modified consortium members but requires the selection of bacterial strains by variable criteria [28, 30–32]. Strain-level genetic and functional variations are human disease-state specific, strongly impact host-microbe interaction, and alter disease severity in experimental colitis models [22, 33–38]. Because defined consortia may omit strain-specific genetic and functional attributes responsible for human disease phenotypes, direct transplant of human disease-associated feces to GF rodents is an appealing method to study human microbiome-driven diseases.

Human IBD patient FMT to colitis-prone, GF mice (*Il-10*^*−/−*^ and *Rag1*^*−/−*^ T cell transfer models) transfers enhanced colitis severity compared to healthy patient FMT and induces a T_H_17- and T_H_2- dominant immune phenotype that is characteristic of human IBD [26, 39–42]. These fecal transplant studies clearly transfer disease phenotype to susceptible mice by human IBD-associated microbes. Importantly, human-to-mouse fecal transplant causes a microbial population bottleneck that engrafts a compositionally distinct microbiome in recipient mice compared to human donor stool, likely due to low human-to-mouse strain-level transfer efficiency (~ 40%) and host-specific microbe preferences [43–46]. We took advantage of the microbiota reassembly associated with human-to-mouse FMT to ask if (1) the host environment controls microbiota assembly and inflammatory potential, and (2) mouse-adaptation of human fecal microbiota forms a microbial community that is stable in serial transplant to GF mice and leads to more reproducible experimental phenotypes.

To evaluate the impact of the host inflammatory environment on gut microbiota assembly we transferred pooled feces from human IBD patients with active disease to wild-type (WT) or *Il-10*^*−/−*^ mice. Human microbiota-associated (HMA) *Il-10*^*−/−*^ mice had lower microbial alpha diversity, higher compositional variability, and expansion of pathobionts compared to HMA WT mice, illustrating the influence of an inflammatory colonic environment on dysbiosis. Serial transfer of non-inflamed (WT) mouse-adapted human microbiota to GF *Il-10*^*−/−*^ mice induced less severe colitis than inflamed (*Il-10*^*−/−*^) mouse-adapted human microbiota. Transplant of human fecal microbiota to GF mice resulted in low human-to-mouse transfer efficiency at the strain level, while mouse-adapted human microbiota yielded high strain-level transfer efficiency. High microbiota compositional variability in HMA *Il-10*^*−/−*^ mice was associated with variable colitis severity, but recipient mice colonized with mouse-adapted human microbiota exhibited low compositional variability and more consistent colitis phenotypes. Our findings suggest that the reproducibility and rigor of HMA animal studies are impacted by the variability of human-to-mouse FMT; however, experimental design can be improved by first adapting the human microbiota to the mouse host followed by transfer of mouse-adapted human microbiota for subsequent highly reproducible mechanistic studies.

## Methods

### Mouse lines

GF 129S6/SvEv background wildtype (WT) and *Il-10*-deficient (*Il-10*^*−/−*^) mice [13] were obtained from the National Gnotobiotic Rodent Resource Center (NGRRC) at the University of North Carolina at Chapel Hill. All animal experiments were conducted under approved Institutional Animal Care and Use Committee protocols.

### Human fecal samples

Human fecal samples from 5 adult patients with active Crohn’s disease (CD) (4 donors) or ulcerative colitis (UC) (1 donor) without prior intestinal surgery or antibiotic exposure within 3 months were collected under an Institutional Review Board-approved protocol (Figure S1A). De-identified stool samples were aliquoted immediately after collection in an anaerobic chamber and stored without preservatives at − 80 °C until use.

### Human fecal microbiota and mouse-adapted fecal microbiota colonization of GF mice

**Fig. 1:**
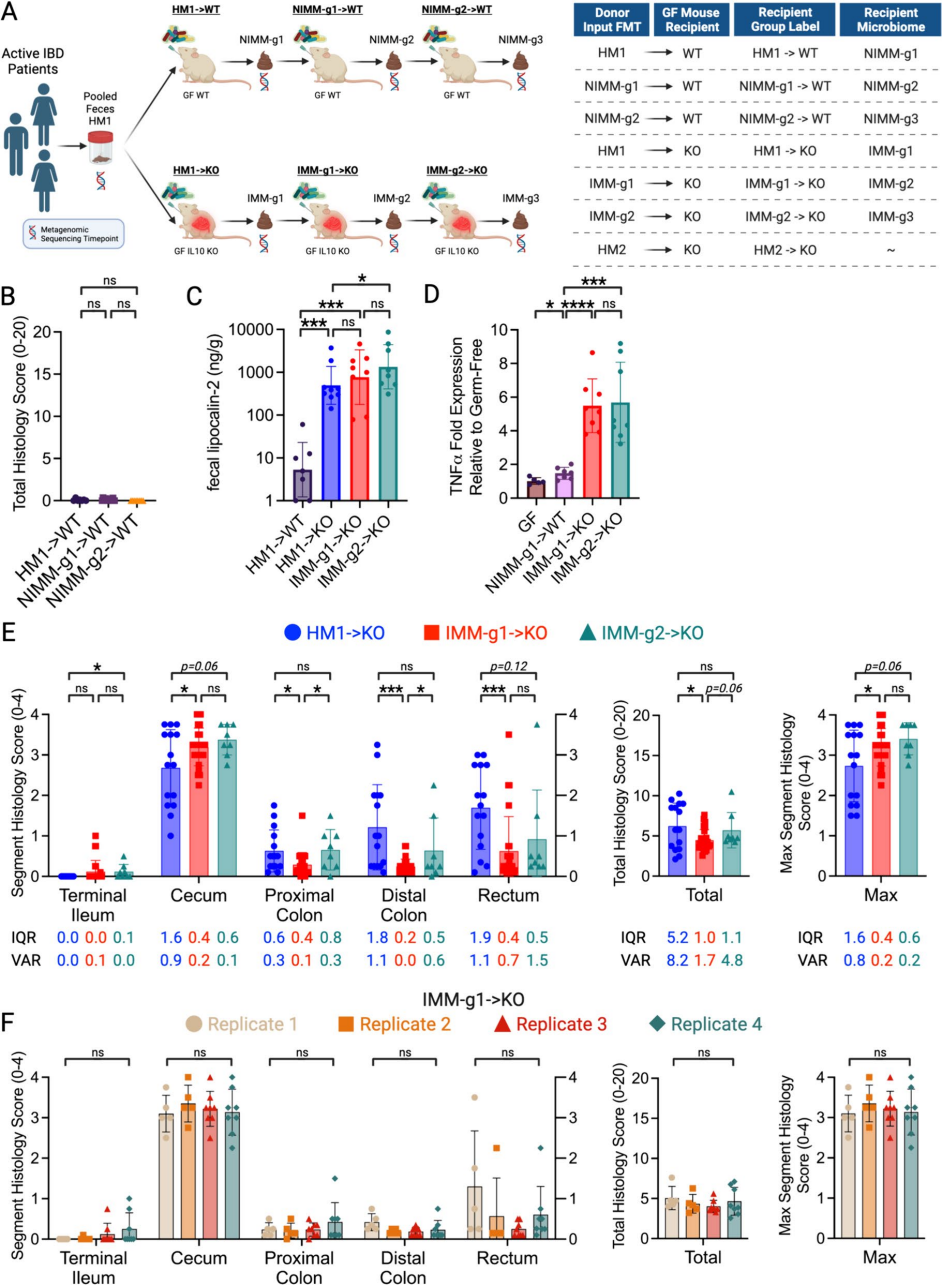
Mouse-adapted human microbiota induces more consistent and reproducible colitis than directly transplanted human microbiota. **A** Experimental design. Pooled feces from 3 humans with active IBD (2 CD, 1UC) were transplanted to non-inflamed WT or colitis-susceptible *Il-10*^−/−^ (IL-10 KO, KO) GF recipient mice. Mouse-adapted microbiotas were serial transplanted to non-inflamed WT or colitis-susceptible *Il-10*^−/−^ GF recipient mice. **B** Total colon and ileum histology score for WT mice at day 28 post-colonization. **C** f-LCN2 level at day 28 post-colonization. **D** TNFα mRNA levels in cecal tissue at day 28 post-colonization. **E** Segment, total colon and ileum, and max segment histology score for *Il-10*^−/−^ mice at day 28 post-colonization. **F** Segment, total colon and ileum, and max segment histology score for IMM-g1 colonized *Il-10*^−/^^−^ mice at day 28 post-colonization from 4 independent experiments. Data shown are representative of **C**–**D** or cumulative **B**, **E**–**F** from 2 to 4 independent experiments. *n* = 7–9 (**B**–**D**), *n* = 15–26 (**E**), *n* = 5–8 (**F**) mice per group. Data are expressed as mean±SD or geometric mean ± geometric SD (**C**). Statistical significance calculated by unpaired *t*-test or Mann–Whitney test (**C**) with *p < 0.05, **p < 0.01, ***p < 0.001

Human fecal material from two sets of 3 human donors with active IBD (HM1: Donors 1, 2, 3; HM2: Donors 3, 4, 5) was thawed and pooled in equal proportions by weight under anaerobic conditions (N_2_:H_2_:CO_2_ = 80:10:10), diluted with anaerobically reduced phosphate-buffered saline (PBS) to generate a fecal slurry, and administered by 150 μl oral gavage to recipient GF 129 WT or 129 *Il-10*^*−/−*^ mice at 2 mg pooled human donor stool per mouse. Mouse-adapted fecal pellets from HMA 129 WT or 129 *Il-10*^*−/−*^ mice were freshly collected and pooled daily between 14 and 21 days post-colonization and frozen at − 80 °C without preservatives. To generate a standardized slurry of mouse-adapted microbiota, mass-collected fecal pellets from all mice in a group were pooled, homogenized, and diluted to 100mg/ml under anaerobic conditions in sterile anaerobically reduced lysogeny broth (LB) with 20% glycerol. Solid particulate matter was pelleted by brief slow centrifugation and slurry supernatant was aliquoted to cryovials for storage at − 80 °C. We have previously demonstrated that slurry supernatant contains the same microbial community composition as whole fecal material [43, 47]. Mouse-adapted microbiota slurry generated as above from fecal pellets of HMA 129 WT mice is called non-inflamed mouse-adapted microbiota (NIMM), while slurry generated from fecal pellets of HMA colitis-prone 129 *Il-10*^*−/−*^ mice is called inflamed mouse-adapted microbiota (IMM) (Fig. 1A). To colonize GF mice with mouse-adapted microbiota, standardized aliquots of 100 mg/ml fecal slurry were thawed under anaerobic conditions, diluted with anaerobically reduced PBS, and administered by oral gavage to recipient GF 129 WT or 129 *Il-10*^*−/−*^ mice at 2 mg per mouse in 150 μl. Fecal pellets from IMM or NIMM associated 129 WT or 129 *Il-10*^*−/−*^ mice were collected daily between 14 and 21 days post-colonization when the *Il-10*^*−/−*^ recipient microbiota has stabilized and before cage effects are reported to develop [48, 49], processed and frozen in aliquots as above to generate standardized slurries of serial passages (-g1, -g2, and -g3) of mouse-adapted microbiota (Fig. 1A). All experiments were performed using aliquots from a single production batch of mouse-adapted microbiota. All mouse fecal transplant experiments were performed in BSL-2 isolation cubicles with HEPA-filtered air on a 12-h dark/light cycle with ad libitum access to autoclaved water and mouse chow (Purina Advanced Protocol Select Rodent 50 IF/6F Auto Diet) using the sterile out-of-isolator gnotobiotic cage technique (Complete cage GM500, Green Line, Tecniplast) [50]. Cage changes and all animal handling were performed in a laminar flow biosafety cabinet under sterile technique following ultraviolet light treatment and 10-min Peroxigard sterilization of all equipment and surfaces. We maintained strict GF conditions with the out-of-isolator gnotobiotic technique for at least 2 weeks. We consider the complex microbiota fecal transplant experiments reported here to be “near-gnotobiotic” with a low risk of environmental contamination, but not strictly gnotobiotic since they are performed with out-of-isolator gnotobiotic cage technique for durations > 2 weeks and sterility could not be monitored due to complex microbiota transplants.

### Gene expression by qRT-PCR, Intestine histopathology score, and Fecal lipocalin-2 quantification

Standard molecular assays and histopathology scoring were performed as previously described [51–53]. Details of these procedures and a list of qPCR primers are found in the Supplemental experimental procedures.

### Statistical analyses

Non-sequencing-based statistical analyses were performed with Prism 10 (GraphPad) with statistical tests and significance thresholds indicated in figure legends.

### 16S rRNA amplicon metagenomic sequencing and analysis

16S rRNA amplicon (variable regions 3–4) sequencing was performed on the Illumina NextSeq 2000 platform, processed, and taxonomically classified through QIIME2 by the UNC Microbiome Core [54]. Additional details of these procedures are found in the Supplemental experimental procedures. Sequence count data at both the genus and phylum level were extracted from the respective QIIME2 artifact files. The amplicon sequence variant (ASV)-level counts table was generated with forward reads using the following parameters with single-end DADA2 on the QIIME2 (version 2021.2) platform: the first 10 base pairs of each sequence were trimmed, and the sequences were truncated to 180 base pairs as determined by sequence quality using FastQC (version 0.11.9) [54, 55]. Statistical analysis was conducted with the *vegan* package (ver.2.6–2) in R (ver. 4.2.2) and visualized with the Shiny application *Plotmicrobiome* and custom R code (Sun et al. GitHub https://github.com/ssun6/plotmicrobiome, Supplemental file 1). To ensure reproducibility and rigor, the results of our analyses were independently reproduced with custom Python code by a second bioinformatician (JBY) with replicated key figures and reproducible tested code available in a Jupyter Notebook file (Supplemental file 1). R and Python code used in our analyses are available at https://github.com/anhmoss/Mouse-Adaptation-of-Human-Inflammatory-Bowel-Disease-Microbiota-Enhances-Colonization-Efficiency and in Supplemental file 1. 16S rRNA amplicon sequencing data are available at https://github.com/anhmoss/Mouse-Adaptation-of-Human-Inflammatory-Bowel-Disease-Microbiota-Enhances-Colonization-Efficiency.

To account for varying sequencing depth, all count data were normalized according to the following formula prior to downstream statistical analyses:

$$\text{log}10 \left(\frac{\text{raw OTU count for }{\text{sample}}_{\text{i}}}{\text{total sequences for }{\text{sample}}_{\text{i}}}\times \text{ average sequence depth }+ 1\right)$$

This formula adjusts the pseudo-count to have a similar effect across samples by scaling all samples to the average sequencing depth. ASV transfer efficiency was measured as Pearson correlation coefficient (*r*) for pairs of samples within a given group or between two groups.

## Results

### Mouse-adapted human microbiota induces more consistent and reproducible colitis than directly transplanted human microbiota

Human fecal microbiota transplantation into GF mice can transfer microbe-dependent pathological phenotypes to recipient animals, allowing investigation of microbial mechanisms of human diseases such as IBD [23, 26, 56]. The large interpersonal variation of human gut microbiota, host-specificity of gut microbial ecology, and variable engraftment of human gut microbes into GF mice pose challenges to transplanted phenotype reproducibility and interpretation [43, 45, 46, 57]. To understand the impact of the recipient host environment on human fecal microbiota engraftment and phenotype transfer in a mouse model of experimental colitis, we transplanted pooled feces (HM1) from 3 humans with active IBD (2 CD, 1UC) to non-inflamed WT or colitis-susceptible *Il-10*^*−/−*^ GF mice (Fig. 1A, S1A). We used pooled feces from UC and CD patients to account for the significant inter-individual heterogeneity of human microbiota composition and because the *Il-10*^*−/−*^ experimental colitis model is not an exact model of either UC or CD but rather has features of both diseases. Key experiments were replicated with a second pooled fecal microbiota containing feces from 3 humans with active CD only (HM2). We then transplanted this mouse-adapted microbiota to sequential cohorts of non-inflamed WT or colitis-susceptible *Il-10*^−/−^ GF recipient mice, generating serial transfers of mouse-adapted human microbiota identified as -g1, -g2, and -g3 (Fig. 1A). In our nomenclature, different human IBD patient fecal pools are called Human Microbiota (HM1 or HM2) (shown in Figure S1A), feces from HMA WT mice are called Non-Inflamed Mouse-adapted Microbiota (NIMM), and feces from HMA *Il-10*^*−/−*^ mice are called inflamed mouse-adapted microbiota (IMM) (Fig. 1A). Serial mouse-adapted fecal transplant experiments were only conducted with HM1-derived HMA mouse stool due to resource constraints; HM1 was selected because the cohort contained both UC and CD donors (Fig. 1A; Figure S1A). Because colonic immune stimulation of GF mice is equivalent following transplant of human or mouse microbiota, HMA mice are a clinically relevant model of experimental colitis [44, 45].

GF 129 WT mice receiving HM1, NIMM-g1, or NIMM-g2 fecal transplant did not develop colitis as assessed by colon histology, non-invasive fecal lipocalin-2 (f-LCN2), and tissue inflammatory cytokine levels (Fig. 1B–D; Figure S1B, C). Transplantation of both human microbiota HM1 and mouse-adapted microbiota IMM-g1 or IMM-g2 to GF 129 *Il-10*^*−/−*^ mice induced severe colitis as assessed by colon histology, non-invasive f-LCN2, and inflammatory cytokine levels (Fig. 1C–F; Figure S1B, D). IMM-g1 and IMM-g2 induced cecal-predominant colitis that was equivalent in severity and kinetics to colitis induced by HM1 (Fig. 1E; Figure S1B, D). However, HM1-induced colitis was more variable than IMM-g1 or IMM-g2-induced colitis as quantified by segment and total histology score variance and interquartile range (Fig. 1E; Figure S1G). The high phenotypic variance of human microbiome-induced colitis was replicated by a separate cohort (HM2) of pooled feces from 3 humans with active CD transplanted to *Il-10*^*−/−*^ GF mice (Figure S1E, F). In contrast to the highly variable phenotype of human microbiome-induced colitis, mouse-adapted microbiome IMM-g1-induced colitis had little variation in severity or distribution within or across independent experiments (Fig. 1E, F; Figure S1G, H). To evaluate whether variability in colitis phenotype was related to microbiome composition, we performed 16S amplicon sequencing of input donor microbiota and fecal samples collected from ex-GF 129 WT and 129 *Il-10*^*−/−*^ mice colonized for 28 days with human microbiota or mouse-adapted microbiotas (Fig. 1A). As we show later in the results (Fig. 3), human microbiota transplant to GF mice was associated with significantly lower microbiota engraftment consistency than mouse-adapted microbiota transplant, suggesting that variability in engrafted human microbiota composition may cause variability in colitis phenotypes.

### Human microbiome restructures with transplant to GF mice

**Fig. 2:**
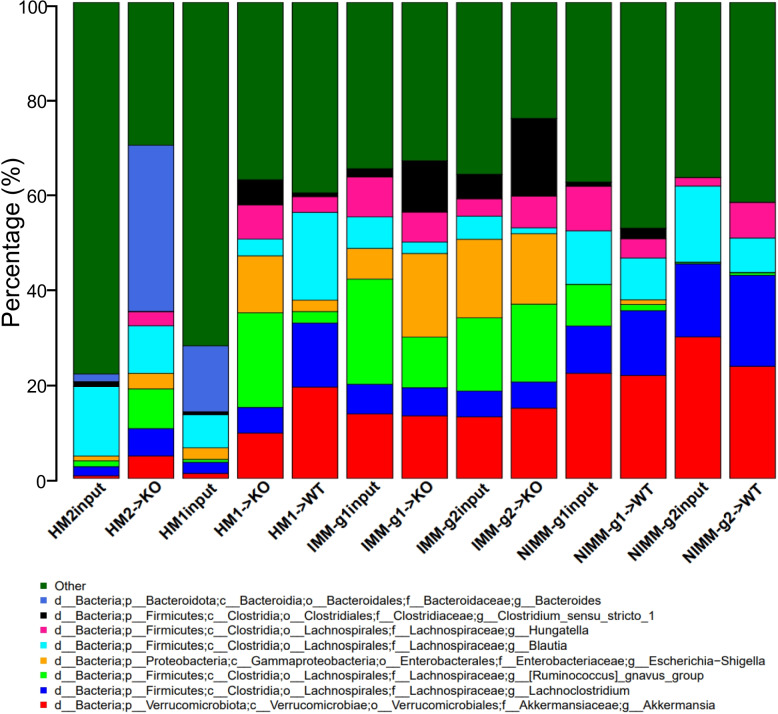
Recipient host environment influences engraftment composition of human-microbiome associated mice. **A** 16S Seq taxonomic bar plots show top 8 most abundant genera in FMT inputs and recipient mouse feces at day 28 post-colonization. For mouse recipient groups, bar plots are average of 16S Seq data from *n* = 7–18 mice/group

To investigate how the recipient host intestinal environment shapes human microbiota engraftment in GF mice, we assessed microbiome compositional variation by calculating the average relative abundance of genera across all samples for each fecal transplant condition. Figure 2 shows taxonomic bar plots of the 8 most abundant genera across groups with the remaining lower abundance taxa grouped as “Other” (Fig. 2, Figure S2A). The 30 most abundant genera across groups and the relative abundance of genera for individual mice are visualized in bar plots in Figure S2. We performed a pairwise Wilcoxon rank sum test to assess differential abundance between groups, excluding genera present in less than 10% of the samples (Table S1).

**Fig. 3:**
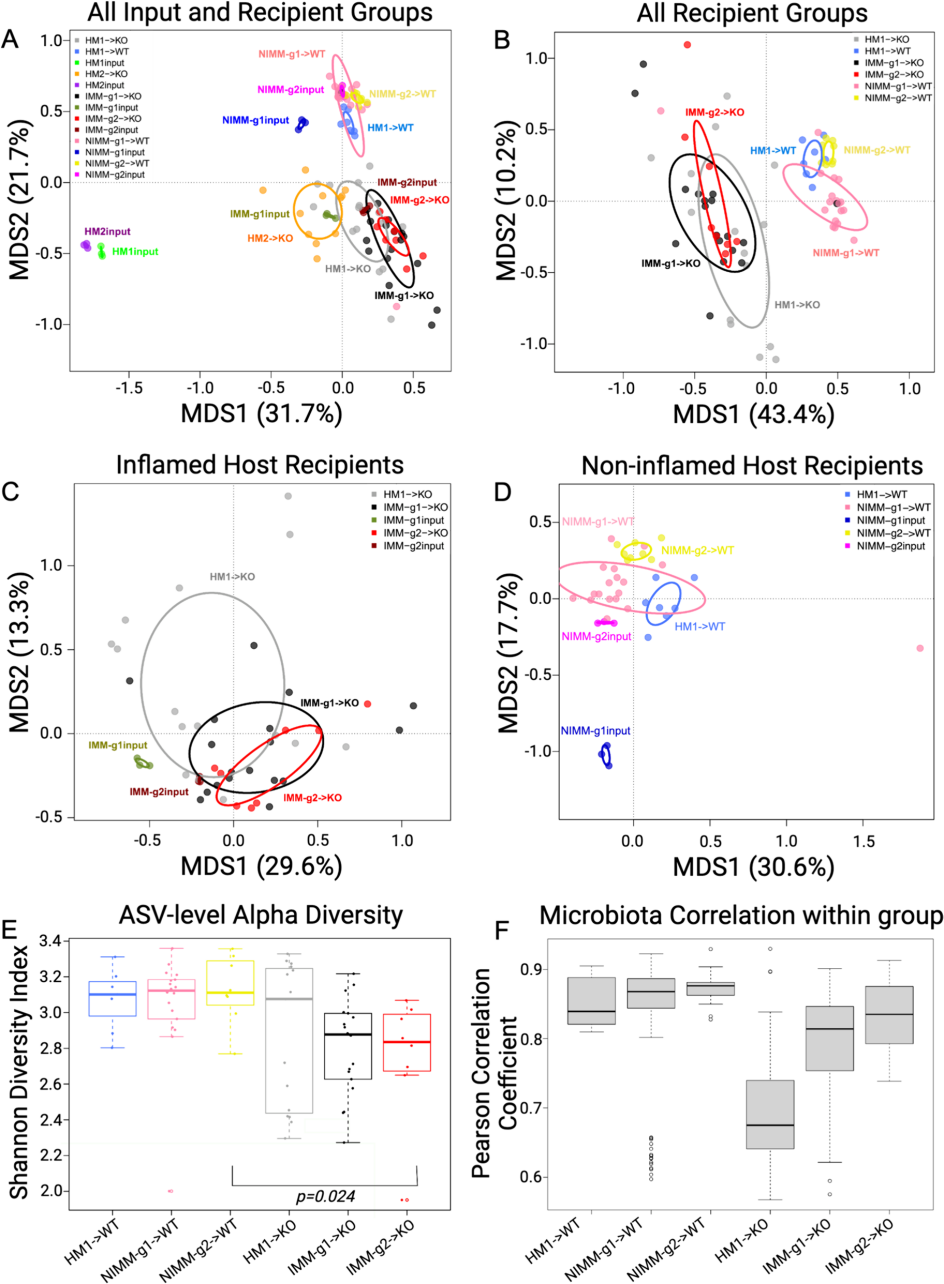
Human microbiome restructuring with transplant to GF mice is host inflammatory environment specific. **A** Principal coordinates analysis, PCoA, of 16S Seq data for human and mouse-adapted FMT inputs and FMT recipient WT and KO mouse groups. **B** PCoA of FMT recipient WT and KO mouse groups. **C** PCoA of FMT recipient KO mouse groups. **D** PCoA of FMT recipient WT mouse groups. **E** Shannon index at ASV level for FMT recipient WT and KO mouse groups. **F** Pearson correlation coefficients (*r*) within group for FMT recipient WT and KO mouse groups quantify the variability of microbiota composition between mice in the same group (microbiota engraftment consistency). Dots in PCoA plots represent individual mice for FMT recipient WT and *Il-10*^−/−^ (KO) mouse groups. For FMT inputs, a single input slurry was used in each experiment and input dots represent sequencing data from three 16S amplicon PCR technical replicates. Analysis conclusions did not change when using average input vs individual technical replicates, so technical replicates are displayed to demonstrate the high consistency of 16S amplicon PCR in our dataset

Pooled human microbiome composition (HM1 input and HM2 input) was compositionally distinct from all colonized mouse groups as visualized by taxonomic bar plots and principal coordinates analysis (PCoA) clustering (aka multidimensional scaling), with the strongest separation existing between human and mouse-adapted microbiotas along the first MDS axis (Figs. 2 and 3A). Compared to human microbiomes, HMA mouse and MA-FMT mouse microbiomes had increased relative abundance of *Akkermansia*, *Lachnoclostridium*, *Ruminococcus gnavus* group, and *Hungatella*, a low-abundance member of the human gut that was not detectable by 16S in HM1 or HM2 inputs (Fig. 2). *Bacteroides*, a major constituent of the human gut microbiome, was present in HM1 input and HM2 input, and expanded in HM2-associated *Il-10*^*−/−*^ mice but reduced in HM1-associated WT and *Il-10*^*−/−*^ mice (Fig. 2, Table S1). The expansion of *Bacteroides* in HM2- but the reduction in HM1-associated *Il-10*^*−/−*^ mice was surprising because *Bacteroides* were more abundant in HM1 input compared to HM2 input, suggesting stochastic factors influence engraftment of human microbiota in GF mice (Fig. 2). These data demonstrate that human microbiota association of GF mice results in major compositional restructuring of the engrafted microbiome that may be partially stochastic.

### Recipient host environment influences the engraftment composition of human-microbiota-associated mice

The recipient host environment shapes the engrafted microbiome composition of HMA mice (Fig. 2, Figure S2, 3A, B). After removing HM1 and HM2 inputs, PCoA showed separation of inflamed mouse-adapted microbiota (IMM) and non-inflamed mouse-adapted microbiota (NIMM) along the first MDS axis (Fig. 3B). PERMANOVA test with all mouse recipient groups as the model term demonstrated that approximately 43% of the variation in the data is explained by the recipient host environment (coefficient of determination, *R*^2^ = 0.43, *p* = 0.001). Mouse adaptation in the inflamed *Il-10*^*−/−*^ host (IMM) enriched for the significantly higher relative abundance of *Escherichia-Shigella*, *Enterococcus, Clostridium_sensu_stricto_1*, *Ruminococcus gnavus group,* and *Bifidobacterium* but significantly lower relative abundance of *Clostridium innocuum, Blautia*, *Lachnoclostridium,* and multiple other genera within *Lachnospiraceae* and *Ruminococaceae* when compared to mouse adaptation in the non-inflamed WT host (NIMM) (Fig. 2, Figure S2, Table S1). PCoA of serial microbiota passage within the non-inflamed WT host environment (NIMM-g1, -g2) showed that the global microbiome structure remained stable with no distinct clustering of groups (Fig. 3D) and only 10 operational taxonomic units (OTUs) demonstrated significant differential abundance between NIMM-g1 and -g2 using a Wilcoxon cutoff of FDR < 0.1 (Fig. 2, Figure S2, Table S1). PCoA of serial microbiota passage within the inflamed *Il-10*^*−/−*^ host environment (IMM-g1, -g2) similarly showed a globally stable microbiome structure with no distinct clustering of groups (Fig. 3C, Figure S3A), while no OTUs were differentially abundant between IMM-g1 and -g2 (Fig. 2, Figure S2, Table S1). Mouse adaptation in the inflamed *Il-10*^*−/−*^ host (IMM) was associated with significantly lower alpha diversity at the amplicon sequence variant (ASV) level compared to the non-inflamed WT host (NIMM), consistent with observations that human IBD patients have lower alpha diversity than healthy humans (Fig. 3E, Figure S3B, C) [8]. Together, these data demonstrate that the composition of the human microbiome is fundamentally restructured with transplant to GF mice and that the recipient host environment strongly shapes the relative abundance of engrafted strains with the inflamed *Il-10*^*−/−*^ host (IMM) driving a dysbiotic microbiome defined by lower alpha-diversity, enrichment of pathobionts, and reduction of protective SCFA-producing bacteria relative to the non-inflamed WT host (NIMM).

### Human microbiota engrafts with variable composition compared to more consistent engraftment by mouse-adapted microbiota

Since the human microbiome restructures with transplant to GF mice, we speculated that variability in engrafted microbiota composition may explain the colitis phenotype variability of HMA *Il-10*^*−/−*^ mice (Fig. 1E, Figure S1E, F). Variability of microbiota composition was quantified by pairwise calculation of the Pearson correlation coefficient for all samples within the same group (i.e., all mice within HM1- > KO). A high Pearson correlation coefficient indicates compositional similarity between samples in a group, while a low coefficient indicates compositional variability between samples in a group. Human microbiota transplant to 129 *Il-10*^*−/−*^ mice (HM1- > KO) was associated with significantly lower Pearson correlation coefficients than mouse-adapted microbiota transplants to 129 *Il-10*^*−/−*^ mice (IMM-g1- > KO, IMM-g2- > KO) (Fig. 3F, Figure S3D). A similar trend was seen with human microbiota or mouse-adapted microbiota transplant to 129 WT mice (Fig. 3F). Pearson correlation coefficients for 129 *Il-10*^*−/−*^ recipient mice were consistently lower than 129 WT recipients at each stage of serial passage (i.e., HM1- > WT vs HM1- > KO or NIMM-g1- > WT vs IMM-g1- > KO), demonstrating that inflammation promotes variability of microbiome composition while health is associated with microbiome stability (Fig. 3F). These results are consistent with observations in humans that the composition of IBD patient microbiomes fluctuate more than healthy controls over time [28, 58]. Together, these data suggest that (1) inflammation promotes microbiome variability and (2) variability in colitis phenotype with human microbiota transplant may be due to variability in engrafted human microbiota composition, while the more consistent colitis induced by mouse-adapted microbiota may be due to homogeneity of engraftment of mouse-adapted microbiota.

### Mouse-adapted human IBD microbiota transfers with higher efficiency than human fecal transplant

**Fig. 4:**
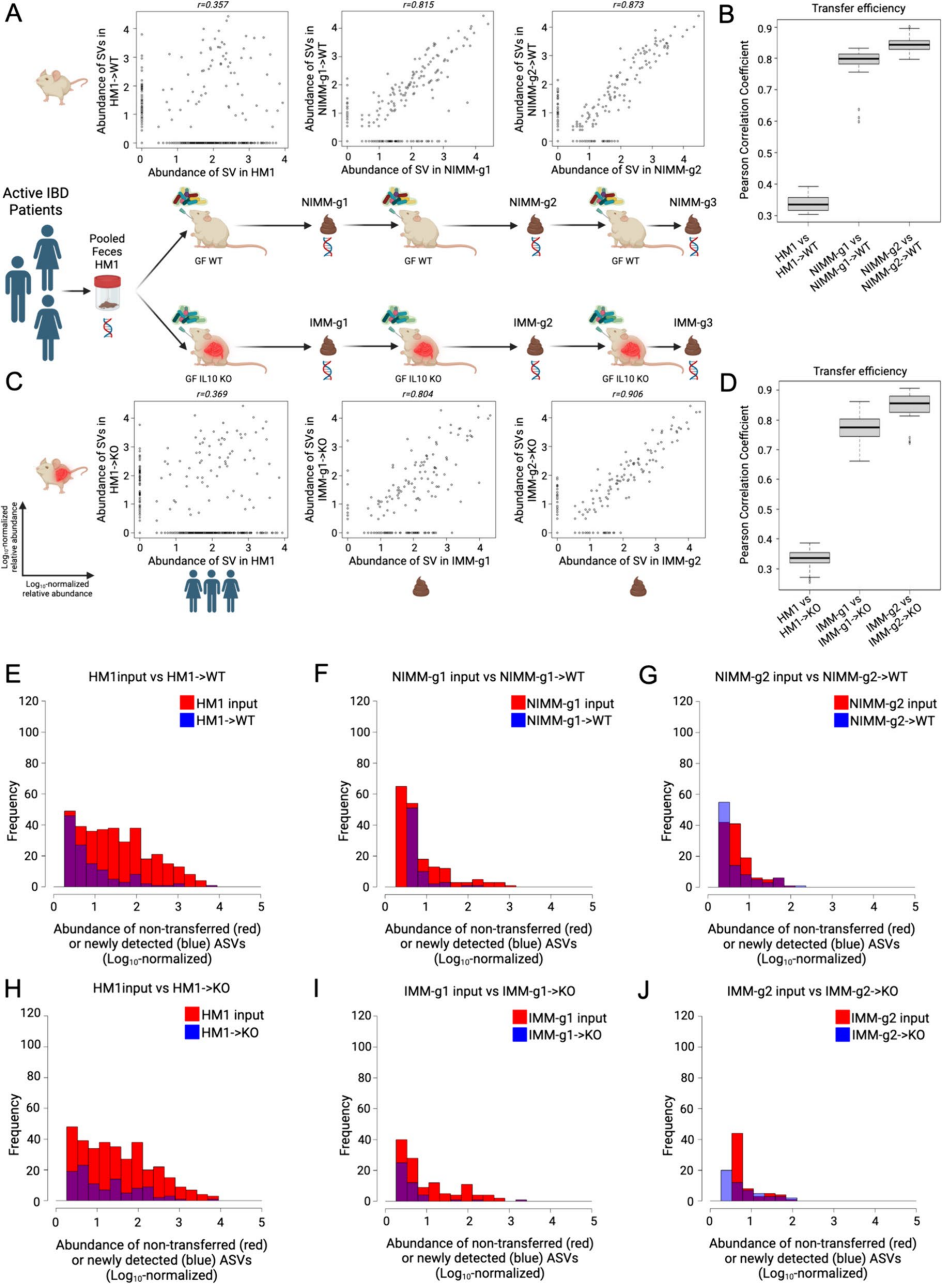
Mouse-adapted human IBD microbiota transfers with higher efficiency than human fecal transplant. **A** ASV level log_10_-normalized relative abundance correlations for FMT input and WT recipient mice where each dot represents a unique ASV plotted in the input microbiome (*x*-axis) vs recipient mouse microbiome (*y*-axis). **B** Transfer efficiency quantified by Pearson correlation coefficient (*r*) between FMT input and WT recipient mouse groups at the ASV level. **C** ASV level log_10_-normalized relative abundance correlations for FMT input and KO recipient mice. **D** Transfer efficiency quantified by Pearson correlation coefficient (*r*) between FMT input and KO recipient mouse groups at the ASV level. **E**–**J** Representative histograms of non-transferring ASVs (red, representing *y* = 0 ASVs in above dot plots) and newly detected in vivo ASVs (blue, representing *x* = 0 ASVs in above dot plots) binned by log_10_-normalized relative abundance for **E** HM1- > WT, **F** NIMM-g1- > WT, **G** NIMM-g2- > WT, **H** HM1- > KO, **I** IMM-g1- > KO, and **J** IMM-g2- > KO FMT recipient mouse groups

Since HMA mice had significantly different microbiome composition than human donor stool but mouse-adapted FMT mice had highly consistent microbiomes between serial transfer, we evaluated whether mouse-adapted microbiota transfers to GF mice more efficiently than human fecal transplant (Fig. 4A–D). To quantify transfer efficiency, we detected all ASV across all samples and compared ASV abundance between human stool, HMA mice, and mouse-adapted FMT mice across serial transfers (Fig. 4A–D). We visualized these data using scatter plots where each dot represents a unique ASV plotted by log_10_ relative abundance in the input microbiome (*x*-axis) vs recipient mouse microbiome (*y*-axis). We quantified transfer efficiency using Pearson correlation coefficient (*r*), where high Pearson *r* indicates consistent ASV abundances between samples and high transfer efficiency. We used deep 16S amplicon sequencing rather than whole genomic shot gun sequencing (WGS) because repeat sequencing of the same region allows for exact identification of ASVs in a database-independent manner without reliance on classification algorithms.

**Fig. 5:**
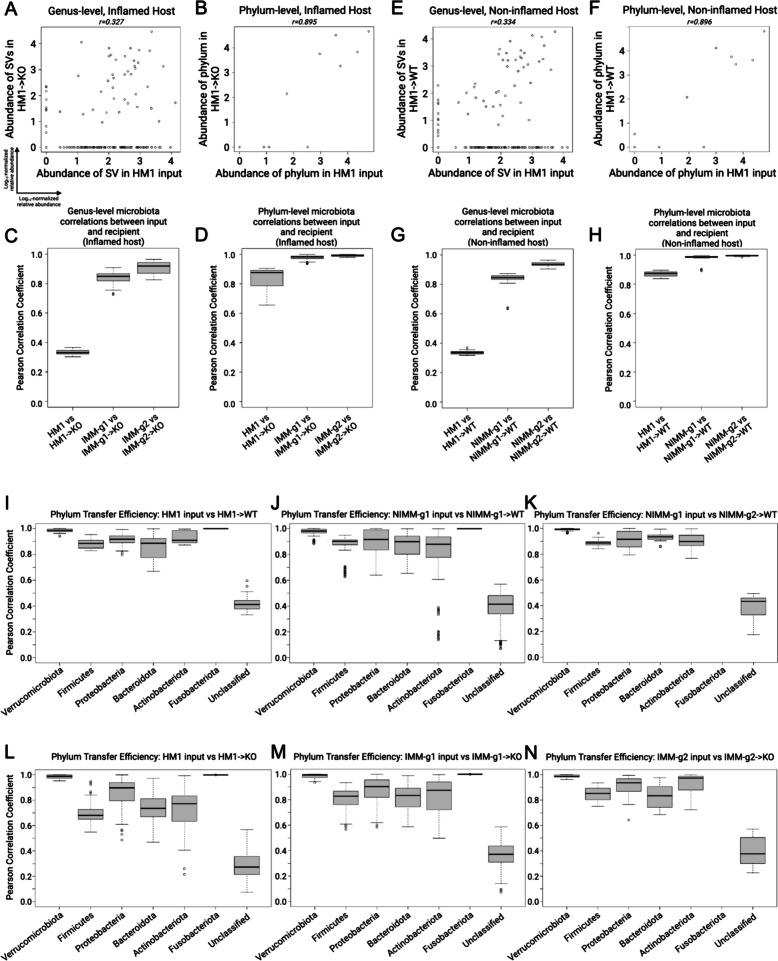
Transfer efficiency varies between taxa. **A** Genus-level and **B** phylum-level log_10_-normalized relative abundance correlations comparing HM1 input to HM1- > KO, **C**–**D** Pearson correlation coefficient (*r*) between input and inflamed (KO) recipient at the **C** genus- and **D** phylum-level. **E** Genus-level and **F** phylum-level log_10_-normalized relative abundance correlations comparing HM1 input to HM1- > WT, **G**–**H** Pearson correlation coefficient (*r*) between input and non-inflamed (WT) recipient at the **G** genus- and **H** phylum-level. **I**–**K** Pearson correlation coefficient (*r*) between input and non-inflamed (WT) recipients by phylum. **L**–**N** Pearson correlation coefficient (*r*) between input and inflamed (KO) recipients by phylum

Human fecal transplant to WT or *Il-10*^*−/−*^ mice was associated with low transfer efficiency and poor transfer of relative composition to recipient mice (Fig. 4A–D, Figure S5D-E). Very similar results were seen in WT and *Il-10*^*−/−*^ mice. A large proportion of ASVs present in human stool did not transfer to recipient mice, which is illustrated by ASVs falling on the x-axis (Fig. 4A, C). The relative abundance (log_10_ normalized) of non-transferring ASVs demonstrated that even moderately to highly abundant ASVs in human stool did not transfer efficiently to GF mice (Fig. 4E, H). ASV relative abundance in human stool had little correlation with relative abundance in recipient mice (Fig. 4A, C), leading to a very low ASV level transfer efficiency for human fecal transplant to WT (*r* = 0.34±0.03) or *Il-10*^*−/−*^ (*r* = 0.33±0.03) mice (Fig. 4B, D). For mouse-adapted FMT, however, ASV relative abundance in MA-FMT input (IMM or NIMM) was highly correlated with relative abundance in recipient mice (Fig. 4A, C). Only a small proportion of ASVs present in mouse-adapted microbiota did not transfer to recipient mice, and those non-transferring ASVs were primarily low-abundance strains (Fig. 4A, C, F–G, I–J). Serial transfer of mouse-adapted microbiota further improved the correlation between input and recipient microbiomes and reduced non-transferring ASV numbers, leading to very high ASV level transfer efficiency for mouse-adapted FMT to WT (*r* = 0.84 ±0.02) or *Il-10*^*−/−*^ mice (*r* = 0.85 ±0.05) (Fig. 4A–D). Similar results were found when comparing human microbiome input to mouse-adapted microbiome inputs (Figure S4A–F). Analysis of transfer efficiency at the genus level also demonstrated low transfer efficiency for human fecal transplant but high transfer efficiency for mouse-adapted FMT; however, phylum level analysis showed high transfer efficiency for all conditions, giving a misleading perception of transfer efficiency (Fig. 5A–H, Figure S5F).

Some ASVs (falling on the *y*-axis) in HMA mice were not detected in human stool, representing either mutation of the V3–V4 sequence, in vivo expansion of very low abundance strains undetected at the depth of 16S sequencing utilized, or environmental contamination (Fig. 4A, C). To rule out environmental contamination, we performed human FMT to GF mice in strictly gnotobiotic isolators and still detected many ASVs in HMA mice that were not detected in human input stool by 16S Seq (Figure S5D–E). We uniquely re-analyzed an independently published 16S Seq dataset of HMA WT mice colonized with feces from a single healthy human and then bred in a gnotobiotic isolator and found similar results of low human-to-mouse but high mouse-adapted-to-offspring mouse transfer efficiency at the ASV level (Figure S5A–C) [44]. The previously published analysis did not directly compare sequence variants between human donors and mouse recipients, as done in our re-analysis. These data demonstrate that a large fraction of the human microbiome does not efficiently engraft GF mice; however, once engrafting strains adapt to the mouse gut they transfer with very high efficiency in serial fecal transplant.

### Transfer efficiency varies between taxa

To assess the transfer efficiency of different taxa from transplant of human microbiota or mouse-adapted microbiota to GF mice, we compared Pearson correlation coefficients (*r*) between phyla (Fig. 5I–N). Unclassified bacteria had the lowest transfer efficiency in all groups, consistent with prior reports (Fig. 5I–N) [43]. *Verrucomicrobiota* and *Fusobacteriota* consistently had very high transfer efficiency, which likely reflected that a single species from each phylum was present in donor stool (Fig. 5I–N). *Akkermansia muciniphila*, a known keystone species, is the only human gut member of *Verrucomicrobiota* and is transferred highly efficiently across all transplant conditions and recipients. Transfer efficiencies trended lower for *Firmicutes, Bacteroidota,* and *Actinobacteriota* and trended somewhat lower for *Proteobacteria* in all *Il-10*^*−/−*^ mice compared to WT mice (Fig. 5I–N).

### Engrafted microbiota structure varies substantially by transplantation event for human but not mouse-adapted FMT

**Fig. 6:**
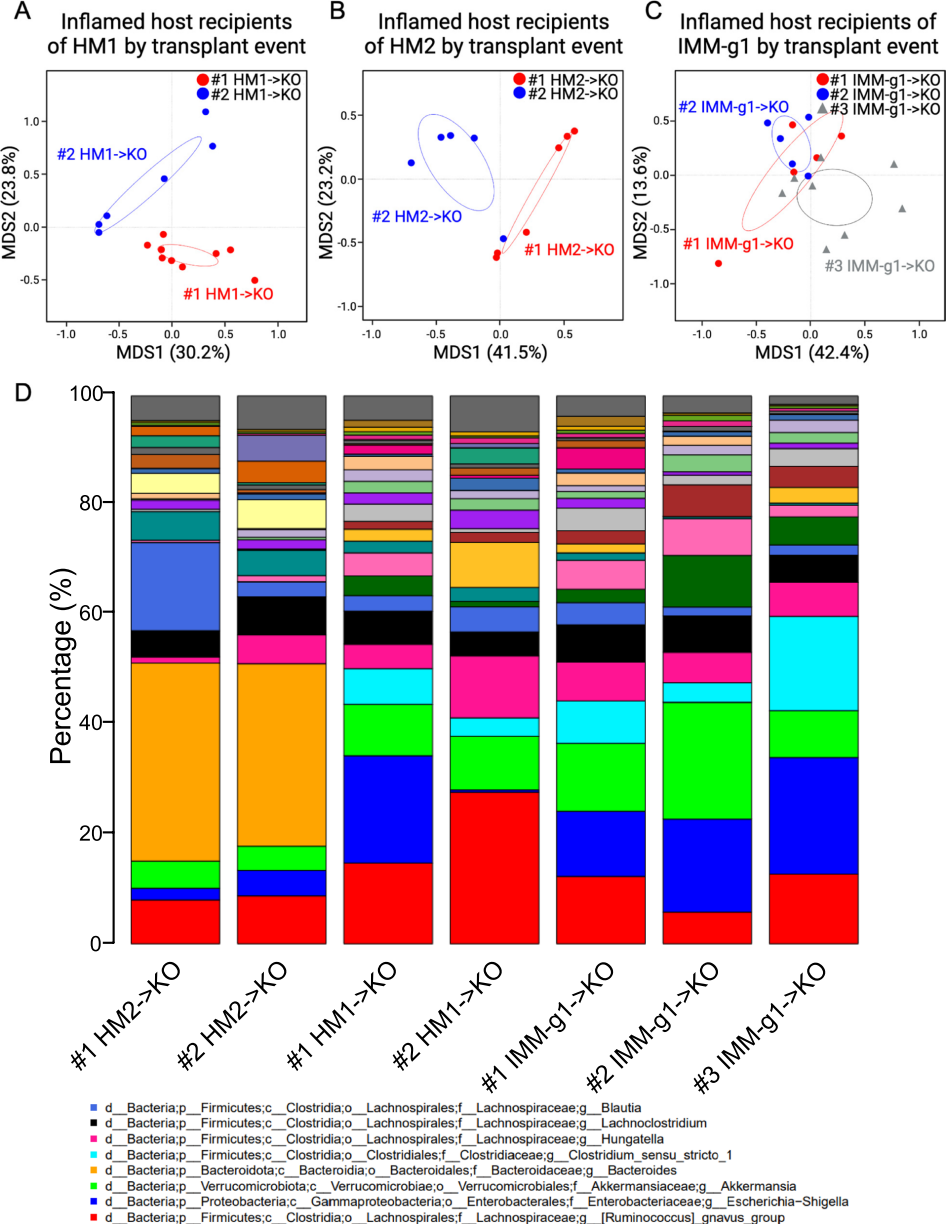
Microbiota engraftment varies with individual transplantation events for human-to-mouse fecal microbiota transplant but is more consistent with replicate mouse-adapted fecal microbiota transplants. **A**–**C** Multidimensional scaling (MDS) of 16S Seq data for FMT recipient *Il-10*^−/−^ (KO) mice for HM1- > KO (**A**), HM2- > KO (**B**), and IMM-g1- > KO (**C**) groups stratified by independent transplantation events where # represent individual experiments/transplantation events. **D** 16S Seq taxonomic bar plots show top 30 most abundant OTUs at the genus level in recipient mice feces at day 28 post-colonization grouped by independent transplantation events where # represent individual experiments / transplantation events. Legend shows top 8 genera. For mouse recipient groups, bar plots are average of 16S Seq data from *n* = 5–11 mice/group for each transplantation event

We speculated that the low transfer efficiency of human fecal microbiota to GF mice might drive stochastic variation of engrafted microbiota structure. We performed replicate fecal microbiota transplants using the same input microbiota at different times (individual transplantation events) and assessed engrafted microbiota variation by taxonomic bar plots and log_10_-ordinated PCoA. PCoA of GF *Il-10*^*−/−*^ mice transplanted with the same human input microbiota at different times demonstrated discrete clustering by transplantation event for both HM1 and HM2 (Fig. 6A, B). In contrast, the microbiota of GF *Il-10*^*−/−*^ mice transplanted with aliquots of the same mouse-adapted human input microbiota at different times was more closely clustered together by PCoA (Fig. 6C). These data demonstrate that mouse-adapted human IBD FMT engrafts a more consistent microbiota structure than human FMT across individual transplantation events. To identify genera driving differences in microbiota structure between multiple transplantation events of the same input microbiota, we visualized taxonomic bar plots for the top 30 most abundant genera across recipient *Il-10*^*−/−*^ mice grouped by transplantation event (Fig. 6D, Figure S6). The top 6–8 most abundant genera were broadly similar between individual transplant events for all FMT conditions (HM1, HM2, and IMM-g1) (Fig. 6D). By performing PCoA using log_10_-normalized data, we were able to visualize the large differences in lower abundance genera between transplantation events (Fig. 6A, B) since the log transformation places greater emphasis on the contribution of lower abundance genera. These data suggest that variable engraftment of lower abundance genera in human FMT drives variation in microbiota structure between individual transplantation events, and that mouse-adaptation largely ameliorates this variability to create a more rigorous and reproducible system.

### Inflamed mouse-adapted microbiome induces faster onset colitis than non-inflamed mouse-adapted microbiome

**Fig. 7:**
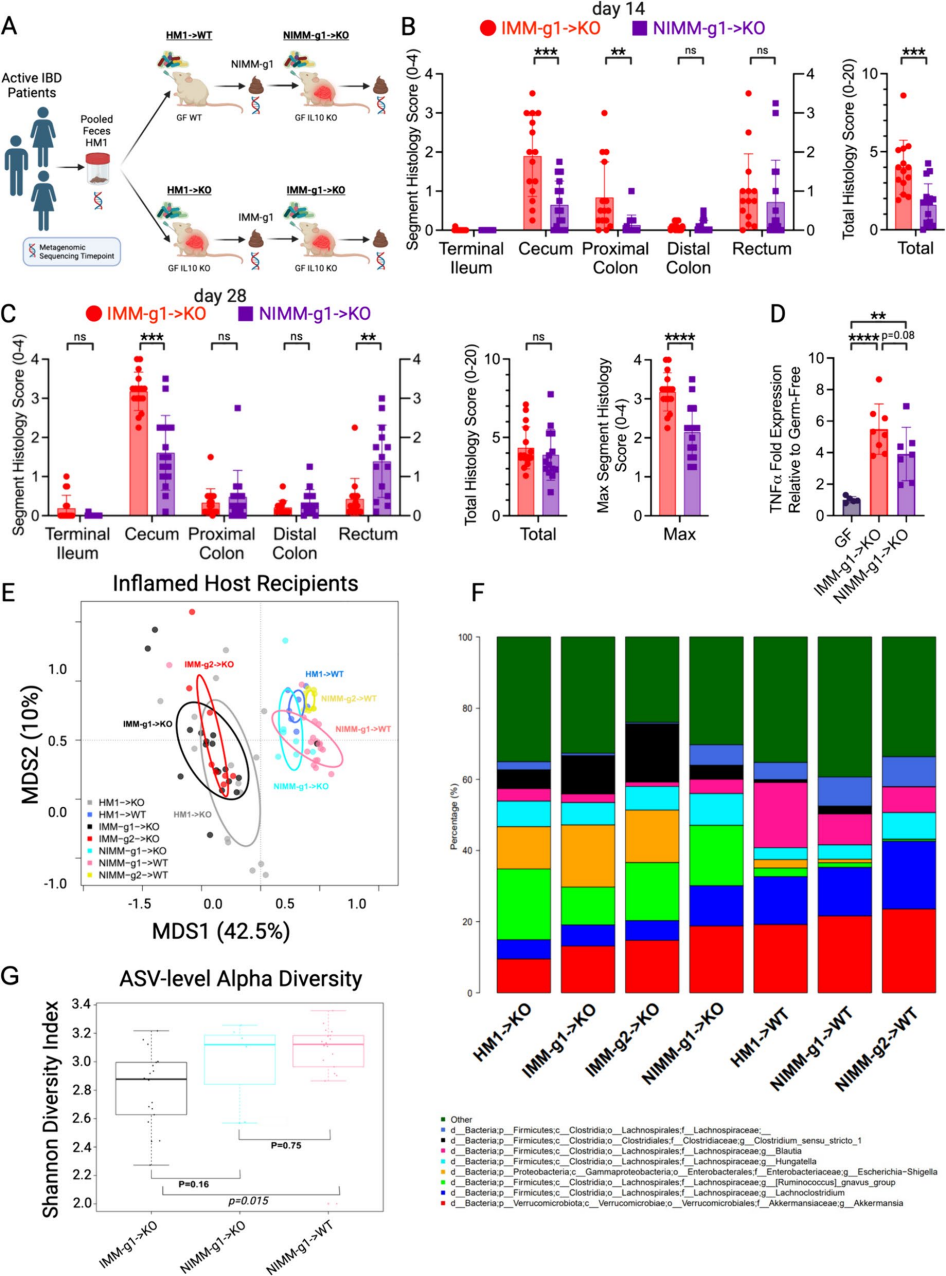
Inflamed mouse-adapted microbiome more rapidly induces severe colitis than non-inflamed mouse adapted microbiome. **A**. Experimental design. Human IBD patient microbiota (HM1) was adapted in the inflamed (IMM-g1) or non-inflamed (NIMM-g1) host, then transplanted to *Il-10*^−/−^ (KO) GF recipient mice. **B** Segment and total colon + ileum histology score for KO mice at day 14 post-colonization. **C** Segment, total colon + ileum, and max segment histology score for KO mice at day 28 post-colonization. **D** TNFα mRNA levels in cecal tissue at day 28 post-colonization. **E** PCoA of FMT recipient WT and KO mouse groups, including NIMM-g1- > KO group. **F** 16S Seq taxonomic bar plots show the top 8 most abundant genera in FMT inputs and recipient mouse feces at day 28 post-colonization. For mouse recipient groups, bar plots are average of 16S Seq data from *n* = 7–18 mice/group. **G** Shannon diversity index at ASV level for IMM-g1- > KO, NIMM-g1- > KO, and NIMM-g1- > WT groups. Data shown are representative of **D** or cumulative (**B**–**C**, **E**–**F**) from 2 to 4 independent experiments. *n* = 15–16 (**B**–**C**), *n* = 5–8 (**D**), *n* = 7–16 (**E**–**G**) mice per group. Data are expressed as mean±SD. Statistical significance calculated by unpaired *t*-test (**B**–**D**, **G**) with *p < 0.05, **p < 0.01, ***p < 0.001, ****p < 0.0001

Since mouse-adaptation of human microbiota in the non-inflamed (WT) host reduced the frequency of pathobionts while expanding putatively protective bacteria, we investigated whether NIMM-g1 induces less severe colitis than IMM-g1 when transplanted to *Il-10*^*−/−*^ GF mice (Fig. 7A). At 14 days post-colonization, NIMM-g1 colonized *Il-10*^*−/−*^ mice had significantly lower f-LCN2 levels, cecum- and total colon histologic inflammation than IMM-g1 colonized *Il-10*^*−/−*^ mice (Fig. 7B, Figure S7A–B). At 28 days post-colonization, NIMM-g1 colonized *Il-10*^*−/−*^ mice continued to have significantly reduced cecal inflammation scores and a trend toward lower cecal inflammatory cytokine levels but had developed increased rectal inflammation compared to IMM-g1 colonized *Il-10*^*−/−*^ mice (Fig. 7C). NIMM-g1 colonized *Il-10*^*−/−*^ mice had significantly lower maximum segment inflammation on a per-mouse basis compared to IMM-g1 colonized *Il-10*^*−/−*^ mice (Fig. 7C). However, the increase in rectal inflammation resulted in a non-significant trend toward lower f-LCN2 levels and no difference in total colon histology scores between NIMM-g1 and IMM-g1 colonized *Il-10*^*−/−*^ mice at 28 days post-colonization (Fig. 7C; Figure S7C). PCoA demonstrated that the microbiome of NIMM-g1 colonized *Il-10*^*−/−*^ mice (NIMM-g1- > KO) clustered with WT HMA and MA-FMT mice, rather than *Il-10*^*−/−*^ HMA or MA-FMT mice (Fig. 7E). Alpha diversity of NIMM-g1 colonized *Il-10*^*−/−*^ mice was equal to NIMM-g1 colonized WT mice and non-significantly higher than IMM-g1 colonized *Il-10*^*−/−*^ mice (Fig. 7G). These data suggest that major changes in community restructuring occur during the initial adaptation of human microbiota to the non-inflamed mouse host, but that once a stable mouse-adapted community forms it transfers with stable global structure in serial transplant to subsequently inflamed GF host mice. Although PCoA demonstrated that the global microbiome structure of NIMM was stable between the inflamed and non-inflamed environments, taxonomic bar plots and differential abundance analysis demonstrate that several taxa undergo changes in frequency (Fig. 7F; Figure S7D; Table S1). Putatively protective *Blautia* and *Lachnospiraceae NK4A136* group were significantly reduced while the pathobiont-containing genera *Ruminococcus gnavus* group and *Hungatella* were significantly expanded in *Il-10*^*−/−*^ compared to WT mice colonized with NIMM-g1, suggesting that these genera may be particularly responsive to the inflammatory environment—consistent with observations in human IBD microbiome profiling studies (Fig. 7F, Figure S7E–H, Table S1) [7, 9, 59, 60]. Together, these data demonstrate that human microbiome adaptation is dependent on the host environment, but once a stable mouse-adapted microbiome has been established it remains remarkably stable in composition despite an altered host environment.

## Discussion

The role of gut microbiota dysbiosis as cause or consequence of intestinal inflammation is an area of active investigation and debate with clinical importance for the management of IBD [1]. Transplanting human disease-associated feces to GF rodents is an approach that captures strain-specific functional and genetic variation responsible for human-microbiome-driven disease phenotypes without biased selection of defined input strains. Although widely accepted, this approach is complicated by phenotypic and experimental variability of unclear etiology. Our study identified that FMT transfer efficiency is an underappreciated source of experimental variability. Using high-depth, low-error rate Illumina 16S amplicon sequencing (16S Seq), we showed that pooled human IBD patient fecal microbiota engrafts GF mice with low ASV-level transfer efficiency, resulting in high recipient-to-recipient variation of microbiota composition and colitis severity in HMA *Il-10*^*−/−*^ mice. Human-to-mouse FMT caused a population bottleneck and ecological filter with reassembly of microbiota composition that was host inflammatory environment specific. In the inflamed environment of HMA *Il-10*^*−/−*^ mice, the microbiota reassembled with lower microbial alpha diversity, higher recipient-to-recipient microbiota compositional variability, and expansion of pathobionts compared to the distinct microbiota reassembled in the non-inflamed environment of HMA WT mice. Following the initial human-to-mouse population bottleneck and microbiota reassembly, the mouse-adapted human IBD patient microbiota transferred with high efficiency and low compositional variability to GF recipients, which correlated with highly consistent and reproducible colitis phenotypes in *Il-10*^*−/−*^ recipient mice. The mouse-adapted microbiota composition was remarkably stable in serial transplant to both inflamed and non-inflamed host environments. We replicated the key finding of low human-to-mouse but high mouse-adapted-to-mouse transfer efficiency at the ASV level by unique analysis, which was not originally performed in the initial publication, of an independently published 16S Seq dataset of HMA WT mice colonized with feces from a single healthy human and then bred for 1 generation in a gnotobiotic isolator [44]. By analyzing the engraftment of the same input microbiota over multiple individual experiments, we demonstrated that mouse-adapted human IBD FMT engrafts a more consistent microbiota structure than human FMT across independent transplantation events. Microbiota adaptation in the inflamed environment assembled a more aggressive microbiota than adaptation in the non-inflamed environment, demonstrating that the genetically determined host inflammatory environment shapes dysbiosis that subsequently drives more severe inflammation. Our data support that host gut inflammation is both a cause and consequence of microbial dysbiosis.

Our data support recent criticism that stochastic ecological processes and donor heterogeneity influence phenotypes in HMA murine models [61]. We found that OTU-based metrics, especially at higher taxonomic levels, over-estimated transfer efficiency compared to ASV analysis [23, 44, 61]. The low transfer efficiency and population bottleneck of human-to-mouse FMT led to high variability in engrafted microbiota composition between individual recipients of the same human input stool, which correlated with significant variability in colitis severity in recipient *Il-10*^*−/−*^ mice. Engraftment of human microbiota to GF mice varied significantly across independent transplantation events, in contrast to mouse-adapted microbiota which engrafted more consistently between separate experiments. We speculate that stochastic differences in engraftment were accentuated by the bottleneck of human-to-mouse FMT and drove phenotypic variability [61]. Large interindividual variability of human donor microbiota likely exacerbates this phenomenon in HMA murine studies [8, 57, 61]. We used pooled human IBD donor stool to mitigate the impact of individual human donor microbiota heterogeneity and replicated our results with 2 pooled human donor pools (HM1 containing UC and CD patients, HM2 containing only CD patients). Our pooling approach is suitable for experimental designs that require a representative human disease-associated microbiome to interrogate mechanistic questions (i.e., the impact of diet or host genetic background) or test therapeutics (i.e., live biotherapeutics or novel biologics); however, studies evaluating microbiome-driven phenotype transfer require an appropriately powered number of individual human donors to establish causality and avoid bias from pseudo-replication [61]. In our study, both pooled human fecal cohorts HM1 and HM2 contained *Bacteroides* genus at high abundance. However, following human-to-mouse transplant, *Bacteroides* abundance dramatically decreased in HM1 recipients but expanded in HM2 recipients. Although our study was not powered to distinguish whether this divergent engraftment arose from microbial ecology of the donor microbiota or stochastic processes, our data suggest that low human-to-mouse transfer efficiency in the setting of donor heterogeneity and stochastic ecological processes is an underappreciated source of variability in HMA animal models. In contrast, transplant of mouse-adapted human microbiota yielded highly reproducible and consistent microbiota composition and colitis phenotypes – an improved model for studying human microbiota-driven diseases.

Multiple factors may influence the variability of microbiota engraftment between independent transplantation events. These include changes in the external environment such as (1) mouse facility variation, (2) batch effects caused by different brands, or production changes from the same vendor, of feed, (3) season of the year, (4) variation of input handling including changes in anaerobic chamber oxygen contamination, and (5) prolonged freezing of samples. In addition to external factors, the genetic background of germ-free recipient mice can change over time due to genetic drift. Although we and other gnotobiotic experimentalists seek to strictly control these factors, our findings suggest that a major source of variability in human microbiota-associated mouse studies is the stochastic ecological filter of human-to-mouse microbiota transplant. Importantly, our study rigorously demonstrates that mouse-adaptation of human fecal microbiota significantly reduces experimental variability in subsequent ‘mouse-adapted-to-mouse’ FMT studies by improving bacterial transfer efficiency and phenotypic stability.

Our data demonstrated that mouse-adaptation of human fecal microbiota was shaped by the host inflammatory environment to form stable microbial communities that reproducibly engrafted GF mice with high efficiency to drive distinct colitis phenotypes. Mouse-adaptation in the inflamed genetically susceptible host assembled an aggressive microbiota with low alpha-diversity and high pathobiont abundance (*Enterobacteriaceae, R. gnavus*) that drove more rapid onset of colitis in serial transplant to *Il-10*^*−/−*^ mice than microbiota adapted in the non-inflamed host. *Firmicutes, Bacteroidota,* and *Actinobacteriota* transferred less efficiently to inflamed than uninflamed recipient hosts, while *Proteobacteria* transferred as efficiently to inflamed and uninflamed hosts. Gut inflammation induces host-derived metabolites, such as nitrate, lactate, and ethanolamine, that enhance fitness, abundance, and virulence of *Proteobacteria* such as resident adherent-invasive *E. coli* and promotes ectopic gut colonization of inflammation-associated *Veillonella* species [62–67]. Adherent and invasive *E. coli* and other inflammation-associated aggressive resident bacteria drive intestinal inflammation in murine colitis models [1, 18, 19, 21]. Together with the literature, our data support a model of IBD pathogenesis in which host inflammation in genetically susceptible hosts promotes the expansion, fitness, and virulence of aggressive resident bacteria, which further drives a feed-forward process of dysbiosis exacerbated gut inflammation. This model implies that effective management of IBD requires treating both the dysregulated host immune response and aggressive inflammation-associated microbiota.

Our study benefitted from several strengths including an experimental design that incorporated multiple serial FMT, independent replicate transplantation of the same human input microbiota to GF mice, high recipient mouse numbers, and application of high-depth low error rate sequencing for accurate ASV tracking; however, there were some limitations. First, most experiments were conducted in out-of-isolator gnotobiotic cages, where contamination risk is extremely low but could not be monitored due to the complex FMT inputs. To address this, we replicated key experiments in strict gnotobiotic isolators, confirming our findings of low human-to-mouse ASV-level transfer efficiency and the emergence of ASVs in HMA mice not detected in human input stool. Second, our study was performed in a single mouse strain background (129SvEv) and a single colitis model (*Il-10*^*−/−*^) due to resource constraints. To partially address this, we performed a unique re-analysis of a published study performed in germ-free C57BL/6NTac strain WT mice which replicated our key findings of low human-to-mouse but high mouse-adapted-to-mouse transfer efficiency. To test the generalizability of our finding that host recipient background shapes microbiota re-assembly with human-to-mouse FMT, further studies are required in other colitis models, such as naïve CD4^+^ T cell transfer to *Rag-1*^*−/−*^ mice, and disease models, such as the Leptin-deficient (Ob/Ob) obesity mouse model. Third, we did not analyze WGS data to compare the transfer efficiency of microbial functions vs taxonomic composition. We used 16S Seq rather than WGS because repeat sequencing of the same region allows for the exact identification of ASVs in a database-independent manner without reliance on classification algorithms. Future WGS studies are needed to evaluate the impact of taxonomic transfer efficiency on the transfer of microbial functions. Fourth, our study utilized feces as the transplant source, so we were unable to evaluate whether ecological niche, such as mucosal-associated bacteria, impacts microbiota assembly and transfer efficiency. Fifth, we did not evaluate the impact of mouse diet on the initial human-to-mouse ecological filter—an important topic for follow-up studies. Finally, although our group has previously demonstrated that healthy human fecal microbiota induces colitis when transplanted to GF *Il-10*^*−/−*^ mice [51], the present study did not compare the relative ability of healthy vs IBD patient fecal microbiota to induce inflammation in *Il-10*^*−/−*^ mice. Comparing healthy vs. IBD donor sources was beyond the scope of our study; however, fecal microbiotas from human IBD patients have been previously demonstrated to cause more severe colitis than healthy human microbiota when transplanted to GF mice using both the T-cell transfer to *Rag1*^*−/−*^ mouse and *Il10*^*−/−*^ mouse models of colitis [26, 39]. We did perform replicate studies of initial human-to-mouse transplantation using 2 pooled human IBD patient cohorts (HM1 and HM2) in independent duplicate studies for each human input microbiota. These replication studies with 2 separate donor cohorts demonstrated that the host inflammatory environment shapes the assembly of the engrafted microbiota.

## Conclusion

Our mouse-adapted human microbiota model is an optimized, reproducible, and rigorous system to study human microbiome-driven disease phenotypes. Multiple approaches (human microbiome profiling, defined consortia animal studies, HMA animal models) can investigate causality and identify mechanisms of microbiota-driven diseases [1, 29, 68]. Mono-association and defined consortium studies are reductionist approaches where a single variable (i.e., single-gene mutations) can interrogate bacterial mechanisms [1, 29]. Representative synthetic microbiota, such as hCOM2, PedsCom, and SIHUMI, provide a more ecologically complex system with known input strain identity and the ability to easily track relative abundance by simplified metagenomic sequencing approaches [28, 31, 32, 68, 69]. However, even large complex defined consortia do not capture the understudied strain level variation that exists in heterogeneous human resident microbiota and contributes to important differences in strain-dependent microbiota aggressiveness [22, 36, 37, 66]. The high transfer efficiency of mouse-adapted human microbiota transplant to GF mice improves phenotype consistency, experiment reproducibility, and rigor of mouse models of human microbiota-driven disease. Homogenous repositories of mouse-adapted human microbiota provide an identical microbial starting point for every experiment that can be replicated over time and between institutions/collaborators without the transfer of human host genetic material present in human feces to collaborators [70, 71]. Because of high transfer efficiency and reproducible engraftment, mouse-adapted human microbiota repositories can be expanded in vivo when stocks run low, mitigating the limitations of finite human fecal samples. While this study focused on colitis, our mouse-adapted human microbiota approach is a framework that may be generalized to mouse FMT models of other human microbiota-modulated diseases, such as metabolic syndrome/obesity, diabetes, autoimmune diseases, and cancer.

### Supplementary Information

Supplementary file 1 contains R and Python code used in our analyses. Supplementary Table S1 contains Differential abundance analysis between groups excluding genera present in less than 10% of the samples. The Supplemental Figures and Experimental Methods file contains supplemental figures with figure legends and supplemental experimental procedures.

## Data Availability

The datasets, sample meta-data, R code, and Python code are publicly available at https://github.com/anhmoss/Mouse-Adaptation-of-Human-Inflammatory-Bowel-Disease-Microbiota-Enhances-Colonization-Efficiency and from the corresponding authors upon reasonable request. Raw sequences are publicly available via the NCBI Sequence Read Archive under BioProject PRJNA1105425 with a detailed explanation of meta-data at the GitHub repository.
