# Supplementary material for: Mouse adaptation of human inflammatory bowel diseases microbiota enhances colonization efficiency and alters microbiome aggressiveness depending on the recipient colonic inflammatory environment
Source: Microbiome. 2024 Aug 7;12:147. doi: 10.1186/s40168-024-01857-2 (PMC11304999; doi:10.1186/s40168-024-01857-2)
Supplement: Supplementary file 2 — Supplementary file 1 contains R and Python code used in our analyses. Supplementary Table S1 contains Differential abundance analysis between groups excluding genera present in less than 10% of the samples. The Supplemental Figures and Experimental Methods file contains supplemental figures with figure legends and supplemental experimental procedures. [file 40168_2024_1857_MOESM1_ESM.zip › Supplemental_Figures_and_Experimental_Methods_ESM.pdf]

Mouse Adaptation of Human Inflammatory Bowel Diseases Microbiota Enhances Colonization Efficiency and Alters Microbiome Aggressiveness Depending on Recipient Colonic Inflammatory Environment

Simon M. Gray[1,3], Anh D. Moss[1,5], Jeremy W. Herzog[3], Saori Kashiwagi[3,6], Bo Liu[3], Jacqueline B. Young[5], Shan Sun[5], Aadra P. Bhatt[3,4], Anthony A. Fodor[2,5,*], R. Balfour Sartor[2,3,7,8,*]

[1]These authors contributed equally to this work.

[2]These authors contributed equally to this work.

[3]Center for Gastrointestinal Biology and Disease, Department of Medicine, Division of Gastroenterology and Hepatology, University of North Carolina at Chapel Hill, Chapel Hill, NC, USA

[4]Lineberger Comprehensive Cancer Center, University of North Carolina at Chapel Hill, Chapel Hill, NC, USA

[5]Department of Bioinformatics and Genomics, University of North Carolina at Charlotte, Charlotte, NC, USA

[6]Molecular Gastroenterology and Hepatology, Kyoto Prefectural University of Medicine, Kyoto, Japan

[7]Department of Microbiology and Immunology, University of North Carolina at Chapel Hill, Chapel Hill, NC, USA

[8]National Gnotobiotic Rodent Resource Center, University of North Carolina at Chapel Hill, Chapel Hill, NC, USA

*Correspondence: ryan_balfour_sartor@med.unc.edu. (R.B.S), afodor@uncc.edu (A.A.F)

Supplemental Experimental Methods

Gene expression by qRT-PCR

Tissues were immediately placed in RNAprotect cell reagent to stabilize RNA. Total RNA extraction from tissues (AllPrep PowerViral DNA/RNA Kit, Qiagen) and cDNA generation (iScript cDNA Synthesis Kit, Bio-Rad) were performed according to the manufacturer's protocols. Quantitative RT-PCR was performed on cDNA in duplicate or triplicate with a QuantStudio3 machine (ThermoFisher) using iTaq™ Universal SYBR Green Supermix (Bio-Rad). Target gene expression was quantified relative to internal control b-actin and expressed using the comparative Ct method ($2^{-\Delta\Delta Ct}$). qPCR primer sequences are: Tnfa-F 5'-ACCCTCACACTCAGATCATCTTCTC-3', Tnfa-R 5'-TGAGATCCATGCCGTTGG-3'. Actb-F 5'-AGCCATGTACGTAGCCATCCAG-3'; Actb-R 5'-TGGCGTGAGGGAGAGCATAG-3'

Intestine histopathology scoring

Small bowel and colon tissue sections were fixed in 10% phosphate-buffered formalin. Fixed tissue was paraffin-embedded, sectioned at 5μm thickness, and stained with hematoxylin and eosin by the UNC Center for Gastrointestinal Biology and Disease Histology Core. Histologic tissue inflammation was quantified by blinded scoring as previously described on a scale of 0-4 for 5 tissue segments (terminal ileum, cecum, proximal colon, distal colon, rectum)[1]. Total inflammatory score was the summation of the 5 segments. Max segment score was the single highest score from the 5 segments.

Fecal lipocalin-2 quantification:

Fecal samples (10-30mg) were homogenized in PBS with 0.1% Tween 20 and incubated at 4°C overnight, followed by centrifugation to pellet solid debris. Lipocalin-2 ELISA was performed on clear fecal supernatant according to manufacturer's instructions (DY1857, R&D Systems)[2].

Microbial and Statistical Analyses

16S rRNA amplicon (variable regions 3-4) PCR and sequencing were performed by the UNC Microbiome Core. Sequencing was performed on an Illumina MiSeq platform. Sequencing outputs were converted to fastq format and demultiplexed using Illumina Bcl2Fastq 2.20.0. The resulting paired-end

reads were processed with the QIIME2 2022-2 wrapper for DADA2 including merging paired ends, quality filtering, error correction, and chimera detection[3, 4]. Amplicon sequencing variants from DADA2 were assigned taxonomy with respect to the Silva databases, their sequences were aligned using maFFT in QIIME2, and a phylogenetic tree was built with FastTree in QIIME2[5-7].

Supplemental Experimental Methods References:

1. Rath HC, Herfarth HH, Ikeda JS, Grenther WB, Hamm TE, Jr., Balish E, et al. Normal luminal bacteria, especially Bacteroides species, mediate chronic colitis, gastritis, and arthritis in HLA-B27/human beta2 microglobulin transgenic rats. J Clin Invest. 1996;98(4):945-53.
2. Chassaing B, Srinivasan G, Delgado MA, Young AN, Gewirtz AT, Vijay-Kumar M. Fecal Lipocalin 2, a Sensitive and Broadly Dynamic Non-Invasive Biomarker for Intestinal Inflammation. PLoS One. 2012;7(9):e44328.
3. Caporaso JG, Kuczynski J, Stombaugh J, Bittinger K, Bushman FD, Costello EK, et al. QIIME allows analysis of high-throughput community sequencing data. Nat Methods. 2010;7(5):335-6.
4. Callahan BJ, McMurdie PJ, Rosen MJ, Han AW, Johnson AJA, Holmes SP. DADA2: High-resolution sample inference from Illumina amplicon data. Nat Methods. 2016;13(7):581-3.
5. Quast C, Pruesse E, Yilmaz P, Gerken J, Schweer T, Yarza P, et al. The SILVA ribosomal RNA gene database project: improved data processing and web-based tools. Nucleic Acids Research. 2013;41(D1):D590-D6.
6. Katoh K, Standley DM. MAFFT Multiple Sequence Alignment Software Version 7: Improvements in Performance and Usability. Molecular Biology and Evolution. 2013;30(4):772-80.
7. Price MN, Dehal PS, Arkin AP. FastTree 2 – Approximately Maximum-Likelihood Trees for Large Alignments. PLoS One. 2010;5(3):e9490.

SUPPLEMENTAL FIGURES and SUPPLEMENTAL FIGURE LEGENDS

Supplemental Figure S1. (related to Figure 1)

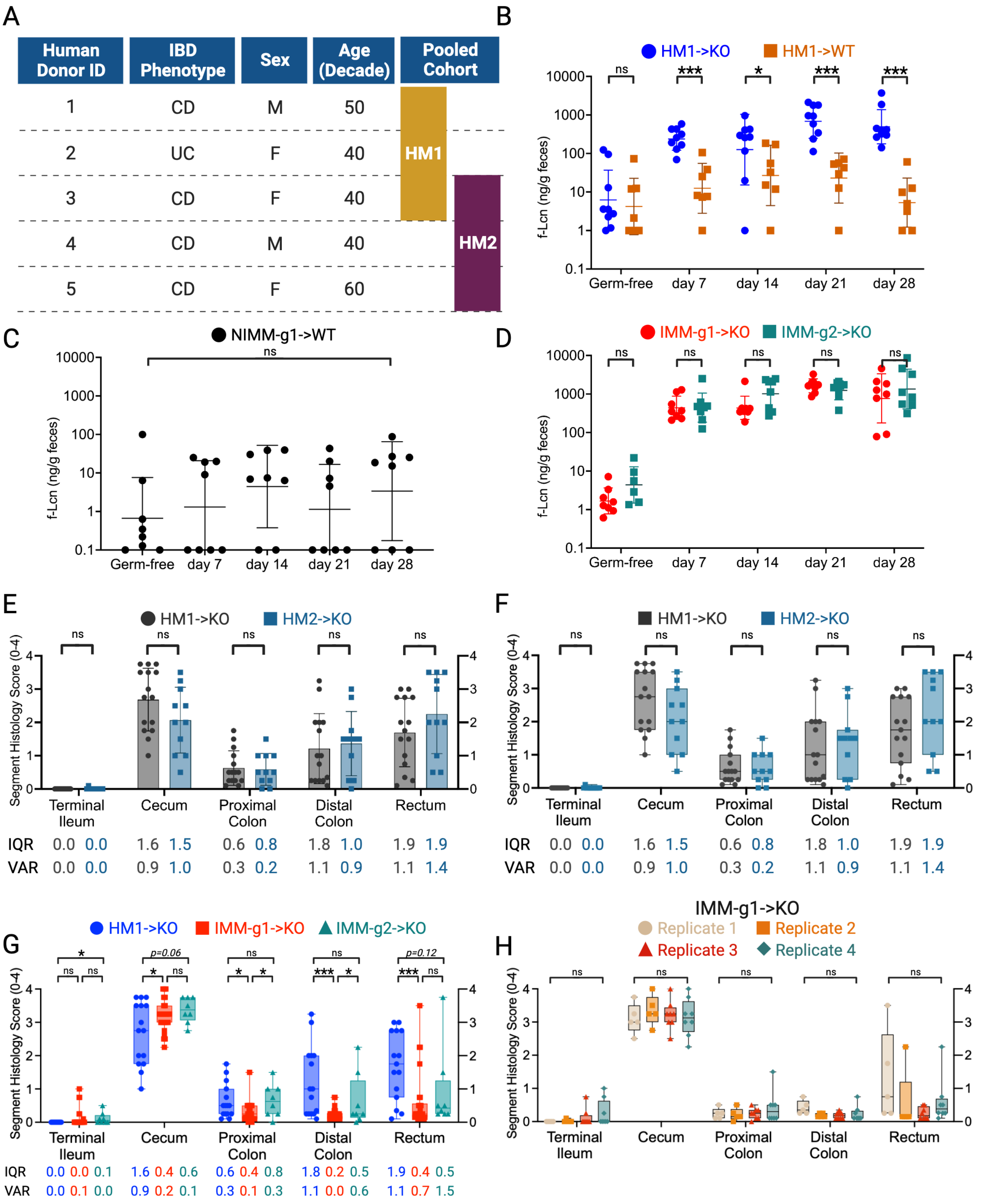

Supplemental Figure S1 (related to Figure 1). Mouse-adapted human microbiota induces more consistent and reproducible colitis than directly transplanted human microbiota. A) Demographics and cohort membership of human IBD donors. B-D) f-LCN2 time-course of (B) HM1 colonized WT and KO mice, (C) NIMM-g1 colonized WT mice, (D) IMM-g1 or IMM-g2 colonized KO mice. E-F) Bar plot (E) and Box-and-whisker plot (F) of segment histology score for HM1 and HM2 colonized KO mice at day 28 post-colonization. G) Box-and-whisker plot of segment histology score for KO mice at day 28 post-colonization. H) Box-and-whisker plot of segment histology score for IMM-g1 colonized KO mice at day 28 post-colonization from 4 independent experiments. Data shown are representative of (B-D, H) or cumulative (E-G) from 2-4 independent experiments. n=7-9 (B-D), n=11-15 (E-F), n=15-26 (G), n=5-8 (H) mice per group. Data are expressed as mean±SD (E) or geometric mean ± geometric SD (B-D). In box-and-whisker plots, box represents lower, median, and upper quartiles; whiskers are min to max. Statistical significance calculated by Mann-Whitney test (B-D) or unpaired t-test (E-H) with *$p<0.05$, **$p<0.01$, ***$p<0.001$.

Supplemental Figure S2. (related to Figure 2)

A

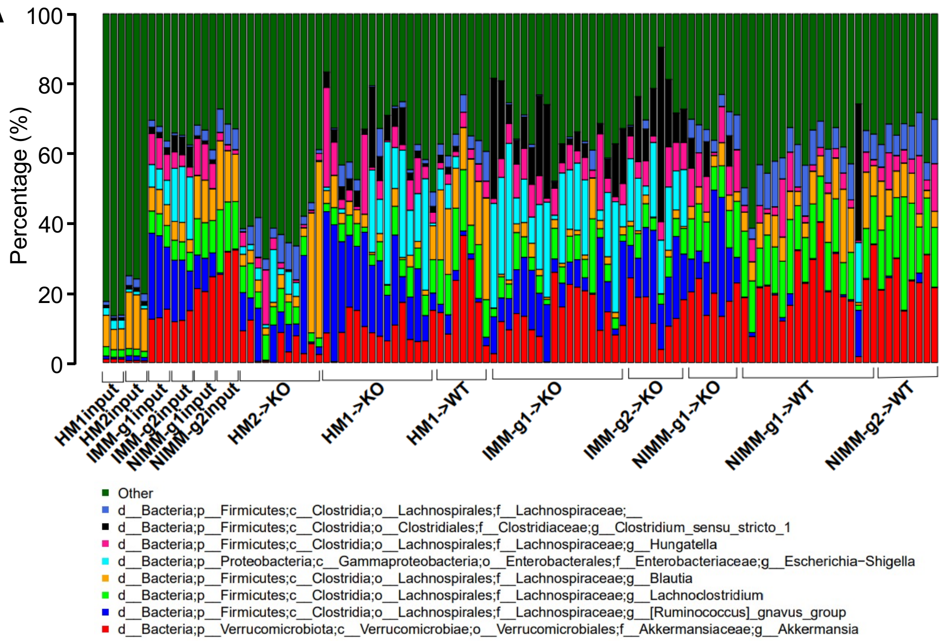

B

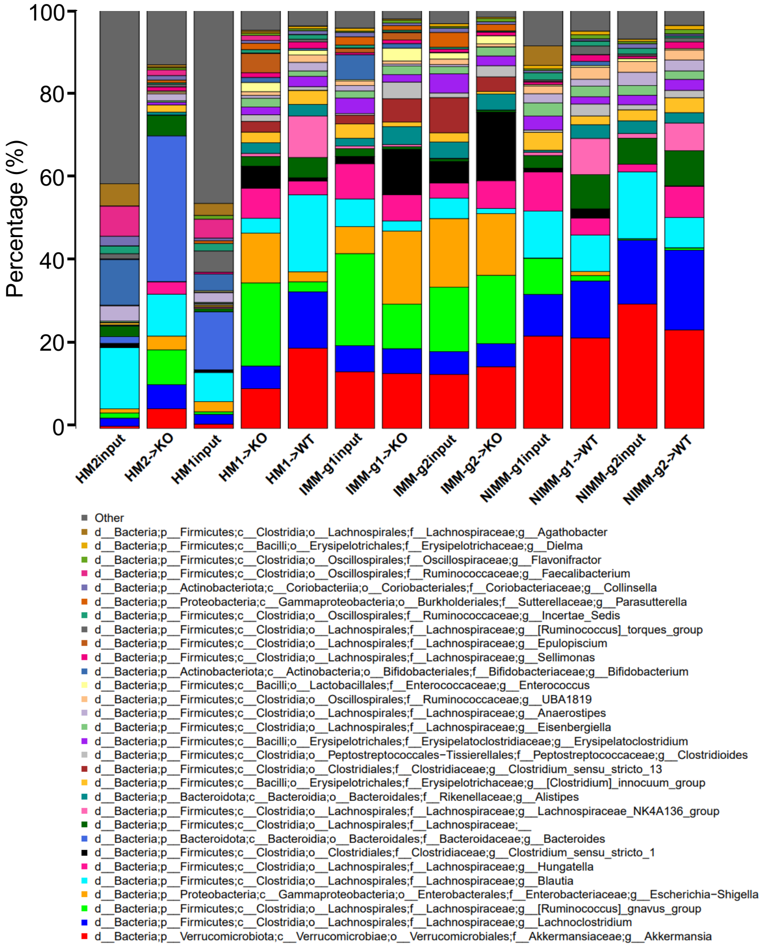

Supplemental Figure S2 (related to Figure 2). Recipient host environment influences engraftment composition of human-microbiome associated mice. A) 16S Seq taxonomic bar plots show top 8 most abundant genera in human and mouse-adapted FMT inputs and individual recipient mouse feces at day 28 post-colonization. B) 16S Seq taxonomic bar plots show top 30 most abundant genera in human and mouse-adapted FMT inputs and recipient mouse feces at day 28 post-colonization. For mouse recipient groups, bar plots are average of 16S Seq data from n=7-18 mice/group.

Supplemental Figure S3 (related to Figure 3).

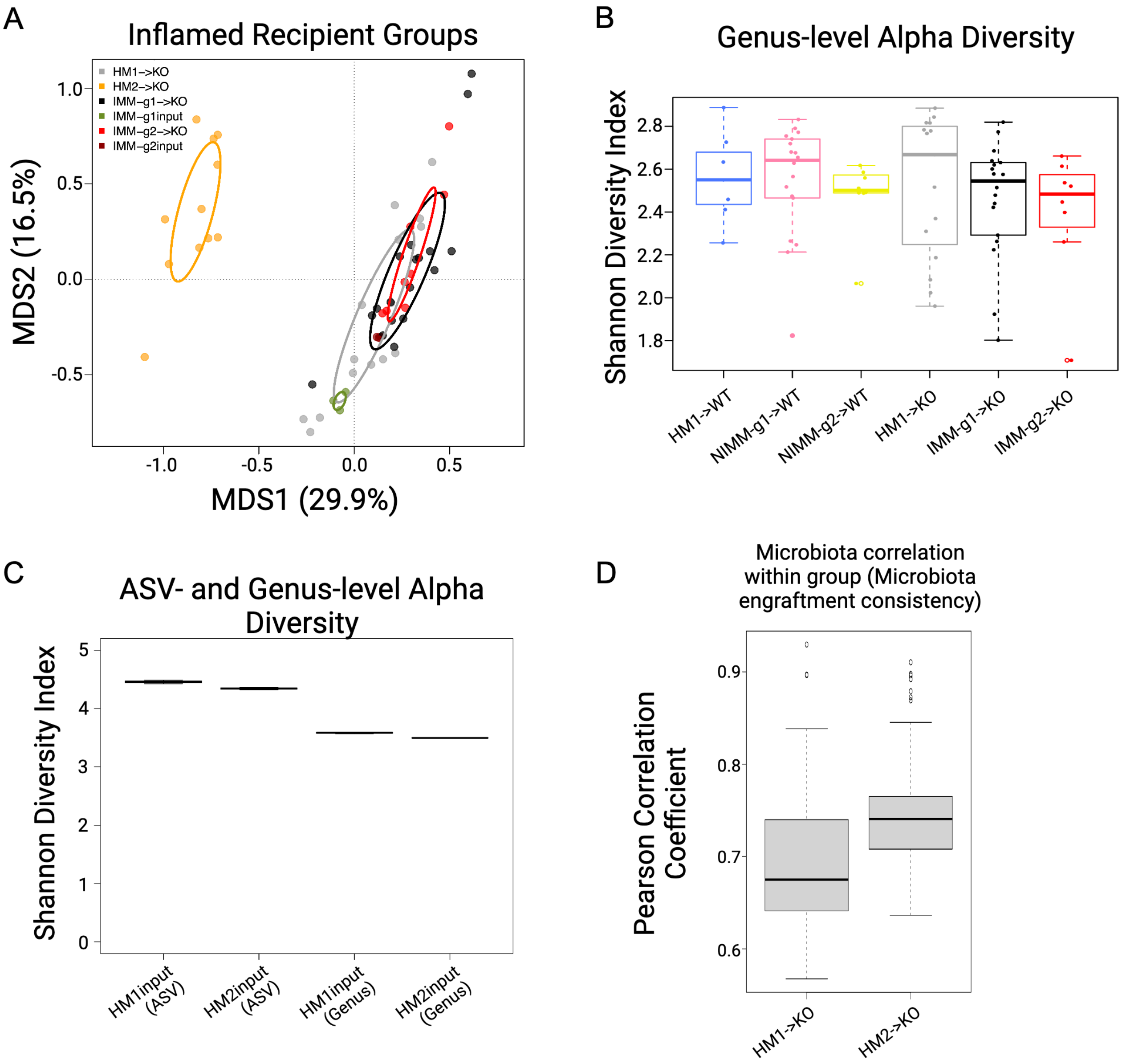

Supplemental Figure S3 (related to Figure 3). Human microbiome restructuring with transplant to GF mice is host inflammatory environment specific. A) PCoA of 16S Seq data for HM1, HM2, and mouse-adapted FMT inputs and FMT recipient KO mouse groups. B) Shannon index at genus level for FMT recipient WT and KO mouse groups. C) Shannon index at ASV and genus level for HM1 and HM2 FMT inputs. D) Pearson correlation coefficient (r) within group for HM1->KO and HM2->KO recipient mouse groups quantifies variability of microbiota composition between mice in the same group (microbiota engraftment consistency).

Supplemental Figure S4 (related to Figure 4).

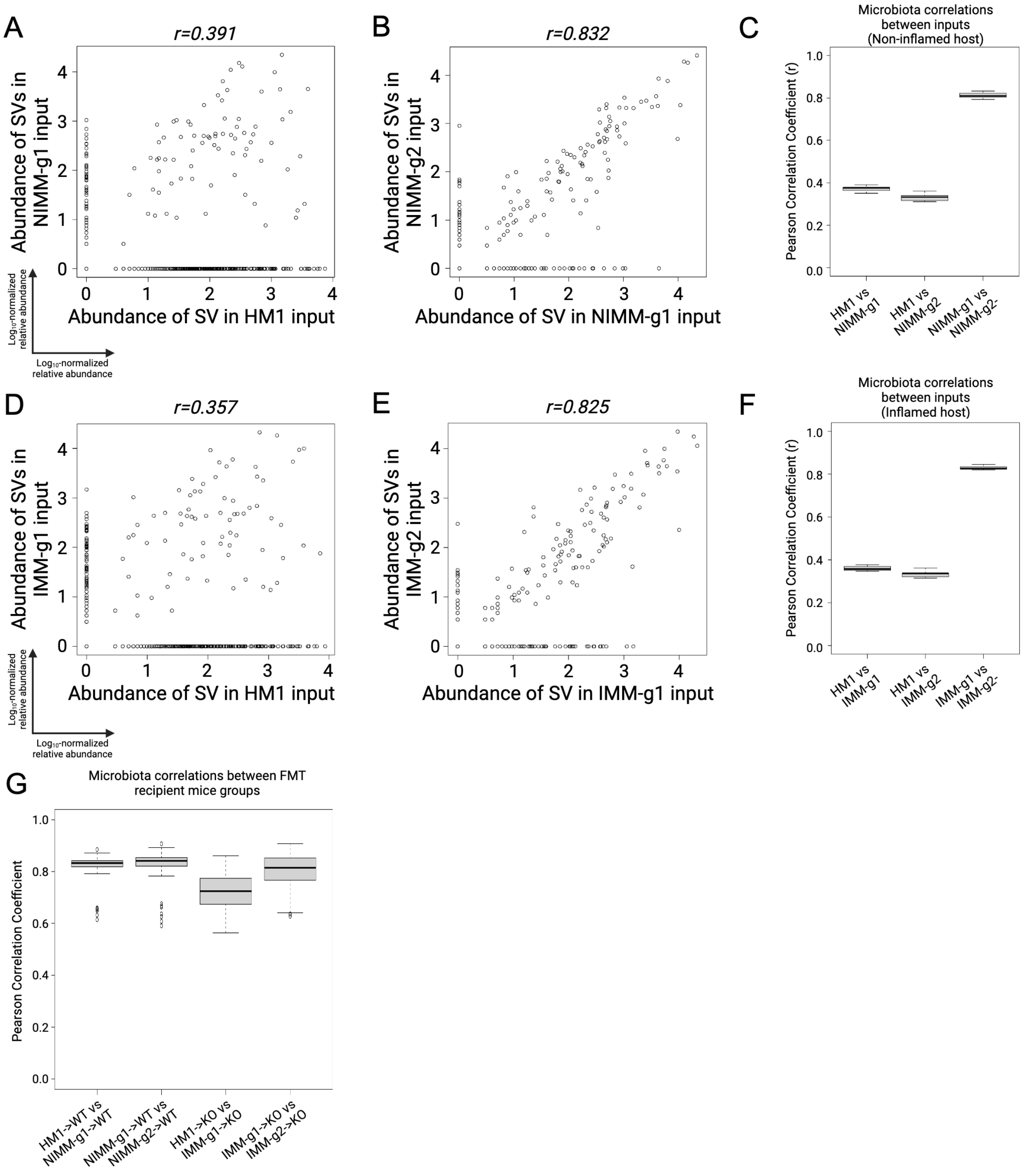

Supplemental Figure S4 (related to Figure 4). Mouse-adapted human IBD microbiota transfers with higher efficiency than human fecal transplant. A-B) ASV level log_{10}-normalized relative abundance correlations comparing (A) HM1 input to NIMM-g1 input and (B) NIMM-g1 input to NIMM-g2 input. C) Pearson correlation coefficient (r) between HM1 input, NIMM-g1 input, and NIMM-g2 inputs at the ASV level. D-E) ASV level log_{10}-normalized relative abundance correlations comparing (D) HM1 input to IMM-g1 input and (E) IMM-g1 input to IMM-g2 input. F) Pearson correlation coefficient (r) between HM1 input, IMM-g1 input, and IMM-g2 inputs at the ASV level. G) Pearson correlation coefficient (r) between WT recipient and KO recipient groups at the ASV level.

Supplemental Figure S5 (related to Figure 5).

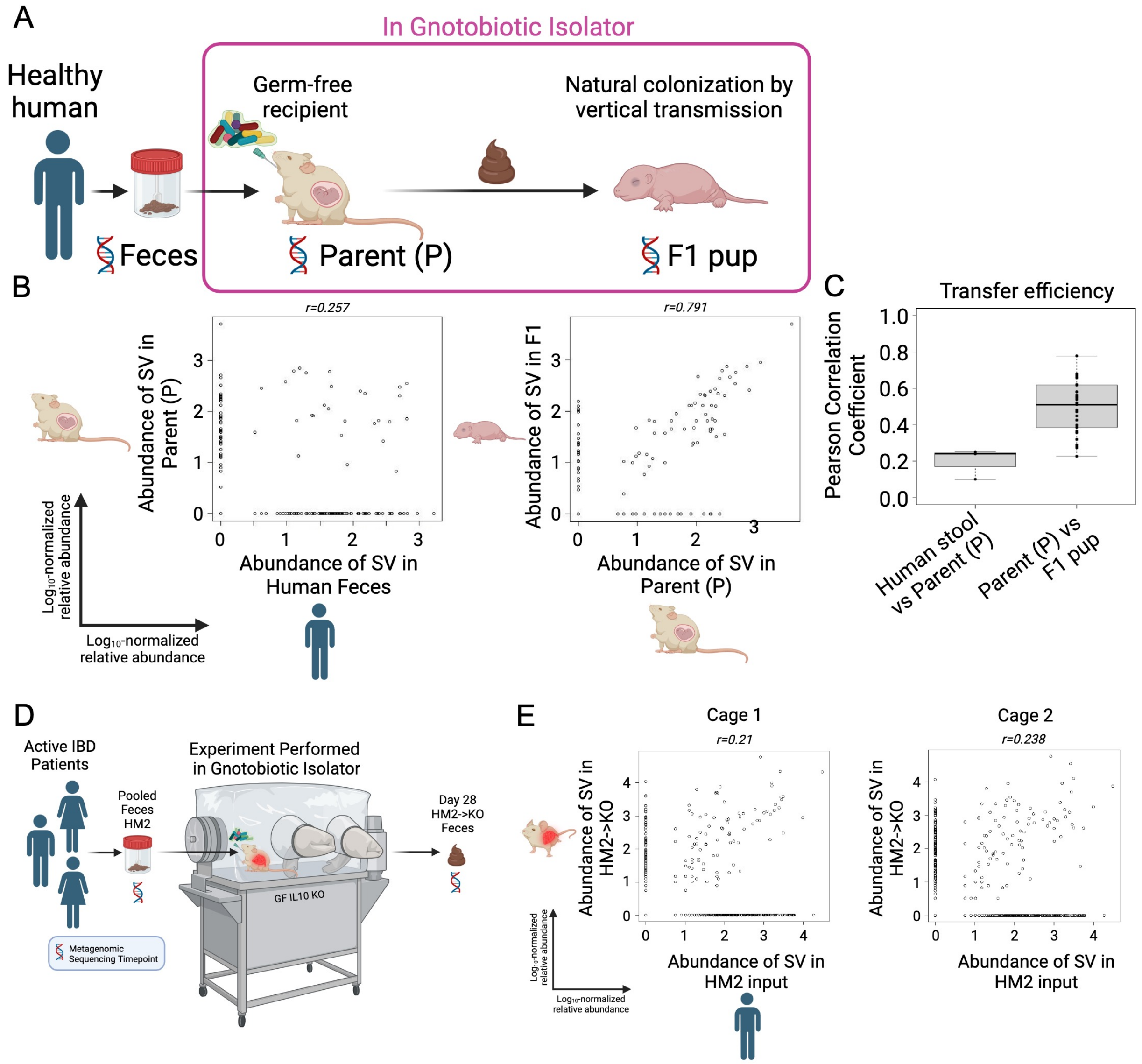

F

Percentage OTU Shared Between Donor Input and Recipient Group

Donor Input FMT	Recipient Group	% Shared ASV OTU	% Shared Genus OTU	% Shared Phylum OTU
HM1	HM1->WT	14.95%	31.36%	68.18%
HM1	HM1->KO	14.53%	30.73%	65.20%
HM2	HM2->KO	18.69%	32.25%	73.20%
IMM-g1	IMM-g1->KO	47.19%	69.63%	96.30%
IMM-g2	IMM-g2->KO	52.04%	80.91%	100%
NIMM-g1	NIMM-g1->WT	44.97%	71.93%	92.59%
NIMM-g2	NIMM-g2->WT	44.76%	84.74%	100%

Supplemental Figure S5 (related to Figure 5). Transfer efficiency varies between taxa. A-C) Re-analysis of published 16S Seq data from Lundberg *et al*.[44] (A) Experimental design. Feces from a single healthy human donor were transplanted to GF adult C57BL/6NTac strain WT mice (Parent, P) in a gnotobiotic isolator. HMA WT mice were bred in-isolator to generate F1 pups. B) ASV level log_{10}-normalized relative abundance correlations for FMT input and recipient mice. For F1 pups, the input was natural colonization by vertical transmission in-isolator from Parent (P). C) Transfer efficiency quantified by Pearson correlation coefficient (r) between FMT input and recipient mouse groups at the ASV level. D) Experimental design. Pooled feces from 3 humans with active IBD (3 CD, HM2) were transplanted to colitis-susceptible *Il-10*$^{-/-}$ (KO) GF recipient mice in a gnotobiotic isolator. E) ASV level log_{10}-normalized relative abundance correlations for HM2 input and HM2->KO recipient mice. F) Table comparing percentage shared OTU between input and recipient group at the ASV, Genus, and Phylum level.

Supplemental Figure S6 (related to Figure 6).

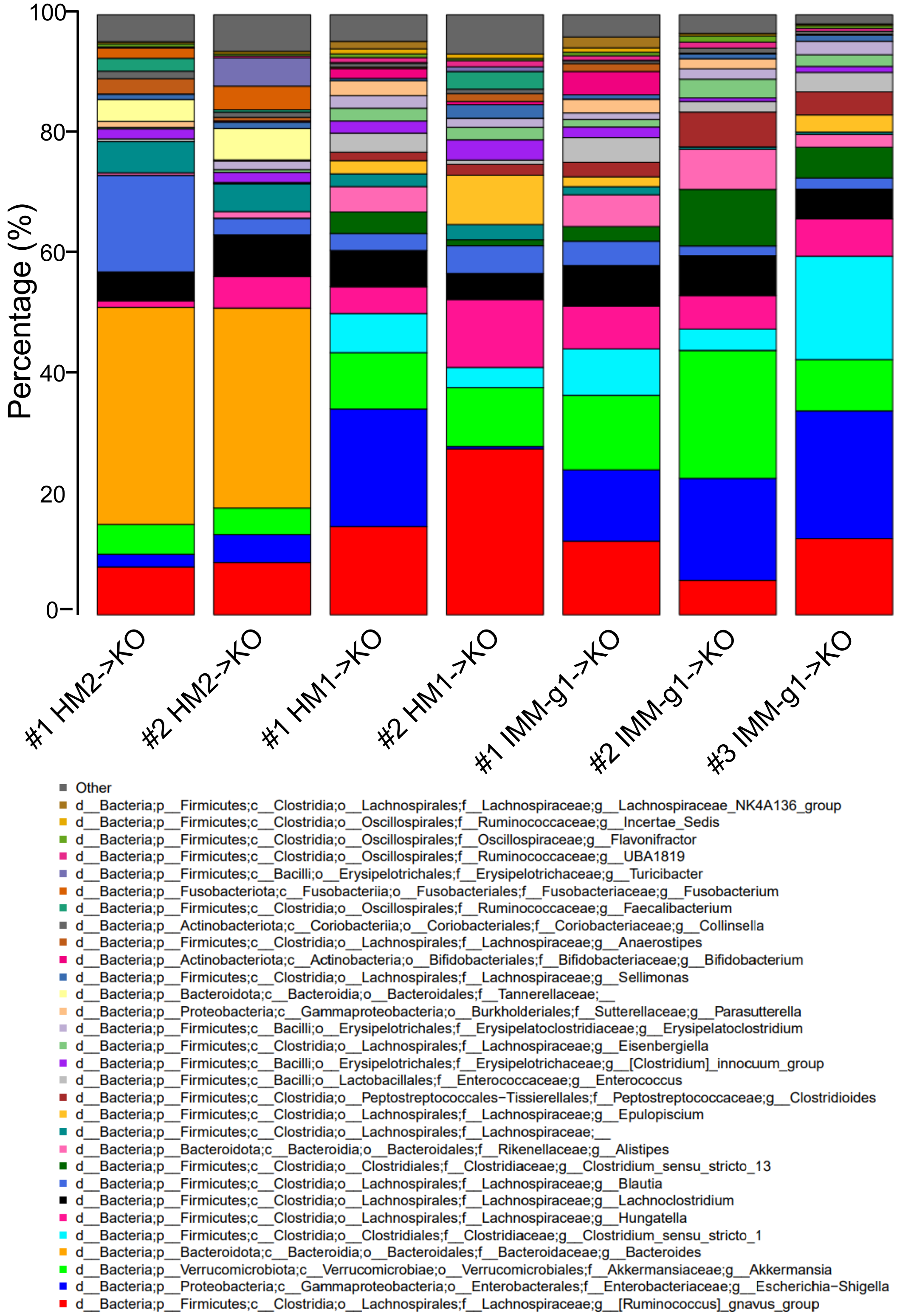

Supplemental Figure S6 (related to Figure 6). Human-to-mouse microbiota engraftment varies with individual transplantation events and is improved by mouse-adapted fecal microbiota transplant. A) 16S Seq taxonomic bar plots show top 30 most abundant OTUs at the genus level in recipient mice feces at day 28 post-colonization grouped by independent transplantation events where # represent individual experiments / transplantation events. Legend shows top 30 genera. For mouse recipient groups, bar plots are average of 16S seq data from n=5-11 mice/group for each transplantation event.

Supplemental Figure S7 (related to Figure 7).

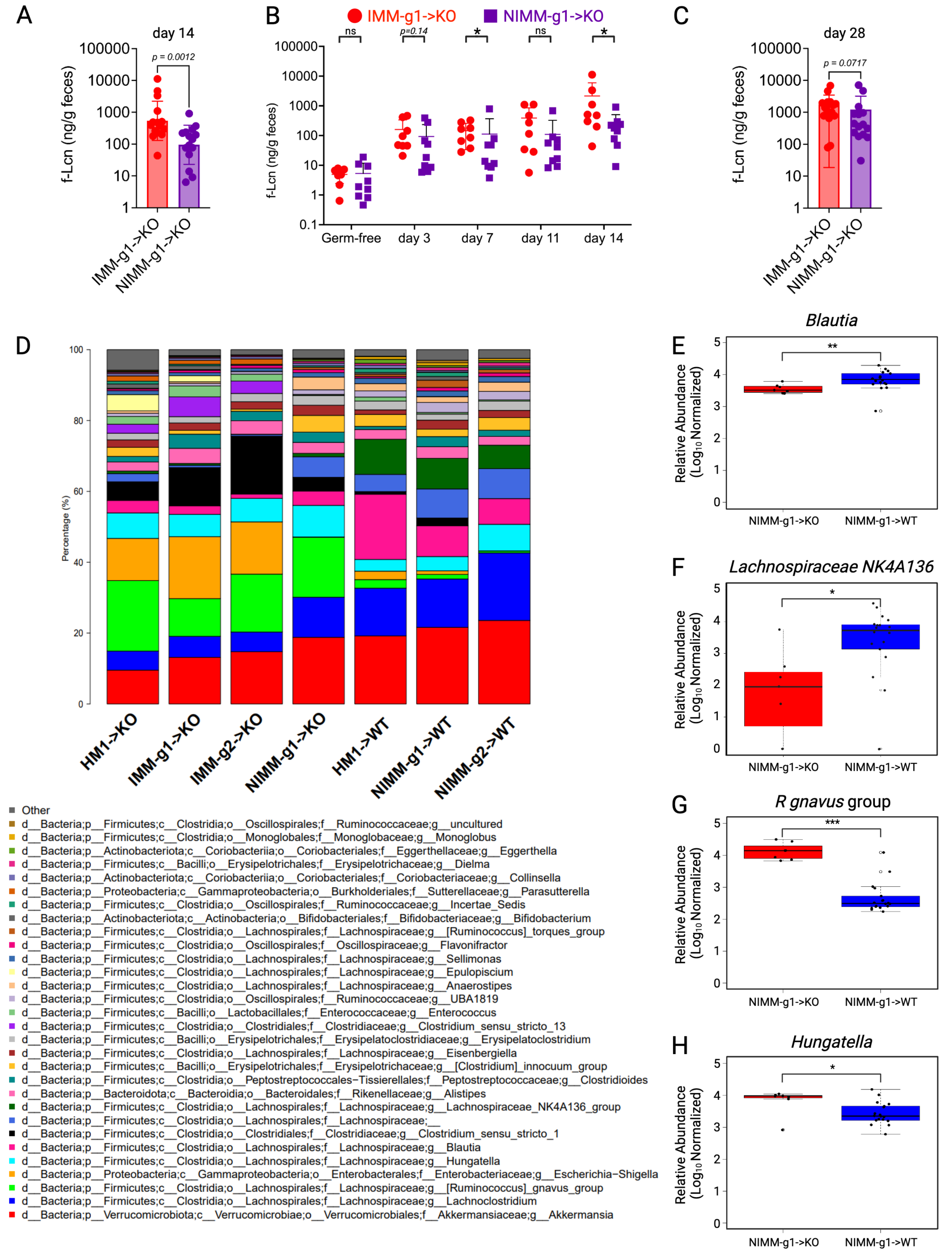

Supplemental Figure S7 (related to Figure 7). Inflamed mouse-adapted microbiome more rapidly induces severe colitis than non-inflamed mouse adapted microbiome. A-C) f-LCN2 level of IMM-g1->KO and NIMM-g1->KO mice at (A) day 14, (B) time-course from day 0 to day 14, and (C) day 28 post-colonization. D) 16S Seq taxonomic bar plots show top 30 most abundant genera in human and mouse-adapted FMT inputs and recipient mouse feces at day 28 post-colonization. E-H) $\log_{10}$-normalized relative abundance of (E) *Blautia,* (F) *Lachnospiraceae NK4A136* group, (G) *R gnavus* group, (H) *Hungatella* in NIMM-g1->KO vs NIMM-g1->WT mice. For mouse recipient groups, bar plots are average of 16S Seq data from n=7-18 mice/group. Data shown are cumulative (A) or representative (B-C) of 2 independent experiments. n=15-16 (A), n=8-9 (B), n=7-8 (C) mice per group. Data are expressed as geometric mean ± geometric SD (A-C). Statistical significance calculated by Mann-Whitney test (A-C) or unpaired t-test (E-H) with *p<0.05, **p<0.01, ***p<0.001.
