# Supplementary material for: Mouse adaptation of human inflammatory bowel diseases microbiota enhances colonization efficiency and alters microbiome aggressiveness depending on the recipient colonic inflammatory environment
Source: Microbiome. 2024 Aug 7;12:147. doi: 10.1186/s40168-024-01857-2 (PMC11304999; doi:10.1186/s40168-024-01857-2)

Analysis of FMT Model for Humanized Mice

Introduction

In our examination of a more stable and reproducible fecal microbiome transfer (FMT) model between human and mice, we used 16S rRNA amplicon sequence data of stool samples from both humans and mice throughout various iterations of fecal transfers. The different iterations of fecal transfers include generational, serial transfers (human to mouse, human-adapted mouse to mouse) and in two different host environments (inflamed and non-inflamed).

This notebook includes R scripts that generated PCoA plots, boxplots, and scatterplots presented in the published manuscript.

Taxonomic barplots and alpha diversity plots were generated through the Plotmicrobiome application.

A core set of figures from the manuscript were independently replicated by Jacqueline B. Young using Python. Both the Python scripts and replicated figures are provided in a Jupyter Notebook file (PDF format).

The Github link to the Plotmicrobiome application and the Python validation files are provided at the end of our manuscript.

Please refer to the ReadMe file on our Github respository for additional notes on running the scripts in this R notebook.

Analysis Overview

Each code chunk follows a step of the analysis pipeline:

- Installing all relevant libraries
- Data import and parsing
- PCoA Plots
- Transfer efficiency calculations of samples within a given FMT group
- Transfer efficiency calculations of samples across a pair of FMT groups
- Visualizing the non-transferred ASV abundance in samples across a pair of FMT groups
- Testing homogeneity of variances among histology tissue scores
- Assessing cage effect on transfer efficiency of samples within groups
- Assessing protein assay data (fLCN2)
- Control Experiment: Human to Mice in a gnotobiotic isolator

Installing libraries

The code below insalls and loads all dependencies used throughout this analysis.

```r
packagesToInstall = c("ggplot2", "vegan", "dplyr", "testthat", "stats","car","here")
install.packages(packagesToInstall,repos = "http://cran.us.r-project.org")
lapply(packagesToInstall, library, character.only=TRUE)
```

Data import and parsing

Next, the script below reads in all the functions that are used throughout this analysis.

We recommend organizing these functions, which exist as external, independent R scripts, in the same directory as the R Notebook file.

```r
allScripts = c("lognorm_function.R","generatePCOAPlots.R",
               "getCorrelationCoefficient_acrossGroup.R",
               "getCorrelationCoefficient_withinGroup.R",
               "getCorrelationCoefficient_withinGroup_phylumLevel_byGroup.R",
               "getCorrelationCoefficient_withinGroup_perCage.R",
               "generateTableWithTaxonomy_NontransferringASVS_samplePairsAcrossGroup.R",
               "generateScatterPlots_samplePairsAcrossGroup_histogramOfNontransferringASVS.R",
               "calculateBartlettPvalue_histologyScoreData.R","calculateLevenesTestPvalue_histologyScoreData.R",
               "generateScatterplots_acrossGroup.R"
               )

for(i in 1:length(allScripts)){
  source(allScripts[i], local = knitr::knit_global())
}
```

The code below reads in the counts data and metadata files, starting with the counts data at the ASV level, with a total sample size 110 sequenced samples. This includes three technical replicates of pooled human donor slurries and for each input mice slurry group.

QIIME2 generates counts table in a feature x sample format, in which features (taxa) are in rows and samples are columns. In our first step of reading in these counts data files into our R scripts, we maintained the format integrity (features as rows and samples as columns). Later, we transpose the counts table to match the format of the metadata file, which has samples as rows and features as columns.

Note: Early in our analysis, we updated the nomenclature system of our FMT groups to improve readability. Hence, we provide the metadata with the original and the updated nomenclature, the latter which we use for our published results and manuscript. In addition to updating nomenclature, which we updated in Excel, we formatted the sample names to make it R-friendly (i.e. change "-" into "." in sample names because R interprets "-" between integers as a mathematics operation). If original counts tables are read into Python, these changes are not needed. The Python validation scripts uses the metadata and counts files with the original nomenclature.

```r
file_asv_updatedName = read.table(here("files","countsTable_asv_importToR_updatedNomenclature.txt"), header = T, sep = "\t")
metamapping_updatedName = read.table(here("files","mappingMetadata_updatedNomenclature.txt"), header = T, sep = "\t")
asv_taxonomy_map= read.table(here("files","asv_taxonomy_map.txt"), sep = "\t", header = TRUE)

## group labels
labels_inputRecipientGroups = unique(metamapping_updatedName$FMTGroupFMTsource..Recipientbackground)

file_asv_updatedName_t = t(file_asv_updatedName)
file_asv_updatedName_t_samplesOnly = file_asv_updatedName_t[-(c(1,nrow(file_asv_updatedName_t))),]
colnames(file_asv_updatedName_t_samplesOnly) = file_asv_updatedName_t[1,]


file_asv_updatedName_t_samplesOnly_toMerge = as.data.frame(file_asv_updatedName_t_samplesOnly)
file_asv_updatedName_t_samplesOnly_toMerge$SampleID = rownames(file_asv_updatedName_t_samplesOnly)

#merge counts and metadata files
myASVandMetadata_newNames = full_join(file_asv_updatedName_t_samplesOnly_toMerge, metamapping_updatedName, by="SampleID")

names(myASVandMetadata_newNames)[names(myASVandMetadata_newNames)=="FMTGroupFMTsource..Recipientbackground"] = "FMTGroupFMTsourcegtRecipientbackground"

##lognorm
file_asv_updatedName_t_samplesOnly_df = as.data.frame(file_asv_updatedName_t_samplesOnly)
file_asv_updatedName_t_samplesOnly_df[] <- lapply(file_asv_updatedName_t_samplesOnly_df, function(x) as.numeric(as.character(x)))

file_asv_updatedName_t_samplesOnly_lognorm = lognorm_function(file_asv_updatedName_t_samplesOnly_df)

# serial passage groups
labels_NIMM_lineage = c("HM1input", "HM1->WT", "NIMM-g1input", "NIMM-g1->WT", "NIMM-g2input", "NIMM-g2->WT")
labels_IMM_lineage = c("HM1input", "HM1->KO", "IMM-g1input", "IMM-g1->KO","IMM-g2input", "IMM-g2->KO")
```

The code below parses and log normalizes the genus counts table:

```r
file_genusCountsTable_updatedName = read.table(here("files","countsTable_genus_importToR_updatedNomenclature.txt"), header = T, sep = "\t")
metamapping_updatedName = read.table(here("files","mappingMetadata_updatedNomenclature.txt"), header = T, sep = "\t")

file_genusCountsTable_updatedName_t = t(file_genusCountsTable_updatedName)

genusCountsTable_samplebyfeatureFormat = file_genusCountsTable_updatedName_t[-1,]
colnames(genusCountsTable_samplebyfeatureFormat) = file_genusCountsTable_updatedName_t[1,]

genusCounts_samplesOnly_toMerge = as.data.frame(genusCountsTable_samplebyfeatureFormat)
genusCounts_samplesOnly_toMerge$SampleID = rownames(genusCountsTable_samplebyfeatureFormat)

#merge counts and metadata files
genusCountsandMetadata = full_join(genusCounts_samplesOnly_toMerge, metamapping_updatedName, by="SampleID")

names(genusCountsandMetadata)[names(genusCountsandMetadata)=="FMTGroupFMTsource..Recipientbackground"] = "FMTGroupFMTsourcegtRecipientbackground"

##lognorm
genusCounts_samplesOnly_df = as.data.frame(genusCountsTable_samplebyfeatureFormat)
genusCounts_samplesOnly_df[] <- lapply(genusCounts_samplesOnly_df, function(x) as.numeric(as.character(x)))

genusCounts_samplesOnly_df_lognorm = lognorm_function(genusCounts_samplesOnly_df)
```

Below, the code parses and log normalizes the phylum counts table:

```r
file_phylumCountsTable_updatedName = read.table(here("files","countsTable_phylum_importToR_updatedNomenclature.txt"), header = T, sep = "\t")
metamapping_updatedName = read.table(here("files","mappingMetadata_updatedNomenclature.txt"), header = T, sep = "\t")

file_phylumCountsTable_updatedName_t = t(file_phylumCountsTable_updatedName)

phylumCountsTable_samplebyfeatureFormat = file_phylumCountsTable_updatedName_t[-1,]
colnames(phylumCountsTable_samplebyfeatureFormat) = file_phylumCountsTable_updatedName_t[1,]

phylumCounts_samplesOnly_toMerge = as.data.frame(phylumCountsTable_samplebyfeatureFormat)
phylumCounts_samplesOnly_toMerge$SampleID = rownames(phylumCountsTable_samplebyfeatureFormat)

#merge counts and metadata files
phylumCountsandMetadata = full_join(phylumCounts_samplesOnly_toMerge, metamapping_updatedName, by="SampleID")

names(phylumCountsandMetadata)[names(phylumCountsandMetadata)=="FMTGroupFMTsource..Recipientbackground"] = "FMTGroupFMTsourcegtRecipientbackground"

##lognorm
phylumCounts_samplesOnly_df = as.data.frame(phylumCountsTable_samplebyfeatureFormat)
phylumCounts_samplesOnly_df[] <- lapply(phylumCounts_samplesOnly_df, function(x) as.numeric(as.character(x)))

phylumCounts_samplesOnly_df_lognorm = lognorm_function(phylumCounts_samplesOnly_df)
```

PCoA Plots

PCoA plots were generated with the capscale function from the vegan package using Bray-Curtis dissimilarity matrix.

```r
dir.create(here("PcoaPlots"))

plotColors_ver2 = c("gray65", "royalblue1", "green", "orange", "purple",
                    "black", "olivedrab", "red", "red4",
                     "cyan","palevioletred1", "blue3", "yellow2", "magenta")


metagroup = factor(myASVandMetadata_newNames$FMTGroupFMTsourcegtRecipientbackground)

micegroups_all = c("HM1->KO", "HM1->WT", "IMM-g1->KO", "IMM-g2->KO", "NIMM-g1->WT","NIMM-g2->WT")
micegroups_inflamed = c("HM1->KO", "IMM-g1->KO","IMM-g2->KO", "IMM-g1input", "IMM-g2input")
micegroups_noninflamed = c("HM1->WT", "NIMM-g1->WT","NIMM-g2->WT", "NIMM-g1input", "NIMM-g2input")
excludecrossoverGroup = unique(genusCountsandMetadata$FMTGroupFMTsourcegtRecipientbackground)
excludecrossoverGroup = excludecrossoverGroup[-11]
micegroups_inflamed_withbothHMCohorts = c("HM1->KO", "HM2->KO","IMM-g1->KO","IMM-g2->KO", "IMM-g1input", "IMM-g2input")
micegroups_serialTransfersPlusCrossover = c("HM1->KO", "HM1->WT", "IMM-g1->KO", "IMM-g2->KO", "NIMM-g1->WT","NIMM-g2->WT", "NIMM-g1->KO")

micegroups_all.samplesindex = genusCountsandMetadata$SampleID[genusCountsandMetadata$FMTGroupFMTsourcegtRecipientbackground %in% micegroups_all]
micegroups_inflamed.samplesindex = genusCountsandMetadata$SampleID[genusCountsandMetadata$FMTGroupFMTsourcegtRecipientbackground %in% micegroups_inflamed]
micegroups_noninflamed.samplesindex = genusCountsandMetadata$SampleID[genusCountsandMetadata$FMTGroupFMTsourcegtRecipientbackground %in% micegroups_noninflamed]
excludcrossoverGroup.samplesindex = genusCountsandMetadata$SampleID[genusCountsandMetadata$FMTGroupFMTsourcegtRecipientbackground %in% excludecrossoverGroup]
micegroups_inflamed_withbothHMCohorts.samplesindex = genusCountsandMetadata$SampleID[genusCountsandMetadata$FMTGroupFMTsourcegtRecipientbackground %in% micegroups_inflamed_withbothHMCohorts]
micegroups_serialTransfersPlusCrossover.samplesindex = genusCountsandMetadata$SampleID[genusCountsandMetadata$FMTGroupFMTsourcegtRecipientbackground %in% micegroups_serialTransfersPlusCrossover]

micegroups_all.metagroup = factor(metagroup[metagroup %in% micegroups_all])
micegroups_inflamed.metagroup = factor(metagroup[metagroup %in% micegroups_inflamed])
micegroups_noninflamed.metagroup = factor(metagroup[metagroup %in% micegroups_noninflamed])
excludecrossoverGroup.metagroup = factor(metagroup[metagroup %in% excludecrossoverGroup])
micegroups_inflamed_withbothHMCohorts.metagroup = factor(metagroup[metagroup %in% micegroups_inflamed_withbothHMCohorts])
micegroups_serialTransfersPlusCrossover.metagroup = factor(metagroup[metagroup %in% micegroups_serialTransfersPlusCrossover])

  ## all mice groups
micegroups_all_genusCounts_samplesOnly_lognorm_df = genusCounts_samplesOnly_df_lognorm[rownames(genusCounts_samplesOnly_df_lognorm) %in% micegroups_all.samplesindex,]
micegroups_all.metagroup
micegroups_all_colors = plotColors_ver2[levels(metagroup) %in% micegroups_all]
  ## KO mice groups
micegroups_inflamed_genusCounts_samplesOnly_lognorm_df = genusCounts_samplesOnly_df_lognorm[rownames(genusCounts_samplesOnly_df_lognorm) %in% micegroups_inflamed.samplesindex,]
micegroups_inflamed.metagroup
micegroups_inflamed_colors = plotColors_ver2[levels(metagroup) %in% micegroups_inflamed]
  ## WT mice groups
micegroups_noninflamed_genusCounts_samplesOnly_lognorm_df = genusCounts_samplesOnly_df_lognorm[rownames(genusCounts_samplesOnly_df_lognorm) %in% micegroups_noninflamed.samplesindex,]
micegroups_noninflamed.metagroup
micegroups_noninflamed_colors = plotColors_ver2[levels(metagroup) %in% micegroups_noninflamed]
  ## excluding crossover group
excludecrossoverGroup_genusCounts_samplesOnly_lognorm_df = genusCounts_samplesOnly_df_lognorm[rownames(genusCounts_samplesOnly_df_lognorm) %in% excludcrossoverGroup.samplesindex,]
excludecrossoverGroup.metagroup
excludecrossoverGroup_colors = plotColors_ver2[levels(metagroup) %in% excludecrossoverGroup]
  ## KO mice groups with both HM cohorts
micegroups_inflamed_withbothHMCohorts_genusCounts_samplesOnly_lognorm_df = genusCounts_samplesOnly_df_lognorm[rownames(genusCounts_samplesOnly_df_lognorm) %in% micegroups_inflamed_withbothHMCohorts.samplesindex,]
micegroups_inflamed_withbothHMCohorts.metagroup
micegroups_inflamed_withbothHMCohorts_colors = plotColors_ver2[levels(metagroup) %in% micegroups_inflamed_withbothHMCohorts]

## serial transfer groups plus crossover group
micegroups_serialTransfersPlusCrossover_genusCounts_samplesOnly_lognorm_df = genusCounts_samplesOnly_df_lognorm[rownames(genusCounts_samplesOnly_df_lognorm) %in% micegroups_serialTransfersPlusCrossover.samplesindex,]
micegroups_serialTransfersPlusCrossover.metagroup
micegroups_serialTransfersPlusCrossover_colors = plotColors_ver2[levels(metagroup) %in% micegroups_serialTransfersPlusCrossover]


pdf(here("PcoaPlots","PCoAPlots.pdf"))
generatePCOAPlots(micegroups_serialTransfersPlusCrossover_genusCounts_samplesOnly_lognorm_df,
                  micegroups_serialTransfersPlusCrossover.metagroup,
                  micegroups_serialTransfersPlusCrossover_colors, legendlocation = "topright",
                  plotTitle="Mice Groups, Including Crossover Group")

generatePCOAPlots(micegroups_inflamed_withbothHMCohorts_genusCounts_samplesOnly_lognorm_df,
                  micegroups_inflamed_withbothHMCohorts.metagroup,
                  micegroups_inflamed_withbothHMCohorts_colors, legendlocation = "topleft", plotTitle="Inflamed Mice Groups, Both HM Cohorts")

generatePCOAPlots(genusCounts_samplesOnly_df_lognorm, metagroup,
                  plotColors_ver2, legendlocation = "bottomleft", plotTitle="All Groups")

generatePCOAPlots(micegroups_all_genusCounts_samplesOnly_lognorm_df, micegroups_all.metagroup,
                  micegroups_all_colors, legendlocation = "topright", plotTitle="Mice Groups Only")

generatePCOAPlots(excludecrossoverGroup_genusCounts_samplesOnly_lognorm_df, excludecrossoverGroup.metagroup,
                  excludecrossoverGroup_colors, legendlocation = "topleft", plotTitle="Excluding Cross-Over Group")

generatePCOAPlots(micegroups_noninflamed_genusCounts_samplesOnly_lognorm_df, micegroups_noninflamed.metagroup,
                  micegroups_noninflamed_colors, legendlocation = "topright", plotTitle="Non-inflamed Mice Groups")

generatePCOAPlots(micegroups_inflamed_genusCounts_samplesOnly_lognorm_df, micegroups_inflamed.metagroup, micegroups_inflamed_colors, legendlocation = "topright", plotTitle="Inflamed Mice Groups")

dev.off()
```

Correlations and Scatterplots

The next few segments of code generates the following:

- Correlation coefficients for sample-pairs 1) within group and
    2. across groups
- Boxplots that summarize the correlation coefficients
- Scatterplots for each sample-pair comparison, which reflects relative abundance for each ASV between a given pair of samples

ASV Transfer Efficiencies Within FMT Groups

This first code chunk calculates the correlation of samples within the same group.

```r
file_asv_updatedName_t_samplesOnly_lognorm
labels_inputRecipientGroups

# serial passage groups
labels_NIMM_lineage = c("HM1input", "HM1->WT", "NIMM-g1input", "NIMM-g1->WT", "NIMM-g2input", "NIMM-g2->WT")
labels_IMM_lineage = c("HM1input", "HM1->KO", "IMM-g1input", "IMM-g1->KO","IMM-g2input", "IMM-g2->KO")
labels_serialpassage_newNames = c("HM1input", "HM1->WT", "NIMM-g1input", "NIMM-g1->WT", "NIMM-g2input", "NIMM-g2->WT","HM1->KO", "IMM-g1input", "IMM-g1->KO","IMM-g2input", "IMM-g2->KO")
myASVandMetadata_newNames
names(myASVandMetadata_newNames)[names(myASVandMetadata_newNames)=="FMTGroupFMTsource..Recipientbackground"] = "FMTGroupFMTsourcegtRecipientbackground"

## scatterplots
dir.create(here("boxplots","acrossGroups_pearson"),recursive = T)
dir.create(here("boxplots","withinGroups_pearson"),recursive = T)
dir.create(here("asvAbundance_scatterplots","acrossGroups_pearson"), recursive = T)

  #within groups
withinGroup_pearson_newNames_serialpassage=getCorrelationCoefficient_withinGroup(groupList=labels_serialpassage_newNames, tableWithMeta=myASVandMetadata_newNames, tableCountsOnly=file_asv_updatedName_t_samplesOnly_lognorm, corrTestMethod="pearson",sampleColumnName = "SampleID", variableName = "FMTGroupFMTsourcegtRecipientbackground")

withinGroup_pearson_newNames_NIMMlineage=getCorrelationCoefficient_withinGroup(groupList=labels_NIMM_lineage, tableWithMeta=myASVandMetadata_newNames, tableCountsOnly=file_asv_updatedName_t_samplesOnly_lognorm, corrTestMethod="pearson",sampleColumnName = "SampleID", variableName = "FMTGroupFMTsourcegtRecipientbackground")

withinGroup_pearson_newNames_IMMlineage=getCorrelationCoefficient_withinGroup(groupList=labels_IMM_lineage, tableWithMeta=myASVandMetadata_newNames, tableCountsOnly=file_asv_updatedName_t_samplesOnly_lognorm, corrTestMethod="pearson",sampleColumnName = "SampleID", variableName = "FMTGroupFMTsourcegtRecipientbackground")


## figure 4s
withinGroup_pearson_IMMlineage_genusCounts =getCorrelationCoefficient_withinGroup(groupList=labels_IMM_lineage, tableWithMeta=myASVandMetadata_newNames, tableCountsOnly=file_asv_updatedName_t_samplesOnly_lognorm, corrTestMethod="pearson",sampleColumnName = "SampleID", variableName = "FMTGroupFMTsourcegtRecipientbackground")


## create pdf of boxplots
pdf(here("boxplots","withinGroups_pearson","asv_boxplots_withinGroup_allSerialPassage.pdf"), height = 8, width = 10)
boxplot(withinGroup_pearson_newNames_serialpassage,names = labels_serialpassage_newNames, las=2, cex.axis=0.70,
        main="Correlation Coefficients Within Groups, All Serial Passage Groups", cex.main=0.8)
dev.off()

pdf(here("boxplots","withinGroups_pearson","asv_boxplots_withinGroup_SerialPassage_NIMM.pdf"), height = 8, width = 10)
boxplot(withinGroup_pearson_newNames_NIMMlineage,names = labels_NIMM_lineage, las=2, cex.axis=0.70,
        main="Correlation Coefficients Within Groups, Serial Passage for NIMM Lineage", cex.main=0.8)
dev.off()

pdf(here("boxplots","withinGroups_pearson","asv_boxplots_withinGroup_SerialPassage_IMM.pdf"), height = 8, width = 10)
boxplot(withinGroup_pearson_newNames_IMMlineage,names = labels_IMM_lineage, las=2, cex.axis=0.70,
        main="Correlation Coefficients Within Groups, Serial Passage for IMM Lineage", cex.main=0.8)
dev.off()


#scatterplots, asv correlation for samples across groups
generateScatterplots_acrossGroup(groupList=labels_serialpassage_newNames, tableWithMeta=myASVandMetadata_newNames,corrTestMethod = "pearson"  ,
                                 variableName = "FMTGroupFMTsourcegtRecipientbackground", sampleColumnName ="SampleID" ,tableCountsOnly=file_asv_updatedName_t_samplesOnly_lognorm, resultsDirectory = here("asvAbundance_scatterplots","acrossGroups_pearson","scatterplot_"))
```

ASV Transfer Efficiencies Within FMT Groups: Human Cohorts

Next, we generate correlations (within groups) for samples within only human cohorts (HM1, HM2), which are then visually summarized as boxplots.

```r
labels_bothHumanCohorts = c("HM1->KO", "HM2->KO")

withinGroup_pearson_humanCohortsOnly = getCorrelationCoefficient_withinGroup(groupList=labels_bothHumanCohorts, tableWithMeta=myASVandMetadata_newNames, tableCountsOnly=file_asv_updatedName_t_samplesOnly_lognorm, corrTestMethod="pearson", variableName = "FMTGroupFMTsourcegtRecipientbackground", sampleColumnName = "SampleID")

pdf(here("boxplots","withinGroups_pearson", "ASV_boxplot_withinGroup_bothHumanCohortsOnly.pdf"))
boxplot(withinGroup_pearson_humanCohortsOnly,names = labels_bothHumanCohorts, las=2, cex.axis=0.70,
        main="Pearson Correlation Coefficients Within Groups, Human Cohorts 1 and 2", cex.main=0.8)
stripchart(withinGroup_pearson_humanCohortsOnly, add=TRUE, pch=16, col=c("green", "purple"), method="jitter", vertical = T)

dev.off()

myList =c("HM1->WT", "NIMM-g1->WT", "NIMM-g2->WT","HM1->KO", "IMM-g1->KO", "IMM-g2->KO")

withinGroup_pearson_theSixGroups = getCorrelationCoefficient_withinGroup(groupList=myList, tableWithMeta=myASVandMetadata_newNames, tableCountsOnly=file_asv_updatedName_t_samplesOnly_lognorm, corrTestMethod="pearson", variableName = "FMTGroupFMTsourcegtRecipientbackground", sampleColumnName = "SampleID")

pdf(here("boxplots","withinGroups_pearson", "ASV_boxplot_withinGroup_WTandKOgroups.pdf"))

boxplot(withinGroup_pearson_theSixGroups,names = myList, las=2, cex.axis=0.70,
        main="Pearson Correlation Coefficients Within Groups, WT vs KO", cex.main=0.8)
stripchart(withinGroup_pearson_theSixGroups, add=TRUE, pch=16, col="blue", method="jitter", vertical = T)

dev.off()
```

ASV Transfer Efficiencies Across FMT Groups

The code below generates ASV scatterplots and boxplots for samples across groups. This first code chunk sets the FMT groups of interest for the following analysis.

```r
file_asv_updatedName_t_samplesOnly_lognorm
labels_inputRecipientGroups

labels_NIMM_lineage = c("HM1input", "HM1->WT", "NIMM-g1input", "NIMM-g1->WT", "NIMM-g2input", "NIMM-g2->WT")
labels_IMM_lineage = c("HM1input", "HM1->KO", "IMM-g1input", "IMM-g1->KO","IMM-g2input", "IMM-g2->KO")
labels_serialpassage_newNames = c("HM1input", "HM1->WT", "NIMM-g1input", "NIMM-g1->WT", "NIMM-g2input", "NIMM-g2->WT","HM1->KO", "IMM-g1input", "IMM-g1->KO","IMM-g2input", "IMM-g2->KO")
myASVandMetadata_newNames

#indexing groups of interest for asv correlation boxplots
fig4a.wtlineage.index = c(1,20,35)
fig4a.wtlineage.supp.index = c(2,4,21)

fig4b.kolineage.index = c(6,50,55)
fig4b.kolineage.supp.index = c(7,9,51)

fig4c.index = c(12,29,47,54)
fig4c.supp.index = c(32,43,34,45)
```

Next, we generate the correlations between samples from across groups at three taxonomic levels: strain level (ASV), genus, and phylum.

```r
#across groups
  ## asv level counts
corr_acrossGroups_pearson_asvCounts_withHuman = getCorrelationCoefficient_acrossGroup(groupList=labels_serialpassage_newNames, tableWithMeta=myASVandMetadata_newNames, tableCountsOnly=file_asv_updatedName_t_samplesOnly_lognorm, corrTestMethod="pearson",variableName = "FMTGroupFMTsourcegtRecipientbackground", sampleColumnName = "SampleID")

  ## genus level counts
corr_acrossGroups_pearson_GenusCounts_withHuman = getCorrelationCoefficient_acrossGroup(groupList=labels_serialpassage_newNames, tableWithMeta=genusCountsandMetadata, tableCountsOnly=genusCounts_samplesOnly_df_lognorm,
corrTestMethod="pearson", variableName = "FMTGroupFMTsourcegtRecipientbackground", sampleColumnName = "SampleID")

    ## phylum level counts
corr_acrossGroups_pearson_PhylumCounts_withHuman = getCorrelationCoefficient_acrossGroup(groupList=labels_serialpassage_newNames, tableWithMeta=phylumCountsandMetadata, tableCountsOnly=phylumCounts_samplesOnly_df_lognorm,
corrTestMethod="pearson", variableName = "FMTGroupFMTsourcegtRecipientbackground", sampleColumnName = "SampleID")
```

These boxplots are for inflamed (KO) groups in our SV transfer comparison across groups.

```r
  #asv level counts
pdf(here("boxplots","acrossGroups_pearson", "fig4_inflamed_asv.pdf"))
boxplot(corr_acrossGroups_pearson_asvCounts_withHuman[[2]][fig4b.kolineage.index],names = corr_acrossGroups_pearson_asvCounts_withHuman[[1]][fig4b.kolineage.index],  cex.axis=0.6,
        main="Correlation Coefficients Across Groups, Inflamed Groups", cex.main=0.8,
        ylim=c(0,1))
dev.off()

pdf(here("boxplots","acrossGroups_pearson","fig4_inflamed_asv_supp.pdf"))
boxplot(corr_acrossGroups_pearson_asvCounts_withHuman[[2]][fig4b.kolineage.supp.index],names = corr_acrossGroups_pearson_asvCounts_withHuman[[1]][fig4b.kolineage.supp.index],  cex.axis=0.6,
        main="Correlation Coefficients Across Groups, Inflamed Groups (SUPPFIG)", cex.main=0.8,
        ylim=c(0,1))
dev.off()

  # genus level counts

pdf(here("boxplots","acrossGroups_pearson","fig4_inflamed_genus.pdf"))
boxplot(corr_acrossGroups_pearson_GenusCounts_withHuman[[2]][fig4b.kolineage.index],names = corr_acrossGroups_pearson_GenusCounts_withHuman[[1]][fig4b.kolineage.index],  cex.axis=0.6,
        main="Correlation Coefficients Across Groups, Inflamed Groups (Genus)", cex.main=0.8,
        ylim=c(0,1))
dev.off()

pdf(here("boxplots","acrossGroups_pearson","fig4_inflamed_genus_supp.pdf"))
boxplot(corr_acrossGroups_pearson_GenusCounts_withHuman[[2]][fig4b.kolineage.supp.index],names = corr_acrossGroups_pearson_GenusCounts_withHuman[[1]][fig4b.kolineage.supp.index],  cex.axis=0.6,
        main="Correlation Coefficients Across Groups, Inflamed Groups (Genus) (SUPPFIG)", cex.main=0.8,
        ylim=c(0,1))
dev.off()

    #phylum counts

pdf(here("boxplots","acrossGroups_pearson", "fig4_inflamed_phylum.pdf"))
boxplot(corr_acrossGroups_pearson_PhylumCounts_withHuman[[2]][fig4b.kolineage.index],names = corr_acrossGroups_pearson_PhylumCounts_withHuman[[1]][fig4b.kolineage.index],  cex.axis=0.6,
        main="Correlation Coefficients Across Groups, Inflamed Groups (Phylum)", cex.main=0.8,
        ylim=c(0,1))
dev.off()

pdf(here("boxplots","acrossGroups_pearson","fig4_inflamed_phylum_supp.pdf"))
boxplot(corr_acrossGroups_pearson_PhylumCounts_withHuman[[2]][fig4b.kolineage.supp.index],names = corr_acrossGroups_pearson_PhylumCounts_withHuman[[1]][fig4b.kolineage.supp.index],  cex.axis=0.6,
        main="Correlation Coefficients Across Groups, Inflamed Groups (Phylum, SUPPFIG)", cex.main=0.8,
        ylim=c(0,1))
dev.off()
```

These boxplots are for non-inflamed (WT) groups in our SV transfer comparison across groups.

```r
#asv counts

pdf(here("boxplots","acrossGroups_pearson","fig4_NONinflamed_asv.pdf"))
boxplot(corr_acrossGroups_pearson_asvCounts_withHuman[[2]][fig4a.wtlineage.index],names = corr_acrossGroups_pearson_asvCounts_withHuman[[1]][fig4a.wtlineage.index],  cex.axis=0.6,
        main="Correlation Coefficients Across Groups, Non-inflamed Groups", cex.main=0.8,
        ylim=c(0,1))
dev.off()

pdf(here("boxplots","acrossGroups_pearson","fig4_NONinflamed_asv_supp.pdf"))
boxplot(corr_acrossGroups_pearson_asvCounts_withHuman[[2]][fig4a.wtlineage.supp.index],names = corr_acrossGroups_pearson_asvCounts_withHuman[[1]][fig4a.wtlineage.supp.index],  cex.axis=0.6,
        main="Correlation Coefficients Across Groups, Non-inflamed Groups (SUPPFIG)", cex.main=0.8,
        ylim=c(0,1))
dev.off()

    #genus counts

pdf(here("boxplots","acrossGroups_pearson", "fig4_NONinflamed_genus.pdf"))
boxplot(corr_acrossGroups_pearson_GenusCounts_withHuman[[2]][fig4a.wtlineage.index],names = corr_acrossGroups_pearson_GenusCounts_withHuman[[1]][fig4a.wtlineage.index],  cex.axis=0.6,
        main="Correlation Coefficients Across Groups, Non-inflamed Groups (Genus)", cex.main=0.8,
        ylim=c(0,1))
dev.off()

pdf(here("boxplots","acrossGroups_pearson","fig4_NONinflamed_genus_supp.pdf"))
boxplot(corr_acrossGroups_pearson_GenusCounts_withHuman[[2]][fig4a.wtlineage.supp.index],names = corr_acrossGroups_pearson_GenusCounts_withHuman[[1]][fig4a.wtlineage.supp.index],  cex.axis=0.6,
        main="Correlation Coefficients Across Groups, Non-inflamed Groups (Genus,SUPPFIG)", cex.main=0.8,
        ylim=c(0,1))
dev.off()

  # phylum counts

pdf(here("boxplots","acrossGroups_pearson","fig4_NONinflamed_phylum.pdf"))
boxplot(corr_acrossGroups_pearson_PhylumCounts_withHuman[[2]][fig4a.wtlineage.index],names = corr_acrossGroups_pearson_PhylumCounts_withHuman[[1]][fig4a.wtlineage.index],  cex.axis=0.6,
        main="Correlation Coefficients Across Groups, Non-inflamed Groups (Phylum)", cex.main=0.8,
        ylim=c(0,1))
dev.off()

pdf(here("boxplots","acrossGroups_pearson","fig4_NONinflamed_phylum_supp.pdf"))
boxplot(corr_acrossGroups_pearson_PhylumCounts_withHuman[[2]][fig4a.wtlineage.supp.index],names = corr_acrossGroups_pearson_PhylumCounts_withHuman[[1]][fig4a.wtlineage.supp.index],  cex.axis=0.6,
        main="Correlation Coefficients Across Groups, Non-inflamed Groups (Phylum,SUPPFIG)", cex.main=0.8,
        ylim=c(0,1))
dev.off()
```

These boxplots include the cross-over groups in our SV transfer comparison across groups.

```r
#asv counts
pdf(here("boxplots","acrossGroups_pearson","fig4_crossover_asv.pdf"), width = 10)
boxplot(corr_acrossGroups_pearson_asvCounts_withHuman[[2]][fig4c.index],names = corr_acrossGroups_pearson_asvCounts_withHuman[[1]][fig4c.index],  cex.axis=0.6,
        main="Correlation Coefficients Across Groups, Cross-over Group", cex.main=0.8,
        ylim=c(0,1))
dev.off()

pdf(here("boxplots","acrossGroups_pearson","fig4_crossover_supp_asv.pdf"))
boxplot(corr_acrossGroups_pearson_asvCounts_withHuman[[2]][fig4c.supp.index],names = corr_acrossGroups_pearson_asvCounts_withHuman[[1]][fig4c.supp.index],  cex.axis=0.6,
        main="Correlation Coefficients Across Groups, Cross-over Group (SUPPFIG)", cex.main=0.8,
        ylim=c(0,1))
dev.off()

    #genus counts
pdf(here("boxplots","acrossGroups_pearson","fig4_crossover_genus.pdf"))
boxplot(corr_acrossGroups_pearson_GenusCounts_withHuman[[2]][fig4c.index],
        names = corr_acrossGroups_pearson_GenusCounts_withHuman[[1]][fig4c.index],  cex.axis=0.6,
        main="Correlation Coefficients Across Groups, Cross-over Group (Genus)", cex.main=0.8,
        ylim=c(0,1))
dev.off()

pdf(here("boxplots","acrossGroups_pearson","fig4_crossover_supp_genus.pdf"))
boxplot(corr_acrossGroups_pearson_GenusCounts_withHuman[[2]][fig4c.supp.index],names = corr_acrossGroups_pearson_GenusCounts_withHuman[[1]][fig4c.supp.index],  cex.axis=0.6,
        main="Correlation Coefficients Across Groups, Cross-over Group (Genus, SUPPFIG)", cex.main=0.8,
        ylim=c(0,1))
dev.off()

    #phylum counts
pdf(here("boxplots","acrossGroups_pearson","fig4_crossover_phylum.pdf"))
boxplot(corr_acrossGroups_pearson_PhylumCounts_withHuman[[2]][fig4c.index],
        names = corr_acrossGroups_pearson_PhylumCounts_withHuman[[1]][fig4c.index],  cex.axis=0.6,
        main="Correlation Coefficients Across Groups, Cross-over Group (Phylum)", cex.main=0.8,
        ylim=c(0,1))
dev.off()

pdf(here("boxplots","acrossGroups_pearson","fig4_crossover_supp_phylum.pdf"))
boxplot(corr_acrossGroups_pearson_PhylumCounts_withHuman[[2]][fig4c.supp.index],names = corr_acrossGroups_pearson_PhylumCounts_withHuman[[1]][fig4c.supp.index],  cex.axis=0.6,
        main="Correlation Coefficients Across Groups, Cross-over Group (Phylum, SUPPFIG)", cex.main=0.8,
        ylim=c(0,1))
dev.off()
```

ASV Transfer Efficiencies Across FMT Groups: Non-transferred ASVs

Here, we plot the abundance of the non-transferred ASVs as histograms.

```r
dir.create(here("histograms_nontransferredASV","tablesWithTaxonomy"),recursive = T)

labels_NIMM_lineage = c("HM1input", "HM1->WT", "NIMM-g1input", "NIMM-g1->WT", "NIMM-g2input", "NIMM-g2->WT")
labels_IMM_lineage = c("HM1input", "HM1->KO", "IMM-g1input", "IMM-g1->KO","IMM-g2input", "IMM-g2->KO")


generateScatterPlots_samplePairsAcrossGroup_histogramOfNontransferringASVS(groupList=labels_serialpassage_newNames,
                                                                           tableWithMeta=myASVandMetadata_newNames,
                                                                             tableCountsOnly=file_asv_updatedName_t_samplesOnly_lognorm, resultsDirectory = here("histograms_nontransferredASV", "histogram_"))
```

```r
#tables of non-transferred asvs with taxa name in each

generateTableWithTaxonomy_NontransferringASVS_samplePairsAcrossGroup(groupList=labels_serialpassage_newNames,
                                                                     taxonomyMap=asv_taxonomy_map,
                                                                     taxaColName="taxonomy",
                                                                     asvColName="OTUID",
                                                                     tableWithMeta=myASVandMetadata_newNames,
                                                                     tableCountsOnly=file_asv_updatedName_t_samplesOnly_lognorm,
                                                                     resultsDirectory=here("histograms_nontransferredASV","tablesWithTaxonomy", "table_"))
```

ASV Transfer Efficiencies Within FMT Groups at the Phylum level

The code below parses the taxonomy column to count the number of different phylum present in this counts table. The phylum "NA" means unclassified.

There are a total of 13 phyla in the ASV counts table. Of these 13 phyla, only four had one ASV present. For statistical testing purposes, these phyla were excluded from the Pearson Correlation Coefficient test: Arthropoda, Cyanobacteria, Desulfobacterota, and Vertebrata. Campilobacterota and Patescibacteria were very sparse (present in 5 or less samples each), and since this led to empty results in the boxplot, we excluded these two phyla as well.

```r
taxanames_all = file_asv_updatedName$taxonomy

taxanames_all_split = strsplit(taxanames_all, ";")

phylum_label = NULL

for(i in 1:length(taxanames_all_split)){
  phylum_label[i] = taxanames_all_split[[i]][2]
}

phylum_label_short = gsub("p__", "", phylum_label)
phylum_label_short = gsub(" ", "", phylum_label_short)

mapping_ASVandPhyla = data.frame("ASV" = file_asv_updatedName$OTUID, "PhylumLabels" = phylum_label_short)
table(mapping_ASVandPhyla$PhylumLabels)

mapping_ASVandPhyla$PhylumLabels[is.na(mapping_ASVandPhyla$PhylumLabels)] = "Unclassified"

#exclude the single ASV phylum
phlyum_groups = unique(mapping_ASVandPhyla$PhylumLabels)
singles = c("Arthropoda","Cyanobacteria","Desulfobacterota", "Vertebrata")
phlyum_groups_subset = phlyum_groups[! phlyum_groups %in% singles]

#rename the NA group in phylum list
phlyum_groups_subset_2 = phlyum_groups_subset
phlyum_groups_subset_2[7] = "Unclassified"

phlyum_groups_subset_withoutSparsePhyla = phlyum_groups_subset_2[phlyum_groups_subset_2 != "Patescibacteria" & phlyum_groups_subset_2 != "Campilobacterota"]

phlyum_groups_subset_withoutSparsePhyla[7] = "Unclassified"

myGroup_serialPassage_miceOnly = c("IMM-g1->KO", "IMM-g2->KO", "NIMM-g1->WT", "NIMM-g2->WT")
myGroup_serialPassage_miceandHuman = c("IMM-g1->KO", "IMM-g2->KO", "NIMM-g1->WT", "NIMM-g2->WT", "HM1->KO", "HM1->WT")
sampleID_for_myGroup_serialPassage_miceOnly = metamapping_updatedName$SampleID[metamapping_updatedName$FMTGroupFMTsource..Recipientbackground %in% myGroup_serialPassage_miceOnly[1]]

mygroup_outfile_labels = gsub("->", "_to_", myGroup_serialPassage_miceandHuman)

## for selected FMT groups, generate correlations (samples within the same FMT group, stratified at the phylum taxa level) and boxplots of those correlations
for(i in 1:length(myGroup_serialPassage_miceandHuman)){

  myCorr_withinGroup_pearson_phlyum_perIRGroup_OUTPUT=getCorrelationCoefficient_withinGroup_phylumLevel_byGroup(groupList=phlyum_groups_subset_withoutSparsePhyla, tableWithMeta=myASVandMetadata_newNames, tableCountsOnly=file_asv_updatedName_t_samplesOnly_lognorm, corrTestMethod="pearson", sampleGroup = myGroup_serialPassage_miceandHuman[i], mappingMetaFile = mapping_ASVandPhyla,mappingMetaFile_taxaColName = "ASV", mappingMetaFile_phylumColName = "PhylumLabels", tableWithMeta_sampleColName = "SampleID" ,tableWithMeta_groupColName = "FMTGroupFMTsourcegtRecipientbackground")

  pdf(paste0(here("boxplots","withinGroups_pearson", "boxplot_phylum_"),mygroup_outfile_labels[i] , ".pdf"), width = 10)
  boxplot(myCorr_withinGroup_pearson_phlyum_perIRGroup_OUTPUT[[2]], names = phlyum_groups_subset_withoutSparsePhyla, main=paste0("Pearson Correlation Coefficient (R), Phylum Level ", myGroup_serialPassage_miceandHuman[i]), cex.axis=0.65, yaxt="n")
  axis(side=2, at=seq(0, 1, by=0.1))
  dev.off()
}
```

Next, we calculate t-test p-values to compare the correlations for each phylum between WT and KO groups to determine if there are significant differences between phylum for each phenotype.

At this time, we performed the t-test on the HM1toWT vs HM1toKO group. Results are generated as a dataframe with unadjusted p-values for each phylum. This dataframe is written out as a tab-delimited text file.

```r
## ttest to compare wt and ko phylum groups

myCorr_withinGroup_pearson_phlyum_immg1toKO=getCorrelationCoefficient_withinGroup_phylumLevel_byGroup(groupList=phlyum_groups_subset_withoutSparsePhyla, tableWithMeta=myASVandMetadata_newNames, tableCountsOnly=file_asv_updatedName_t_samplesOnly_lognorm, corrTestMethod="pearson", sampleGroup = myGroup_serialPassage_miceandHuman[1], mappingMetaFile = mapping_ASVandPhyla,mappingMetaFile_taxaColName = "ASV", mappingMetaFile_phylumColName = "PhylumLabels", tableWithMeta_sampleColName = "SampleID" ,tableWithMeta_groupColName = "FMTGroupFMTsourcegtRecipientbackground")

myCorr_withinGroup_pearson_phlyum_nimmg1toWT=getCorrelationCoefficient_withinGroup_phylumLevel_byGroup(groupList=phlyum_groups_subset_withoutSparsePhyla, tableWithMeta=myASVandMetadata_newNames, tableCountsOnly=file_asv_updatedName_t_samplesOnly_lognorm, corrTestMethod="pearson", sampleGroup = myGroup_serialPassage_miceandHuman[3], mappingMetaFile = mapping_ASVandPhyla, mappingMetaFile_taxaColName = "ASV", mappingMetaFile_phylumColName = "PhylumLabels", tableWithMeta_sampleColName = "SampleID" ,tableWithMeta_groupColName = "FMTGroupFMTsourcegtRecipientbackground")

myCorr_withinGroup_pearson_phlyum_hm1toWT=getCorrelationCoefficient_withinGroup_phylumLevel_byGroup(groupList=phlyum_groups_subset_withoutSparsePhyla, tableWithMeta=myASVandMetadata_newNames, tableCountsOnly=file_asv_updatedName_t_samplesOnly_lognorm, corrTestMethod="pearson", sampleGroup = myGroup_serialPassage_miceandHuman[6], mappingMetaFile = mapping_ASVandPhyla,mappingMetaFile_taxaColName = "ASV", mappingMetaFile_phylumColName = "PhylumLabels", tableWithMeta_sampleColName = "SampleID" ,tableWithMeta_groupColName = "FMTGroupFMTsourcegtRecipientbackground")

myCorr_withinGroup_pearson_phlyum_hm1toKO=getCorrelationCoefficient_withinGroup_phylumLevel_byGroup(groupList=phlyum_groups_subset_withoutSparsePhyla, tableWithMeta=myASVandMetadata_newNames, tableCountsOnly=file_asv_updatedName_t_samplesOnly_lognorm, corrTestMethod="pearson", sampleGroup = myGroup_serialPassage_miceandHuman[5], mappingMetaFile = mapping_ASVandPhyla,mappingMetaFile_taxaColName = "ASV", mappingMetaFile_phylumColName = "PhylumLabels", tableWithMeta_sampleColName = "SampleID" ,tableWithMeta_groupColName = "FMTGroupFMTsourcegtRecipientbackground")


ttestpval_list = NULL
for(i in 1:length(phlyum_groups_subset_withoutSparsePhyla)){

  a = unlist(myCorr_withinGroup_pearson_phlyum_hm1toWT[[2]][i])
  b = unlist(myCorr_withinGroup_pearson_phlyum_hm1toKO[[2]][i])
  if(sum(is.na(a)) >=1 | sum(is.na(b)) >=1){print(paste0(phlyum_groups_subset_withoutSparsePhyla[i], " : NA"))} else {

  ttest_pval = t.test(a,b)$p.value
  resultstoprint = paste0(phlyum_groups_subset_withoutSparsePhyla[i],
                          ": ", ttest_pval)
  ttestpval_list[i] = ttest_pval

  }
}

mydf = data.frame("Phylum"=phlyum_groups_subset_withoutSparsePhyla,
                  "Pvalue" = ttestpval_list)

write.table(mydf, here("boxplots","withinGroups_pearson","phylum_boxplot_ttest_hm1toWT_vs_hm1toKO.txt"), sep = "\t", quote = F, row.names = F)
```

The code below generates scatterplots of samples across all groups with phylum-level counts data. This differs from the above figures, which were based on ASV counts, but was sorted according to phyla taxonomic classification.

```r
dir.create(here("asvAbundance_scatterplots","acrossGroups_pearson_phylumCounts"), recursive = T)

corr_acrossGroups_pearson_PhylumCounts_withHuman = getCorrelationCoefficient_acrossGroup(groupList=labels_serialpassage_newNames, tableWithMeta=phylumCountsandMetadata, tableCountsOnly=phylumCounts_samplesOnly_df_lognorm,
corrTestMethod="pearson", variableName = "FMTGroupFMTsourcegtRecipientbackground", sampleColumnName = "SampleID")

generateScatterplots_acrossGroup(groupList=labels_serialpassage_newNames, tableWithMeta=phylumCountsandMetadata,corrTestMethod = "pearson"  ,
                                 variableName = "FMTGroupFMTsourcegtRecipientbackground", sampleColumnName ="SampleID" ,tableCountsOnly=phylumCounts_samplesOnly_df_lognorm, resultsDirectory = here("asvAbundance_scatterplots","acrossGroups_pearson_phylumCounts","scatterplot_"))
```

Testing homogeneity of variances

Bartlett's test checks for equality of variances between two or more groups.

Levene's test is the non-parametric version of variance equality testing.

After testing the data for normality (Shapiro-Wilkes test), the data presented with a normal distribution.

However, there were some outlier scores within some groups, so we also performed the non-parametric Levene's test for a more robust assessment of the histology scores.

```r
histologyScores_hm1immg1immg2toKO = read.table(here("files", "histologyScores_hm1immg1immg2toKO.txt"), sep = "\t", header = T)

phenotype_groups = unique(histologyScores_hm1immg1immg2toKO$Group)
colnames(histologyScores_hm1immg1immg2toKO)
regions_list = c("Ileum", "Cecum","Proximal.Colon","Distal.Colon","Rectum")

bartlettResults_list = list()
for(i in 1:length(regions_list)){
  bartlettPvalues= calculateBartlettPvalue_histologyScoreData(histologyScores_hm1immg1immg2toKO,
                                                              regionColName = regions_list[i],      groupColName = "Group", phenotypeNames=phenotype_groups)

  bartlettResults_list[[i]]= bartlettPvalues
}

names(bartlettResults_list)=regions_list


barlettResult_totalColonScore = calculateBartlettPvalue_histologyScoreData(histologyScores_hm1immg1immg2toKO,
                                                 regionColName = "Total.1",
                                                 groupColName = "Group", phenotypeNames=phenotype_groups)

levenesTest_totalColonScore=calculateLevenesTestPvalue_histologyScoreData(histologyScores_hm1immg1immg2toKO,regionColName = "Total.1", groupColName = "Group",phenotypeNames=phenotype_groups)


colnames(histologyScores_hm1immg1immg2toKO)


leveneResults_list = list()
for(i in 1:length(regions_list)){
  levenePvalues= calculateLevenesTestPvalue_histologyScoreData(histologyScores_hm1immg1immg2toKO,
                                                               regionColName = regions_list[i], groupColName = "Group",phenotypeNames=phenotype_groups)
```

```
  leveneResults_list[[i]]= levenePvalues
}

names(leveneResults_list)=regions_list
```

Cage Effect

We assess transfer inefficiencies for samples within the same groups per cage. Since the transfer efficiency trends were consistent with the same figure that was generated without cage data, we suggest that our initially observed transfer efficiency values were not driven by cage effect.

```
metadataNames= c("SampleID","UniversalCageNumber","Background", "FMTGroupFMTsourcegtRecipientbackground",
               "Passge", "Sex", "Agewk", "SampleType")
fmtList_withinGroup = c("HM1->WT","NIMM-g1->WT", "NIMM-g2->WT",
                        "HM1->KO", "IMM-g1->KO", "IMM-g2->KO")
withinGroups_perCage = getCorrelationCoefficient_withinGroup_perCage(tableWithMeta=myASVandMetadata_newNames,
                                                                     corrTestMethod="pearson",
                                                                     phenotypeGroup=fmtList_withinGroup,
                                                                     tableWithMeta_sampleColName="SampleID",
                                                                     tableWithMeta_groupColName="FMTGroupFMTsourcegtR
ecipientbackground",
                                                                     tableWithMeta_cageColName="UniversalCageNumber",
                                                                    metadataNames = metadataNames)


pdf(here("boxplots","Boxplots_WithinGroups_PerCage.pdf"))

par(mar=c(6, 4, 2, 2))
boxplot(withinGroups_perCage[[2]], names = withinGroups_perCage[[1]], las=2)
stripchart(withinGroups_perCage[[2]], method="jitter",add=T, pch=16, vertical = T)

dev.off()
```

Protein Assay: fLCN2

We compared log10 transformed and non-transformed protein assay data with both t-test and Wilcoxon test.

```
# protein assay data: fLCN2

#histology scores, not in log space

fLCN2_immg1toko = c(228.75,178.26,489.264,396.486,260.886,4679.712,381.708,
                    507.51,3328.215,1100.715,300.915,43.543,11174.805,199.605,477.075
                    )
fLCN2_nimmg1toko = c(76.281,92.0298,6.3465,13.908,195.102,370.494,142.946,292.641,
                     177.647,9.036,221.677,237.329,47.194,910.155,77.11
                       )


#log10 + 1
fLCN2_immg1toko_log10 = log10(fLCN2_immg1toko + 1)
fLCN2_nimmg1toko_log10 = log10(fLCN2_nimmg1toko + 1)

#histogram
par(mfrow=c(2,2))
hist(fLCN2_immg1toko, main="",xlim=c(0,10000))
hist(fLCN2_nimmg1toko, main="",xlim=c(0,10000))
hist(fLCN2_immg1toko_log10, main="",xlim=c(0,5))
hist(fLCN2_nimmg1toko_log10, main="",xlim=c(0,5))
```

```
# stat tests
ttest_pvals = t.test(fLCN2_immg1toko, fLCN2_nimmg1toko)$p.value
wilcoxon_pvals = wilcox.test(fLCN2_immg1toko, fLCN2_nimmg1toko)$p.value

ttest_pvals_log10input = t.test(fLCN2_immg1toko_log10, fLCN2_nimmg1toko_log10)$p.value
wilcoxon_pvals_log10input = wilcox.test(fLCN2_immg1toko_log10, fLCN2_nimmg1toko_log10)$p.value

#boxplots
pdf(here("boxplots","fLCN2_histologyScores.pdf"))

par(mfrow=c(1,2))
boxplot(list(fLCN2_immg1toko, fLCN2_nimmg1toko), names=c("IMMg1toKO", "NIMMg1toKO"),
        main=paste0("fLCN2 (Non-transformed Data)", "\nT-test Pval=", format(ttest_pvals, digits = 4),
                    ", Wilcoxon test Pval=", format(wilcoxon_pvals, digits = 4)),
        cex.main=0.85, col=c("red","royalblue"))
stripchart(list(fLCN2_immg1toko, fLCN2_nimmg1toko),
           add=TRUE, vertical = T, pch=16)

boxplot(list(fLCN2_immg1toko_log10, fLCN2_nimmg1toko_log10), names=c("IMMg1toKO", "NIMMg1toKO"),
        main=paste0("fLCN2 (Log10 transformed Data)", "\nT-test Pval=", format(ttest_pvals_log10input, digits =
4),
                    ", Wilcoxon test Pval=", format(wilcoxon_pvals_log10input, digits = 4)),
        cex.main=0.85, col=c("red","royalblue"))
stripchart(list(fLCN2_immg1toko_log10, fLCN2_nimmg1toko_log10),
           add=TRUE, vertical = T, pch=16)

dev.off()
```

Human to Mice FMT: Gnotobiotic Isolator

In this control experiment, nine mice (129.IL10KO recipient mice) received human stool as input (HM2) in a fecal microbiome transfer within a gnotobiotic transfer. We generated scatterplots to visualize the transfer efficiency of samples from human to mice to see if we still observe non-transferred ASVS that are bottlenecked in the human to mice transfer; that is, we are ruling out environmental contamination as an explanation of this phenomena.

As we observed in these scatterplots, there is still a microbial bottleneck in the human to mice transfer.

```
dir.create(here("asvAbundance_scatterplots","acrossGroups_pearson_gotobioticIsolator_data"), recursive = T)

asv_isolatedFMT = read.table(here("files","countsTable_asv_importToR_isolatorFMT.txt"), header = T, sep = "\t")
isolatedFMT_metadata_all = read.table(here("files","metadata_importToR_isolatorFMT.txt"), header = T, sep = "\t")

matchSampleIDFormat = gsub("-",".",isolatedFMT_metadata_all$SampleID)
isolatedFMT_metadata_all$SampleID = matchSampleIDFormat

asv_isolatedFMT_fmtGroups = unique(isolatedFMT_metadata_all$FMTGroup)

asv_isolatedFMT_t = t(asv_isolatedFMT)
asv_isolatedFMT_t_samplesOnly = asv_isolatedFMT_t[-(c(1,nrow(asv_isolatedFMT_t))),]
colnames(asv_isolatedFMT_t_samplesOnly) = asv_isolatedFMT_t[1,]


asv_isolatedFMT_t_samplesOnly_toMerge = as.data.frame(asv_isolatedFMT_t_samplesOnly)
asv_isolatedFMT_t_samplesOnly_toMerge$SampleID = rownames(asv_isolatedFMT_t_samplesOnly)

#merge counts and metadata files
isolatedFMT_ASVandMetadata_newNames = full_join(asv_isolatedFMT_t_samplesOnly_toMerge, isolatedFMT_metadata_all,
by="SampleID")

##lognorm
asv_isolatedFMT_t_samplesOnly_df = as.data.frame(asv_isolatedFMT_t_samplesOnly)
asv_isolatedFMT_t_samplesOnly_df[] <- lapply(asv_isolatedFMT_t_samplesOnly_df, function(x) as.numeric(as.characte
r(x)))

asv_isolatedFMT_t_samplesOnly_lognorm = lognorm_function(asv_isolatedFMT_t_samplesOnly_df)

# fmt groups
my_asv_isolatedFMT_fmtGroups = c("h3-4-5_input", "h3-4-5->KO")

# across group correlations
corr_acrossGroups_pearson_isolated_asvs = getCorrelationCoefficient_acrossGroup(groupList=my_asv_isolatedFMT_fmtG
roups, tableWithMeta=isolatedFMT_ASVandMetadata_newNames,
                                                                                tableCountsOnly=asv_isolatedFMT_t
_samplesOnly_lognorm,
                                                                                corrTestMethod="pearson",
                                                                                variableName = "FMTGroup", sample
ColumnName = "SampleID")

#scatterplots
generateScatterplots_acrossGroup(groupList=my_asv_isolatedFMT_fmtGroups, tableWithMeta=isolatedFMT_ASVandMetadata
_newNames,
                                 corrTestMethod = "pearson"  ,
                                 variableName = "FMTGroup",
                                 sampleColumnName ="SampleID" ,tableCountsOnly=asv_isolatedFMT_t_samplesOnly_logn
orm,
                                 resultsDirectory = here("asvAbundance_scatterplots","acrossGroups_pearson_gotobi
oticIsolator_data", "scatterplot_"))
```

Figure 6: PCoA ordination and taxonomic relative abundance

In Figure 6 and 6S, to assess the variation and stability of the initial transfer with the HM1 cohort, we generated PCoA plots and taxonomic bar plots for replicate experiments within the following FMT treatment groups: HM1, HM2, and IMM-g1->KO using the Plotmicrobiome application. These figures were tested and validated by Jacqueline B. Young, with the respective Python scripts available on the Github repository under the “Testing” directory.

Contents

- **Aim**
- **Main Python Methods**
- **Data Processing Steps**
  - Step 1: Retrieve original counts table
  - Step 2: Retrieve original metadata table
  - Step 3: Create dictionary from metadata table
  - Step 4: Calculate total read counts per sample
  - Step 5: Extract counts for all six sample groups
  - Step 6: Calculate Spearman Correlation Coefficients for each sample group
  - Step 7: Generate final figure
  - Step 8: Compare original and regenerated figures

Aim: Replicate Figure 1D

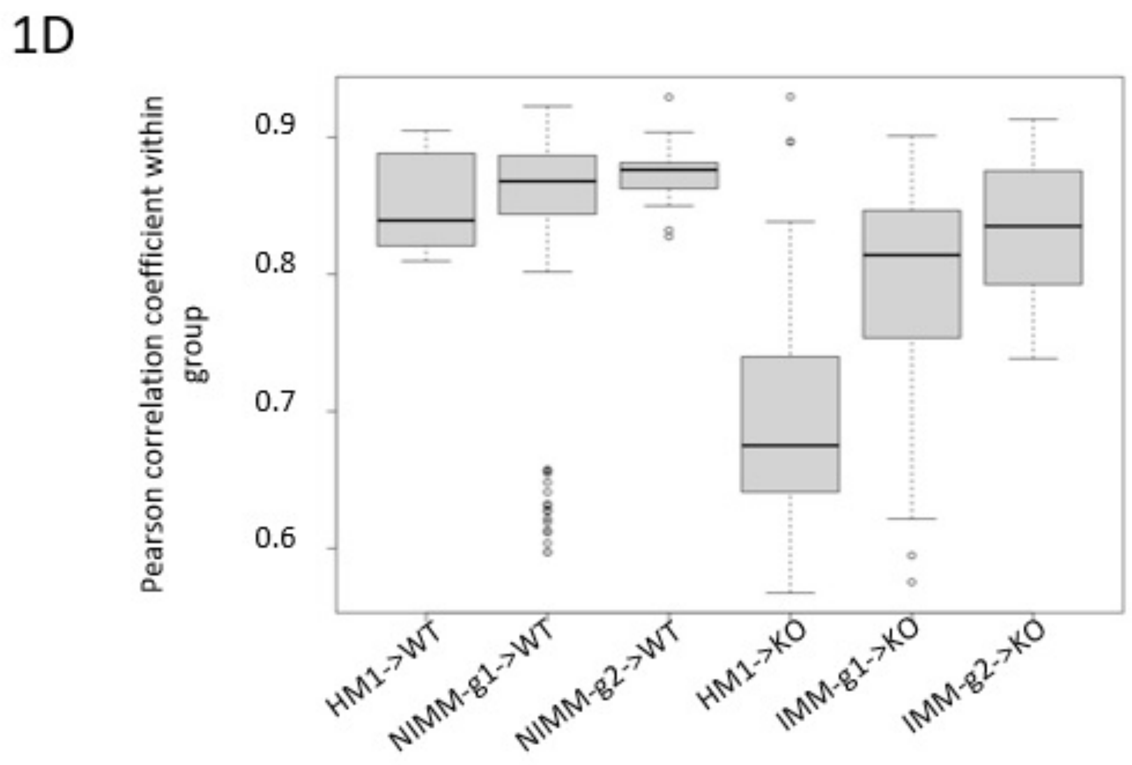

Main Python Methods

In [1]:
```
import numpy as np
import pandas as pd
import matplotlib.pyplot as plt
import os
import re
from scipy import stats
from scipy.special import comb

# ******************************* MAIN METHODS ******************************
```

```
# Retrieve tab delimited file
def read_csv_file(file_path, skiprows=None, header = 0, sep = '\t', index_col=
False):
    df = pd.read_csv(file_path, skiprows=skiprows, sep=sep, header=header, ind
ex_col=index_col)

    return df

# Create dictionary from metadata table
def get_dict_from_metadata(input_df):
    mydict = {}
    for row in input_df.iterrows():
        obj = row[1]
        sample_id = obj['SampleID']
        key = obj['FMTGroupFMTsourcegtRecipientbackground']
        if key not in mydict:
            mydict[key] = [sample_id]
        else:
            mydict[key].append(sample_id)
    return mydict

# Calculate Pearson correlation coefficients from input set of samples
def get_pearson_corr_values(input_df, ser_sample_count_sums):
    overall_mean_count = ser_sample_count_sums.mean()
    col_names = input_df.columns
    df_input_norm_logged = _get_norm_counts(input_df, col_names, ser_sample_co
unt_sums, overall_mean_count)
    final_corr_result = _get_corr_values(col_names, df_input_norm_logged)
    return final_corr_result

# ****************************** HELPER METHODS *****************************
**
# Normalize counts table
def _get_norm_counts(input_df, col_names, ser_sample_count_sums, overall_mean_
count):
    df_norm_logged =    pd.DataFrame()
    for col_name in col_names:
        df_norm_logged.loc[:, col_name] = \
        np.log10(((input_df.loc[:, col_name] / ser_sample_count_sums[col_name
]) * overall_mean_count) + 1)

    return df_norm_logged

# Calculate Pearson Correlation Coefficients
def _get_corr_values(col_names, input_norm_logged_df):
    corr_result = []
    tup_list = _get_tup_list(col_names)

    for tup in tup_list:
        x_col_name = col_names[tup[0]]
        y_col_name = col_names[tup[1]]
        rho_value = \
        stats.pearsonr(input_norm_logged_df.loc[:, x_col_name], input_norm_log
ged_df.loc[:, y_col_name]).statistic
        corr_result.append(rho_value)

    # Remove nan values
    corr_result = [val for val in corr_result if not np.isnan(val)]

    return corr_result

# Generate tuple list of sample column names
```

```
def _get_tup_list(col_names):
    tup_list = []
    corr_result = []
    col_name_length = len(col_names)
    for i in range(col_name_length):
        for j in range(i+1, col_name_length):
            tup_list.append((i, j))

    return tup_list

# ******************************* INPUT FILE PATHS ***************************
****
# Get current working directory
current_working_dir = os.getcwd()
# original counts table
asv_tbl_file_path = os.path.join(current_working_dir, 'asv_biom-with-taxonomy.
txt')
# original metadata table
metadata_file_path = os.path.join(current_working_dir, 'mappingMetadata.txt')
```

Data Processing Steps

Step 1: Retrieve original counts table

In [2]:

```
df_asv = read_csv_file(asv_tbl_file_path, 1)
df_asv = df_asv.astype({col:'int32' for col in df_asv.columns[1:-1] }, copy=Fa
lse)
df_asv
```

Out[2]:

	#OTU ID	1gKO.1	1gKO.2	1gKO.3	1gWT.1	1gWT.2	1gWT.3	2gKO.1
**0**	1ba8c796d07406783c96d016a6a5cace	13615	16637	17148	20227	23630	25656	14832
**1**	a6c38249aff7768283faf6cfbdeb05a8	26439	30129	19743	8955	10759	7074	18489
**2**	062f38ff92cfaee0654200b6f5be5ddf	7451	8774	8754	174	214	148	21958
**3**	1183cc23f552d81e63c93ca9fcba2f2c	225	223	184	13762	16856	18692	269
**4**	5e15ecfb579e72bf87c0bea3920bbf42	10108	12117	8633	10027	11910	7424	5979
**...**	...	...	...	...	...	...	...	...
**4070**	92bb8f4683ef5c8651e7d34dbb37ab2e	0	0	0	0	0	0	0
**4071**	92f09070a4fd5786bb34e756217e6ee1	0	0	0	0	0	0	0
**4072**	919b82324c41ed0046323c63aa1550da	0	0	0	0	0	0	0
**4073**	dbc0dad15ec1c8ad9d826cab94e18696	0	0	0	0	0	0	0

**4074**	1ff2d07d10264c23dc43e08d3097cd7c	0	0	0	0	0	0	0

4075 rows × 112 columns

Step 2: Retrieve original metadata table

In [3]:
```
df_metadata = read_csv_file(metadata_file_path)
df_metadata
```

Out[3]:

	SampleID	UniversalCageNumber	Background	FMTGroupFMTsourcegtRecipientbackground	Passage
**0**	F8-1	F8-cage-1	129.IL10KO	1gKOgtKO	8
**1**	F8-2	F8-cage-1	129.IL10KO	1gKOgtKO	8
**2**	F8-3	F8-cage-2	129.IL10KO	1gKOgtKO	8
**3**	F8-4	F8-cage-2	129.IL10KO	1gKOgtKO	8
**4**	F8-5	F8-cage-3	129.IL10KO	1gKOgtKO	8
**...**	...	...	...	...	...
**105**	1gWT.2	NaN	NaN	1gWTinput	1gWT
**106**	1gWT.3	NaN	NaN	1gWTinput	1gWT
**107**	2gWT.1	NaN	NaN	2gWTinput	2gWT
**108**	2gWT.2	NaN	NaN	2gWTinput	2gWT
**109**	2gWT.3	NaN	NaN	2gWTinput	2gWT

110 rows × 8 columns

Step 3: Create dictionary from metadata table

In [4]:
```
dict_metadata = get_dict_from_metadata(df_metadata)
dict_metadata.keys()
```

Out[4]:
```
dict_keys(['1gKOgtKO', '2gKOgtKO', '1gWTgtKO', '1gWTgtWT', '2gWTgtWT', 'hFM
T.1.2.3.gtKO', 'hFMT.3.4.5.gtKO', 'hFMT.1.2.3.gtWT', 'hFMT.1.2.3.input', 'hF
MT.3.4.5.input', '1gKOinput', '2gKOinput', '1gWTinput', '2gWTinput'])
```

Step 4: Calculate total read counts per sample

In [5]:
```
sample_count_sums = df_asv.iloc[:, 1:-1].sum(axis=0)
sample_count_sums
```

Out[5]:
```
1gKO.1       118256
1gKO.2       141891
1gKO.3       123292
1gWT.1       119717
1gWT.2       146158
               ...
h1-2-3.2     135326
h1-2-3.3     133745
```

```
h3-4-5.1    129613
h3-4-5.2    140316
h3-4-5.3    132984
Length: 110, dtype: int64
```

Step 5: Extract counts for all six sample groups

In [6]:

```python
# 'hFMT.1.2.3.gtWT' -->  'HM1->WT'
key_name = 'hFMT.1.2.3.gtWT'
print('Grouped columns: {}'.format(dict_metadata[key_name]))
print('Number of expected correlation values: {}'.format(int(comb(len(dict_metadata[key_name]), 2))))
HM1_WT = df_asv[[col_name for col_name in dict_metadata[key_name] ]]
HM1_WT
```

```
Grouped columns: ['F1-16', 'F1-17', 'F1-18', 'F1-19', 'F1-20', 'F1-21', 'F1-22']
Number of expected correlation values: 21
```

Out[6]:

	F1-16	F1-17	F1-18	F1-19	F1-20	F1-21	F1-22
**0**	19186	10727	29599	32588	32950	18853	5199
**1**	2938	6816	3233	1118	1710	1047	2202
**2**	9072	10348	5289	176	196	207	269
**3**	247	258	275	18297	22544	19687	21014
**4**	2389	3858	1904	3904	2928	5286	4884
**...**	...	...	...	...	...	...	...
**4070**	0	0	0	0	0	0	0
**4071**	0	0	0	0	0	0	0
**4072**	0	0	0	0	0	0	0
**4073**	0	0	0	0	0	0	0
**4074**	0	0	0	0	0	0	0

4075 rows × 7 columns

In [7]:

```python
# '1gWTgtWT' --> 'NIMM-g1->WT'
key_name = '1gWTgtWT'
print('Grouped columns: {}'.format(dict_metadata[key_name]))
print('Number of expected correlation values: {}'.format(int(comb(len(dict_metadata[key_name]), 2))))

NIMM_g1_WT = df_asv[[col_name for col_name in dict_metadata[key_name] ]]
NIMM_g1_WT
```

```
Grouped columns: ['F8-27', 'F8-28', 'F8-29', 'F8-30', 'F8-31', 'F8-32', 'F8-33', 'F11-1', 'F11-2', 'F11-3', 'F11-4', 'F11-5', 'F11-6', 'F11-7', 'F11-8', 'F11-9', 'F11-10', 'F11-11']
Number of expected correlation values: 153
```

Out[7]:

	F8-27	F8-28	F8-29	F8-30	F8-31	F8-32	F8-33	F11-1	F11-2	F11-3	F11-4	F11-5	F11-6	F
**0**	21313	6113	24573	19347	18028	10482	11761	34742	24360	31173	45582	18929	22349	1
**1**	[illegible]	[illegible]	[illegible]	[illegible]	[illegible]	[illegible]	[illegible]	[illegible]	[illegible]	[illegible]	[illegible]	[illegible]	[illegible]	[illegible]

**1**	300	972	397	332	573	825	1995	410	434	376	376	370	337	
**2**	135	243	197	193	99	207	237	129	291	167	282	412	190	
**3**	21071	16272	27247	21302	26135	23762	22465	25848	26231	19490	22181	18524	12915	2
**4**	2245	5227	5760	1189	2327	14279	7911	1416	2031	1899	2725	2679	1920	[illegible]
**...**	...	...	...	...	...	...	...	...	...	...	...	...	...	
**4070**	0	0	0	0	0	0	0	0	0	0	0	0	0	
**4071**	0	0	0	0	0	0	0	0	0	0	0	0	0	
**4072**	0	0	0	0	0	0	0	0	0	0	0	0	0	
**4073**	0	0	0	0	0	0	0	0	0	0	0	0	0	
**4074**	0	0	0	0	0	0	0	0	0	0	0	0	0	

4075 rows × 18 columns

In [8]:
```python
# '2gWTgtWT' --> 'NIMM-g2->WT'
key_name = '2gWTgtWT'
print('Grouped columns: {}'.format(dict_metadata[key_name]))
print('Number of expected correlation values: {}'.format(int(comb(len(dict_met
adata[key_name]), 2))))
NIMM_g2_WT = df_asv[[col_name for col_name in dict_metadata[key_name] ]]
NIMM_g2_WT
```

```
Grouped columns: ['F8-34', 'F8-35', 'F8-36', 'F8-37', 'F8-38', 'F8-39', 'F8-
40', 'F8-41']
Number of expected correlation values: 28
```

Out[8]:

	F8-34	F8-35	F8-36	F8-37	F8-38	F8-39	F8-40	F8-41
**0**	23089	23716	26435	13911	32356	18934	23958	18944
**1**	329	537	320	362	473	1848	258	282
**2**	172	254	238	146	225	135	189	66
**3**	11455	20177	11798	13910	13422	24886	20107	18889
**4**	4360	10659	3839	3613	5501	13735	2324	12215
**...**	...	...	...	...	...	...	...	...
**4070**	0	0	0	0	0	0	0	0
**4071**	0	0	0	0	0	0	0	0
**4072**	0	0	0	0	0	0	0	0
**4073**	0	0	0	0	0	0	0	0
**4074**	0	0	0	0	0	0	0	0

4075 rows × 8 columns

In [9]:
```python
# 'hFMT.1.2.3.gtKO' --> 'HM1->KO'
key_name = 'hFMT.1.2.3.gtKO'
print('Grouped columns: {}'.format(dict_metadata[key_name]))
print('Number of expected correlation values: {}'.format(int(comb(len(dict_met
adata[key_name]), 2))))
HM1_KO = df_asv[[col_name for col_name in dict_metadata[key_name] ]]
HM1_KO
```

```
Grouped columns: ['F3-7', 'F3-8', 'F3-9', 'F3-10', 'F3-11', 'F3-12', 'F1-7',
'F1-8', 'F1-9', 'F1-10', 'F1-11', 'F1-12', 'F1-13', 'F1-14', 'F1-15']
```

```
Number of expected correlation values: 105
```

Out[9]:

	F3-7	F3-8	F3-9	F3-10	F3-11	F3-12	F1-7	F1-8	F1-9	F1-10	F1-11	F1-12	F1-13	F
**0**	10097	574	12959	18737	17857	12650	10738	9576	7304	14409	14382	8787	7694	
**1**	35102	51633	31840	22887	20446	33188	23565	21707	12802	26719	5701	15715	25125	
**2**	1328	91	2203	189	182	244	33299	13739	53077	17570	34094	18922	19204	4
**3**	137	315	305	134	221	290	203	348	363	238	219	205	255	
**4**	34960	13573	4109	3811	1155	28938	1144	6973	666	8230	1287	11346	13195	[illegible]
**...**	...	...	...	...	...	...	...	...	...	...	...	...	...	
**4070**	0	0	0	0	0	0	0	0	0	0	0	0	0	
**4071**	0	0	0	0	0	0	0	0	0	0	0	0	0	
**4072**	0	0	0	0	0	0	0	0	0	0	0	0	0	
**4073**	0	0	0	0	0	0	0	0	0	0	0	0	0	
**4074**	0	0	0	0	0	0	0	0	0	0	0	0	0	

4075 rows × 15 columns

In [10]:

```
# '1gKOgtKO' --> 'IMM-g1->KO'
key_name = '1gKOgtKO'
print('Grouped columns: {}'.format(dict_metadata[key_name]))
print('Number of expected correlation values: {}'.format(int(comb(len(dict_met
adata[key_name]), 2))))
IMM_g1_KO = df_asv[[col_name for col_name in dict_metadata[key_name] ]]
IMM_g1_KO
```

```
Grouped columns: ['F8-1', 'F8-2', 'F8-3', 'F8-4', 'F8-5', 'F8-6', 'F8-7', 'F
8-8', 'F4-1', 'F4-2', 'F4-3', 'F4-4', 'F4-5', 'F3-1', 'F3-2', 'F3-4', 'F3-
5', 'F3-6']
Number of expected correlation values: 153
```

Out[10]:

	F8-1	F8-2	F8-3	F8-4	F8-5	F8-6	F8-7	F8-8	F4-1	F4-2	F4-3	F4-4	F4-5	
**0**	3339	15223	11423	18205	12486	10761	9339	466	29629	20134	27588	26741	25644	2
**1**	10283	6602	9314	13651	8789	16118	12045	13841	3687	6998	6904	7796	4011	
**2**	41532	40602	52337	11038	16781	8926	25758	31166	1627	37042	23825	26508	30376	
**3**	240	276	331	302	341	231	226	306	275	198	213	271	172	2
**4**	1806	6778	6725	12734	3901	10948	10347	1234	8891	3718	9188	4419	8315	
**...**	...	...	...	...	...	...	...	...	...	...	...	...	...	
**4070**	0	0	0	0	0	0	0	0	0	0	0	0	0	
**4071**	0	0	0	0	0	0	0	0	0	0	0	0	0	
**4072**	0	0	0	0	0	0	0	0	0	0	0	0	0	
**4073**	0	0	0	0	0	0	0	0	0	0	0	0	0	
**4074**	0	0	0	0	0	0	0	0	0	0	0	0	0	

4075 rows × 18 columns

In [11]:

```
# '2gKOgtKO' --> 'IMM-g2->KO'
key_name = '2gKOgtKO'
```

```
print('Grouped columns: {}'.format(dict_metadata[key_name]))
print('Number of expected correlation values: {}'.format(int(comb(len(dict_met
adata[key_name]), 2))))
IMM_g2_KO = df_asv[[col_name for col_name in dict_metadata[key_name] ]]
IMM_g2_KO
```

```
Grouped columns: ['F8-9', 'F8-10', 'F8-11', 'F8-12', 'F8-13', 'F8-14', 'F8-1
5', 'F8-16']
Number of expected correlation values: 28
```

Out[11]:

	F8-9	F8-10	F8-11	F8-12	F8-13	F8-14	F8-15	F8-16
0	23451	24742	31312	13542	4593	10228	14477	23819
1	8030	14203	27872	26539	12663	13720	23316	14430
2	19653	22616	4603	28535	20096	20133	14650	24062
3	266	344	300	269	257	330	249	226
4	6490	6143	12353	2229	6367	11645	9607	10622
...	...	...	...	...	...	...	...	...
4070	0	0	0	0	0	0	0	0
4071	0	0	0	0	0	0	0	0
4072	0	0	0	0	0	0	0	0
4073	0	0	0	0	0	0	0	0
4074	0	0	0	0	0	0	0	0

4075 rows × 8 columns

Step 6: Calculate Spearman Correlation Coefficients for each sample group

In [12]:

```
HM1_WT_data = get_pearson_corr_values(HM1_WT, sample_count_sums)
NIMM_g1_WT_data = get_pearson_corr_values(NIMM_g1_WT, sample_count_sums)
NIMM_g2_WT_data = get_pearson_corr_values(NIMM_g2_WT, sample_count_sums)
HM1_KO_data = get_pearson_corr_values(HM1_KO, sample_count_sums)
IMM_g1_KO_data = get_pearson_corr_values(IMM_g1_KO, sample_count_sums)
IMM_g2_KO_data = get_pearson_corr_values(IMM_g2_KO, sample_count_sums)
```

Step 7: Generate final figure

In [13]:

```
data = [HM1_WT_data,
        NIMM_g1_WT_data,
        NIMM_g2_WT_data,
        HM1_KO_data,
        IMM_g1_KO_data,
       IMM_g2_KO_data]

sample_groups = ['HM->WT',
                'NIMM-g1->WT',
                 'NIMM-g2->WT',
                 'HM1->KO',
                 'IMM-g1->KO',
                 'IMM-g2->KO']
```

```
# Multiple box plots on one Axes
fig, ax = plt.subplots()
ax.boxplot(data, widths=0.7)
ax.set_title('Regenerated Figure 1D')
ax.set_yticks([0.6, 0.7, 0.8, 0.9])
ax.set_xticklabels(sample_groups,
                   rotation=45, fontsize=12)

plt.show()
```

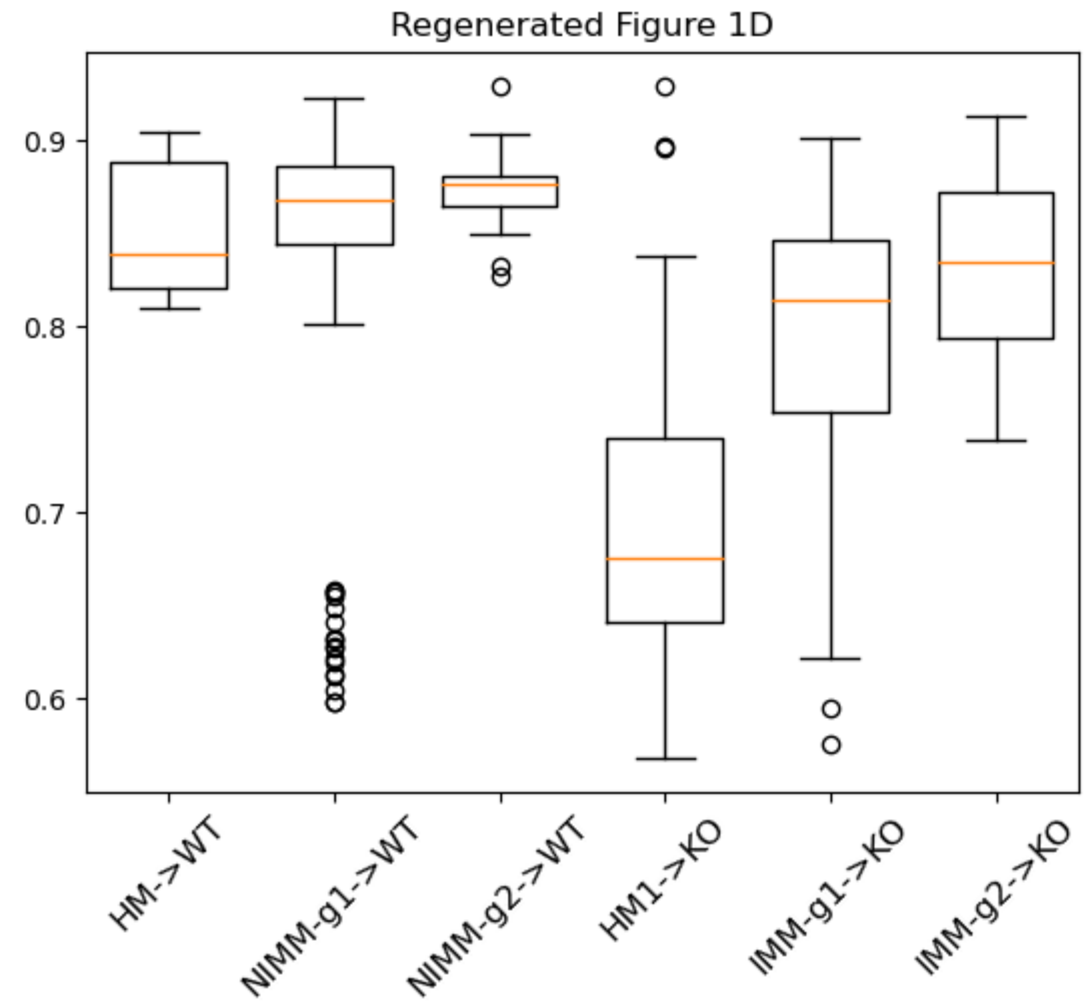

Step 8: Compare original and regenerated figures

1D

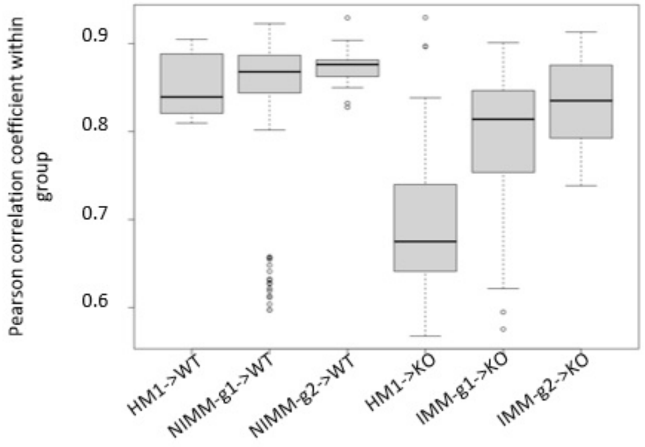

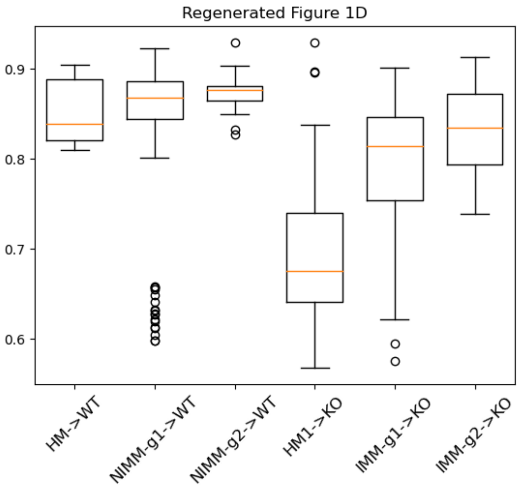

Contents

- **Aim**
- **Main Python Methods**
- **Data Processing Steps**
    - Step 1: Retrieve original metadata table
    - Step 2: Create dictionary from metadata table
    - Step 3: Retrieve feature counts table with filtered sample columns
    - Steo 4: Calculate total read counts per sample
    - Step 5: Generate normalized counts feature table
    - Step 6: Generate Bray-Curtis dissimilarity distance matrix
    - Step 7: Perform Principal Coordinate Analysis (PCoA)
    - Step 8: Plot PCoA Results
    - Step 9: Compare Original and Regenerated Figures

Aim: Replicate Figure 3A

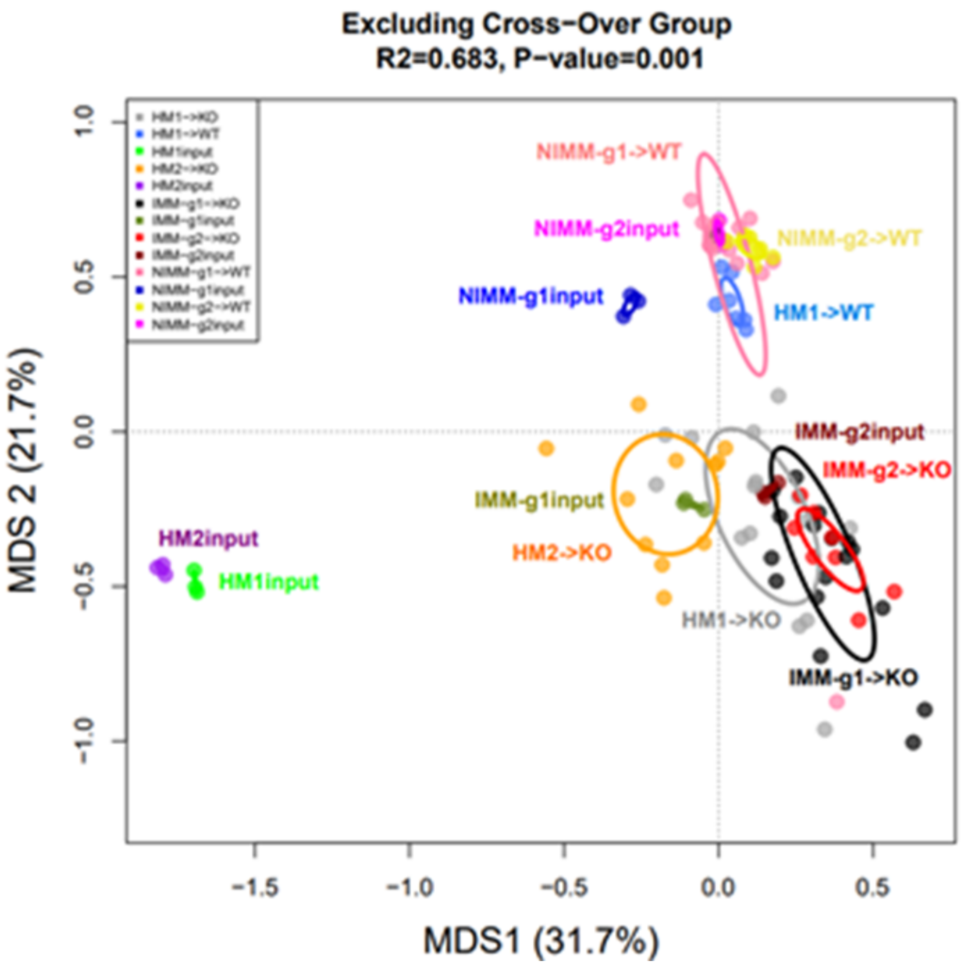

Main Python Methods

In [1]:

```
import numpy as np
import pandas as pd
```

```
import matplotlib.pyplot as plt
import os
import re
import skbio
from scipy import stats
from scipy.spatial import distance
from matplotlib.patches import Ellipse
import matplotlib.transforms as transforms

# ******************************** MAIN METHODS ********************************
# Retrieve tab delimited file
def read_csv_file(file_path, skiprows=None, header = 0, sep = '\t', index_col=
False):
    df = pd.read_csv(file_path, skiprows=skiprows, sep=sep, header=header, ind
ex_col=index_col)

    return df

# Create dictionary from metadata table
def get_dict_from_metadata(input_df):
    mydict = {}
    for row in input_df.iterrows():
        obj = row[1]
        sample_id = obj['SampleID']
        key = obj['FMTGroupFMTsourcegtRecipientbackground']
        if key not in mydict:
            mydict[key] = [sample_id]
        else:
            mydict[key].append(sample_id)
    return mydict

def write_dataframe(input_df, file_path):
    input_df.to_csv(file_path, sep='\t', header=True, index=False)

# Reference:  Function "get_cov_ellipse" extracted from the following URL:
# https://scipython.com/book/chapter-7-matplotlib/examples/bmi-data-with-confi
dence-ellipses/
def get_cov_ellipse(cov, centre, nstd, **kwargs):
    """
    Return a matplotlib Ellipse patch representing the covariance matrix
    cov centred at centre and scaled by the factor nstd.
    """
    # Find and sort eigenvalues and eigenvectors into descending order
    eigvals, eigvecs = np.linalg.eigh(cov)
    order = eigvals.argsort()[::-1]
    eigvals, eigvecs = eigvals[order], eigvecs[:, order]

    # The anti-clockwise angle to rotate our ellipse by
    vx, vy = eigvecs[:,0][0], eigvecs[:,0][1]
    theta = np.arctan2(vy, vx)

    # Width and height of ellipse to draw
    width, height = 2 * nstd * np.sqrt(eigvals)
    return Ellipse(xy=centre, width=width, height=height,
                   angle=np.degrees(theta), fill=False, **kwargs)

# ******************************** INPUT FILE PATHS ****************************
****
# Get current working directory
current_working_dir = os.getcwd()
# original counts table
feature_tbl_file_path = os.path.join(current_working_dir, 'feature-table_balfo
```

```
ur.txt')
# original metadata table
metadata_file_path = os.path.join(current_working_dir, 'mappingMetadata.txt')

# cross reference dictionary that maps original sample group nomenclature to r
evised nomenclature
cross_ref_dict = { '1gKOgtKO':'IMM-g1->KO',
                   '2gKOgtKO':'IMM-g2->KO',
                   '1gWTgtKO':'NIMM-g1->KO',
                   '1gWTgtWT':'NIMM-g1->WT',
                   '2gWTgtWT':'NIMM-g2->WT',
                   'hFMT.1.2.3.gtKO':'HM1->KO',
                   'hFMT.3.4.5.gtKO':'HM2->KO',
                   'hFMT.1.2.3.gtWT':'HM1->WT',
                   'hFMT.1.2.3.input':'HM1input',
                   'hFMT.3.4.5.input': 'HM2input',
                   '1gKOinput':'IMM-g1input',
                   '2gKOinput':'IMM-g2input',
                   '1gWTinput':'NIMM-g1input',
                   '2gWTinput':'NIMM-g2input'
                 }

# target sample group names for Figure 3A
dict_keys_ordered = ['hFMT.1.2.3.gtKO',
                     'hFMT.1.2.3.gtWT',
                     'hFMT.1.2.3.input',
                     'hFMT.3.4.5.gtKO',
                     'hFMT.3.4.5.input',
                     '1gKOgtKO',
                     '1gKOinput',
                     '2gKOgtKO',
                     '2gKOinput',
                     '1gWTgtWT',
                     '1gWTinput',
                     '2gWTgtWT',
                     '2gWTinput']
```

Data Processing Steps

Step 1: Retrieve original metadata table

In [2]:
```
df_metadata = read_csv_file(metadata_file_path)
df_metadata
```

Out[2]:

	SampleID	UniversalCageNumber	Background	FMTGroupFMTsourcegtRecipientbackground	Passage
**0**	F8-1	F8-cage-1	129.IL10KO	1gKOgtKO	8
**1**	F8-2	F8-cage-1	129.IL10KO	1gKOgtKO	8
**2**	F8-3	F8-cage-2	129.IL10KO	1gKOgtKO	8
**3**	F8-4	F8-cage-2	129.IL10KO	1gKOgtKO	8
**4**	F8-5	F8-cage-3	129.IL10KO	1gKOgtKO	8
**...**	...	...	...	...	...
**105**	1gWT.2	NaN	NaN	1gWTinput	1gWT
**106**	1gWT.3	NaN	NaN	1gWTinput	1gWT

**107**	2gWT.1	NaN	NaN		2gWTinput	2gWT
**108**	2gWT.2	NaN	NaN		2gWTinput	2gWT
**109**	2gWT.3	NaN	NaN		2gWTinput	2gWT

110 rows × 8 columns

Step 2: Create dictionary from metadata table

In [3]:

```
dict_metadata = get_dict_from_metadata(df_metadata)
dict_keys_filtered = dict_keys_ordered
filtered_columns = [col for sublist in [dict_metadata[key] for key in dict_key
s_filtered] for col in sublist]
dict_keys_filtered
```

Out[3]:

```
['hFMT.1.2.3.gtKO',
 'hFMT.1.2.3.gtWT',
 'hFMT.1.2.3.input',
 'hFMT.3.4.5.gtKO',
 'hFMT.3.4.5.input',
 '1gKOgtKO',
 '1gKOinput',
 '2gKOgtKO',
 '2gKOinput',
 '1gWTgtWT',
 '1gWTinput',
 '2gWTgtWT',
 '2gWTinput']
```

Step 3: Retrieve feature counts table with filtered sample columns

In [4]:

```
df_feature_counts = read_csv_file(feature_tbl_file_path, 1)
df_feature_counts = df_feature_counts.astype({col:'int32' for col in df_featur
e_counts.columns[1:]})
df_feature_counts
```

Out[4]:

	#OTU ID	1gKO.1	1gKO.2	1gKO.3	1gWT.1	1gWT.2	1gWT.:
**0**	d__Bacteria;p__Verrucomicrobiota;c__Verrucomic...	11370	14120	14648	16632	19817	2170(
**1**	d__Bacteria;p__Firmicutes;c__Clostridia;o__Lac...	22545	25836	17048	7547	9272	599(
**2**	d__Bacteria;p__Proteobacteria;c__Gammaproteoba...	5919	6481	6714	31	31	3(
**3**	d__Bacteria;p__Firmicutes;c__Clostridia;o__Lac...	8134	9827	7090	8264	10100	632
**4**	d__Bacteria;p__Firmicutes;c__Clostridia;o__Lac...	6280	7649	5719	9715	11851	804
**...**	...	...	...	...	...	...	..
**191**	d__Bacteria;p__Firmicutes;c__Clostridia;o__Pep...	0	0	0	0	0	(
**192**	d__Bacteria;p__Firmicutes;c__Clostridia;o__Clo...	0	0	0	0	0	(
**193**	d__Bacteria;p__Firmicutes;c__Clostridia;o__Chr...	0	0	0	0	0	(
**194**	d__Bacteria;p__Firmicutes;c__Incertae_Sedis;o_...	0	0	0	0	0	(

**195**	d__Bacteria;p__Firmicutes;c__Bacilli;o__Lactob...	0	0	0	0	0	[illegible]

196 rows × 111 columns

In [5]:
```
df_feature_counts_filtered = df_feature_counts[filtered_columns]
df_feature_counts_filtered
```

Out[5]:

	F3-7	F3-8	F3-9	F3-10	F3-11	F3-12	F1-7	F1-8	F1-9	F1-10	...	F8-35	F8-36	F8-37
**0**	8445	319	10551	15735	14058	10445	8869	8035	5953	11460	...	20081	21965	11552
**1**	34435	43961	31682	20382	17233	27241	19991	23011	12533	27427	...	374	113	200
**2**	978	13	1555	34	27	51	24450	10453	39194	12486	...	64	55	14
**3**	28326	10652	3300	3168	900	23043	886	5723	480	6491	...	8678	3211	2961
**4**	3647	10380	3639	3886	3693	3755	655	2478	205	5444	...	6252	5577	7567
**...**	...	...	...	...	...	...	...	...	...	...	...	...	...	...
**191**	0	0	0	0	0	0	0	0	0	0	...	0	0	0
**192**	0	0	0	0	0	0	0	0	0	0	...	0	0	0
**193**	0	0	0	0	0	0	0	0	0	0	...	0	0	0
**194**	0	0	0	0	0	0	0	0	0	0	...	0	0	0
**195**	0	0	0	0	0	0	0	0	0	0	...	0	0	0

196 rows × 103 columns

Step 4: Calculate total read counts per sample

In [6]:
```
sample_count_sums = df_feature_counts_filtered.sum(axis=0)
sample_count_sums
```

Out[6]:
```
F3-7       99046
F3-8      112057
F3-9      121801
F3-10      98907
F3-11      93879
           ...
F8-40      63669
F8-41      72906
2gWT.1     86473
2gWT.2     76015
2gWT.3     78810
Length: 103, dtype: int64
```

Step 5: Generate normalized counts feature table

In [7]:
```
df_feature_counts_filtered_norm = np.log10((df_feature_counts_filtered/sample_
count_sums)*sample_count_sums.mean() + 1)
df_feature_counts_filtered_norm
```

Out[7]:

	F3-7	F3-8	F3-9	F3-10	F3-11	F3-12	F1-7	F1-8	F1-9	F1-1
**0**	3.904009	2.429163	3.910888	4.174860	4.148578	3.988447	3.906037	3.851763	3.766695	4.00565

**1**	4.514368	4.566832	4.388371	4.287234	4.237009	4.404734	4.258967	4.308672	4.089975	4.38462
**2**	2.968161	1.075794	3.079633	1.522678	1.447759	1.686105	4.346407	3.966004	4.585116	4.04289
**3**	4.429556	3.951232	3.406225	3.478894	2.955347	4.332053	2.906082	3.704425	2.674047	3.75881
**4**	3.539416	3.940000	3.448677	3.567585	3.568120	3.544228	2.775080	3.341015	2.305792	3.68243
**...**	...	...	...	...	...	...	...	...	...	.
**191**	0.000000	0.000000	0.000000	0.000000	0.000000	0.000000	0.000000	0.000000	0.000000	0.00000
**192**	0.000000	0.000000	0.000000	0.000000	0.000000	0.000000	0.000000	0.000000	0.000000	0.00000
**193**	0.000000	0.000000	0.000000	0.000000	0.000000	0.000000	0.000000	0.000000	0.000000	0.00000
**194**	0.000000	0.000000	0.000000	0.000000	0.000000	0.000000	0.000000	0.000000	0.000000	0.00000
**195**	0.000000	0.000000	0.000000	0.000000	0.000000	0.000000	0.000000	0.000000	0.000000	0.00000

196 rows × 103 columns

Step 6: Generate Bray-Curtis dissimilarity distance matrix

In [8]:

```
col_name_list = df_feature_counts_filtered_norm.columns
num_of_cols = len(col_name_list)
dist_mat = np.zeros((num_of_cols, num_of_cols))
tup_list = [(col_name_list.get_loc(col2), col_name_list.get_loc(col1))  for co
l1 in col_name_list for col2 in col_name_list if col_name_list.get_loc(col2) >
 col_name_list.get_loc(col1)]

for tup in tup_list:
    col_1 = tup[0]
    col_2 = tup[1]
    dist = distance.braycurtis(df_feature_counts_filtered_norm.iloc[:, col_1],
 df_feature_counts_filtered_norm.iloc[:, col_2])
    dist_mat[col_1, col_2] = dist

dist_mat_sym = dist_mat + dist_mat.T
print('Dimensions of matrix: {}'.format(dist_mat_sym.shape))
dist_mat_sym
```

```
Dimensions of matrix: (103, 103)
```

Out[8]:

```
array([[0.        , 0.17459747, 0.34079072, ..., 0.32584445, 0.34141039,
        0.35352788],
       [0.17459747, 0.        , 0.38958066, ..., 0.34970683, 0.36941559,
        0.37073948],
       [0.34079072, 0.38958066, 0.        , ..., 0.30164799, 0.30201668,
        0.31426109],
       ...,
       [0.32584445, 0.34970683, 0.30164799, ..., 0.        , 0.03575365,
        0.04063024],
       [0.34141039, 0.36941559, 0.30201668, ..., 0.03575365, 0.        ,
        0.03552113],
       [0.35352788, 0.37073948, 0.31426109, ..., 0.04063024, 0.03552113,
        0.        ]])
```

Step 7: Perform Principal Coordinate Analysis (PCoA)

In [9]:
```
my_pcoa = skbio.stats.ordination.pcoa(dist_mat_sym)
df_pcoa = my_pcoa.samples[['PC1', 'PC2']]
my_pcoa.proportion_explained
```

```
C:\ProgramData\Anaconda3\lib\site-packages\skbio\stats\ordination\_principal
_coordinate_analysis.py:143: RuntimeWarning: The result contains negative ei
genvalues. Please compare their magnitude with the magnitude of some of the
largest positive eigenvalues. If the negative ones are smaller, it's probabl
y safe to ignore them, but if they are large in magnitude, the results won't
be useful. See the Notes section for more details. The smallest eigenvalue i
s -0.03986325960166524 and the largest is 1.9638240904410669.
  warn(
```

Out[9]:
```
PC1      0.317344
PC2      0.216448
PC3      0.100538
PC4      0.059908
PC5      0.035616
           ...
PC99     0.000000
PC100    0.000000
PC101    0.000000
PC102    0.000000
PC103    0.000000
Length: 103, dtype: float64
```

Step 8: Plot PCoA Results

In [10]:
```
group_counts = [len(dict_metadata[key]) for key in dict_keys_filtered]
group_names = [cross_ref_dict[key] for key in dict_keys_filtered]
colors = ["#A6A6A6", "#4169E1", "#00FF00", "#FFA500", "#A020F0",
                    "#000000", "#6B8E23", "#FF0000", "#8B0000",
                     "#FF82AB", "#0000CD", "#EEEE00", "#FF00FF"]
text_tup = [(-0.05, -0.09),
            (0.04, -0.02),
            (0.02, -0.01),
            (-0.13,-0.06),
            (0.0, 0.02),
            (-0.02, -0.1),
            (-0.175, -0.01),
            (-0.01, 0.06),
            (0.03, 0.04),
            (-0.15, 0.08),
            (-0.13, 0.0),
            (0.03, 0.0),
            (-0.14, 0.0)]

fig, ax = plt.subplots()
ax.set_ylim((-0.3, 0.3))
ax.set_xlabel('MDS1 ({}%)'.format(np.round(my_pcoa.proportion_explained.PC1 *
100, 1)))
ax.set_ylabel('MDS2 ({}%)'.format(np.round(my_pcoa.proportion_explained.PC2 *
100, 1)))
ax.set_title('Regenerated Figure 3A')

end = 0
for idx, group_count in enumerate(group_counts):
    start = end
```

```
    end = start + group_count

    # multiply by -1 to rotate vector 180 degrees in order to match figure 3A
    pc1 = df_pcoa.iloc[start:end, 0] * -1.0
    # multiply by -1 to rotate vector 180 degrees in order to match figure 3A
    pc2 = df_pcoa.iloc[start:end, 1] * -1.0

    group_name = group_names[idx]
    ax.scatter(pc1, pc2, c=colors[idx],  label=group_name,
               alpha=1.0, edgecolors='none')
    cov = np.cov(pc1,pc2)
    x_mean = pc1.mean()
    y_mean = pc2.mean()
    e = get_cov_ellipse(cov, (x_mean, y_mean), 1,
                        ec=colors[idx], linewidth=2.0, alpha=0.4)
    ax.text(x_mean + text_tup[idx][0], y_mean + text_tup[idx][1], group_name,
fontsize='x-small', c=colors[idx])
    ax.add_artist(e)

ax.legend(fontsize=6, loc='upper left')
plt.show()
```

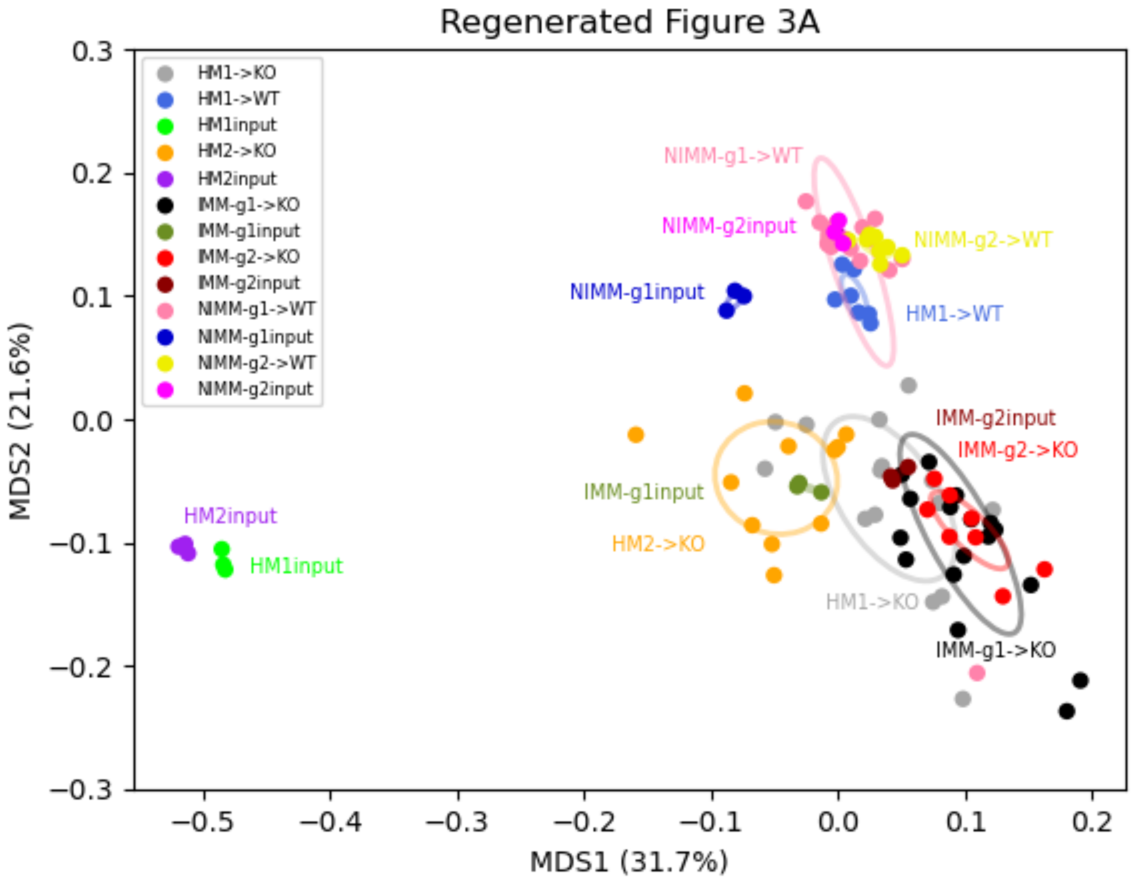

Step 9: Compare Original and Regenerated Figures

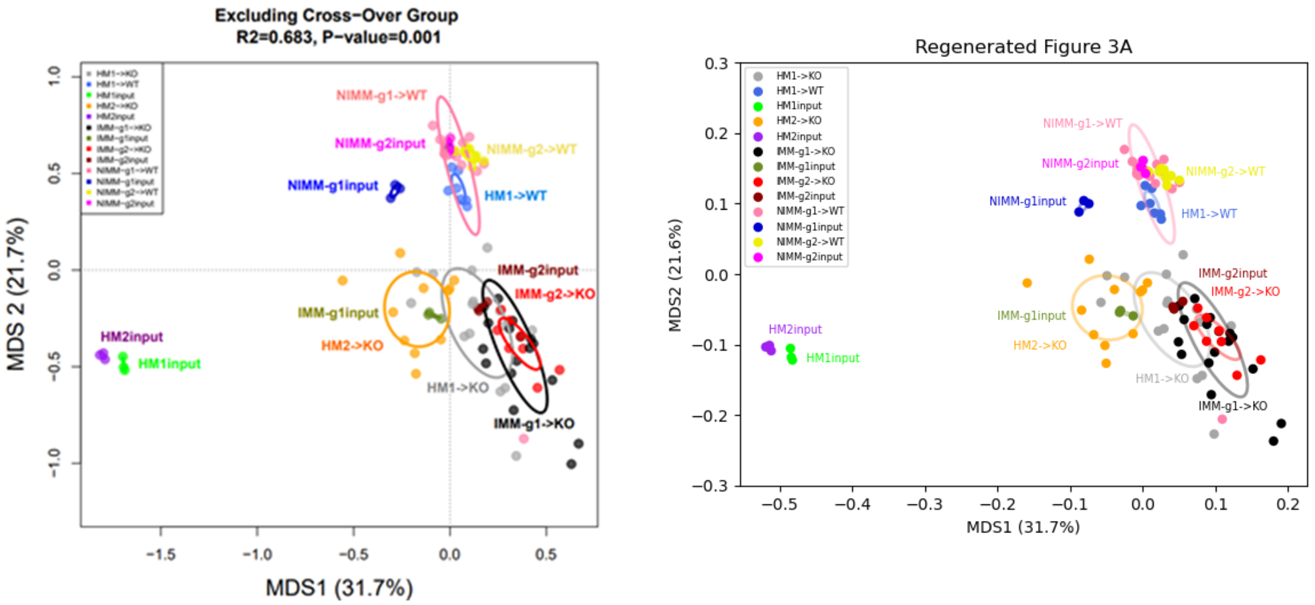
Excluding Cross−Over Group
R2=0.683, P−value=0.001
MDS 2 (21.7%)
MDS1 (31.7%)
NIMM-g1->WT
NIMM-g2input
NIMM-g2->WT
NIMM-g1input
HM1->WT
IMM-g2input
IMM-g2->KO
IMM-g1input
HM2->KO
HM2input
HM1input
HM1->KO
IMM-g1->KO
Regenerated Figure 3A
MDS2 (21.6%)
MDS1 (31.7%)
HM1->KO
HM1->WT
HM1input
HM2->KO
HM2input
IMM-g1->KO
IMM-g1input
IMM-g2->KO
IMM-g2input
NIMM-g1->WT
NIMM-g1input
NIMM-g2->WT
NIMM-g2input

Contents

- **Aim**
- **Main Python Methods**
- **Data Processing Steps**
  - Step 1: Retrieve original counts table
  - Step 2: Retrieve original metadata table
  - Step 3: Create dictionary from metadata table
  - Step 4: Calculate total read counts per sample
  - Step 5: Extract counts for all six sample groups
  - Step 6: Normalize count values for all six sample groups
  - Step 7: Calculate Spearman Correlation Coefficients for each sample group
  - Step 8: Generate final figure
  - Step 9: Compare original and regenerated figures

Aim: Replicate Figure 4B

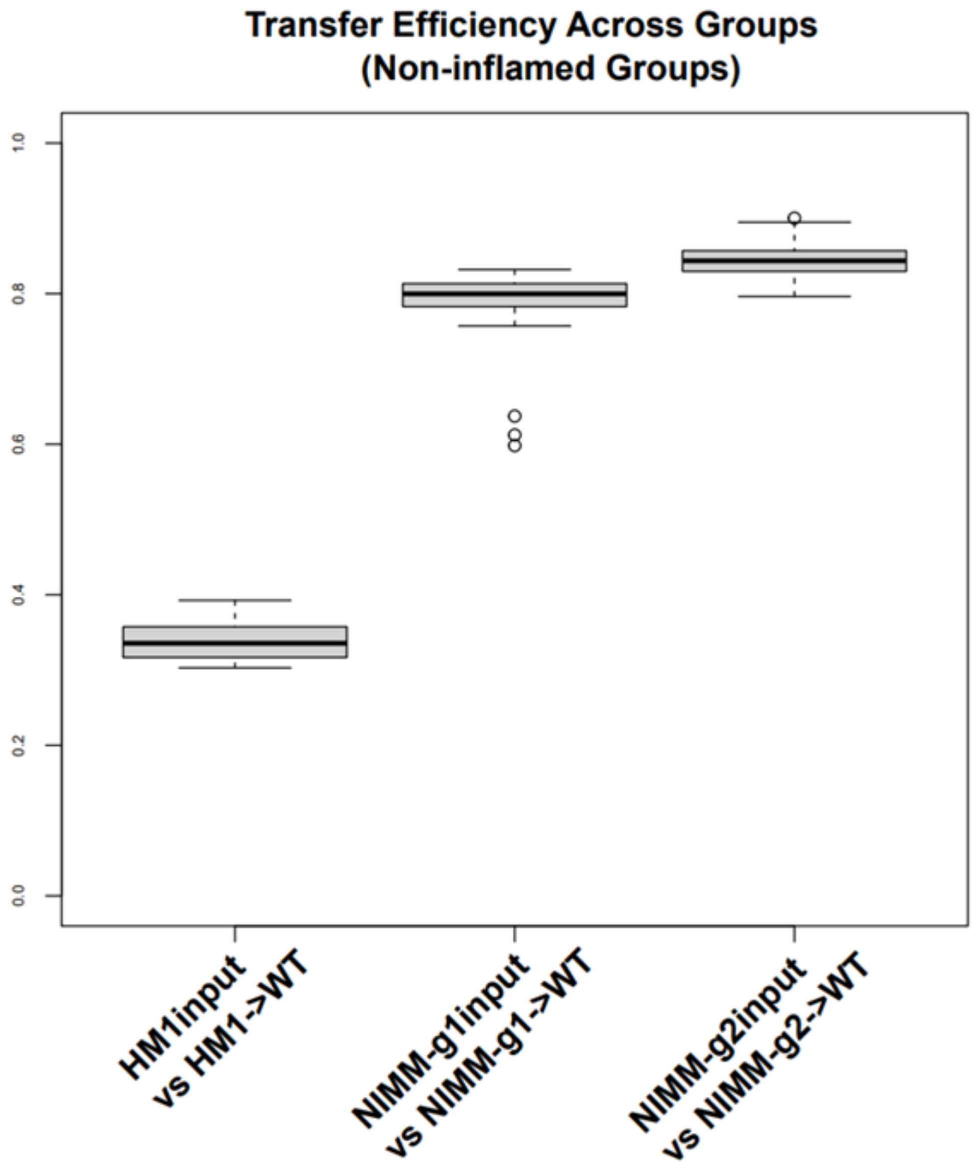

Main Python Methods

In [20]:

```
import numpy as np
import pandas as pd
import matplotlib.pyplot as plt
import os
import re
from scipy import stats
from scipy.special import comb

# ******************************* MAIN METHODS *******************************
# calculate Pearson correlation coefficients from input set of samples
def get_inter_corr_values(df1, df2):
    corr_values = [stats.pearsonr(df1.loc[:, col_A], df2.loc[:, col_B]).statis
tic \
                for col_A in df1.columns for col_B in df2.columns]
    # Remove nan values
    corr_values = [val for val in corr_values if not np.isnan(val)]

    return corr_values

# Retrieve tab delimited file
def read_csv_file(file_path, skiprows=None, header = 0, sep = '\t', index_col=
False):
    df = pd.read_csv(file_path, skiprows=skiprows, sep=sep, header=header, ind
ex_col=index_col)

    return df

# Create dictionary from metadata table
def get_dict_from_metadata(input_df):
    mydict = {}
    for row in input_df.iterrows():
        obj = row[1]
        sample_id = obj['SampleID']
        key = obj['FMTGroupFMTsourcegtRecipientbackground']
        if key not in mydict:
            mydict[key] = [sample_id]
        else:
            mydict[key].append(sample_id)
    return mydict

# ******************************* HELPER METHODS *****************************
**
# Normalize sample counts
def _get_norm_counts(input_df, ser_sample_count_sums):
    overall_mean_count = ser_sample_count_sums.mean()
    df_norm_logged =    pd.DataFrame()
    for col_name in input_df.columns:
        df_norm_logged.loc[:, col_name] = \
        np.log10(((input_df.loc[:, col_name] / ser_sample_count_sums[col_name
]) * overall_mean_count) + 1)

    return df_norm_logged

# Get tuples of sample column names
def _get_tup_list(col_names):
    tup_list = []
    corr_result = []
    col_name_length = len(col_names)
    for i in range(col_name_length):
```

```
        for j in range(i+1, col_name_length):
            tup_list.append((i, j))

    return tup_list

# ******************************* INPUT FILE PATHS ***************************
****
# Get current working directory
current_working_dir = os.getcwd()
# original counts table
asv_tbl_file_path = os.path.join(current_working_dir, 'asv_biom-with-taxonomy.
txt')
# original metadata table
metadata_file_path = os.path.join(current_working_dir, 'mappingMetadata.txt')
```

Data Processing Steps

Step 1: Retrieve original counts table

In [21]:

```
df_asv = read_csv_file(asv_tbl_file_path, 1)
df_asv = df_asv.astype({col:'int32' for col in df_asv.columns[1:-1] }, copy=Fa
lse)
df_asv
```

Out[21]:

	#OTU ID	1gKO.1	1gKO.2	1gKO.3	1gWT.1	1gWT.2	1gWT.3	2gKO.1
**0**	1ba8c796d07406783c96d016a6a5cace	13615	16637	17148	20227	23630	25656	14832
**1**	a6c38249aff7768283faf6cfbdeb05a8	26439	30129	19743	8955	10759	7074	18489
**2**	062f38ff92cfaee0654200b6f5be5ddf	7451	8774	8754	174	214	148	21958
**3**	1183cc23f552d81e63c93ca9fcba2f2c	225	223	184	13762	16856	18692	269
**4**	5e15ecfb579e72bf87c0bea3920bbf42	10108	12117	8633	10027	11910	7424	5979
**...**	...	...	...	...	...	...	...	...
**4070**	92bb8f4683ef5c8651e7d34dbb37ab2e	0	0	0	0	0	0	0
**4071**	92f09070a4fd5786bb34e756217e6ee1	0	0	0	0	0	0	0
**4072**	919b82324c41ed0046323c63aa1550da	0	0	0	0	0	0	0
**4073**	dbc0dad15ec1c8ad9d826cab94e18696	0	0	0	0	0	0	0
**4074**	1ff2d07d10264c23dc43e08d3097cd7c	0	0	0	0	0	0	0

4075 rows × 112 columns

Step 2: Retrieve original metadata table

In [22]:
```
df_metadata = read_csv_file(metadata_file_path)
df_metadata
```

Out[22]:

	SampleID	UniversalCageNumber	Background	FMTGroupFMTsourcegtRecipientbackground	Passage
**0**	F8-1	F8-cage-1	129.IL10KO	1gKOgtKO	8
**1**	F8-2	F8-cage-1	129.IL10KO	1gKOgtKO	8
**2**	F8-3	F8-cage-2	129.IL10KO	1gKOgtKO	8
**3**	F8-4	F8-cage-2	129.IL10KO	1gKOgtKO	8
**4**	F8-5	F8-cage-3	129.IL10KO	1gKOgtKO	8
**...**	...	...	...	...	...
**105**	1gWT.2	NaN	NaN	1gWTinput	1gWT
**106**	1gWT.3	NaN	NaN	1gWTinput	1gWT
**107**	2gWT.1	NaN	NaN	2gWTinput	2gWT
**108**	2gWT.2	NaN	NaN	2gWTinput	2gWT
**109**	2gWT.3	NaN	NaN	2gWTinput	2gWT

110 rows × 8 columns

Step 3: Create dictionary from metadata table

In [4]:
```
dict_metadata = get_dict_from_metadata(df_metadata)
dict_metadata.keys()
```

Out[4]:
```
dict_keys(['1gKOgtKO', '2gKOgtKO', '1gWTgtKO', '1gWTgtWT', '2gWTgtWT', 'hFM
T.1.2.3.gtKO', 'hFMT.3.4.5.gtKO', 'hFMT.1.2.3.gtWT', 'hFMT.1.2.3.input', 'hF
MT.3.4.5.input', '1gKOinput', '2gKOinput', '1gWTinput', '2gWTinput'])
```

Step 4: Calculate total read counts per sample

In [5]:
```
sample_count_sums = df_asv.iloc[:, 1:-1].sum(axis=0)
sample_count_sums
```

Out[5]:
```
1gKO.1      118256
1gKO.2      141891
1gKO.3      123292
1gWT.1      119717
1gWT.2      146158
             ...
h1-2-3.2    135326
h1-2-3.3    133745
h3-4-5.1    129613
h3-4-5.2    140316
h3-4-5.3    132984
Length: 110, dtype: int64
```

Step 5: Extract counts for target sample groups

In [6]:

```
# 'hFMT.1.2.3.input' -->  'HM1Input'
key_name ='hFMT.1.2.3.input'
print('Grouped columns: {}'.format(dict_metadata[key_name]))
HM1_Input = df_asv[[col_name for col_name in dict_metadata[key_name] ]]
HM1_Input
```

```
Grouped columns: ['h1-2-3.1', 'h1-2-3.2', 'h1-2-3.3']
```

Out[6]:

	h1-2-3.1	h1-2-3.2	h1-2-3.3
**0**	1483	1515	1402
**1**	1129	691	725
**2**	2927	3523	3254
**3**	298	231	238
**4**	218	160	111
**...**	...	...	...
**4070**	0	0	0
**4071**	0	0	0
**4072**	0	0	0
**4073**	0	0	0
**4074**	0	0	0

4075 rows × 3 columns

In [7]:

```
# 'hFMT.1.2.3.gtWT' -->  'HM1->WT'
key_name = 'hFMT.1.2.3.gtWT'
print('Grouped columns: {}'.format(dict_metadata[key_name]))
print('Number of expected correlation values: {}'.format(int(comb(len(dict_met
adata[key_name]), 2))))
HM1_WT = df_asv[[col_name for col_name in dict_metadata[key_name] ]]
HM1_WT
```

```
Grouped columns: ['F1-16', 'F1-17', 'F1-18', 'F1-19', 'F1-20', 'F1-21', 'F1-
22']
Number of expected correlation values: 21
```

Out[7]:

	F1-16	F1-17	F1-18	F1-19	F1-20	F1-21	F1-22
**0**	19186	10727	29599	32588	32950	18853	5199
**1**	2938	6816	3233	1118	1710	1047	2202
**2**	9072	10348	5289	176	196	207	269
**3**	247	258	275	18297	22544	19687	21014
**4**	2389	3858	1904	3904	2928	5286	4884
**...**	...	...	...	...	...	...	...
**4070**	0	0	0	0	0	0	0
**4071**	0	0	0	0	0	0	0
**4072**	0	0	0	0	0	0	0

**4072**	0	0	0	0	0	0	0
**4073**	0	0	0	0	0	0	0
**4074**	0	0	0	0	0	0	0

4075 rows × 7 columns

In [8]:
```
# '1gWTinput' -->  'NIMM-g1input'
key_name ='1gWTinput'
print('Grouped columns: {}'.format(dict_metadata[key_name]))
NIMM_g1input = df_asv[[col_name for col_name in dict_metadata[key_name] ]]
NIMM_g1input
```

```
Grouped columns: ['1gWT.1', '1gWT.2', '1gWT.3']
```

Out[8]:

	1gWT.1	1gWT.2	1gWT.3
**0**	20227	23630	25656
**1**	8955	10759	7074
**2**	174	214	148
**3**	13762	16856	18692
**4**	10027	11910	7424
**...**	...	...	...
**4070**	0	0	0
**4071**	0	0	0
**4072**	0	0	0
**4073**	0	0	0
**4074**	0	0	0

4075 rows × 3 columns

In [9]:
```
# '1gWTgtWT' -->  'NIMM-g1->WT'
key_name ='1gWTgtWT'
print('Grouped columns: {}'.format(dict_metadata[key_name]))
NIMM_g1_WT = df_asv[[col_name for col_name in dict_metadata[key_name] ]]
NIMM_g1_WT
```

```
Grouped columns: ['F8-27', 'F8-28', 'F8-29', 'F8-30', 'F8-31', 'F8-32', 'F8-
33', 'F11-1', 'F11-2', 'F11-3', 'F11-4', 'F11-5', 'F11-6', 'F11-7', 'F11-8',
'F11-9', 'F11-10', 'F11-11']
```

Out[9]:

	F8-27	F8-28	F8-29	F8-30	F8-31	F8-32	F8-33	F11-1	F11-2	F11-3	F11-4	F11-5	F11-6	F
**0**	21313	6113	24573	19347	18028	10482	11761	34742	24360	31173	45582	18929	22349	1
**1**	300	972	397	332	573	825	1995	410	434	376	376	370	337	
**2**	135	243	197	193	99	207	237	129	291	167	282	412	190	
**3**	21071	16272	27247	21302	26135	23762	22465	25848	26231	19490	22181	18524	12915	2
**4**	2245	5227	5760	1189	2327	14279	7911	1416	2031	1899	2725	2679	1920	
**...**	...	...	...	...	...	...	...	...	...	...	...	...	...	
**4070**	0	0	0	0	0	0	0	0	0	0	0	0	0	
**4071**	0	0	0	0	0	0	0	0	0	0	0	0	0	

**4071**	0	0	0	0	0	0	0	0	0	0	0	0	0
**4072**	0	0	0	0	0	0	0	0	0	0	0	0	0
**4073**	0	0	0	0	0	0	0	0	0	0	0	0	0
**4074**	0	0	0	0	0	0	0	0	0	0	0	0	0

4075 rows × 18 columns

In [10]:

```
# '2gWTinput' -->  'NIMM-g2input'
key_name ='2gWTinput'
print('Grouped columns: {}'.format(dict_metadata[key_name]))
NIMM_g2input = df_asv[[col_name for col_name in dict_metadata[key_name] ]]
NIMM_g2input
```

```
Grouped columns: ['2gWT.1', '2gWT.2', '2gWT.3']
```

Out[10]:

	2gWT.1	2gWT.2	2gWT.3
**0**	26154	27846	29545
**1**	487	298	403
**2**	221	152	77
**3**	18419	20399	21089
**4**	2446	1550	1249
**...**	...	...	...
**4070**	0	0	0
**4071**	0	0	0
**4072**	0	0	0
**4073**	0	0	0
**4074**	0	0	0

4075 rows × 3 columns

In [11]:

```
# '2gWTgtWT' -->  'NIMM-g2->WT'
key_name ='2gWTgtWT'
print('Grouped columns: {}'.format(dict_metadata[key_name]))
NIMM_g2_WT = df_asv[[col_name for col_name in dict_metadata[key_name] ]]
NIMM_g2_WT
```

```
Grouped columns: ['F8-34', 'F8-35', 'F8-36', 'F8-37', 'F8-38', 'F8-39', 'F8-
40', 'F8-41']
```

Out[11]:

	F8-34	F8-35	F8-36	F8-37	F8-38	F8-39	F8-40	F8-41
**0**	23089	23716	26435	13911	32356	18934	23958	18944
**1**	329	537	320	362	473	1848	258	282
**2**	172	254	238	146	225	135	189	66
**3**	11455	20177	11798	13910	13422	24886	20107	18889
**4**	4360	10659	3839	3613	5501	13735	2324	12215
**...**	...	...	...	...	...	...	...	...
**4070**	0	0	0	0	0	0	0	0
**4071**	0	0	0	0	0	0	0	0
**4072**	0	0	0	0	0	0	0	0

**4072**	0	0	0	0	0	0	0	0
**4073**	0	0	0	0	0	0	0	0
**4074**	0	0	0	0	0	0	0	0

4075 rows × 8 columns

Step 6: Normalize count values for target sample groups

In [12]:

```
HM1_Input_norm = _get_norm_counts(HM1_Input, sample_count_sums)
HM1_Input_norm
```

Out[12]:

	h1-2-3.1	h1-2-3.2	h1-2-3.3
**0**	3.170992	3.168281	3.139741
**1**	3.052636	2.827698	2.853624
**2**	3.466129	3.534613	3.505231
**3**	2.475230	2.353116	2.371106
**4**	2.340004	2.194478	2.041962
**...**	...	...	...
**4070**	0.000000	0.000000	0.000000
**4071**	0.000000	0.000000	0.000000
**4072**	0.000000	0.000000	0.000000
**4073**	0.000000	0.000000	0.000000
**4074**	0.000000	0.000000	0.000000

4075 rows × 3 columns

In [13]:

```
HM1_WT_norm = _get_norm_counts(HM1_WT, sample_count_sums)
HM1_WT_norm
```

Out[13]:

	F1-16	F1-17	F1-18	F1-19	F1-20	F1-21	F1-22
**0**	4.245267	3.999519	4.464795	4.552695	4.457225	4.238876	3.673620
**1**	3.430471	3.802595	3.503245	3.088422	3.172643	2.983868	3.300643
**2**	3.920013	3.983899	3.716960	2.287385	2.234151	2.281717	2.389137
**3**	2.356868	2.382421	2.434436	4.302027	4.292408	4.257674	4.280140
**4**	3.340672	3.555481	3.273402	3.631237	3.406096	3.686687	3.646482
**...**	...	...	...	...	...	...	...
**4070**	0.000000	0.000000	0.000000	0.000000	0.000000	0.000000	0.000000
**4071**	0.000000	0.000000	0.000000	0.000000	0.000000	0.000000	0.000000
**4072**	0.000000	0.000000	0.000000	0.000000	0.000000	0.000000	0.000000
**4073**	0.000000	0.000000	0.000000	0.000000	0.000000	0.000000	0.000000
**4074**	0.000000	0.000000	0.000000	0.000000	0.000000	0.000000	0.000000

4075 rows × 7 columns

In [14]:
```
NIMM_g1input_norm = _get_norm_counts(NIMM_g1input, sample_count_sums)
NIMM_g1input_norm
```

Out[14]:

	1gWT.1	1gWT.2	1gWT.3
**0**	4.346750	4.327617	4.390865
**1**	3.992909	3.985949	3.831387
**2**	2.283615	2.286796	2.154970
**3**	4.179510	4.180916	4.253338
**4**	4.042010	4.030085	3.852357
**...**	...	...	...
**4070**	0.000000	0.000000	0.000000
**4071**	0.000000	0.000000	0.000000
**4072**	0.000000	0.000000	0.000000
**4073**	0.000000	0.000000	0.000000
**4074**	0.000000	0.000000	0.000000

4075 rows × 3 columns

In [15]:
```
NIMM_g1_WT_norm = _get_norm_counts(NIMM_g1_WT, sample_count_sums)
NIMM_g1_WT_norm
```

Out[15]:

	F8-27	F8-28	F8-29	F8-30	F8-31	F8-32	F8-33	F11-1	F11-2	F11
**0**	4.269712	3.868181	4.305071	4.313638	4.241945	4.043398	4.160521	4.485555	4.333532	4.4697
**1**	2.419820	3.069904	2.514711	2.549369	2.744909	2.939868	3.390166	2.558671	2.585453	2.5523
**2**	2.075047	2.468951	2.211733	2.314670	1.986115	2.340871	2.466282	2.059083	2.412411	2.2014
**3**	4.264752	4.293331	4.349929	4.355443	4.403212	4.398815	4.441569	4.357132	4.365668	4.2658
**4**	3.292482	3.800189	3.675105	3.102528	3.352961	4.177641	3.988323	3.096097	3.254786	3.2547
**...**	...	...	...	...	...	...	...	...	...	
**4070**	0.000000	0.000000	0.000000	0.000000	0.000000	0.000000	0.000000	0.000000	0.000000	0.0000
**4071**	0.000000	0.000000	0.000000	0.000000	0.000000	0.000000	0.000000	0.000000	0.000000	0.0000
**4072**	0.000000	0.000000	0.000000	0.000000	0.000000	0.000000	0.000000	0.000000	0.000000	0.0000
**4073**	0.000000	0.000000	0.000000	0.000000	0.000000	0.000000	0.000000	0.000000	0.000000	0.0000
**4074**	0.000000	0.000000	0.000000	0.000000	0.000000	0.000000	0.000000	0.000000	0.000000	0.0000

4075 rows × 18 columns

In [16]:
```
NIMM_g2input_norm = _get_norm_counts(NIMM_g2input, sample_count_sums)
NIMM_g2input_norm
```

Out[16]:

	2gWT.1	2gWT.2	2gWT.3
**0**	4.410161	4.472699	4.474499
**1**	2.681041	2.503502	2.610372
**2**	2.338993	2.212436	1.896044

**3**	4.257896	4.337551	4.328077
**4**	3.381243	3.218516	3.100908
**...**	...	...	...
**4070**	0.000000	0.000000	0.000000
**4071**	0.000000	0.000000	0.000000
**4072**	0.000000	0.000000	0.000000
**4073**	0.000000	0.000000	0.000000
**4074**	0.000000	0.000000	0.000000

4075 rows × 3 columns

In [17]:
```
NIMM_g2_WT_norm = _get_norm_counts(NIMM_g2_WT, sample_count_sums)
NIMM_g2_WT_norm
```

Out[17]:

	F8-34	F8-35	F8-36	F8-37	F8-38	F8-39	F8-40	F8-41
**0**	4.326180	4.345537	4.473561	4.148775	4.388825	4.288827	4.446174	4.297041
**1**	2.481386	2.701315	2.557721	2.565278	2.554926	3.278493	2.479769	2.471266
**2**	2.201025	2.377136	2.429562	2.172667	2.233579	2.145017	2.345136	1.845337
**3**	4.021791	4.275356	4.123208	4.148744	4.006713	4.407534	4.370073	4.295778
**4**	3.602349	3.998236	3.635686	3.563373	3.619399	4.149421	3.433104	4.106475
**...**	...	...	...	...	...	...	...	...
**4070**	0.000000	0.000000	0.000000	0.000000	0.000000	0.000000	0.000000	0.000000
**4071**	0.000000	0.000000	0.000000	0.000000	0.000000	0.000000	0.000000	0.000000
**4072**	0.000000	0.000000	0.000000	0.000000	0.000000	0.000000	0.000000	0.000000
**4073**	0.000000	0.000000	0.000000	0.000000	0.000000	0.000000	0.000000	0.000000
**4074**	0.000000	0.000000	0.000000	0.000000	0.000000	0.000000	0.000000	0.000000

4075 rows × 8 columns

Step 7: Calculate Spearman Correlation Coefficients for each sample group

In [18]:
```
corr_value1 = get_inter_corr_values(HM1_Input_norm, HM1_WT_norm)
corr_value2 = get_inter_corr_values(NIMM_g1input_norm, NIMM_g1_WT_norm)
corr_value3 = get_inter_corr_values(NIMM_g2input_norm, NIMM_g2_WT_norm)
```

Step 8: Generate final figure

In [25]:
```
data = []
x_tick_labels = ['HM1input\nvs HM1->WT',
                 'NIMM-g1input\nvs NIMM-g1->WT',
                 'NIMM-g2input\nvs NIMM-g2->WT']

data.extend([corr_value1, corr_value2, corr_value3])
fig, ax = plt.subplots()
```

```
ax.boxplot(data, widths=0.7)
ax.set_title('Regenerated Figure 4B')
ax.set_yticks([0.0, 0.2, 0.4, 0.6, 0.8, 1.0])
ax.set_xticklabels(x_tick_labels,
                   rotation=45, fontsize=10, fontweight='bold')

plt.show()
```

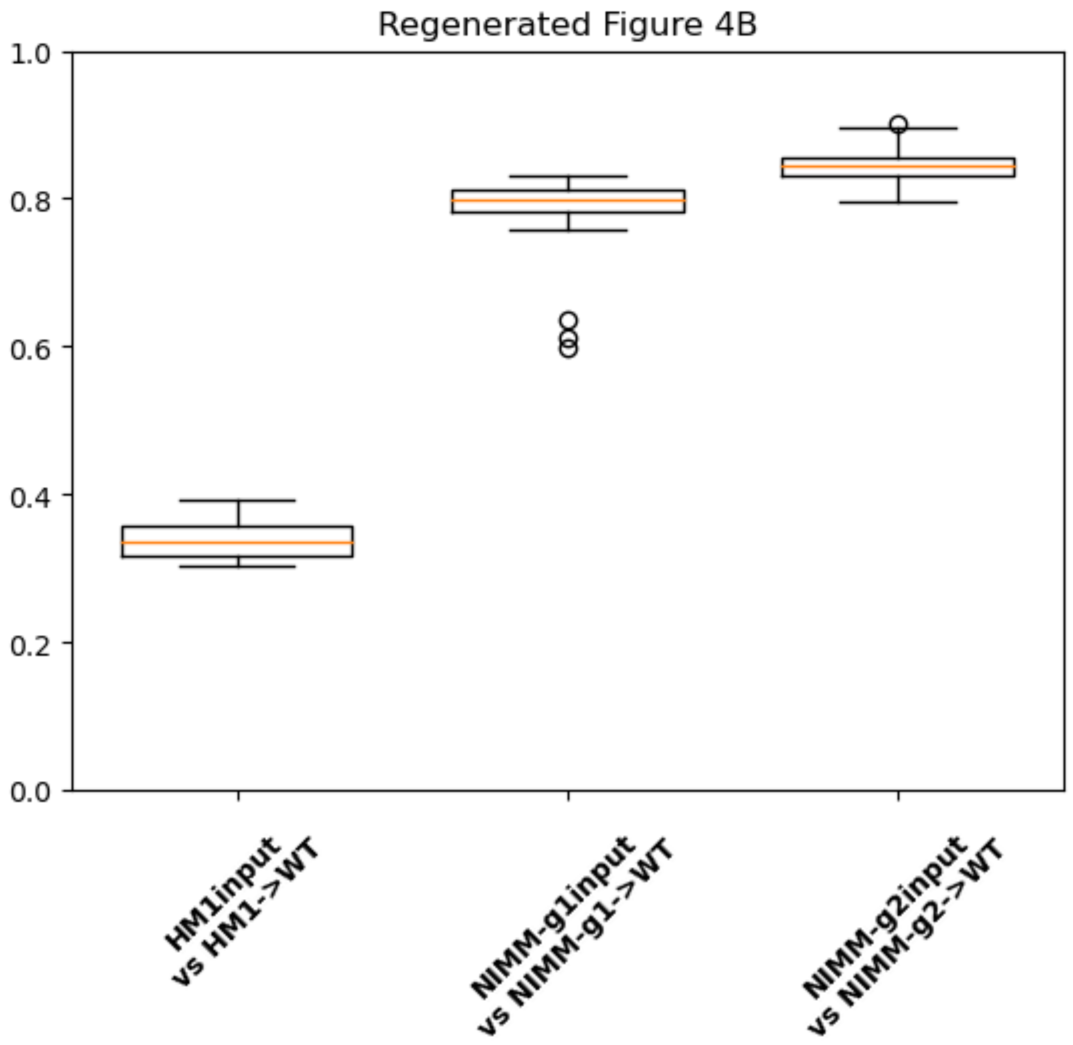

Step 9: Compare original and regenerated figures

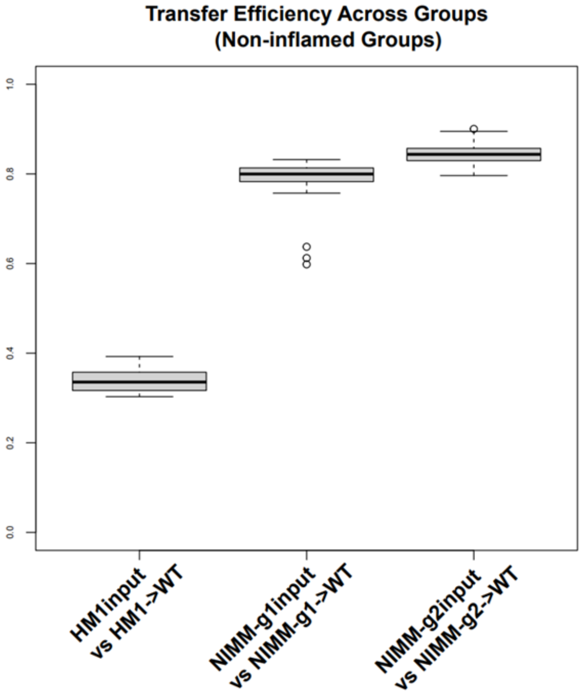
Transfer Efficiency Across Groups
(Non-inflamed Groups)
0.0
0.2
0.4
0.6
0.8
1.0
HM1input vs HM1->WT
NIMM-g1input vs NIMM-g1->WT
NIMM-g2input vs NIMM-g2->WT

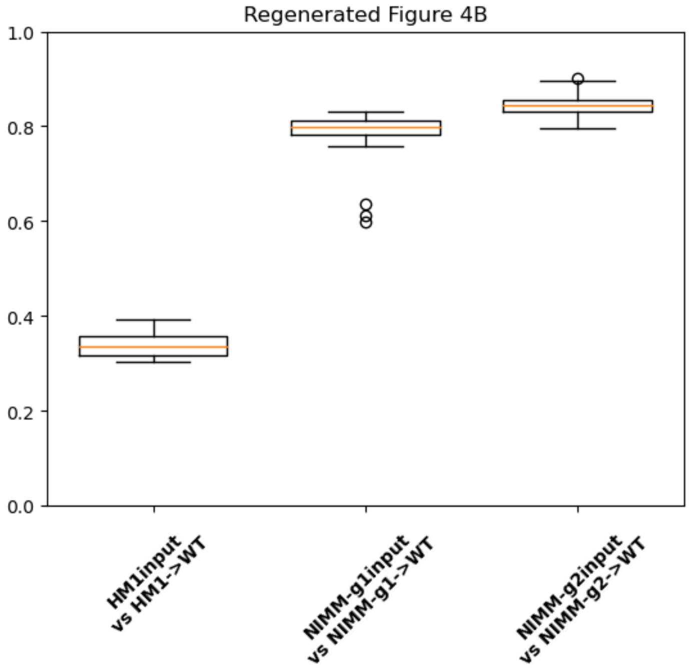
Regenerated Figure 4B
0.0
0.2
0.4
0.6
0.8
1.0
HM1input vs HM1->WT
NIMM-g1input vs NIMM-g1->WT
NIMM-g2input vs NIMM-g2->WT

In [ ]:

Contents

- **Aim**
- **Main Python Methods**
- **Data Processing Steps**
  - Step 1: Retrieve original counts table
  - Step 2: Retrieve original metadata table
  - Step 3: Create dictionary from metadata table
  - Step 4: Calculate total read counts per sample
  - Step 5: Extract counts for all six sample groups
  - Step 6: Normalize count values for all six sample groups
  - Step 7: Calculate Spearman Correlation Coefficients for each sample group
  - Step 8: Generate final figure
  - Step 9: Compare original and regenerated figures

Aim: Replicate Figure 4D

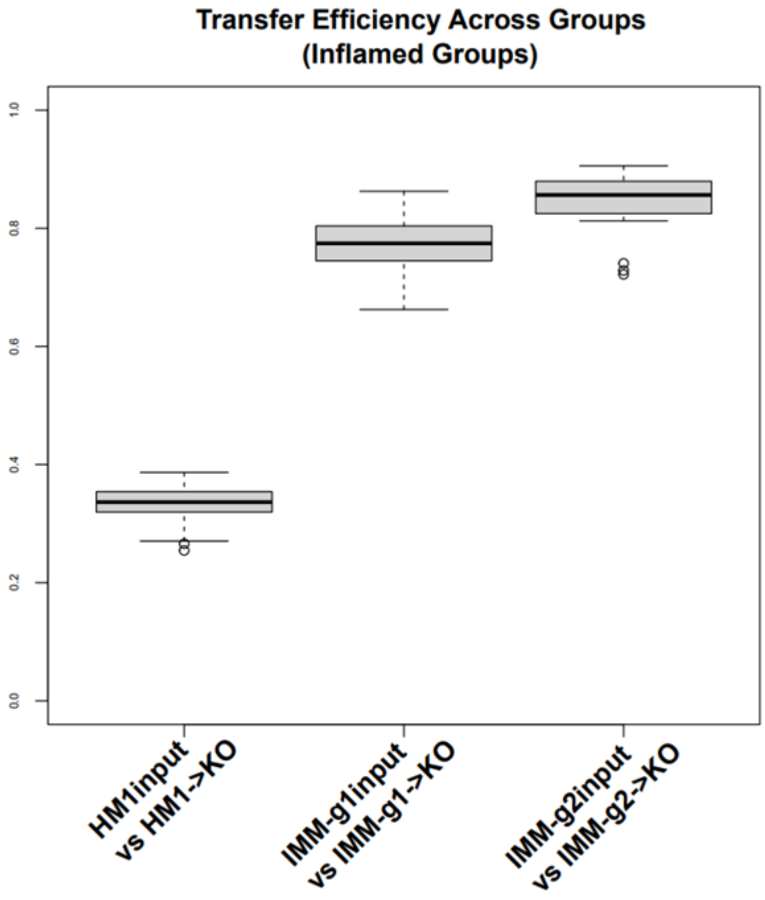

Main Python Methods

In [1]:
```
import numpy as np
import pandas as pd
import matplotlib.pyplot as plt
import os
```

```
import re
from scipy import stats
from scipy.special import comb

# ******************************* MAIN METHODS *******************************
# Calculate Pearson correlation coefficients from input set of samples
def get_inter_corr_values(df1, df2):
    corr_values = [stats.pearsonr(df1.loc[:, col_A], df2.loc[:, col_B]).statis
tic \
            for col_A in df1.columns for col_B in df2.columns]
    # Remove nan values
    corr_values = [val for val in corr_values if not np.isnan(val)]

    return corr_values

# Retrieve tab delimited file
def read_csv_file(file_path, skiprows=None, header = 0, sep = '\t', index_col=
False):
    df = pd.read_csv(file_path, skiprows=skiprows, sep=sep, header=header, ind
ex_col=index_col)

    return df

# Create dictionary from metadata table
def get_dict_from_metadata(input_df):
    mydict = {}
    for row in input_df.iterrows():
        obj = row[1]
        sample_id = obj['SampleID']
        key = obj['FMTGroupFMTsourcegtRecipientbackground']
        if key not in mydict:
            mydict[key] = [sample_id]
        else:
            mydict[key].append(sample_id)
    return mydict

# ******************************* HELPER METHODS *****************************
**
# Normalize values in counts table
def _get_norm_counts(input_df, ser_sample_count_sums):
    overall_mean_count = ser_sample_count_sums.mean()
    df_norm_logged =    pd.DataFrame()
    for col_name in input_df.columns:
        df_norm_logged.loc[:, col_name] = \
        np.log10(((input_df.loc[:, col_name] / ser_sample_count_sums[col_name
]) * overall_mean_count) + 1)

    return df_norm_logged

# ******************************* INPUT FILE PATHS ***************************
****
current_working_dir = os.getcwd()
base_dir = r'C:\Users\young\Documents\anh_validation'
# original counts table
asv_tbl_file_path = os.path.join(base_dir, 'asv_biom-with-taxonomy.txt')
# original metadata table
metadata_file_path = os.path.join(base_dir, 'mappingMetadata.txt')
```

Data Processing Steps

Step 1: Retrieve original counts table

In [2]:
```
df_asv = read_csv_file(asv_tbl_file_path, 1)
df_asv = df_asv.astype({col:'int32' for col in df_asv.columns[1:-1] }, copy=False)
df_asv
```

Out[2]:

	#OTU ID	1gKO.1	1gKO.2	1gKO.3	1gWT.1	1gWT.2	1gWT.3	2gKO.1
**0**	1ba8c796d07406783c96d016a6a5cace	13615	16637	17148	20227	23630	25656	14832
**1**	a6c38249aff7768283faf6cfbdeb05a8	26439	30129	19743	8955	10759	7074	18489
**2**	062f38ff92cfaee0654200b6f5be5ddf	7451	8774	8754	174	214	148	21958
**3**	1183cc23f552d81e63c93ca9fcba2f2c	225	223	184	13762	16856	18692	269
**4**	5e15ecfb579e72bf87c0bea3920bbf42	10108	12117	8633	10027	11910	7424	5979
**...**	...	...	...	...	...	...	...	...
**4070**	92bb8f4683ef5c8651e7d34dbb37ab2e	0	0	0	0	0	0	0
**4071**	92f09070a4fd5786bb34e756217e6ee1	0	0	0	0	0	0	0
**4072**	919b82324c41ed0046323c63aa1550da	0	0	0	0	0	0	0
**4073**	dbc0dad15ec1c8ad9d826cab94e18696	0	0	0	0	0	0	0
**4074**	1ff2d07d10264c23dc43e08d3097cd7c	0	0	0	0	0	0	0

4075 rows × 112 columns

Step 2: Retrieve original metadata table

In [3]:
```
df_metadata = read_csv_file(metadata_file_path)
df_metadata
```

Out[3]:

	SampleID	UniversalCageNumber	Background	FMTGroupFMTsourcegtRecipientbackground	Passage
**0**	F8-1	F8-cage-1	129.IL10KO	1gKOgtKO	8
**1**	F8-2	F8-cage-1	129.IL10KO	1gKOgtKO	8
**2**	F8-3	F8-cage-2	129.IL10KO	1gKOgtKO	8
**3**	F8-4	F8-cage-2	129.IL10KO	1gKOgtKO	8
**4**	F8-5	F8-cage-3	129.IL10KO	1gKOgtKO	8
**...**	...	...	...	...	...

**105**	1gWT.2	NaN	NaN		1gWTinput	1gWT
**106**	1gWT.3	NaN	NaN		1gWTinput	1gWT
**107**	2gWT.1	NaN	NaN		2gWTinput	2gWT
**108**	2gWT.2	NaN	NaN		2gWTinput	2gWT
**109**	2gWT.3	NaN	NaN		2gWTinput	2gWT

110 rows × 8 columns

Step 3: Create dictionary from metadata table

In [4]:
```
dict_metadata = get_dict_from_metadata(df_metadata)
dict_metadata.keys()
```

Out[4]:
```
dict_keys(['1gKOgtKO', '2gKOgtKO', '1gWTgtKO', '1gWTgtWT', '2gWTgtWT', 'hFM
T.1.2.3.gtKO', 'hFMT.3.4.5.gtKO', 'hFMT.1.2.3.gtWT', 'hFMT.1.2.3.input', 'hF
MT.3.4.5.input', '1gKOinput', '2gKOinput', '1gWTinput', '2gWTinput'])
```

Step 4: Calculate total read counts per sample

In [5]:
```
sample_count_sums = df_asv.iloc[:, 1:-1].sum(axis=0)
sample_count_sums
```

Out[5]:
```
1gKO.1       118256
1gKO.2       141891
1gKO.3       123292
1gWT.1       119717
1gWT.2       146158
              ...
h1-2-3.2     135326
h1-2-3.3     133745
h3-4-5.1     129613
h3-4-5.2     140316
h3-4-5.3     132984
Length: 110, dtype: int64
```

Step 5: Extract counts for target sample groups

In [6]:
```
# 'hFMT.1.2.3.input' -->  'HM1Input'
key_name ='hFMT.1.2.3.input'
print('Grouped columns: {}'.format(dict_metadata[key_name]))
HM1_Input = df_asv[[col_name for col_name in dict_metadata[key_name] ]]
HM1_Input
```

```
Grouped columns: ['h1-2-3.1', 'h1-2-3.2', 'h1-2-3.3']
```

Out[6]:

	h1-2-3.1	h1-2-3.2	h1-2-3.3
**0**	1483	1515	1402
**1**	1129	691	725
**2**	2927	3523	3254
**3**	298	231	238

3	[illegible]	[illegible]	[illegible]
4	218	160	111
...	...	...	...
4070	0	0	0
4071	0	0	0
4072	0	0	0
4073	0	0	0
4074	0	0	0

4075 rows × 3 columns

In [7]:

```
# 'hFMT.1.2.3.gtKO' -->  'HM1->KO'
key_name = 'hFMT.1.2.3.gtKO'
print('Grouped columns: {}'.format(dict_metadata[key_name]))
HM1_KO = df_asv[[col_name for col_name in dict_metadata[key_name] ]]
HM1_KO
```

```
Grouped columns: ['F3-7', 'F3-8', 'F3-9', 'F3-10', 'F3-11', 'F3-12', 'F1-7',
'F1-8', 'F1-9', 'F1-10', 'F1-11', 'F1-12', 'F1-13', 'F1-14', 'F1-15']
```

Out[7]:

	F3-7	F3-8	F3-9	F3-10	F3-11	F3-12	F1-7	F1-8	F1-9	F1-10	F1-11	F1-12	F1-13	F[illegible]
0	10097	574	12959	18737	17857	12650	10738	9576	7304	14409	14382	8787	7694	
1	35102	51633	31840	22887	20446	33188	23565	21707	12802	26719	5701	15715	25125	
2	1328	91	2203	189	182	244	33299	13739	53077	17570	34094	18922	19204	4[illegible]
3	137	315	305	134	221	290	203	348	363	238	219	205	255	
4	34960	13573	4109	3811	1155	28938	1144	6973	666	8230	1287	11346	13195	[illegible]
...	...	...	...	...	...	...	...	...	...	...	...	...	...	
4070	0	0	0	0	0	0	0	0	0	0	0	0	0	
4071	0	0	0	0	0	0	0	0	0	0	0	0	0	
4072	0	0	0	0	0	0	0	0	0	0	0	0	0	
4073	0	0	0	0	0	0	0	0	0	0	0	0	0	
4074	0	0	0	0	0	0	0	0	0	0	0	0	0	

4075 rows × 15 columns

In [8]:

```
# '1gKOinput' -->  'IMM-g1input'
key_name = '1gKOinput'
print('Grouped columns: {}'.format(dict_metadata[key_name]))
IMM_g1input = df_asv[[col_name for col_name in dict_metadata[key_name] ]]
IMM_g1input
```

```
Grouped columns: ['1gKO.1', '1gKO.2', '1gKO.3']
```

Out[8]:

	1gKO.1	1gKO.2	1gKO.3
0	13615	16637	17148
1	26439	30129	19743
2	7451	8774	8754
3	225	223	184
4	10108	12117	8633

**4**	[illegible]	[illegible]	[illegible]
**...**	...	...	...
**4070**	0	0	0
**4071**	0	0	0
**4072**	0	0	0
**4073**	0	0	0
**4074**	0	0	0

4075 rows × 3 columns

In [9]:

```
# '1gKOgtKO' -->  'IMM-g1->KO'
key_name = '1gKOgtKO'
print('Grouped columns: {}'.format(dict_metadata[key_name]))
IMM_g1_KO = df_asv[[col_name for col_name in dict_metadata[key_name] ]]
IMM_g1_KO
```

```
Grouped columns: ['F8-1', 'F8-2', 'F8-3', 'F8-4', 'F8-5', 'F8-6', 'F8-7', 'F
8-8', 'F4-1', 'F4-2', 'F4-3', 'F4-4', 'F4-5', 'F3-1', 'F3-2', 'F3-4', 'F3-
5', 'F3-6']
```

Out[9]:

	F8-1	F8-2	F8-3	F8-4	F8-5	F8-6	F8-7	F8-8	F4-1	F4-2	F4-3	F4-4	F4-5	
**0**	3339	15223	11423	18205	12486	10761	9339	466	29629	20134	27588	26741	25644	2
**1**	10283	6602	9314	13651	8789	16118	12045	13841	3687	6998	6904	7796	4011	
**2**	41532	40602	52337	11038	16781	8926	25758	31166	1627	37042	23825	26508	30376	
**3**	240	276	331	302	341	231	226	306	275	198	213	271	172	2
**4**	1806	6778	6725	12734	3901	10948	10347	1234	8891	3718	9188	4419	8315	
**...**	...	...	...	...	...	...	...	...	...	...	...	...	...	
**4070**	0	0	0	0	0	0	0	0	0	0	0	0	0	
**4071**	0	0	0	0	0	0	0	0	0	0	0	0	0	
**4072**	0	0	0	0	0	0	0	0	0	0	0	0	0	
**4073**	0	0	0	0	0	0	0	0	0	0	0	0	0	
**4074**	0	0	0	0	0	0	0	0	0	0	0	0	0	

4075 rows × 18 columns

In [10]:

```
# '2gKOinput' -->  'IMM-g2input'
key_name = '2gKOinput'
print('Grouped columns: {}'.format(dict_metadata[key_name]))
IMM_g2input = df_asv[[col_name for col_name in dict_metadata[key_name] ]]
IMM_g2input
```

```
Grouped columns: ['2gKO.1', '2gKO.2', '2gKO.3']
```

Out[10]:

	2gKO.1	2gKO.2	2gKO.3
**0**	14832	14973	13992
**1**	18489	18178	9122
**2**	21958	22435	17629
**3**	269	153	230
**4**	5979	4360	2762

4	[illegible]	[illegible]	[illegible]
...	...	...	...
4070	0	0	0
4071	0	0	0
4072	0	0	0
4073	0	0	0
4074	0	0	0

4075 rows × 3 columns

In [11]:

```
# '2gKOgtKO' -->  'IMM-g2->KO'
key_name = '2gKOgtKO'
print('Grouped columns: {}'.format(dict_metadata[key_name]))
IMM_g2_KO = df_asv[[col_name for col_name in dict_metadata[key_name] ]]
IMM_g2_KO
```

```
Grouped columns: ['F8-9', 'F8-10', 'F8-11', 'F8-12', 'F8-13', 'F8-14', 'F8-1
5', 'F8-16']
```

Out[11]:

	F8-9	F8-10	F8-11	F8-12	F8-13	F8-14	F8-15	F8-16
0	23451	24742	31312	13542	4593	10228	14477	23819
1	8030	14203	27872	26539	12663	13720	23316	14430
2	19653	22616	4603	28535	20096	20133	14650	24062
3	266	344	300	269	257	330	249	226
4	6490	6143	12353	2229	6367	11645	9607	10622
...	...	...	...	...	...	...	...	...
4070	0	0	0	0	0	0	0	0
4071	0	0	0	0	0	0	0	0
4072	0	0	0	0	0	0	0	0
4073	0	0	0	0	0	0	0	0
4074	0	0	0	0	0	0	0	0

4075 rows × 8 columns

Step 6: Normalize count values for target sample groups

In [12]:

```
HM1_Input_norm = _get_norm_counts(HM1_Input, sample_count_sums)
HM1_Input_norm
```

Out[12]:

	h1-2-3.1	h1-2-3.2	h1-2-3.3
0	3.170992	3.168281	3.139741
1	3.052636	2.827698	2.853624
2	3.466129	3.534613	3.505231
3	2.475230	2.353116	2.371106
4	2.340004	2.194478	2.041962

...	...	...	...
**4070**	0.000000	0.000000	0.000000
**4071**	0.000000	0.000000	0.000000
**4072**	0.000000	0.000000	0.000000
**4073**	0.000000	0.000000	0.000000
**4074**	0.000000	0.000000	0.000000

4075 rows × 3 columns

In [13]:
```
HM1_KO_norm = _get_norm_counts(HM1_KO, sample_count_sums)
HM1_KO_norm
```

Out[13]:

	F3-7	F3-8	F3-9	F3-10	F3-11	F3-12	F1-7	F1-8	F1-9	F1-
**0**	4.023995	2.715632	4.028643	4.300323	4.281648	4.108629	4.026117	3.977907	3.888433	4.1297
**1**	4.565105	4.668820	4.419021	4.387208	4.340445	4.527498	4.367439	4.333297	4.132126	4.3978
**2**	3.143272	1.920176	3.259285	2.306214	2.292111	2.395644	4.517597	4.134664	4.749730	4.2158
**3**	2.159501	2.455717	2.402055	2.157737	2.376040	2.470375	2.304807	2.539512	2.585851	2.3495
**4**	4.563345	4.088595	3.529896	3.608747	3.092750	4.467988	3.053962	3.840159	2.848906	3.8865
**...**	...	...	...	...	...	...	...	...	...	...
**4070**	0.000000	0.000000	0.000000	0.000000	0.000000	0.000000	0.000000	0.000000	0.000000	0.0000
**4071**	0.000000	0.000000	0.000000	0.000000	0.000000	0.000000	0.000000	0.000000	0.000000	0.0000
**4072**	0.000000	0.000000	0.000000	0.000000	0.000000	0.000000	0.000000	0.000000	0.000000	0.0000
**4073**	0.000000	0.000000	0.000000	0.000000	0.000000	0.000000	0.000000	0.000000	0.000000	0.0000
**4074**	0.000000	0.000000	0.000000	0.000000	0.000000	0.000000	0.000000	0.000000	0.000000	0.0000

4075 rows × 15 columns

In [14]:
```
IMM_g1input_norm = _get_norm_counts(IMM_g1input, sample_count_sums)
IMM_g1input_norm
```

Out[14]:

	1gKO.1	1gKO.2	1gKO.3
**0**	4.180178	4.188104	4.262257
**1**	4.468392	4.446001	4.323454
**2**	3.918399	3.910251	3.970273
**3**	2.400047	2.317401	2.295045
**4**	4.050836	4.050434	3.964229
**...**	...	...	...
**4070**	0.000000	0.000000	0.000000
**4071**	0.000000	0.000000	0.000000
**4072**	0.000000	0.000000	0.000000
**4073**	0.000000	0.000000	0.000000
**4074**	0.000000	0.000000	0.000000

4075 rows × 3 columns

In [15]:
```
IMM_g1_KO_norm = _get_norm_counts(IMM_g1_KO, sample_count_sums)
IMM_g1_KO_norm
```

Out[15]:

	F8-1	F8-2	F8-3	F8-4	F8-5	F8-6	F8-7	F8-8	F4-1	F4
**0**	3.530045	4.171099	4.067413	4.239122	4.320109	4.065412	3.978399	2.753708	4.509011	4.2994
**1**	4.018461	3.808313	3.978778	4.114104	4.167634	4.240858	4.088895	4.225749	3.604062	3.8405
**2**	4.624693	4.597128	4.728413	4.021838	4.448499	3.984224	4.418981	4.578248	3.248913	4.5642
**3**	2.388291	2.431092	2.530706	2.460420	2.757180	2.398871	2.364043	2.571443	2.478060	2.2944
**4**	3.263255	3.819737	3.837351	4.083907	3.814908	4.072893	4.022908	3.176160	3.986276	3.5659
**...**	...	...	...	...	...	...	...	...	...	
**4070**	0.000000	0.000000	0.000000	0.000000	0.000000	0.000000	0.000000	0.000000	0.000000	0.0000
**4071**	0.000000	0.000000	0.000000	0.000000	0.000000	0.000000	0.000000	0.000000	0.000000	0.0000
**4072**	0.000000	0.000000	0.000000	0.000000	0.000000	0.000000	0.000000	0.000000	0.000000	0.0000
**4073**	0.000000	0.000000	0.000000	0.000000	0.000000	0.000000	0.000000	0.000000	0.000000	0.0000
**4074**	0.000000	0.000000	0.000000	0.000000	0.000000	0.000000	0.000000	0.000000	0.000000	0.0000

4075 rows × 18 columns

In [16]:
```
IMM_g2input_norm = _get_norm_counts(IMM_g2input, sample_count_sums)
IMM_g2input_norm
```

Out[16]:

	2gKO.1	2gKO.2	2gKO.3
**0**	4.143317	4.154522	4.242762
**1**	4.239025	4.238754	4.056985
**2**	4.313700	4.330129	4.343104
**3**	2.403557	2.166842	2.460093
**4**	3.748792	3.618774	3.538206
**...**	...	...	...
**4070**	0.000000	0.000000	0.000000
**4071**	0.000000	0.000000	0.000000
**4072**	0.000000	0.000000	0.000000
**4073**	0.000000	0.000000	0.000000
**4074**	0.000000	0.000000	0.000000

4075 rows × 3 columns

In [17]:
```
IMM_g2_KO_norm = _get_norm_counts(IMM_g2_KO, sample_count_sums)
IMM_g2_KO_norm
```

Out[17]:

	F8-9	F8-10	F8-11	F8-12	F8-13	F8-14	F8-15	F8-16
**0**	4.480880	4.378895	4.379459	4.143025	3.698748	4.105404	4.195335	4.353703
**1**	4.015462	4.137854	4.328918	4.435212	4.139133	4.232959	4.402300	4.136058
**2**	4.404150	4.339877	3.546894	4.466704	4.339694	4.399505	4.200494	4.358111

**3**	2.536849	2.523304	2.362739	2.442634	2.448049	2.615148	2.432436	2.332891
**4**	3.923001	3.773895	3.975548	3.359611	3.840562	4.161749	4.017259	4.003009
**...**	...	...	...	...	...	...	...	...
**4070**	0.000000	0.000000	0.000000	0.000000	0.000000	0.000000	0.000000	0.000000
**4071**	0.000000	0.000000	0.000000	0.000000	0.000000	0.000000	0.000000	0.000000
**4072**	0.000000	0.000000	0.000000	0.000000	0.000000	0.000000	0.000000	0.000000
**4073**	0.000000	0.000000	0.000000	0.000000	0.000000	0.000000	0.000000	0.000000
**4074**	0.000000	0.000000	0.000000	0.000000	0.000000	0.000000	0.000000	0.000000

4075 rows × 8 columns

Step 7: Calculate Spearman Correlation Coefficients for each sample group

In [18]:
```
corr_value1 = get_inter_corr_values(HM1_Input_norm, HM1_KO_norm)
corr_value2 = get_inter_corr_values(IMM_g1input_norm, IMM_g1_KO_norm)
corr_value3 = get_inter_corr_values(IMM_g2input_norm, IMM_g2_KO_norm)
```

Step 8: Generate final figure

In [19]:
```
data = [corr_value1, corr_value2, corr_value3]
x_tick_labels = ['HM1Input\nvs HM1->KO',
                 'IMM-g1input\nvs IMM-g1->KO',
                 'IMM-g2input\nvs IMM-g2->KO']

fig, ax = plt.subplots()
ax.boxplot(data, widths=0.8)
ax.set_title('Regenerated Figure 4D')
ax.set_yticks([0.0, 0.2, 0.4, 0.6, 0.8, 1.0])
ax.set_xticklabels(x_tick_labels,
                   rotation=45, fontsize=10, weight='bold')

plt.show()
```

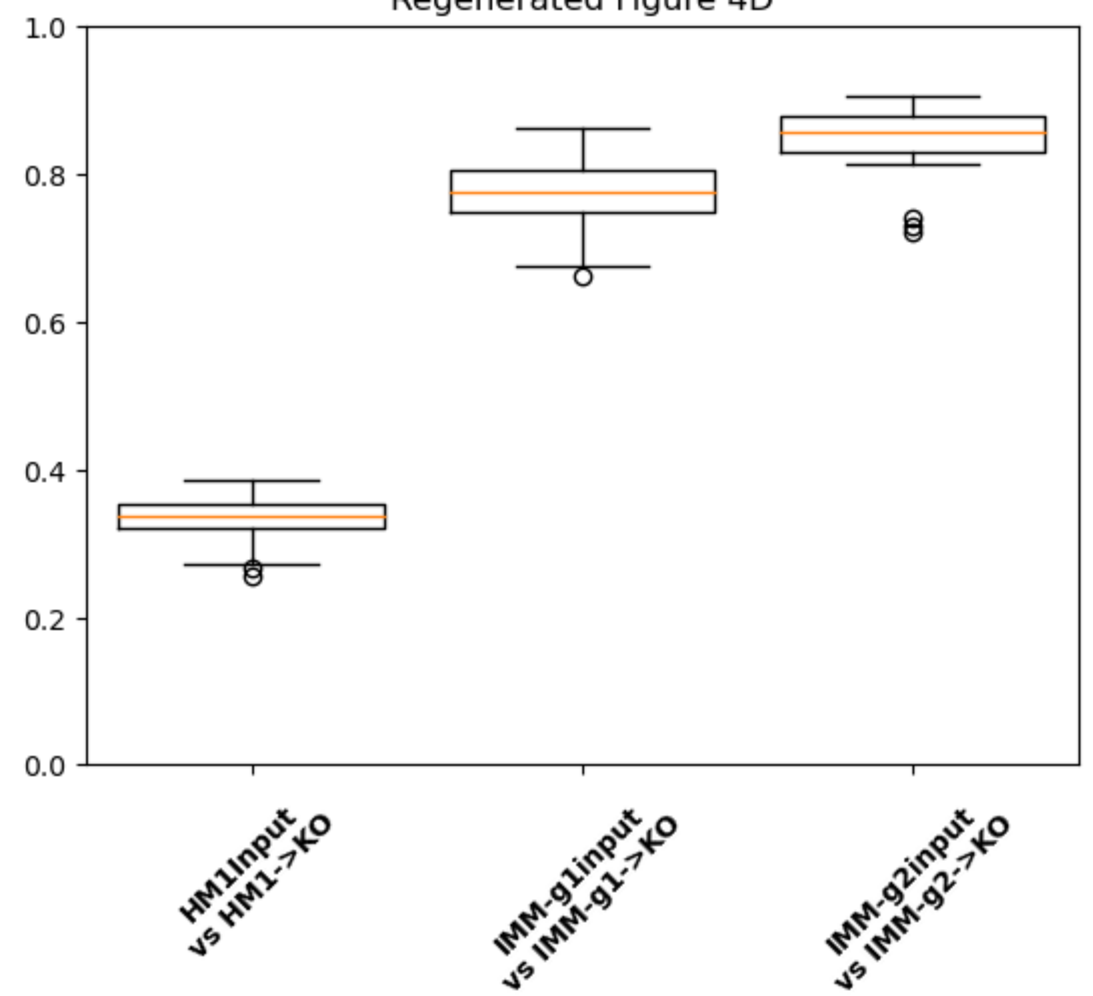

Step 9: Compare original and regenerated figures

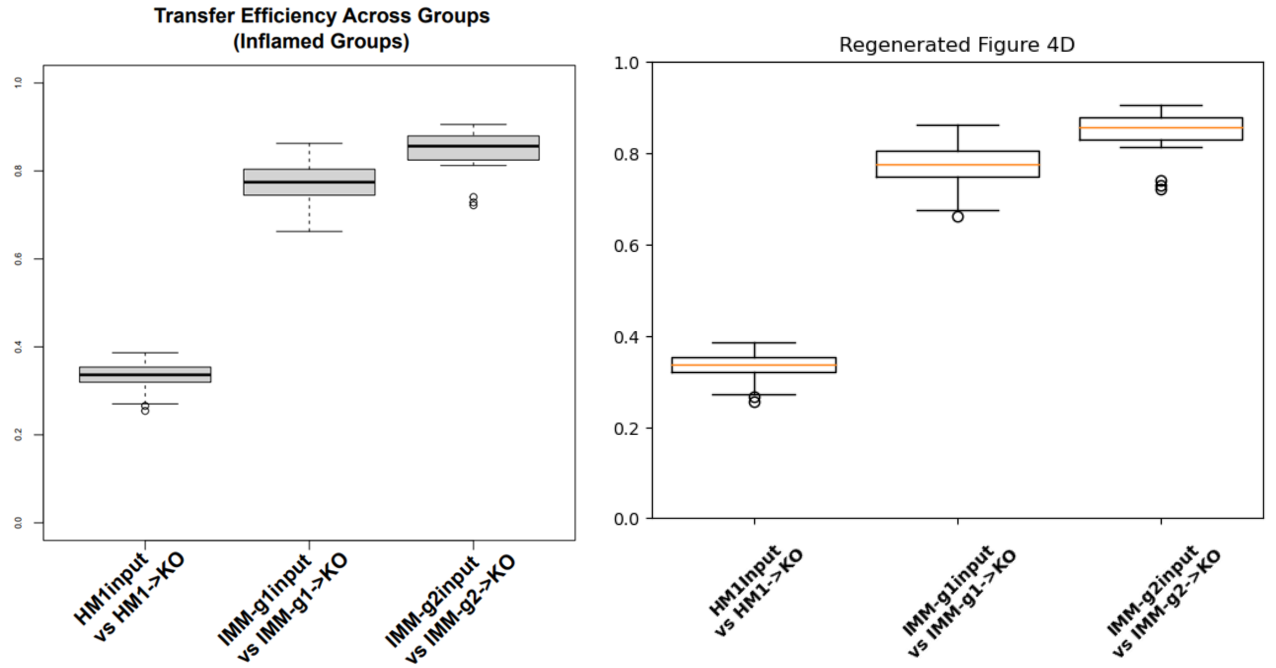

Contents

- **Aim**
- **Main Python Methods**
- **Data Processing Steps**
  - Step 1: Retrieve original counts table
  - Step 2: Retrieve original metadata table
  - Step 3: Create dictionary from metadata table
  - Step 4: Calculate total read counts per sample
  - Step 5: Extract counts for sample group HM1->KO
  - Step 6: Normalize count values for sample group HM1->KOO
  - Step 7: Calculate Spearman Correlation Coefficients for each sample pair
  - Step 8: Generate final figure
  - Step 9: Compare original and regenerated figures

Aim: Replicate Figure 5D

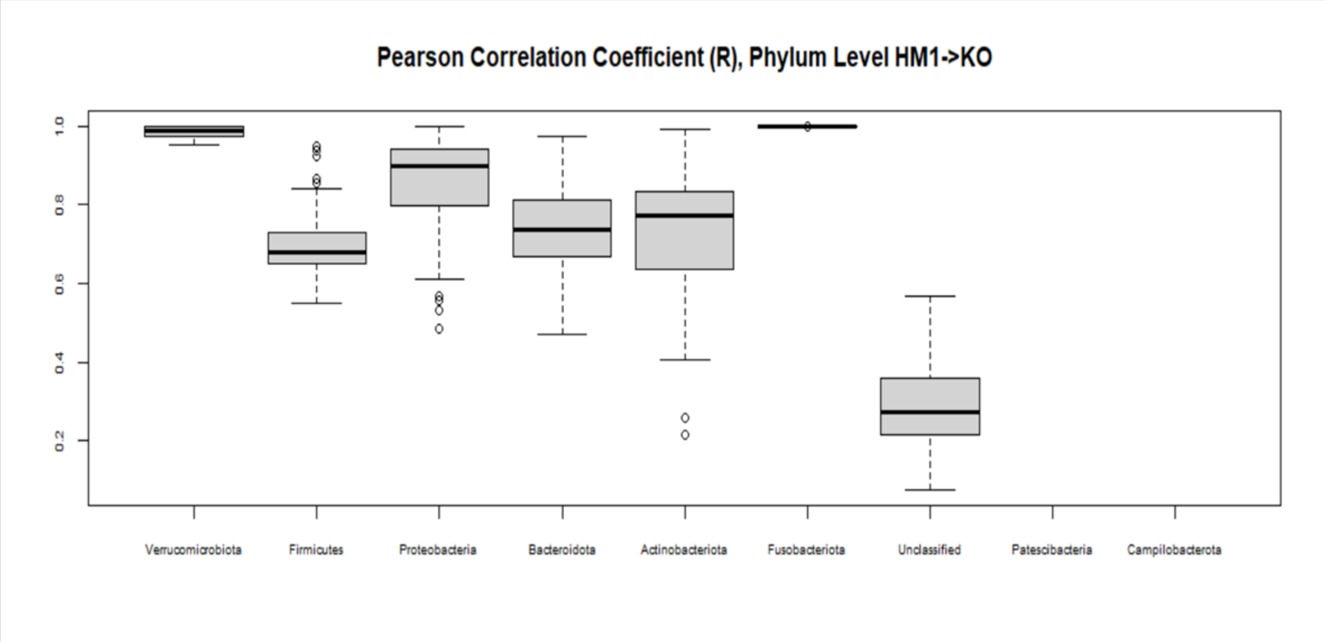

Main Python Methods

In [1]:
```
import numpy as np
import pandas as pd
import matplotlib.pyplot as plt
import os
import re
from scipy import stats
from scipy.special import comb

# ****************************** MAIN METHODS ******************************
# Filter rows for target taxa
def filter_rows(taxa_query, taxa_target):
    final_result = False
```

```
    search_result = re.search(taxa_target, taxa_query)
    if search_result:
        final_result = True

    return final_result
# Calculate Pearson correlation coefficients from input set of samples
def get_inter_corr_values(input_df):
    tup_list_column_names = [(col1, col2) for col1 in input_df.columns for col
2 in input_df.columns if input_df.columns.get_loc(col2) > input_df.columns.get
_loc(col1)]
    corr_values = [stats.pearsonr(input_df.loc[:, tup[0]], input_df.loc[:, tup
[1]]).statistic \
            for tup in tup_list_column_names]
    # Remove nan values
    corr_values = [val for val in corr_values if not np.isnan(val)]
    return corr_values

# Retrieve tab delimited file
def read_csv_file(file_path, skiprows=None, header = 0, sep = '\t', index_col=
False):
    df = pd.read_csv(file_path, skiprows=skiprows, sep=sep, header=header, ind
ex_col=index_col)

    return df

# Create dictionary from metadata table
def get_dict_from_metadata(input_df):
    mydict = {}
    for row in input_df.iterrows():
        obj = row[1]
        sample_id = obj['SampleID']
        key = obj['FMTGroupFMTsourcegtRecipientbackground']
        if key not in mydict:
            mydict[key] = [sample_id]
        else:
            mydict[key].append(sample_id)
    return mydict

# ******************************* HELPER METHODS ******************************
**
# Normalize sample count values
def _get_norm_counts(input_df, ser_sample_count_sums):
    overall_mean_count = ser_sample_count_sums.mean()
    df_norm_logged =    pd.DataFrame()
    for col_name in input_df.columns:
        df_norm_logged.loc[:, col_name] = \
        np.log10(((input_df.loc[:, col_name] / ser_sample_count_sums[col_name
]) * overall_mean_count) + 1)

    return df_norm_logged

# ******************************* INPUT FILE PATHS ***************************
****
# Get current working directory
current_working_dir = os.getcwd()
# original counts table
asv_tbl_file_path = os.path.join(current_working_dir, 'asv_biom-with-taxonomy.
txt')
# original metadata table
metadata_file_path = os.path.join(current_working_dir, 'mappingMetadata.txt')
```

Data Processing Steps

Step 1: Retrieve original counts table

In [2]:
```
df_asv = read_csv_file(asv_tbl_file_path, 1)
df_asv
```

Out[2]:

	#OTU ID	1gKO.1	1gKO.2	1gKO.3	1gWT.1	1gWT.2	1gWT.3	2gKO.1
**0**	1ba8c796d07406783c96d016a6a5cace	13615.0	16637.0	17148.0	20227.0	23630.0	25656.0	14832.0
**1**	a6c38249aff7768283faf6cfbdeb05a8	26439.0	30129.0	19743.0	8955.0	10759.0	7074.0	18489.0
**2**	062f38ff92cfaee0654200b6f5be5ddf	7451.0	8774.0	8754.0	174.0	214.0	148.0	21958.0
**3**	1183cc23f552d81e63c93ca9fcba2f2c	225.0	223.0	184.0	13762.0	16856.0	18692.0	269.0
**4**	5e15ecfb579e72bf87c0bea3920bbf42	10108.0	12117.0	8633.0	10027.0	11910.0	7424.0	5979.0
**...**	...	...	...	...	...	...	...	...
**4070**	92bb8f4683ef5c8651e7d34dbb37ab2e	0.0	0.0	0.0	0.0	0.0	0.0	0.0
**4071**	92f09070a4fd5786bb34e756217e6ee1	0.0	0.0	0.0	0.0	0.0	0.0	0.0
**4072**	919b82324c41ed0046323c63aa1550da	0.0	0.0	0.0	0.0	0.0	0.0	0.0
**4073**	dbc0dad15ec1c8ad9d826cab94e18696	0.0	0.0	0.0	0.0	0.0	0.0	0.0
**4074**	1ff2d07d10264c23dc43e08d3097cd7c	0.0	0.0	0.0	0.0	0.0	0.0	0.0

4075 rows × 112 columns

In [3]:
```
df_asv = read_csv_file(asv_tbl_file_path, 1)
df_asv = df_asv.astype({col:'int32' for col in df_asv.columns[1:-1] }, copy=Fa
lse)
df_asv
```

Out[3]:

	#OTU ID	1gKO.1	1gKO.2	1gKO.3	1gWT.1	1gWT.2	1gWT.3	2gKO.1
**0**	1ba8c796d07406783c96d016a6a5cace	13615	16637	17148	20227	23630	25656	14832
**1**	a6c38249aff7768283faf6cfbdeb05a8	26439	30129	19743	8955	10759	7074	18489
**2**	062f38ff92cfaee0654200b6f5be5ddf	7451	8774	8754	174	214	148	21958

**3**	1183cc23f552d81e63c93ca9fcba2f2c	225	223	184	13762	16856	18692	269
**4**	5e15ecfb579e72bf87c0bea3920bbf42	10108	12117	8633	10027	11910	7424	5979
**...**	...	...	...	...	...	...	...	...
**4070**	92bb8f4683ef5c8651e7d34dbb37ab2e	0	0	0	0	0	0	0
**4071**	92f09070a4fd5786bb34e756217e6ee1	0	0	0	0	0	0	0
**4072**	919b82324c41ed0046323c63aa1550da	0	0	0	0	0	0	0
**4073**	dbc0dad15ec1c8ad9d826cab94e18696	0	0	0	0	0	0	0
**4074**	1ff2d07d10264c23dc43e08d3097cd7c	0	0	0	0	0	0	0

4075 rows × 112 columns

Step 2: Retrieve original metadata table

In [4]:

```
df_metadata = read_csv_file(metadata_file_path)
df_metadata
```

Out[4]:

	SampleID	UniversalCageNumber	Background	FMTGroupFMTsourcegtRecipientbackground	Passage
**0**	F8-1	F8-cage-1	129.IL10KO	1gKOgtKO	8
**1**	F8-2	F8-cage-1	129.IL10KO	1gKOgtKO	8
**2**	F8-3	F8-cage-2	129.IL10KO	1gKOgtKO	8
**3**	F8-4	F8-cage-2	129.IL10KO	1gKOgtKO	8
**4**	F8-5	F8-cage-3	129.IL10KO	1gKOgtKO	8
**...**	...	...	...	...	...
**105**	1gWT.2	NaN	NaN	1gWTinput	1gWT
**106**	1gWT.3	NaN	NaN	1gWTinput	1gWT
**107**	2gWT.1	NaN	NaN	2gWTinput	2gWT
**108**	2gWT.2	NaN	NaN	2gWTinput	2gWT
**109**	2gWT.3	NaN	NaN	2gWTinput	2gWT

110 rows × 8 columns

Step 3: Create dictionary from metadata table

In [5]:

```
dict_metadata = get_dict_from_metadata(df_metadata)
dict_metadata.keys()
```

Out[5]:

```
dict_keys(['1gKOgtKO', '2gKOgtKO', '1gWTgtKO', '1gWTgtWT', '2gWTgtWT', 'hFM
T.1.2.3.gtKO', 'hFMT.3.4.5.gtKO', 'hFMT.1.2.3.gtWT', 'hFMT.1.2.3.input', 'hF
MT.3.4.5.input', '1gKOinput', '2gKOinput', '1gWTinput', '2gWTinput'])
```

Step 4: Calculate total read counts per sample

In [6]:
```
sample_count_sums = df_asv.iloc[:, 1:-1].sum(axis=0)
sample_count_sums
```

Out[6]:
```
1gKO.1       118256
1gKO.2       141891
1gKO.3       123292
1gWT.1       119717
1gWT.2       146158
              ...
h1-2-3.2     135326
h1-2-3.3     133745
h3-4-5.1     129613
h3-4-5.2     140316
h3-4-5.3     132984
Length: 110, dtype: int64
```

Step 5: Extract counts for sample group HM1->KO

In [7]:
```
# 'hFMT.1.2.3.gtKO' -->  'HM1->KO'
key_name = 'hFMT.1.2.3.gtKO'
group_columns = dict_metadata[key_name]
group_columns.extend(['#OTU ID', 'taxonomy'])
key_name = 'hFMT.1.2.3.gtKO'
HM1_KO = df_asv[[col_name for col_name in group_columns ]]
HM1_KO
```

Out[7]:

	F3-7	F3-8	F3-9	F3-10	F3-11	F3-12	F1-7	F1-8	F1-9	F1-10	F1-11	F1-12	F1-13	F
**0**	10097	574	12959	18737	17857	12650	10738	9576	7304	14409	14382	8787	7694	
**1**	35102	51633	31840	22887	20446	33188	23565	21707	12802	26719	5701	15715	25125	
**2**	1328	91	2203	189	182	244	33299	13739	53077	17570	34094	18922	19204	4
**3**	137	315	305	134	221	290	203	348	363	238	219	205	255	
**4**	34960	13573	4109	3811	1155	28938	1144	6973	666	8230	1287	11346	13195	[illegible]
**...**	...	...	...	...	...	...	...	...	...	...	...	...	...	
**4070**	0	0	0	0	0	0	0	0	0	0	0	0	0	
**4071**	0	0	0	0	0	0	0	0	0	0	0	0	0	
**4072**	0	0	0	0	0	0	0	0	0	0	0	0	0	
**4073**	0	0	0	0	0	0	0	0	0	0	0	0	0	
**4074**	0	0	0	0	0	0	0	0	0	0	0	0	0	

4075 rows × 17 columns

In [8]:
```
target_phylum = 'p__Verrucomicrobiota'
df_Verrucomicrobiota = HM1_KO[HM1_KO.taxonomy.apply(lambda x: filter_rows(x, t
arget_phylum))]
print('Number of rows: {}'.format(len(df_Verrucomicrobiota.index)))
print('Columns: {}'.format(', '.join(df_Verrucomicrobiota.columns)))
```

```
Number of rows: 24
Columns: F3-7, F3-8, F3-9, F3-10, F3-11, F3-12, F1-7, F1-8, F1-9, F1-10, F1-
11, F1-12, F1-13, F1-14, F1-15, #OTU ID, taxonomy
```

In [9]:
```
target_phylum = 'p__Firmicutes'
df_Firmicutes = HM1_KO[HM1_KO.taxonomy.apply(lambda x: filter_rows(x, target_p
hylum))]
print('Number of rows: {}'.format(len(df_Firmicutes.index)))
print('Columns: {}'.format(', '.join(df_Firmicutes.columns)))
```

```
Number of rows: 830
Columns: F3-7, F3-8, F3-9, F3-10, F3-11, F3-12, F1-7, F1-8, F1-9, F1-10, F1-
11, F1-12, F1-13, F1-14, F1-15, #OTU ID, taxonomy
```

In [10]:
```
target_phylum = 'p__Proteobacteria'
df_Proteobacteria = HM1_KO[HM1_KO.taxonomy.apply(lambda x: filter_rows(x, targ
et_phylum))]
print('Number of rows: {}'.format(len(df_Proteobacteria.index)))
print('Columns: {}'.format(', '.join(df_Proteobacteria.columns)))
```

```
Number of rows: 51
Columns: F3-7, F3-8, F3-9, F3-10, F3-11, F3-12, F1-7, F1-8, F1-9, F1-10, F1-
11, F1-12, F1-13, F1-14, F1-15, #OTU ID, taxonomy
```

In [11]:
```
target_phylum = 'p__Bacteroidota'
df_Bacteroidota = HM1_KO[HM1_KO.taxonomy.apply(lambda x: filter_rows(x, target
_phylum))]
print('Number of rows: {}'.format(len(df_Bacteroidota.index)))
print('Columns: {}'.format(', '.join(df_Bacteroidota.columns)))
```

```
Number of rows: 91
Columns: F3-7, F3-8, F3-9, F3-10, F3-11, F3-12, F1-7, F1-8, F1-9, F1-10, F1-
11, F1-12, F1-13, F1-14, F1-15, #OTU ID, taxonomy
```

In [12]:
```
target_phylum = 'p__Actinobacteriota'
df_Actinobacteriota = HM1_KO[HM1_KO.taxonomy.apply(lambda x: filter_rows(x, ta
rget_phylum))]
print('Number of rows: {}'.format(len(df_Actinobacteriota.index)))
print('Columns: {}'.format(', '.join(df_Actinobacteriota.columns)))
```

```
Number of rows: 71
Columns: F3-7, F3-8, F3-9, F3-10, F3-11, F3-12, F1-7, F1-8, F1-9, F1-10, F1-
11, F1-12, F1-13, F1-14, F1-15, #OTU ID, taxonomy
```

In [13]:
```
target_phylum = 'p__Fusobacteriota'
df_Fusobacteriota = HM1_KO[HM1_KO.taxonomy.apply(lambda x: filter_rows(x, targ
et_phylum))]
print('Number of rows: {}'.format(len(df_Fusobacteriota.index)))
print('Columns: {}'.format(', '.join(df_Fusobacteriota.columns)))
```

```
Number of rows: 11
Columns: F3-7, F3-8, F3-9, F3-10, F3-11, F3-12, F1-7, F1-8, F1-9, F1-10, F1-
11, F1-12, F1-13, F1-14, F1-15, #OTU ID, taxonomy
```

In [14]:
```
target_phylum = 'p__Patescibacteria'
df_Patescibacteria = HM1_KO[HM1_KO.taxonomy.apply(lambda x: filter_rows(x, tar
get_phylum))]
print('Number of rows: {}'.format(len(df_Patescibacteria.index)))
print('Columns: {}'.format(', '.join(df_Patescibacteria.columns)))
```

```
Number of rows: 3
Columns: F3-7, F3-8, F3-9, F3-10, F3-11, F3-12, F1-7, F1-8, F1-9, F1-10, F1-
11, F1-12, F1-13, F1-14, F1-15, #OTU ID, taxonomy
```

In [15]:
```
target_phylum = 'p__Campilobacterota'
df_Campilobacterota = HM1_KO[HM1_KO.taxonomy.apply(lambda x: filter_rows(x, ta
rget_phylum))]
print('Number of rows: {}'.format(len(df_Campilobacterota.index)))
print('Columns: {}'.format(', '.join(df_Campilobacterota.columns)))
```

```
Number of rows: 3
Columns: F3-7, F3-8, F3-9, F3-10, F3-11, F3-12, F1-7, F1-8, F1-9, F1-10, F1-
11, F1-12, F1-13, F1-14, F1-15, #OTU ID, taxonomy
```

In [16]:
```
target_phylum = 'Unassigned'
df_Unassigned = HM1_KO[HM1_KO.taxonomy.apply(lambda x: filter_rows(x, target_p
hylum))]
print('Number of rows: {:,}'.format(len(df_Unassigned.index)))
print('Columns: {}'.format(', '.join(df_Unassigned.columns)))
```

```
Number of rows: 2,196
Columns: F3-7, F3-8, F3-9, F3-10, F3-11, F3-12, F1-7, F1-8, F1-9, F1-10, F1-
11, F1-12, F1-13, F1-14, F1-15, #OTU ID, taxonomy
```

Step 6: Normalize count values for sample group HM1->KO

In [17]:
```
df_Verrucomicrobiota_norm = _get_norm_counts(df_Verrucomicrobiota.iloc[:, :-2
], sample_count_sums)
print('Number of rows: {}'.format(len(df_Verrucomicrobiota_norm.index)))
print('Columns: {}'.format(', '.join(df_Verrucomicrobiota_norm.columns)))
```

```
Number of rows: 24
Columns: F3-7, F3-8, F3-9, F3-10, F3-11, F3-12, F1-7, F1-8, F1-9, F1-10, F1-
11, F1-12, F1-13, F1-14, F1-15
```

In [18]:
```
df_Firmicutes_norm = _get_norm_counts(df_Firmicutes.iloc[:, :-2], sample_count
_sums)
print('Number of rows: {}'.format(len(df_Firmicutes_norm.index)))
print('Columns: {}'.format(', '.join(df_Firmicutes_norm.columns)))
```

```
Number of rows: 830
Columns: F3-7, F3-8, F3-9, F3-10, F3-11, F3-12, F1-7, F1-8, F1-9, F1-10, F1-
11, F1-12, F1-13, F1-14, F1-15
```

In [19]:
```
df_Proteobacteria_norm = _get_norm_counts(df_Proteobacteria.iloc[:, :-2], samp
le_count_sums)
```

```
print('Number of rows: {}'.format(len(df_Proteobacteria_norm.index)))
print('Columns: {}'.format(', '.join(df_Proteobacteria_norm.columns)))
```

```
Number of rows: 51
Columns: F3-7, F3-8, F3-9, F3-10, F3-11, F3-12, F1-7, F1-8, F1-9, F1-10, F1-
11, F1-12, F1-13, F1-14, F1-15
```

In [20]:
```
df_Bacteroidota_norm = _get_norm_counts(df_Bacteroidota.iloc[:, :-2], sample_c
ount_sums)
print('Number of rows: {}'.format(len(df_Bacteroidota_norm.index)))
print('Columns: {}'.format(', '.join(df_Bacteroidota_norm.columns)))
```

```
Number of rows: 91
Columns: F3-7, F3-8, F3-9, F3-10, F3-11, F3-12, F1-7, F1-8, F1-9, F1-10, F1-
11, F1-12, F1-13, F1-14, F1-15
```

In [21]:
```
df_Actinobacteriota_norm = _get_norm_counts(df_Actinobacteriota.iloc[:, :-2],
sample_count_sums)
print('Number of rows: {}'.format(len(df_Actinobacteriota_norm.index)))
print('Columns: {}'.format(', '.join(df_Actinobacteriota_norm.columns)))
```

```
Number of rows: 71
Columns: F3-7, F3-8, F3-9, F3-10, F3-11, F3-12, F1-7, F1-8, F1-9, F1-10, F1-
11, F1-12, F1-13, F1-14, F1-15
```

In [22]:
```
df_Fusobacteriota_norm = _get_norm_counts(df_Fusobacteriota.iloc[:, :-2], samp
le_count_sums)
print('Number of rows: {}'.format(len(df_Fusobacteriota_norm.index)))
print('Columns: {}'.format(', '.join(df_Fusobacteriota_norm.columns)))
```

```
Number of rows: 11
Columns: F3-7, F3-8, F3-9, F3-10, F3-11, F3-12, F1-7, F1-8, F1-9, F1-10, F1-
11, F1-12, F1-13, F1-14, F1-15
```

In [23]:
```
df_Unassigned_norm = _get_norm_counts(df_Unassigned.iloc[:, :-2], sample_count
_sums)
print('Number of rows: {:,}'.format(len(df_Unassigned_norm.index)))
print('Columns: {}'.format(', '.join(df_Unassigned_norm.columns)))
```

```
Number of rows: 2,196
Columns: F3-7, F3-8, F3-9, F3-10, F3-11, F3-12, F1-7, F1-8, F1-9, F1-10, F1-
11, F1-12, F1-13, F1-14, F1-15
```

In [24]:
```
df_Patescibacteria_norm = _get_norm_counts(df_Patescibacteria.iloc[:, :-2], sa
mple_count_sums)
print('Number of rows: {:,}'.format(len(df_Patescibacteria_norm.index)))
print('Columns: {}'.format(', '.join(df_Patescibacteria_norm.columns)))
```

```
Number of rows: 3
Columns: F3-7, F3-8, F3-9, F3-10, F3-11, F3-12, F1-7, F1-8, F1-9, F1-10, F1-
11, F1-12, F1-13, F1-14, F1-15
```

In [25]:
```
df_Campilobacterota_norm = _get_norm_counts(df_Campilobacterota.iloc[:, :-2],
sample_count_sums)
print('Number of rows: {:,}'.format(len(df_Campilobacterota_norm.index)))
print('Columns: {}'.format(', '.join(df_Campilobacterota_norm.columns)))
```

```
Number of rows: 3

Columns: F3-7, F3-8, F3-9, F3-10, F3-11, F3-12, F1-7, F1-8, F1-9, F1-10, F1-
11, F1-12, F1-13, F1-14, F1-15
```

Step 7: Calculate Spearman Correlation Coefficients for each sample pair

In [26]:
```
corr_Verrucomicrobiota_norm = get_inter_corr_values(df_Verrucomicrobiota_norm)
norm_Firmicutes_norm = get_inter_corr_values(df_Firmicutes_norm)
corr_Proteobacteria_norm = get_inter_corr_values(df_Proteobacteria_norm)
corr_Bacteroidota_norm = get_inter_corr_values(df_Bacteroidota_norm)
corr_Actinobacteriota_norm = get_inter_corr_values(df_Actinobacteriota_norm)
corr_Fusobacteriota_norm = get_inter_corr_values(df_Fusobacteriota_norm)
corr_Unassigned_norm = get_inter_corr_values(df_Unassigned_norm)
corr_Patescibacteria_norm = get_inter_corr_values(df_Patescibacteria_norm)
corr_Campilobacterota_norm = get_inter_corr_values(df_Campilobacterota_norm)
```

```
C:\ProgramData\Anaconda3\lib\site-packages\scipy\stats\_stats_py.py:4424: Co
nstantInputWarning: An input array is constant; the correlation coefficient
is not defined.
  warnings.warn(stats.ConstantInputWarning(msg))
```

Step 8: Generate final figure

In [27]:
```
data = [corr_Verrucomicrobiota_norm,
            norm_Firmicutes_norm,
            corr_Proteobacteria_norm,
            corr_Bacteroidota_norm,
            corr_Actinobacteriota_norm,
            corr_Fusobacteriota_norm,
            corr_Unassigned_norm,
            corr_Patescibacteria_norm,
            corr_Campilobacterota_norm]

x_tick_labels = ['Verrucomicrobiota',
                 'Firmicutes',
                 'Proteobacteria',
                 'Bacteroidota',
                 'Actinobacteriota',
                 'Fusobacteriota',
                 'Unassigned',
                 'Patescibacteria',
                 'Campilobacterota']
fig, ax = plt.subplots(figsize=(9, 3))
ax.boxplot(data, widths=0.8)
ax.set_title('Regenerated Figure 5D')
ax.set_yticks([0.2, 0.4, 0.6, 0.8, 1.0])
ax.set_xticklabels(x_tick_labels,
                    rotation=0, fontsize=6)
plt.show()
```

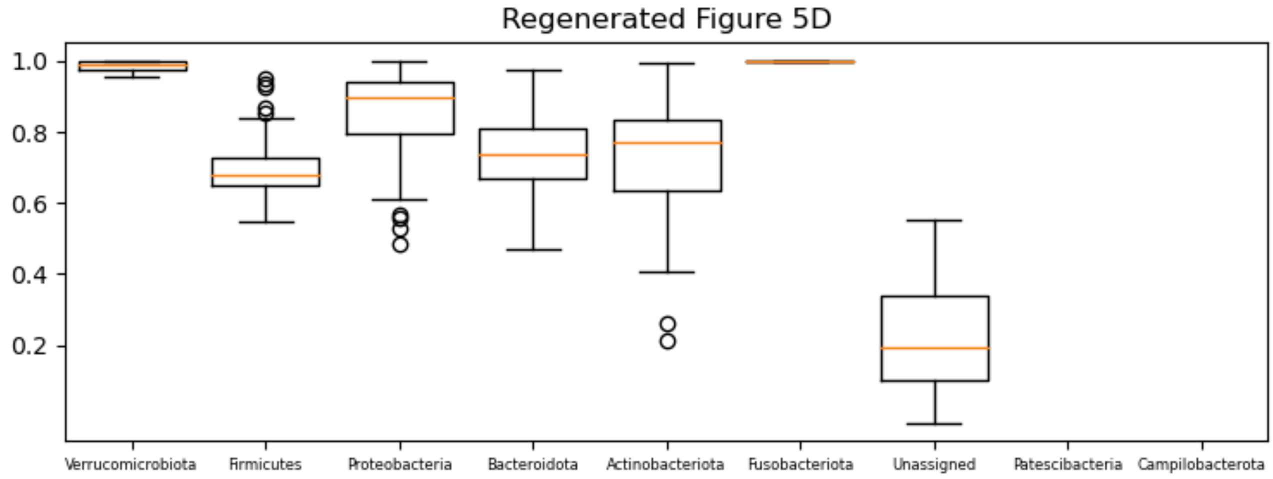

Step 9: Compare original and regenerated figures

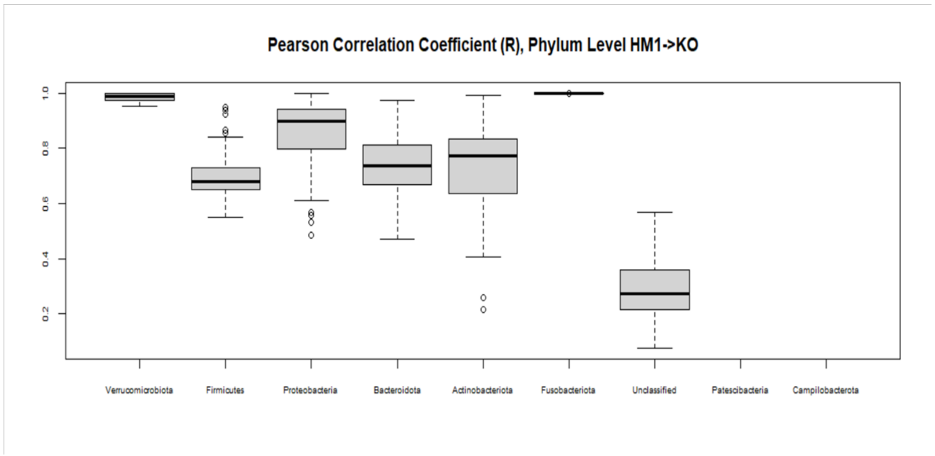

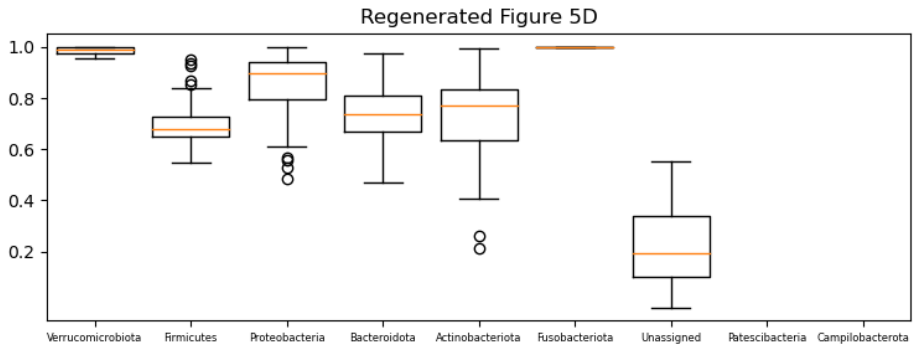

Figure Validation Summary

Figure 1D Comparision (source code at figure_validation_1D.pdf)

1D

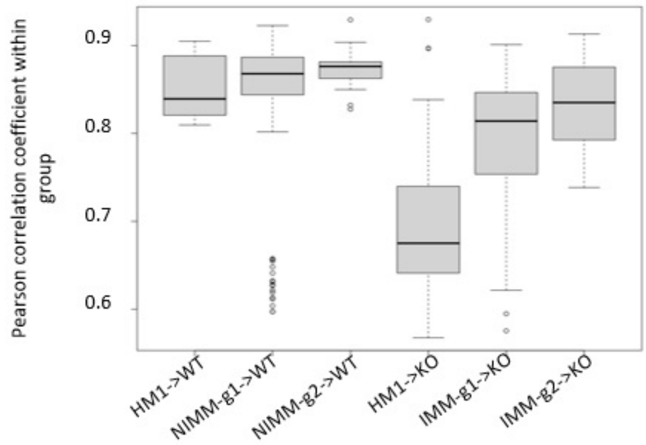

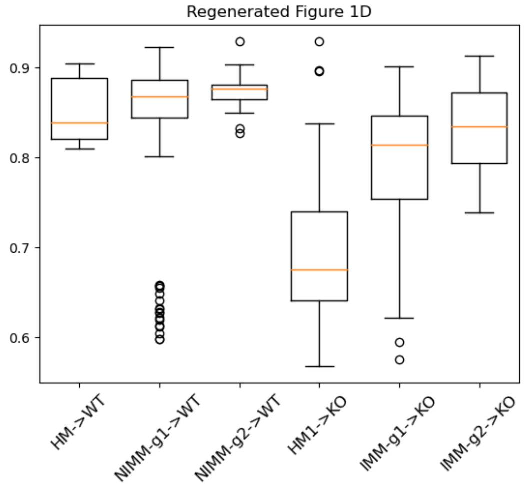

Figure 3A Comparision (source code at figure_validation_3A.pdf)

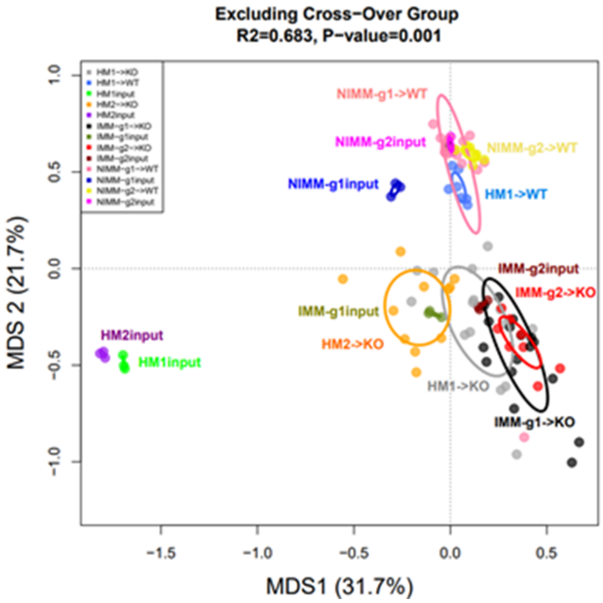

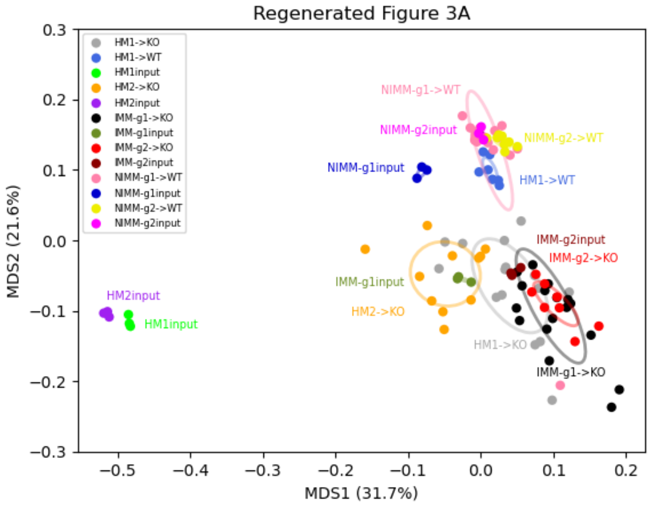

Figure 4B Comparision (source code at figure_validation_4B.pdf)

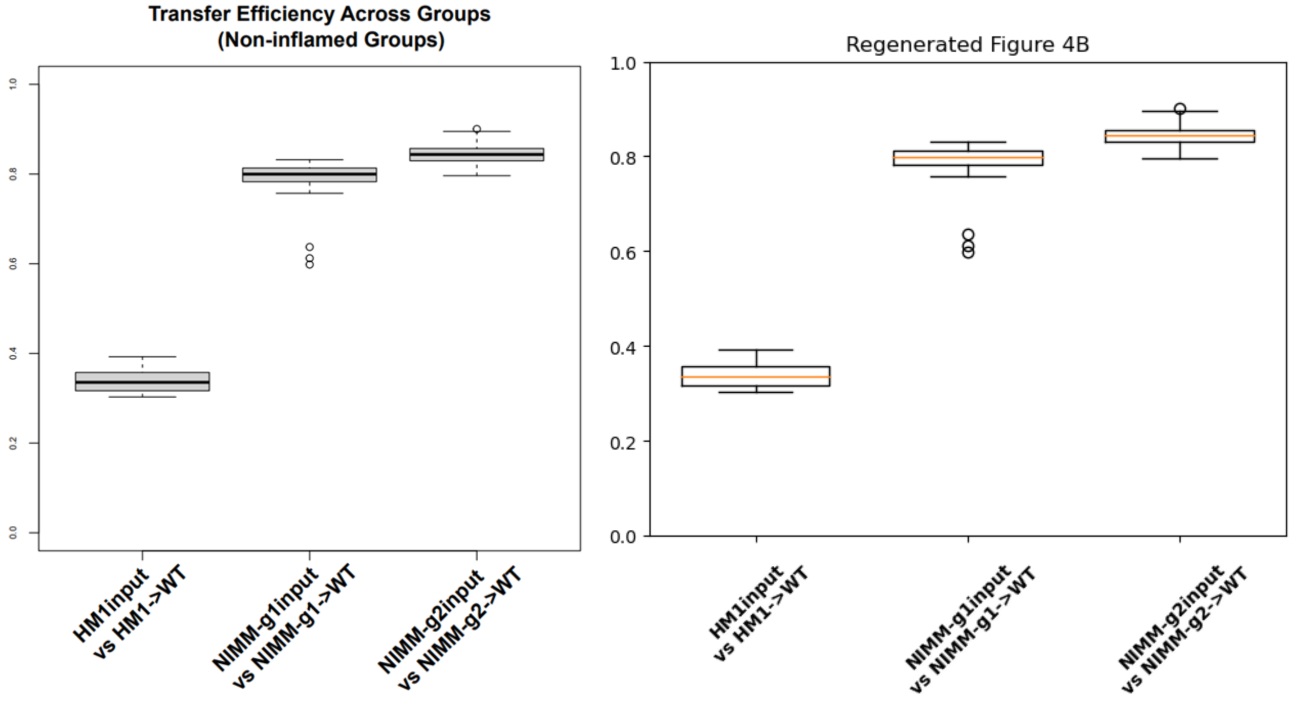

Figure 4D Comparision (source code at figure_validation_4D.pdf)

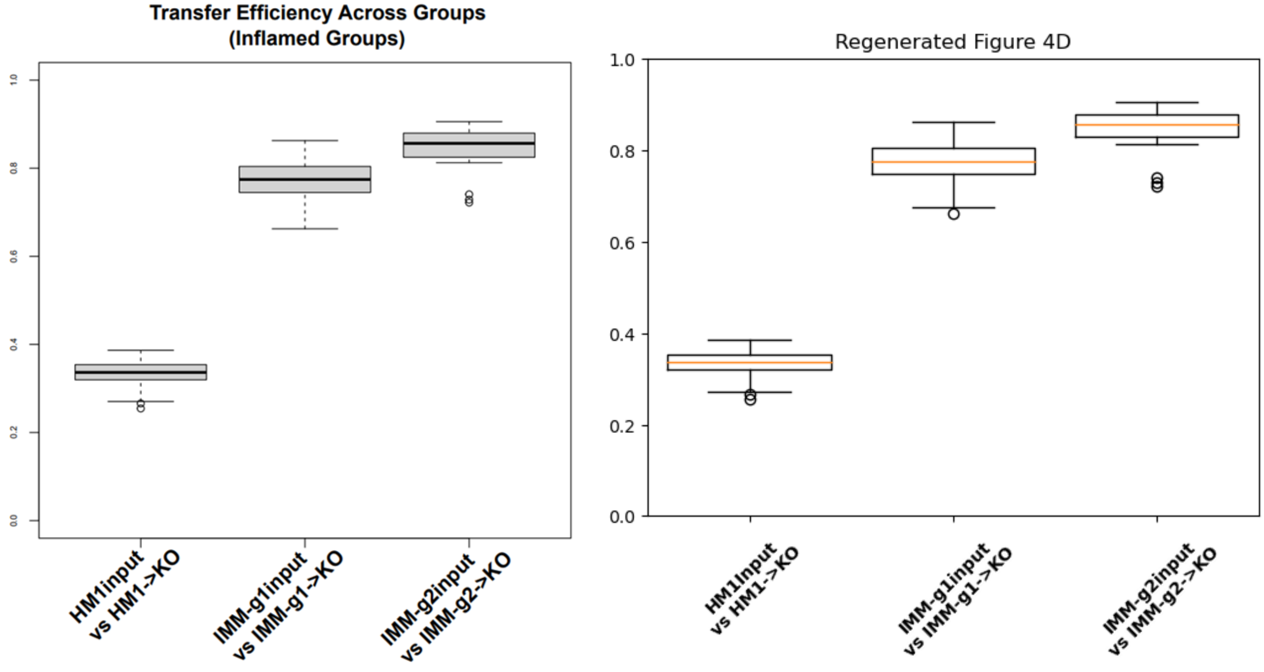

Figure 5D Comparision (source code at figure_validation_5D.pdf)

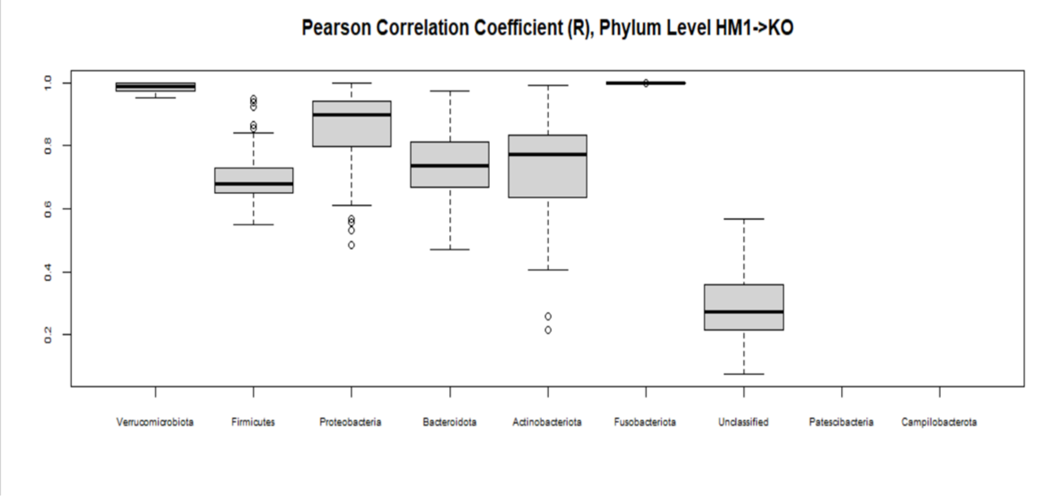
Pearson Correlation Coefficient (R), Phylum Level HM1->KO
1.0
0.8
0.6
0.4
0.2
Verrucomicrobiota
Firmicutes
Proteobacteria
Bacteroidota
Actinobacteriota
Fusobacteriota
Unclassified
Patescibacteria
Campilobacterota

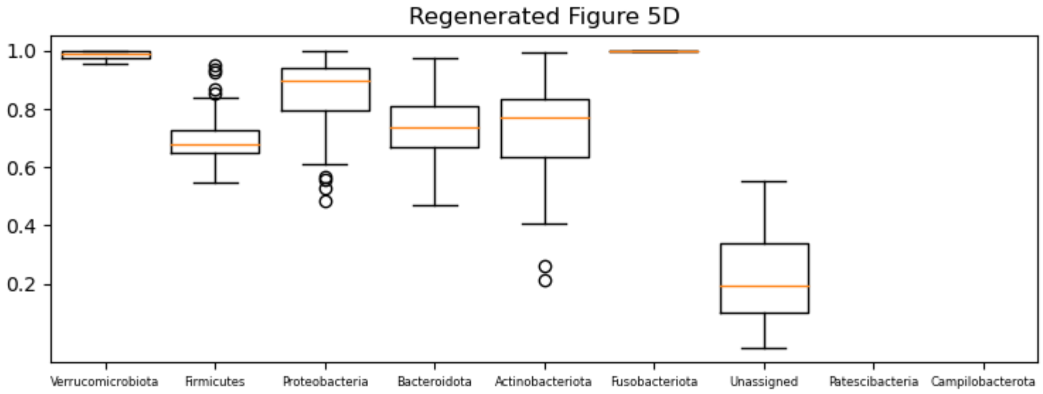
Regenerated Figure 5D
1.0
0.8
0.6
0.4
0.2
Verrucomicrobiota
Firmicutes
Proteobacteria
Bacteroidota
Actinobacteriota
Fusobacteriota
Unassigned
Patescibacteria
Campilobacterota

Contents

- **Aim**
- **Main Python Methods**
- **Data Processing Steps**
  - Step 1: Retrieve source feature counts table
  - Step 2: Filter feature counts table with target sample columns
  - Step 3: Calculate total read counts per sample
  - Step 4: Generate normalized counts feature table
  - Step 5: Generate Bray-Curtis dissimilarity distance matrix
  - Step 6: Perform principal coordinate analysis (PCoA)
  - Step 7: Plot PCoA results
  - Step 8: Compare original and regenerated figures

Aim: Replicate figure

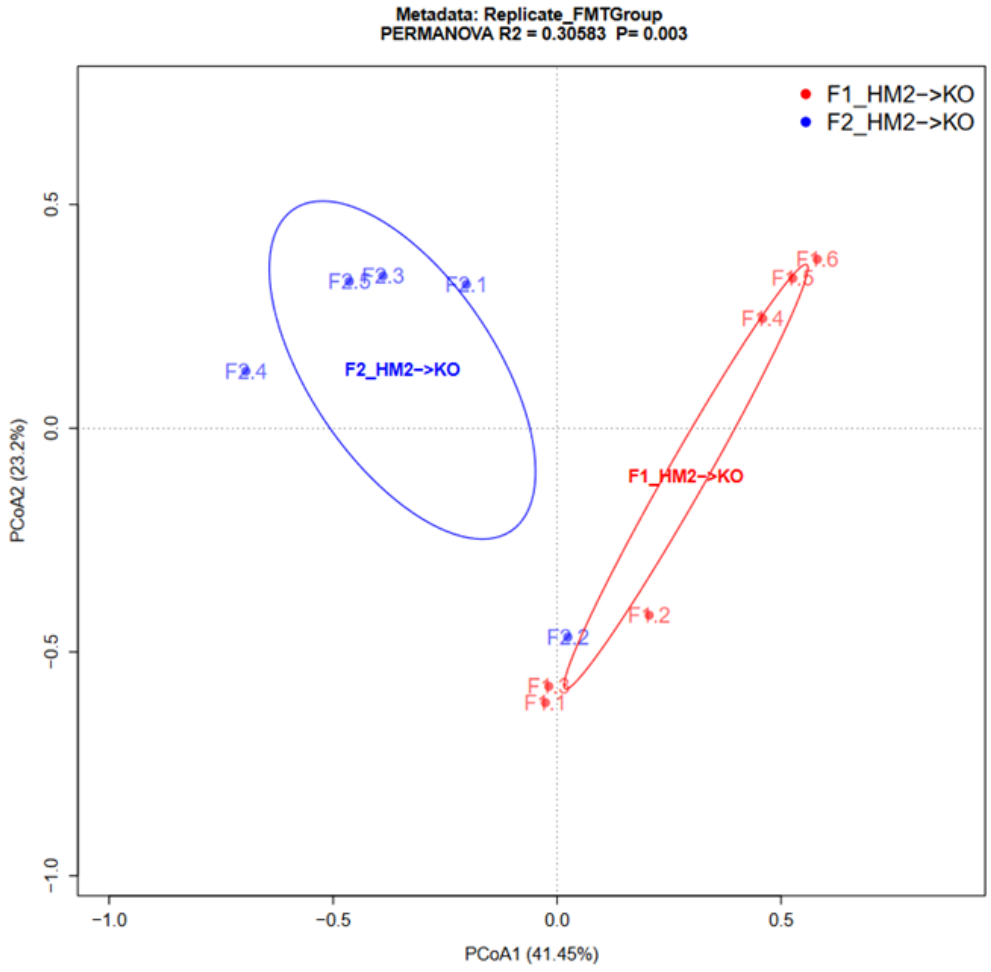

Main Python Methods

In [1]:
```
import numpy as np
import pandas as pd
import matplotlib.pyplot as plt
```

```
import os
import skbio
from scipy import stats
from scipy.spatial.distance import pdist, squareform
from matplotlib.patches import Ellipse
import matplotlib.transforms as transforms

# ******************************* MAIN METHODS ******************************
# Retrieve tab delimited file
def read_csv_file(file_path, skiprows=None, header = 0, sep = '\t', index_col=
False):
    df = pd.read_csv(file_path, skiprows=skiprows, sep=sep, header=header, ind
ex_col=index_col)

    return df

# Reference:  Function "get_cov_ellipse" was extracted from the following URL:

# https://scipython.com/book/chapter-7-matplotlib/examples/bmi-data-with-confi
dence-ellipses/
# (Learning Scientific Programming with Python by Christian Hill)
def get_cov_ellipse(cov, centre, nstd, **kwargs):
    """
    Return a matplotlib Ellipse patch representing the covariance matrix
    cov centred at centre and scaled by the factor nstd.
    """
    # Find and sort eigenvalues and eigenvectors into descending order
    eigvals, eigvecs = np.linalg.eigh(cov)
    order = eigvals.argsort()[::-1]
    eigvals, eigvecs = eigvals[order], eigvecs[:, order]

    # The anti-clockwise angle to rotate our ellipse by
    vx, vy = eigvecs[:,0][0], eigvecs[:,0][1]
    theta = np.arctan2(vy, vx)

    # Width and height of ellipse to draw
    width, height = 2 * nstd * np.sqrt(eigvals)
    return Ellipse(xy=centre, width=width, height=height,
                   angle=np.degrees(theta), fill=False, **kwargs)

# ******************************* INPUT FILE PATHS ***************************
****
# Get current working directory
current_working_dir = os.getcwd()
# original counts table file path
feature_tbl_file_path = os.path.join(current_working_dir, 'feature-table_balfo
ur.txt')
# dictionary for two sample groupings
dict_metadata = {'129.IL10KO_1':['F1-1', 'F1-2', 'F1-3', 'F1-4', 'F1-5', 'F1-
6'],
                 '129.IL10KO_2':['F2-1' ,'F2-2', 'F2-3', 'F2-4', 'F2-5']}
# cross reference for sample grouping names
cross_ref_dict = {'129.IL10KO_1':'F1_HM2->KO', '129.IL10KO_2':'F2_HM2->KO'}
# cross reference for sample names
sample_cross_ref_dict = {'F1-1':'F1.1', 'F1-2':'F1.2', 'F1-3':'F1.3', 'F1-4':
'F1.4', 'F1-5':'F1.5', 'F1-6':'F1.6',
                         'F2-1':'F2.1','F2-2':'F2.2', 'F2-3':'F2.3', 'F2-4':'F
2.4', 'F2-5':'F2.5'}
```

Data Processing Steps

Step 1: Retrieve source feature counts table

In [2]:
```
df_feature_counts = read_csv_file(feature_tbl_file_path, 1)
df_feature_counts = df_feature_counts.astype({col:'int32' for col in df_featur
e_counts.columns[1:]})
df_feature_counts
```

Out[2]:

	#OTU ID	1gKO.1	1gKO.2	1gKO.3	1gWT.1	1gWT.2	1gWT.3
**0**	d__Bacteria;p__Verrucomicrobiota;c__Verrucomic...	11370	14120	14648	16632	19817	21706
**1**	d__Bacteria;p__Firmicutes;c__Clostridia;o__Lac...	22545	25836	17048	7547	9272	5996
**2**	d__Bacteria;p__Proteobacteria;c__Gammaproteoba...	5919	6481	6714	31	31	30
**3**	d__Bacteria;p__Firmicutes;c__Clostridia;o__Lac...	8134	9827	7090	8264	10100	6324
**4**	d__Bacteria;p__Firmicutes;c__Clostridia;o__Lac...	6280	7649	5719	9715	11851	8047
**...**	...	...	...	...	...	...	...
**191**	d__Bacteria;p__Firmicutes;c__Clostridia;o__Pep...	0	0	0	0	0	0
**192**	d__Bacteria;p__Firmicutes;c__Clostridia;o__Clo...	0	0	0	0	0	0
**193**	d__Bacteria;p__Firmicutes;c__Clostridia;o__Chr...	0	0	0	0	0	0
**194**	d__Bacteria;p__Firmicutes;c__Incertae_Sedis;o_...	0	0	0	0	0	0
**195**	d__Bacteria;p__Firmicutes;c__Bacilli;o__Lactob...	0	0	0	0	0	0

196 rows × 111 columns

Step 2: Filter feature counts table with target sample columns

In [3]:
```
target_samples = [ sample for sample_group in list(dict_metadata.values()) for
 sample in sample_group ]
df_feature_counts_filtered = df_feature_counts[target_samples]
df_feature_counts_filtered
```

Out[3]:

	F1-1	F1-2	F1-3	F1-4	F1-5	F1-6	F2-1	F2-2	F2-3	F2-4	F2-5
**0**	10401	2753	7914	2398	6595	2313	8957	10961	344	460	63
**1**	7133	7051	3523	26054	524	2105	10749	5732	14621	319	9372
**2**	2132	5867	4116	54	87	284	2194	1425	3296	1071	13246
**3**	1965	394	779	828	929	1973	4084	39	2827	17547	2891
**4**	4785	1982	3119	2274	41521	49604	3306	3350	3278	2247	1094
**...**	...	...	...	...	...	...	...	...	...	...	...
**191**	0	0	0	0	0	0	0	0	0	0	0
**192**	0	0	0	0	0	0	0	0	0	0	0
**193**	0	0	0	0	0	0	0	0	0	0	0
**194**	0	0	0	0	0	0	0	0	0	0	0

195	0	0	0	0	0	0	0	0	0	0	0

196 rows × 11 columns

Step 3: Calculate total read counts per sample

In [4]:
```
sample_count_sums = df_feature_counts_filtered.sum(axis=0)
sample_count_sums
```

Out[4]:
```
F1-1     120620
F1-2      89533
F1-3     103243
F1-4      92643
F1-5     120934
F1-6      98057
F2-1      98214
F2-2      90013
F2-3      95932
F2-4     111659
F2-5      87600
dtype: int64
```

Step 4: Generate normalized counts feature table

In [5]:
```
df_feature_counts_filtered_norm = np.log10((df_feature_counts_filtered/sample_
count_sums)*sample_count_sums.mean() + 1)
df_feature_counts_filtered_norm
```

Out[5]:

	F1-1	F1-2	F1-3	F1-4	F1-5	F1-6	F2-1	F2-2	F2-3	F2-
0	3.939028	3.491286	3.887914	3.416526	3.740068	3.376202	3.963359	4.088903	2.559118	2.61923
1	3.775248	3.899645	3.536501	4.452400	2.641099	3.335297	4.042557	3.807391	4.186364	2.46072
2	3.250934	3.819821	3.604045	1.776236	1.866243	2.466648	3.352579	3.203104	3.539472	2.98566
3	3.215530	2.647814	2.881570	2.955022	2.889351	3.307185	3.622339	1.649917	3.472831	4.19965
4	3.601894	3.348638	3.483620	3.393476	4.539055	4.707369	3.530580	3.574178	3.537094	3.30724
...	...	...	...	...	...	...	...	...	...	.
191	0.000000	0.000000	0.000000	0.000000	0.000000	0.000000	0.000000	0.000000	0.000000	0.00000
192	0.000000	0.000000	0.000000	0.000000	0.000000	0.000000	0.000000	0.000000	0.000000	0.00000
193	0.000000	0.000000	0.000000	0.000000	0.000000	0.000000	0.000000	0.000000	0.000000	0.00000
194	0.000000	0.000000	0.000000	0.000000	0.000000	0.000000	0.000000	0.000000	0.000000	0.00000
195	0.000000	0.000000	0.000000	0.000000	0.000000	0.000000	0.000000	0.000000	0.000000	0.00000

196 rows × 11 columns

Step 5: Generate Bray-Curtis dissimilarity distance matrix

In [6]:
```
condensed_arr = pdist(df_feature_counts_filtered_norm.T, metric='braycurtis')
```

```
dist_mat_sym = squareform(condensed_arr)
print('Dimensions of symmetrical Bray-Curtris distance matrix: {}'.format(dist
_mat_sym.shape))
dist_mat_sym
```

```
Dimensions of symmetrical Bray-Curtris distance matrix: (11, 11)
```

Out[6]:
```
array([[0.        , 0.17543969, 0.1210743 , 0.24413798, 0.26614686,
        0.27932716, 0.24563653, 0.16897739, 0.25286129, 0.25787212,
        0.26180328],
       [0.17543969, 0.        , 0.12735125, 0.21321068, 0.23873452,
        0.23562064, 0.27308808, 0.22960201, 0.24593477, 0.32563064,
        0.25044773],
       [0.1210743 , 0.12735125, 0.        , 0.25242907, 0.24876063,
        0.27042458, 0.25521475, 0.17566823, 0.23123946, 0.2648364 ,
        0.23475823],
       [0.24413798, 0.21321068, 0.25242907, 0.        , 0.15078165,
        0.16505002, 0.24546878, 0.23891727, 0.2609215 , 0.34959776,
        0.27547553],
       [0.26614686, 0.23873452, 0.24876063, 0.15078165, 0.        ,
        0.12648274, 0.24629414, 0.26384811, 0.27647145, 0.34676066,
        0.31165956],
       [0.27932716, 0.23562064, 0.27042458, 0.16505002, 0.12648274,
        0.        , 0.29074424, 0.29024396, 0.29012887, 0.36578334,
        0.30479539],
       [0.24563653, 0.27308808, 0.25521475, 0.24546878, 0.24629414,
        0.29074424, 0.        , 0.20846872, 0.17002751, 0.26274988,
        0.1835378 ],
       [0.16897739, 0.22960201, 0.17566823, 0.23891727, 0.26384811,
        0.29024396, 0.20846872, 0.        , 0.24951014, 0.30229098,
        0.27901923],
       [0.25286129, 0.24593477, 0.23123946, 0.2609215 , 0.27647145,
        0.29012887, 0.17002751, 0.24951014, 0.        , 0.18683878,
        0.11773076],
       [0.25787212, 0.32563064, 0.2648364 , 0.34959776, 0.34676066,
        0.36578334, 0.26274988, 0.30229098, 0.18683878, 0.        ,
        0.1907071 ],
       [0.26180328, 0.25044773, 0.23475823, 0.27547553, 0.31165956,
        0.30479539, 0.1835378 , 0.27901923, 0.11773076, 0.1907071 ,
        0.        ]])
```

Step 6: Perform principal coordinate analysis (PCoA)

In [7]:
```
import warnings
warnings.filterwarnings('ignore')
my_pcoa = skbio.stats.ordination.pcoa(dist_mat_sym)
df_pcoa = my_pcoa.samples[['PC1', 'PC2']]
# Normalize PC1 and PC2 into unit vectors
df_pcoa = pd.DataFrame(df_pcoa.to_numpy()/np.linalg.norm(df_pcoa.to_numpy(), a
xis=0))
print('PCoA proportion explained:')
my_pcoa.proportion_explained
```

```
PCoA proportion explained:
```

Out[7]:
```
PC1      0.415128
PC2      0.231760
PC3      0.129317
PC4      0.093920
```

```
PC5      0.044440
PC6      0.033396
PC7      0.028290
PC8      0.017374
PC9      0.006376
PC10     0.000000
PC11     0.000000
dtype: float64
```

Step 7: Plot PCoA results

In [8]:

```
colors = ['red', 'blue']
text_tup = [(-0.09, 0.00),
            (-0.1, -0.02)]

fig, ax = plt.subplots()
ax.set_xlim((-1.0, 0.70))
ax.set_ylim((-1.0, 0.70))
ax.set_yticks([-1.0, -0.5, 0.0, 0.5])
ax.set_xticks([-1.0, -0.5, 0.0, 0.5])
ax.set_xlabel('PCoA1 ({}%)'.format(np.round(my_pcoa.proportion_explained.PC1 *
 100, 2)))
ax.set_ylabel('PCoA2 ({}%)'.format(np.round(my_pcoa.proportion_explained.PC2 *
 100, 2)))
ax.set_title('Regenerated Figure')

end = 0
for idx, group_name in enumerate(dict_metadata):
    sample_names = dict_metadata[group_name]
    group_name = cross_ref_dict[group_name]
    group_count = len(sample_names)
    start = end
    end = start + group_count

    # multiply by -1 to rotate vector 180 degrees in order to match target fig
ure
    pc1 = df_pcoa.iloc[start:end, 0] * -1.0
    pc2 = df_pcoa.iloc[start:end, 1]

    # plot points
    ax.scatter(pc1, pc2, s=15, c=colors[idx],  label=group_name, alpha=0.5, ed
gecolors='none')
    cov = np.cov(pc1,pc2)
    x_mean = pc1.mean()
    y_mean = pc2.mean()
    e = get_cov_ellipse(cov, (x_mean, y_mean), 1,
                        ec=colors[idx], linewidth=2.0, alpha=0.4)
    ax.text(x_mean + text_tup[idx][0], y_mean + text_tup[idx][1], group_name,
            fontsize='x-small', c=colors[idx])
    ax.add_artist(e)

    for i, sample_name in enumerate(sample_names):
        ax.text(pc1.iloc[i], pc2.iloc[i], sample_cross_ref_dict[sample_name],
                fontsize='x-small', c=colors[idx], horizontalalignment='cente
r',
               verticalalignment='center')
ax.legend(fontsize=6, loc='upper right')
plt.show()
```

Regenerated Figure

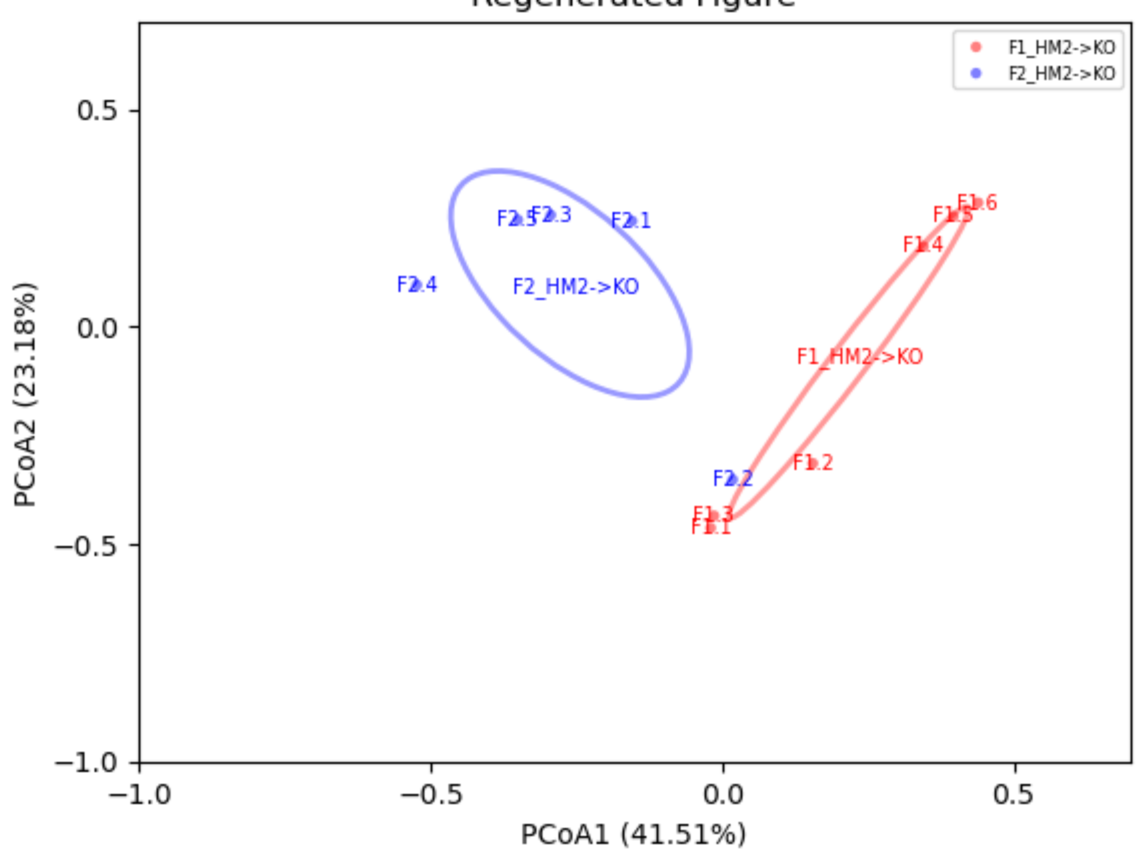

Step 8: Compare original and regenerated figures

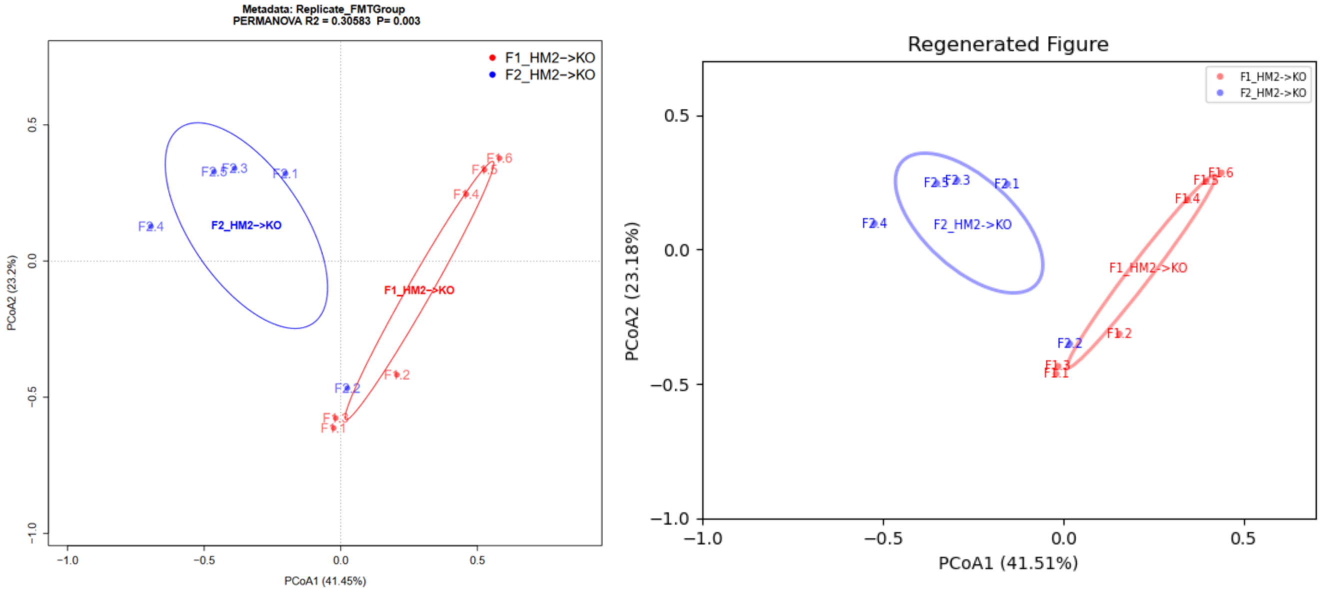

Contents

- **Aim**
- **Main Python Methods**
- **Data Processing Steps**
  - Step 1: Retrieve source feature counts table
  - Step 2: Filter feature counts table with target sample columns
  - Step 3: Calculate total read counts per sample
  - Step 4: Generate normalized counts feature table
  - Step 5: Generate Bray-Curtis dissimilarity distance matrix
  - Step 6: Perform principal coordinate analysis (PCoA)
  - Step 7: Plot PCoA results
  - Step 8: Compare original and regenerated figures

Aim: Replicate figure

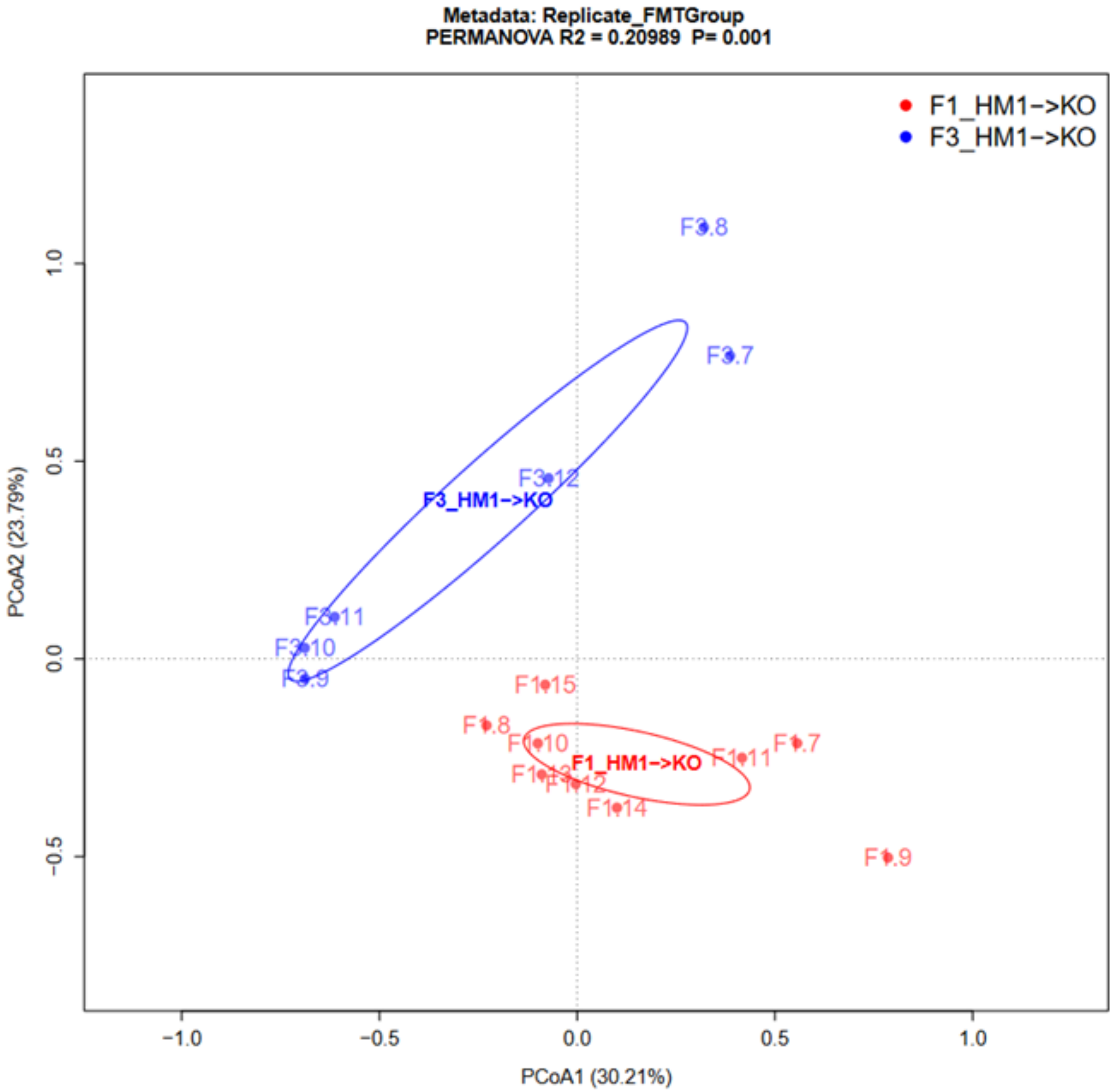

Main Python Methods

In [1]:
```
import numpy as np
import pandas as pd
import matplotlib.pyplot as plt
import os
import skbio
from scipy import stats
from scipy.spatial.distance import pdist, squareform
from matplotlib.patches import Ellipse
import matplotlib.transforms as transforms

# ******************************** MAIN METHODS ********************************
# Retrieve tab delimited file
def read_csv_file(file_path, skiprows=None, header = 0, sep = '\t', index_col=
False):
    df = pd.read_csv(file_path, skiprows=skiprows, sep=sep, header=header, ind
ex_col=index_col)

    return df

# Reference:  Function "get_cov_ellipse" was extracted from the following URL:

# https://scipython.com/book/chapter-7-matplotlib/examples/bmi-data-with-confi
dence-ellipses/
# (Learning Scientific Programming with Python by Christian Hill)
def get_cov_ellipse(cov, centre, nstd, **kwargs):
    """
    Return a matplotlib Ellipse patch representing the covariance matrix
    cov centred at centre and scaled by the factor nstd.
    """
    # Find and sort eigenvalues and eigenvectors into descending order
    eigvals, eigvecs = np.linalg.eigh(cov)
    order = eigvals.argsort()[::-1]
    eigvals, eigvecs = eigvals[order], eigvecs[:, order]

    # The anti-clockwise angle to rotate our ellipse by
    vx, vy = eigvecs[:,0][0], eigvecs[:,0][1]
    theta = np.arctan2(vy, vx)

    # Width and height of ellipse to draw
    width, height = 2 * nstd * np.sqrt(eigvals)
    return Ellipse(xy=centre, width=width, height=height,
                   angle=np.degrees(theta), fill=False, **kwargs)

# ******************************** INPUT FILE PATHS ****************************
****
# Get current working directory
current_working_dir = os.getcwd()
# original counts table file path
feature_tbl_file_path = os.path.join(current_working_dir, 'feature-table_balfo
ur.txt')
# dictionary for two sample groupings
dict_metadata = {'hFMT.1.2.3.gtKO_1':['F1-7', 'F1-8', 'F1-9', 'F1-10', 'F1-11'
, 'F1-12', 'F1-13', 'F1-14', 'F1-15'],
                 'hFMT.1.2.3.gtKO_2':['F3-7', 'F3-8', 'F3-9', 'F3-10', 'F3-11'
, 'F3-12']}
# cross reference for sample grouping names
cross_ref_dict = {'hFMT.1.2.3.gtKO_1':'F1_HM1->KO', 'hFMT.1.2.3.gtKO_2':'F3_HM
2->KO'}
# criss referebce for sample names
```

```
sample_cross_ref_dict = {'F1-7':'F1.7', 'F1-8':'F1.8', 'F1-9':'F1.9', 'F1-10':
'F1.10',
                         'F1-11':'F1.11', 'F1-12':'F1.12', 'F1-13':'F1.13','F1
-14':'F1.14', 'F1-15':'F1.15',
                        'F3-7':'F3.7', 'F3-8':'F3.8', 'F3-9':'F3.9', 'F3-10':
'F3.10', 'F3-11':'F3.11', 'F3-12':'F3.12'}
```

Data Processing Steps

Step 1: Retrieve source feature counts table

In [2]:
```
df_feature_counts = read_csv_file(feature_tbl_file_path, 1)
df_feature_counts = df_feature_counts.astype({col:'int32' for col in df_featur
e_counts.columns[1:]})
df_feature_counts
```

Out[2]:

	#OTU ID	1gKO.1	1gKO.2	1gKO.3	1gWT.1	1gWT.2	1gWT.[illegible]
**0**	d__Bacteria;p__Verrucomicrobiota;c__Verrucomic...	11370	14120	14648	16632	19817	2170[illegible]
**1**	d__Bacteria;p__Firmicutes;c__Clostridia;o__Lac...	22545	25836	17048	7547	9272	599[illegible]
**2**	d__Bacteria;p__Proteobacteria;c__Gammaproteoba...	5919	6481	6714	31	31	3[illegible]
**3**	d__Bacteria;p__Firmicutes;c__Clostridia;o__Lac...	8134	9827	7090	8264	10100	632[illegible]
**4**	d__Bacteria;p__Firmicutes;c__Clostridia;o__Lac...	6280	7649	5719	9715	11851	804[illegible]
**...**	...	...	...	...	...	...	..
**191**	d__Bacteria;p__Firmicutes;c__Clostridia;o__Pep...	0	0	0	0	0	[illegible]
**192**	d__Bacteria;p__Firmicutes;c__Clostridia;o__Clo...	0	0	0	0	0	[illegible]
**193**	d__Bacteria;p__Firmicutes;c__Clostridia;o__Chr...	0	0	0	0	0	[illegible]
**194**	d__Bacteria;p__Firmicutes;c__Incertae_Sedis;o_...	0	0	0	0	0	[illegible]
**195**	d__Bacteria;p__Firmicutes;c__Bacilli;o__Lactob...	0	0	0	0	0	[illegible]

196 rows × 111 columns

Step 2: Filter feature counts table with target sample columns

In [3]:
```
target_samples = [ sample for sample_group in list(dict_metadata.values()) for
 sample in sample_group]
df_feature_counts_filtered = df_feature_counts[target_samples]
df_feature_counts_filtered
```

Out[3]:

	F1-7	F1-8	F1-9	F1-10	F1-11	F1-12	F1-13	F1-14	F1-15	F3-7	F3-8	F3-9	F3-10	F[illegible]
**0**	8869	8035	5953	11460	10562	6795	6439	6312	13082	8445	319	10551	15735	14[illegible]
**1**	19991	23011	12533	27427	4496	12442	22199	7442	4318	34435	43961	31682	20382	17[illegible]
**2**	24450	10453	39194	12486	18793	13233	14613	32605	675	978	13	1555	34	
**3**	886	5723	480	6491	986	8890	10948	3644	2698	28326	10652	3300	3168	

**4**	655	2478	205	5444	384	3389	2020	1481	8495	3647	10380	3639	3886	3
**...**	...	...	...	...	...	...	...	...	...	...	...	...	...	
**191**	0	0	0	0	0	0	0	0	0	0	0	0	0	
**192**	0	0	0	0	0	0	0	0	0	0	0	0	0	
**193**	0	0	0	0	0	0	0	0	0	0	0	0	0	
**194**	0	0	0	0	0	0	0	0	0	0	0	0	0	
**195**	0	0	0	0	0	0	0	0	0	0	0	0	0	

196 rows × 15 columns

Step 3: Calculate total read counts per sample

In [4]:

```
sample_count_sums = df_feature_counts_filtered.sum(axis=0)
sample_count_sums
```

Out[4]:

```
F1-7      103534
F1-8      106286
F1-9       95787
F1-10     106356
F1-11      61083
F1-12     102329
F1-13     106433
F1-14     101965
F1-15      87272
F3-7       99046
F3-8      112057
F3-9      121801
F3-10      98907
F3-11      93879
F3-12     100855
dtype: int64
```

Step 4: Generate normalized counts feature table

In [5]:

```
df_feature_counts_filtered_norm = np.log10((df_feature_counts_filtered/sample_
count_sums)*sample_count_sums.mean() + 1)
df_feature_counts_filtered_norm
```

Out[5]:

	F1-7	F1-8	F1-9	F1-10	F1-11	F1-12	F1-13	F1-14	F1-15	F3-
**0**	3.932144	3.877869	3.792801	4.031765	4.237153	3.821558	3.781116	3.791088	4.175130	3.93011
**1**	4.285076	4.334781	4.116083	4.410735	3.866267	4.084229	4.318580	3.862600	3.693797	4.54047
**2**	4.372516	3.992111	4.611225	4.069000	4.487392	4.110995	4.136996	4.504148	2.888293	2.99424
**3**	2.932160	3.730530	2.700104	3.784923	3.207528	3.938255	4.011602	3.552551	3.489609	4.45566
**4**	2.801147	3.367113	2.331777	3.708543	2.798404	3.519506	3.277806	3.161705	3.987635	3.56551
**...**	...	...	...	...	...	...	...	...	...	.
**191**	0.000000	0.000000	0.000000	0.000000	0.000000	0.000000	0.000000	0.000000	0.000000	0.00000
**192**	0.000000	0.000000	0.000000	0.000000	0.000000	0.000000	0.000000	0.000000	0.000000	0.00000
**193**	0.000000	0.000000	0.000000	0.000000	0.000000	0.000000	0.000000	0.000000	0.000000	0.00000

**194**	0.000000	0.000000	0.000000	0.000000	0.000000	0.000000	0.000000	0.000000	0.000000	0.00000
**195**	0.000000	0.000000	0.000000	0.000000	0.000000	0.000000	0.000000	0.000000	0.000000	0.00000

196 rows × 15 columns

Step 5: Generate Bray-Curtis dissimilarity distance matrix

In [6]:

```
condensed_arr = pdist(df_feature_counts_filtered_norm.T, metric='braycurtis')
dist_mat_sym = squareform(condensed_arr)
print('Dimensions of symmetrical Bray-Curtris distance matrix: {}'.format(dist
_mat_sym.shape))
dist_mat_sym
```

```
Dimensions of symmetrical Bray-Curtris distance matrix: (15, 15)
```

Out[6]:

```
array([[0.        , 0.2354373 , 0.22808031, 0.22417016, 0.12012291,
        0.23853699, 0.24808563, 0.22766537, 0.28355288, 0.2810975 ,
        0.31556637, 0.36293577, 0.32842803, 0.31651299, 0.30864063],
       [0.2354373 , 0.        , 0.33151514, 0.14967141, 0.24991627,
        0.24001849, 0.21987228, 0.26156663, 0.27932507, 0.30678854,
        0.34797223, 0.23344469, 0.21036296, 0.20676359, 0.30008727],
       [0.22808031, 0.33151514, 0.        , 0.27522759, 0.23980917,
        0.30038263, 0.31849063, 0.27440435, 0.35510262, 0.33107932,
        0.40318452, 0.39587151, 0.40440186, 0.39791723, 0.37538382],
       [0.22417016, 0.14967141, 0.27522759, 0.        , 0.19583741,
        0.20769077, 0.19516   , 0.19688723, 0.26357676, 0.28888256,
        0.33578638, 0.22325478, 0.19780983, 0.21319542, 0.23524972],
       [0.12012291, 0.24991627, 0.23980917, 0.19583741, 0.        ,
        0.25426323, 0.26428184, 0.24114472, 0.28099155, 0.27165053,
        0.34444904, 0.3306451 , 0.30140723, 0.29320604, 0.31426941],
       [0.23853699, 0.24001849, 0.30038263, 0.20769077, 0.25426323,
        0.        , 0.07849586, 0.11983502, 0.17587685, 0.30023051,
        0.34731476, 0.27896171, 0.25896355, 0.26408819, 0.26365952],
       [0.24808563, 0.21987228, 0.31849063, 0.19516   , 0.26428184,
        0.07849586, 0.        , 0.12215787, 0.16324204, 0.2992072 ,
        0.34827216, 0.25906799, 0.24790667, 0.26258638, 0.25885452],
       [0.22766537, 0.26156663, 0.27440435, 0.19688723, 0.24114472,
        0.11983502, 0.12215787, 0.        , 0.19308055, 0.29340743,
        0.35498576, 0.28113353, 0.27029515, 0.28299124, 0.28161598],
       [0.28355288, 0.27932507, 0.35510262, 0.26357676, 0.28099155,
        0.17587685, 0.16324204, 0.19308055, 0.        , 0.30740146,
        0.32011341, 0.26906954, 0.28062787, 0.28590995, 0.29277325],
       [0.2810975 , 0.30678854, 0.33107932, 0.28888256, 0.27165053,
        0.30023051, 0.2992072 , 0.29340743, 0.30740146, 0.        ,
        0.17442066, 0.3409591 , 0.33767882, 0.32261034, 0.24950042],
       [0.31556637, 0.34797223, 0.40318452, 0.33578638, 0.34444904,
        0.34731476, 0.34827216, 0.35498576, 0.32011341, 0.17442066,
        0.        , 0.38938968, 0.3599006 , 0.33861661, 0.30290218],
       [0.36293577, 0.23344469, 0.39587151, 0.22325478, 0.3306451 ,
        0.27896171, 0.25906799, 0.28113353, 0.26906954, 0.3409591 ,
        0.38938968, 0.        , 0.17416425, 0.19202294, 0.29804967],
       [0.32842803, 0.21036296, 0.40440186, 0.19780983, 0.30140723,
        0.25896355, 0.24790667, 0.27029515, 0.28062787, 0.33767882,
        0.3599006 , 0.17416425, 0.        , 0.06813747, 0.27586168],
       [0.31651299, 0.20676359, 0.39791723, 0.21319542, 0.29320604,
        0.26408819, 0.26258638, 0.28299124, 0.28590995, 0.32261034,

        0.33861661, 0.19202294, 0.06813747, 0.        , 0.2958373 ],
```

```
 [0.30864063, 0.30008727, 0.37538382, 0.23524972, 0.31426941,
  0.26365952, 0.25885452, 0.28161598, 0.29277325, 0.24950042,
  0.30290218, 0.29804967, 0.27586168, 0.2958373 , 0.        ]])
```

Step 6: Perform principal coordinate analysis (PCoA)

In [7]:

```
import warnings
warnings.filterwarnings('ignore')
my_pcoa = skbio.stats.ordination.pcoa(dist_mat_sym)
df_pcoa = my_pcoa.samples[['PC1', 'PC2']]
# Normalize PC1 and PC2 into unit vectors
df_pcoa = pd.DataFrame(df_pcoa.to_numpy()/np.linalg.norm(df_pcoa.to_numpy(), a
xis=0))
print('PCoA proportion explained:')
my_pcoa.proportion_explained
```

```
PCoA proportion explained:
```

Out[7]:

```
PC1     0.302200
PC2     0.237754
PC3     0.153340
PC4     0.084033
PC5     0.065666
PC6     0.045122
PC7     0.039731
PC8     0.028952
PC9     0.018222
PC10    0.012539
PC11    0.007886
PC12    0.003585
PC13    0.000970
PC14    0.000000
PC15    0.000000
dtype: float64
```

Step 7: Plot PCoA results

In [8]:

```
colors = ['red', 'blue']
text_tup = [(-0.09, 0.00),
            (-0.1, -0.02)]

fig, ax = plt.subplots()
ax.set_xlim((-1.0, 1.25))
ax.set_ylim((-1.0, 1.25))
ax.set_yticks([-1.0, -0.5, 0.0, 0.5, 1.0])
ax.set_xticks([-1.0, -0.5, 0.0, 0.5, 1.0])
ax.set_xlabel('PCoA1 ({}%)'.format(np.round(my_pcoa.proportion_explained.PC1 *
 100, 2)))
ax.set_ylabel('PCoA2 ({}%)'.format(np.round(my_pcoa.proportion_explained.PC2 *
 100, 2)))
ax.set_title('Regenerated Figure')

end = 0
for idx, group_name in enumerate(dict_metadata):
    sample_names = dict_metadata[group_name]
    group_name = cross_ref_dict[group_name]
```

```
    group_count = len(sample_names)
    start = end
    end = start + group_count

    # multiply by -1 to rotate vector 180 degrees in order to match figure
    pc1 = df_pcoa.iloc[start:end, 0] * -1.0
    pc2 = df_pcoa.iloc[start:end, 1]
    # plot points
    ax.scatter(pc1, pc2, s=15, c=colors[idx],  label=group_name,
               alpha=0.5, edgecolors='none')
    cov = np.cov(pc1,pc2)
    x_mean = pc1.mean()
    y_mean = pc2.mean()
    e = get_cov_ellipse(cov, (x_mean, y_mean), 1,
                        ec=colors[idx], linewidth=2.0, alpha=0.4)
    ax.text(x_mean + text_tup[idx][0], y_mean + text_tup[idx][1], group_name,
            fontsize='x-small', c=colors[idx])
    ax.add_artist(e)

    for i, sample_name in enumerate(sample_names):
        ax.text(pc1.iloc[i], pc2.iloc[i], sample_cross_ref_dict[sample_name],
                fontsize='x-small', c=colors[idx], horizontalalignment='center',
               verticalalignment='center')
ax.legend(fontsize=6, loc='upper right')
plt.show()
```

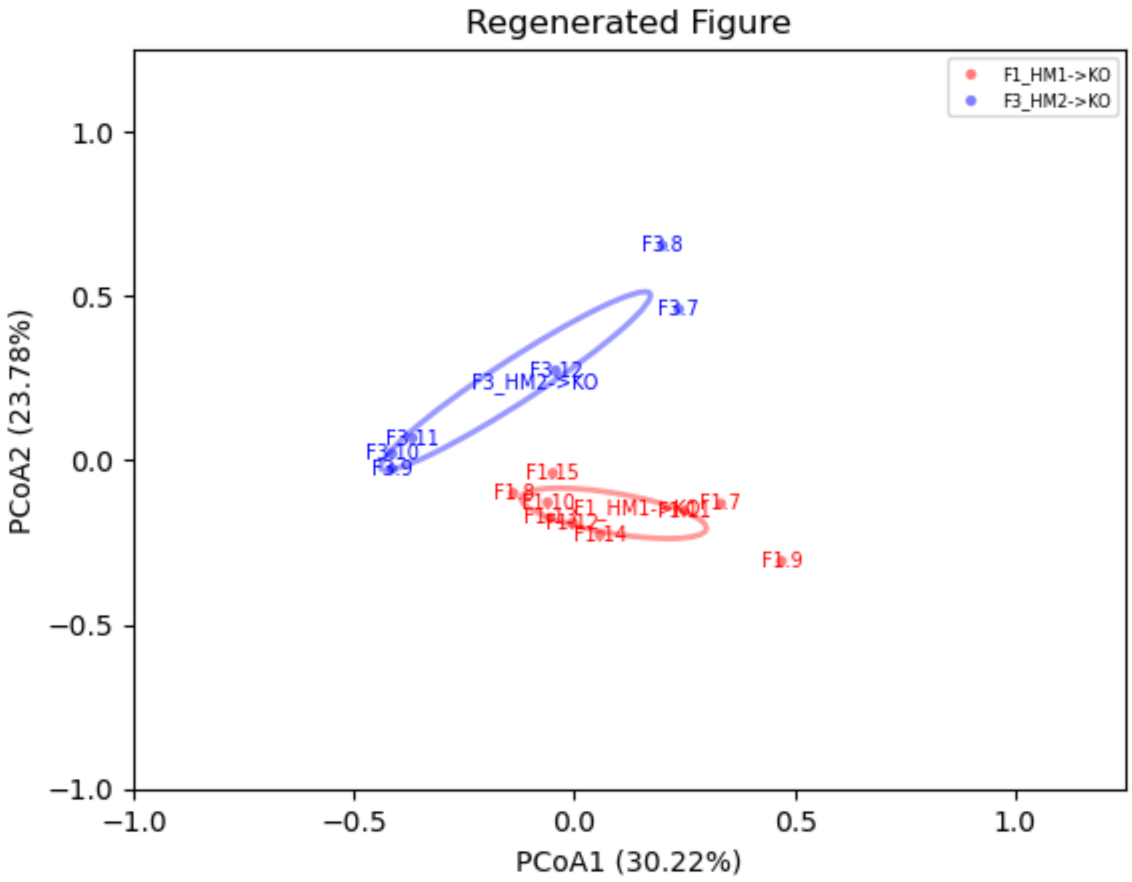

Step 8: Compare original and regenerated figures

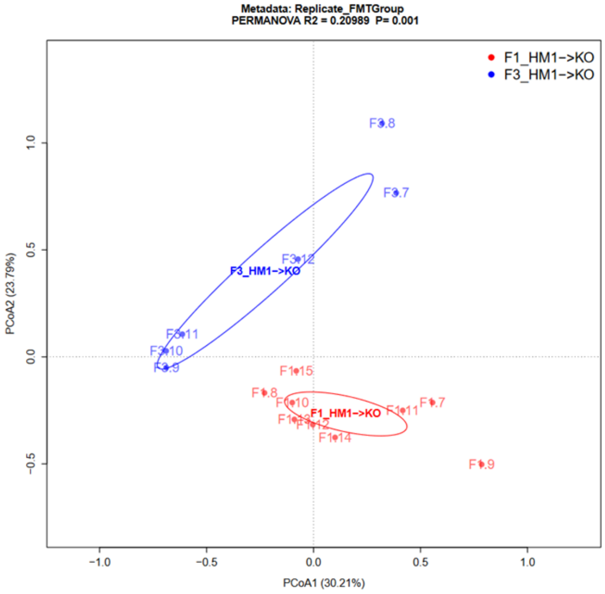
Metadata: Replicate_FMTGroup
PERMANOVA R2 = 0.20989 P= 0.001
F1_HM1->KO
F3_HM1->KO
F3_HM1->KO
F1_HM1->KO
PCoA1 (30.21%)
PCoA2 (23.79%)

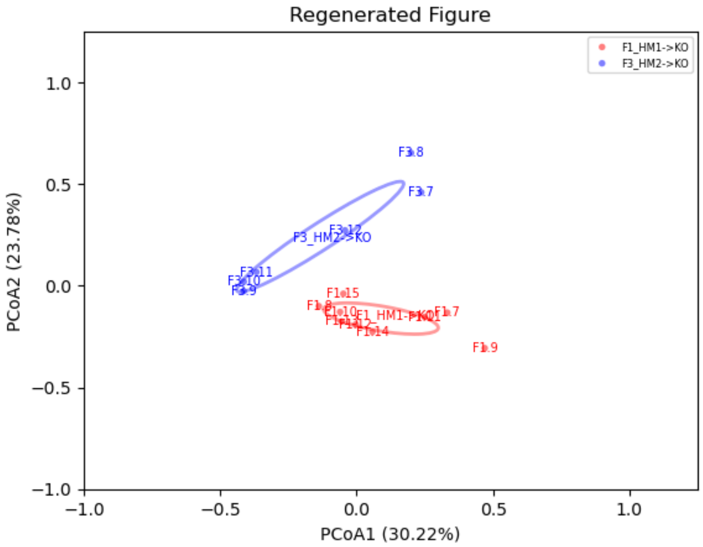
Regenerated Figure
F1_HM1->KO
F3_HM2->KO
F3_HM2->KO
PCoA1 (30.22%)
PCoA2 (23.78%)

Contents

- **Aim**
- **Main Python Methods**
- **Data Processing Steps**
  - Step 1: Retrieve source feature counts table
  - Step 2: Filter feature counts table with target sample columns
  - Step 3: Calculate total read counts per sample
  - Step 4: Generate normalized counts feature table
  - Step 5: Generate Bray-Curtis dissimilarity distance matrix
  - Step 6: Perform principal coordinate analysis (PCoA)
  - Step 7: Plot PCoA results
  - Step 8: Compare original and regenerated figures

Aim: Replicate figure

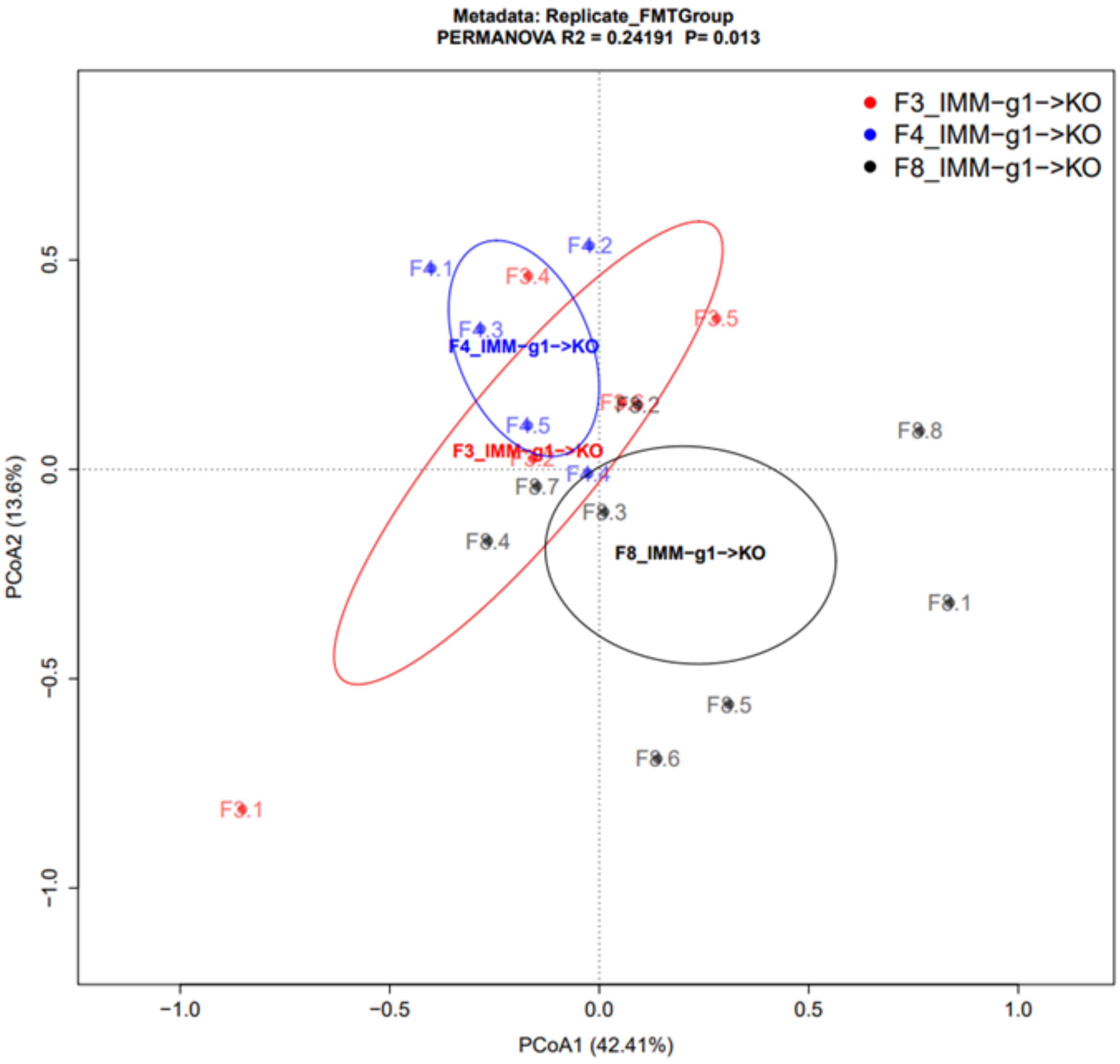

Main Python Methods

In [1]:
```
import numpy as np
import pandas as pd
import matplotlib.pyplot as plt
import os
import skbio
from scipy import stats
from scipy.spatial.distance import pdist, squareform
from matplotlib.patches import Ellipse
import matplotlib.transforms as transforms

# ******************************* MAIN METHODS ******************************
# Retrieve tab delimited file
def read_csv_file(file_path, skiprows=None, header = 0, sep = '\t', index_col=
False):
    df = pd.read_csv(file_path, skiprows=skiprows, sep=sep, header=header, ind
ex_col=index_col)

    return df

# Reference:  Function "get_cov_ellipse" was extracted from the following URL:

# https://scipython.com/book/chapter-7-matplotlib/examples/bmi-data-with-confi
dence-ellipses/
# (Learning Scientific Programming with Python by Christian Hill)
def get_cov_ellipse(cov, centre, nstd, **kwargs):
    """
    Return a matplotlib Ellipse patch representing the covariance matrix
    cov centred at centre and scaled by the factor nstd.
    """
    # Find and sort eigenvalues and eigenvectors into descending order
    eigvals, eigvecs = np.linalg.eigh(cov)
    order = eigvals.argsort()[::-1]
    eigvals, eigvecs = eigvals[order], eigvecs[:, order]

    # The anti-clockwise angle to rotate our ellipse by
    vx, vy = eigvecs[:,0][0], eigvecs[:,0][1]
    theta = np.arctan2(vy, vx)

    # Width and height of ellipse to draw
    width, height = 2 * nstd * np.sqrt(eigvals)
    return Ellipse(xy=centre, width=width, height=height,
                   angle=np.degrees(theta), fill=False, **kwargs)

# ******************************* INPUT FILE PATHS ***************************
****
# Get current working directory
current_working_dir = os.getcwd()
# original counts table file path
feature_tbl_file_path = os.path.join(current_working_dir, 'feature-table_balfo
ur.txt')
# dictionary for three sample groupings
dict_metadata = {'1gKOgtKO_1':['F3-1', 'F3-2', 'F3-4', 'F3-5', 'F3-6'],
                 '1gKOgtKO_2':['F4-1', 'F4-2', 'F4-3', 'F4-4', 'F4-5'],
                '1gKOgtKO_3':['F8-1', 'F8-2', 'F8-3', 'F8-4', 'F8-5', 'F8-6',
'F8-7', 'F8-8']}
# cross reference for sample grouping names
cross_ref_dict = {'1gKOgtKO_1':'F3_IMM-g1->KO', '1gKOgtKO_2':'F4_IMM-g1->KO',
'1gKOgtKO_3':'F8_IMM-g1->KO'}
# criss referebce for sample names
sample_cross_ref_dict = {'F3-1':'F3.1', 'F3-2':'F3.2', 'F3-4':'F3.4', 'F3-5':
'F3.5', 'F3-6':'F3.6',
```

```
                        'F4-1':'F4.1', 'F4-2':'F4.2', 'F4-3':'F4.3', 'F4-4':'F
4.4', 'F4-5':'F4.5',
                        'F8-1':'F8.1', 'F8-2':'F8.2', 'F8-3':'F8.3', 'F8-4':'F
8.4',
                         'F8-5':'F8.5', 'F8-6':'F8.6', 'F8-7':'F8.7', 'F8-8':
'F8.8'}
```

Data Processing Steps

Step 1: Retrieve source feature counts table

In [2]:

```
df_feature_counts = read_csv_file(feature_tbl_file_path, 1)
df_feature_counts = df_feature_counts.astype({col:'int32' for col in df_featur
e_counts.columns[1:]})
df_feature_counts
```

Out[2]:

	#OTU ID	1gKO.1	1gKO.2	1gKO.3	1gWT.1	1gWT.2	1gWT.[illegible]
**0**	d__Bacteria;p__Verrucomicrobiota;c__Verrucomic...	11370	14120	14648	16632	19817	2170[illegible]
**1**	d__Bacteria;p__Firmicutes;c__Clostridia;o__Lac...	22545	25836	17048	7547	9272	599[illegible]
**2**	d__Bacteria;p__Proteobacteria;c__Gammaproteoba...	5919	6481	6714	31	31	3[illegible]
**3**	d__Bacteria;p__Firmicutes;c__Clostridia;o__Lac...	8134	9827	7090	8264	10100	632[illegible]
**4**	d__Bacteria;p__Firmicutes;c__Clostridia;o__Lac...	6280	7649	5719	9715	11851	804[illegible]
**...**	...	...	...	...	...	...	..
**191**	d__Bacteria;p__Firmicutes;c__Clostridia;o__Pep...	0	0	0	0	0	[illegible]
**192**	d__Bacteria;p__Firmicutes;c__Clostridia;o__Clo...	0	0	0	0	0	[illegible]
**193**	d__Bacteria;p__Firmicutes;c__Clostridia;o__Chr...	0	0	0	0	0	[illegible]
**194**	d__Bacteria;p__Firmicutes;c__Incertae_Sedis;o__...	0	0	0	0	0	[illegible]
**195**	d__Bacteria;p__Firmicutes;c__Bacilli;o__Lactob...	0	0	0	0	0	[illegible]

196 rows × 111 columns

Step 2: Filter feature counts table with target sample columns

In [3]:

```
target_samples = [ sample for sample_group in list(dict_metadata.values()) for
 sample in sample_group]
df_feature_counts_filtered = df_feature_counts[target_samples]
df_feature_counts_filtered
```

Out[3]:

	F3-1	F3-2	F3-4	F3-5	F3-6	F4-1	F4-2	F4-3	F4-4	F4-5	F8-1	F8-2	F8-3	F[illegible]
**0**	17605	8824	16088	9737	11518	24109	16317	23844	22240	22298	2627	12243	9359	15[illegible]
**1**	292	25296	9605	2068	25952	3552	6985	6857	8085	4026	10750	7020	8879	13[illegible]
**2**	40	2810	20375	38880	6836	1158	26515	18156	19808	23320	30563	28879	38922	8[illegible]
**3**	1952	20829	4524	1738	6522	7172	2984	7713	3631	7010	1440	5601	5679	10[illegible]

**4**	10538	2268	2403	1978	2505	2330	1026	2151	1346	1347	56	739	1149	2
**...**	...	...	...	...	...	...	...	...	...	...	...	...	...	
**191**	0	0	2	0	0	0	0	0	0	0	0	0	0	
**192**	0	0	0	2	0	0	0	0	0	0	0	0	0	
**193**	0	0	0	0	0	0	0	0	0	0	0	0	0	
**194**	0	0	0	0	0	0	0	0	0	0	0	0	0	
**195**	0	0	0	0	0	0	0	0	0	0	0	0	0	

196 rows × 18 columns

Step 3: Calculate total read counts per sample

In [4]:

```
sample_count_sums = df_feature_counts_filtered.sum(axis=0)
sample_count_sums
```

Out[4]:

```
F3-1     89907
F3-2     95182
F3-4    110663
F3-5    122538
F3-6    107733
F4-1     93069
F4-2    102268
F4-3    106452
F4-4    102778
F4-5    108196
F8-1    101846
F8-2    103946
F8-3     99383
F8-4    108840
F8-5     19323
F8-6     96062
F8-7    102664
F8-8     84853
dtype: int64
```

Step 4: Generate normalized counts feature table

In [5]:

```
df_feature_counts_filtered_norm = np.log10((df_feature_counts_filtered/sample_
count_sums)*sample_count_sums.mean() + 1)
df_feature_counts_filtered_norm
```

Out[5]:

	F3-1	F3-2	F3-4	F3-5	F3-6	F4-1	F4-2	F4-3	F4-4	F4-
**0**	4.281044	3.956337	4.151709	3.889389	4.018249	4.402570	4.192107	4.339424	4.324434	4.30325
**1**	2.502137	4.413692	3.927725	3.216722	4.371019	3.570963	3.823670	3.798228	3.885015	3.55996
**2**	1.647339	3.459481	4.254298	4.490648	3.791702	3.084440	4.402948	4.221071	4.274142	4.32271
**3**	3.326070	4.329312	3.600808	3.141271	3.771284	3.876072	3.454390	3.849309	3.537431	3.80075
**4**	4.058181	3.366454	3.326135	3.197410	3.355829	3.387907	2.991030	3.294885	3.106663	3.08469
**...**	...	...	...	...	...	...	...	...	...	.
**191**	0.000000	0.000000	0.441351	0.000000	0.000000	0.000000	0.000000	0.000000	0.000000	0.00000

**192**	0.000000	0.000000	0.000000	0.413632	0.000000	0.000000	0.000000	0.000000	0.000000	0.00000
**193**	0.000000	0.000000	0.000000	0.000000	0.000000	0.000000	0.000000	0.000000	0.000000	0.00000
**194**	0.000000	0.000000	0.000000	0.000000	0.000000	0.000000	0.000000	0.000000	0.000000	0.00000
**195**	0.000000	0.000000	0.000000	0.000000	0.000000	0.000000	0.000000	0.000000	0.000000	0.00000

196 rows × 18 columns

Step 5: Generate Bray-Curtis dissimilarity distance matrix

In [6]:

```
condensed_arr = pdist(df_feature_counts_filtered_norm.T, metric='braycurtis')
dist_mat_sym = squareform(condensed_arr)
print('Dimensions of symmetrical Bray-Curtris distance matrix: {}'.format(dist
_mat_sym.shape))
dist_mat_sym
```

```
Dimensions of symmetrical Bray-Curtris distance matrix: (18, 18)
```

Out[6]:

```
array([[0.        , 0.28454686, 0.29267021, 0.36808693, 0.32967506,
        0.2605009 , 0.29730312, 0.24727062, 0.28361299, 0.25890973,
        0.44438252, 0.29948291, 0.27046481, 0.21882052, 0.36697641,
        0.30325325, 0.25152437, 0.45398825],
       [0.28454686, 0.        , 0.15342963, 0.21946027, 0.1471099 ,
        0.20831996, 0.18695832, 0.18255075, 0.21447577, 0.19886628,
        0.33151212, 0.21464976, 0.17580187, 0.18108425, 0.2389772 ,
        0.19459264, 0.17578575, 0.29076401],
       [0.29267021, 0.15342963, 0.        , 0.14439185, 0.1778078 ,
        0.1458174 , 0.11984601, 0.11024685, 0.145016  , 0.12118633,
        0.32270016, 0.17400607, 0.16635111, 0.14562586, 0.22162812,
        0.20358452, 0.14600089, 0.28600799],
       [0.36808693, 0.21946027, 0.14439185, 0.        , 0.24023838,
        0.24230403, 0.18393303, 0.20344634, 0.19103171, 0.19224901,
        0.24405912, 0.18131433, 0.19226307, 0.2595383 , 0.19738032,
        0.21403131, 0.2074863 , 0.23233857],
       [0.32967506, 0.1471099 , 0.1778078 , 0.24023838, 0.        ,
        0.22602656, 0.18825317, 0.20670047, 0.22431224, 0.2196271 ,
        0.28836239, 0.19908431, 0.18851052, 0.19963634, 0.2392397 ,
        0.22310475, 0.20029594, 0.24800114],
       [0.2605009 , 0.20831996, 0.1458174 , 0.24230403, 0.22602656,
        0.        , 0.13228615, 0.08405766, 0.16189807, 0.15142551,
        0.36028806, 0.20185117, 0.17992468, 0.12857937, 0.28657616,
        0.2400629 , 0.14094868, 0.32119404],
       [0.29730312, 0.18695832, 0.11984601, 0.18393303, 0.18825317,
        0.13228615, 0.        , 0.0956604 , 0.141664  , 0.1371242 ,
        0.25628948, 0.13370686, 0.1419186 , 0.15473474, 0.26008824,
        0.22197027, 0.14452128, 0.22554503],
       [0.24727062, 0.18255075, 0.11024685, 0.20344634, 0.20670047,
        0.08405766, 0.0956604 , 0.        , 0.11693841, 0.10950776,
        0.3218014 , 0.17444426, 0.16195855, 0.125435  , 0.2403541 ,
        0.21527653, 0.13417896, 0.28984225],
       [0.28361299, 0.21447577, 0.145016  , 0.19103171, 0.22431224,
        0.16189807, 0.141664  , 0.11693841, 0.        , 0.09469814,
        0.26741369, 0.14436788, 0.13571197, 0.13232554, 0.19829445,
        0.15854515, 0.12310822, 0.23427458],
       [0.25890973, 0.19886628, 0.12118633, 0.19224901, 0.2196271 ,
        0.15142551, 0.1371242 , 0.10950776, 0.09469814, 0.        ,

        0.30136331, 0.13609655, 0.11308482, 0.11049238, 0.21923821,
```

```
        0.19349934, 0.11755898, 0.26633493],
       [0.44438252, 0.33151212, 0.32270016, 0.24405912, 0.28836239,
        0.36028806, 0.25628948, 0.3218014 , 0.26741369, 0.30136331,
        0.        , 0.2350507 , 0.25292598, 0.3100213 , 0.25768962,
        0.24297859, 0.29501488, 0.11959038],
       [0.29948291, 0.21464976, 0.17400607, 0.18131433, 0.19908431,
        0.20185117, 0.13370686, 0.17444426, 0.14436788, 0.13609655,
        0.2350507 , 0.        , 0.12171157, 0.15559515, 0.24067187,
        0.21444788, 0.13243287, 0.20929897],
       [0.27046481, 0.17580187, 0.16635111, 0.19226307, 0.18851052,
        0.17992468, 0.1419186 , 0.16195855, 0.13571197, 0.11308482,
        0.25292598, 0.12171157, 0.        , 0.11402347, 0.20420303,
        0.18106997, 0.11758264, 0.20746761],
       [0.21882052, 0.18108425, 0.14562586, 0.2595383 , 0.19963634,
        0.12857937, 0.15473474, 0.125435  , 0.13232554, 0.11049238,
        0.3100213 , 0.15559515, 0.11402347, 0.        , 0.2390292 ,
        0.17429227, 0.10710297, 0.26585477],
       [0.36697641, 0.2389772 , 0.22162812, 0.19738032, 0.2392397 ,
        0.28657616, 0.26008824, 0.2403541 , 0.19829445, 0.21923821,
        0.25768962, 0.24067187, 0.20420303, 0.2390292 , 0.        ,
        0.15346262, 0.20293655, 0.24230826],
       [0.30325325, 0.19459264, 0.20358452, 0.21403131, 0.22310475,
        0.2400629 , 0.22197027, 0.21527653, 0.15854515, 0.19349934,
        0.24297859, 0.21444788, 0.18106997, 0.17429227, 0.15346262,
        0.        , 0.1867873 , 0.23780244],
       [0.25152437, 0.17578575, 0.14600089, 0.2074863 , 0.20029594,
        0.14094868, 0.14452128, 0.13417896, 0.12310822, 0.11755898,
        0.29501488, 0.13243287, 0.11758264, 0.10710297, 0.20293655,
        0.1867873 , 0.        , 0.2521906 ],
       [0.45398825, 0.29076401, 0.28600799, 0.23233857, 0.24800114,
        0.32119404, 0.22554503, 0.28984225, 0.23427458, 0.26633493,
        0.11959038, 0.20929897, 0.20746761, 0.26585477, 0.24230826,
        0.23780244, 0.2521906 , 0.        ]])
```

Step 6: Perform principal coordinate analysis (PCoA)

In [7]:
```python
import warnings
warnings.filterwarnings('ignore')
my_pcoa = skbio.stats.ordination.pcoa(dist_mat_sym)
df_pcoa = my_pcoa.samples[['PC1', 'PC2']]
# Normalize PC1 and PC2 into unit vectors
df_pcoa = pd.DataFrame(df_pcoa.to_numpy()/np.linalg.norm(df_pcoa.to_numpy(), a
xis=0))
print('PCoA proportion explained:')
my_pcoa.proportion_explained
```

```
PCoA proportion explained:
```

Out[7]:
```
PC1     0.423829
PC2     0.136141
PC3     0.125652
PC4     0.102058
PC5     0.065312
PC6     0.044789
PC7     0.028576
PC8     0.022913
PC9     0.017731
PC10    0.013240
```

```
PC11     0.009806
PC12     0.005152
PC13     0.003683
PC14     0.001117
PC15     0.000000
PC16     0.000000
PC17     0.000000
PC18     0.000000
dtype: float64
```

Step 7: Plot PCoA results

In [8]:
```
colors = ['red', 'blue', 'black']
text_tup = [(-0.1, 0.00),
            (-0.1, -0.02),
            (-0.1, -0.02)]

fig, ax = plt.subplots()
ax.set_xlim((-1.0, 0.8))
ax.set_ylim((-1.0, 0.8))
ax.set_yticks([-1.0, -0.5, 0.0, 0.5])
ax.set_xticks([-1.0, -0.5, 0.0, 0.5])
ax.set_xlabel('PCoA1 ({}%)'.format(np.round(my_pcoa.proportion_explained.PC1 *
 100, 2)))
ax.set_ylabel('PCoA2 ({}%)'.format(np.round(my_pcoa.proportion_explained.PC2 *
 100, 2)))
ax.set_title('Regenerated Figure')

end = 0
for idx, group_name in enumerate(dict_metadata):
    sample_names = dict_metadata[group_name]
    group_name = cross_ref_dict[group_name]
    group_count = len(sample_names)
    start = end
    end = start + group_count

    # multiply by -1 to rotate vector 180 degrees in order to match figure
    pc1 = df_pcoa.iloc[start:end, 0] *-1.0
    # multiply by -1 to rotate vector 180 degrees in order to match figure
    pc2 = df_pcoa.iloc[start:end, 1] *-1.0
    # plot points
    ax.scatter(pc1, pc2, s=15, c=colors[idx],  label=group_name,
               alpha=0.5, edgecolors='none')
    cov = np.cov(pc1,pc2)
    x_mean = pc1.mean()
    y_mean = pc2.mean()
    e = get_cov_ellipse(cov, (x_mean, y_mean), 1,
                        ec=colors[idx], linewidth=2.0, alpha=0.4)
    ax.text(x_mean + text_tup[idx][0], y_mean + text_tup[idx][1], group_name,
            fontsize='6', c=colors[idx])
    ax.add_artist(e)

    for i, sample_name in enumerate(sample_names):
        ax.text(pc1.iloc[i], pc2.iloc[i], sample_cross_ref_dict[sample_name],
                fontsize='x-small', c=colors[idx], horizontalalignment='cente
r',
               verticalalignment='center')
ax.legend(fontsize=8, loc='upper right')
plt.show()
```

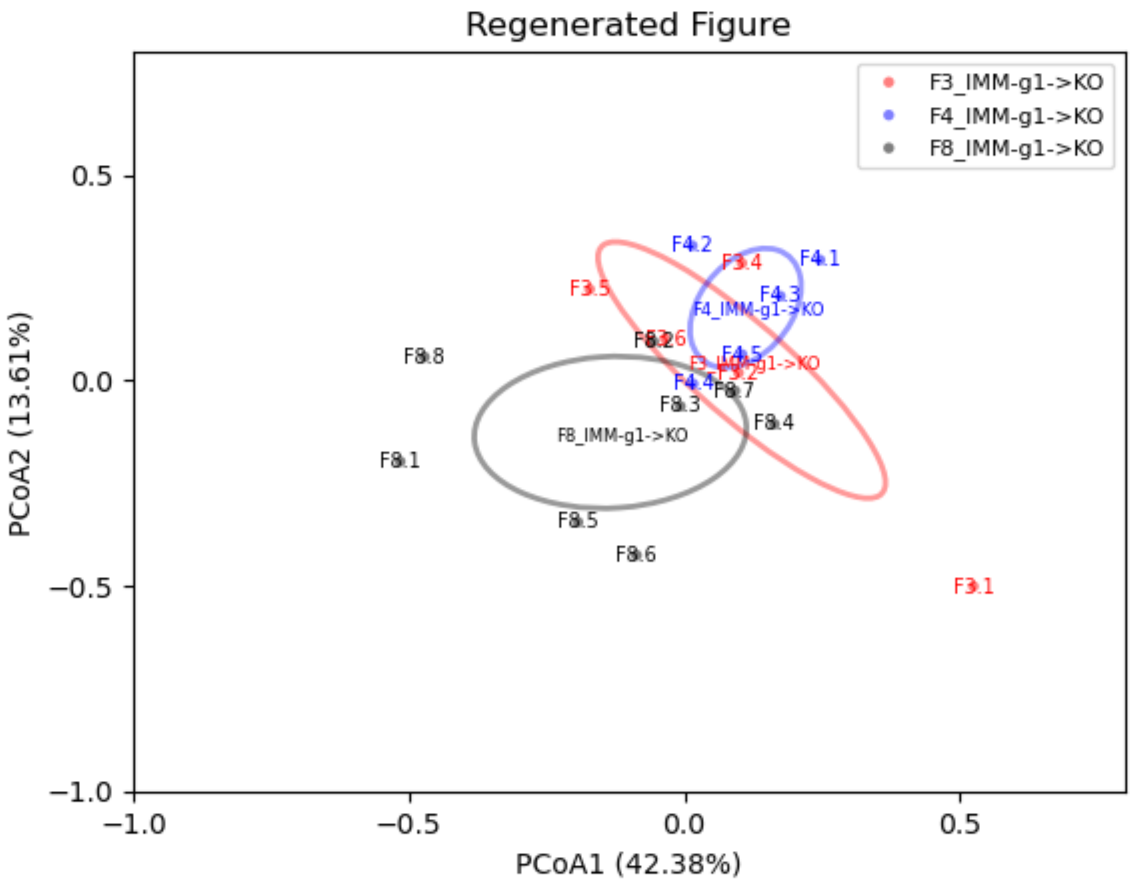

Step 8: Compare original and regenerated figures

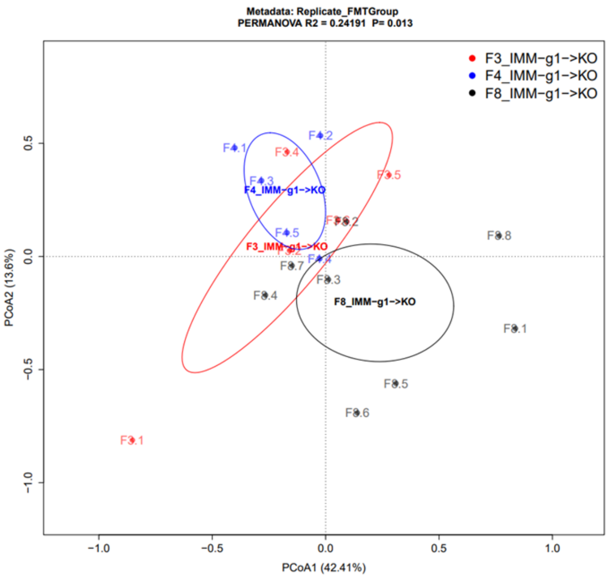

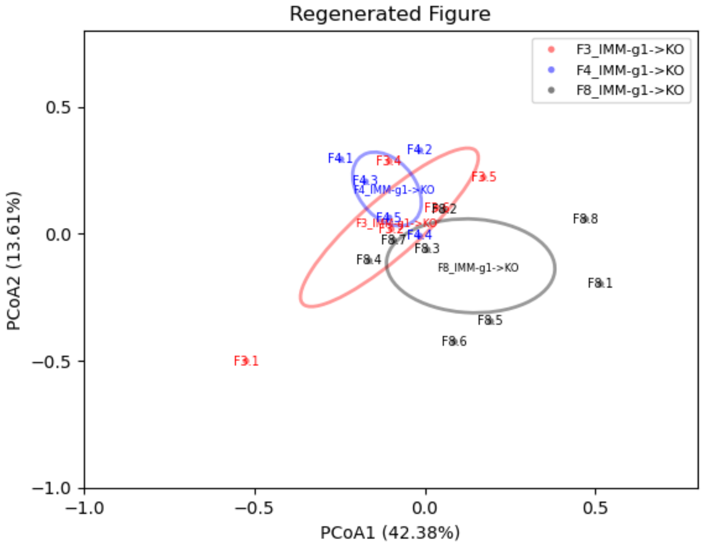

Contents

- **Aim**
- **Main Python Methods**
- **Data Processing Steps**
  - Step 1: Retrieve source feature counts table
  - Step 2: Filter feature counts table with seven target sample group columns
  - Step 3: Calculate total read counts per sample
  - Step 4: Generate normalized counts feature table (without log10)
  - Step 5: Identify top eight most abundant taxa
  - Step 6: Calculate proportional taxa abundance for each sample group
  - Step 7: Plot stacked bar plot results for each sample group
  - Step 8: Compare original and regenerated figures

Aim: Replicate figure

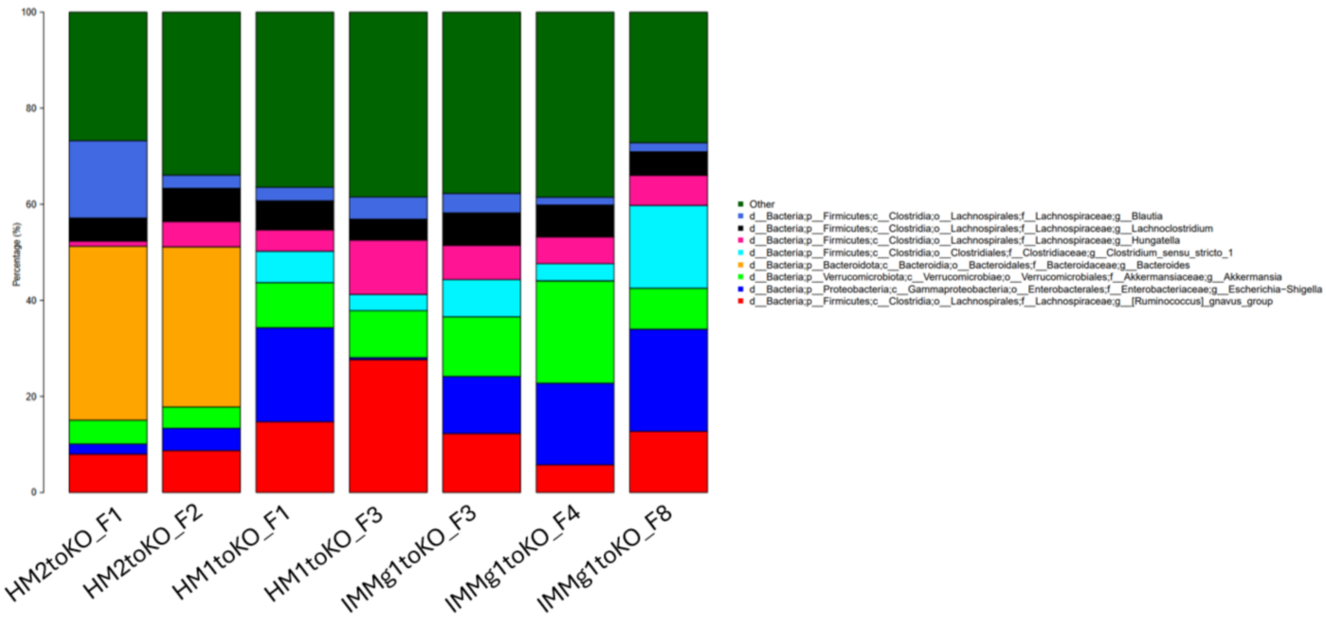

Main Python Methods

In [1]:
```
import numpy as np
import pandas as pd
import matplotlib.pyplot as plt
import os

# ****************************** MAIN METHODS ******************************
# Retrieve tab delimited file
def read_csv_file(file_path, skiprows=None, header = 0, sep = '\t', index_col=
False):
    df = pd.read_csv(file_path, skiprows=skiprows, sep=sep, header=header, ind
ex_col=index_col)

    return df
```

```
# Reference:  Function "get_cov_ellipse" was extracted from the following URL:

# https://scipython.com/book/chapter-7-matplotlib/examples/bmi-data-with-confi
dence-ellipses/
# (Learning Scientific Programming with Python by Christian Hill)
def get_cov_ellipse(cov, centre, nstd, **kwargs):
    """
    Return a matplotlib Ellipse patch representing the covariance matrix
    cov centred at centre and scaled by the factor nstd.
    """
    # Find and sort eigenvalues and eigenvectors into descending order
    eigvals, eigvecs = np.linalg.eigh(cov)
    order = eigvals.argsort()[::-1]
    eigvals, eigvecs = eigvals[order], eigvecs[:, order]

    # The anti-clockwise angle to rotate our ellipse by
    vx, vy = eigvecs[:,0][0], eigvecs[:,0][1]
    theta = np.arctan2(vy, vx)

    # Width and height of ellipse to draw
    width, height = 2 * nstd * np.sqrt(eigvals)
    return Ellipse(xy=centre, width=width, height=height,
                   angle=np.degrees(theta), fill=False, **kwargs)

# ****************************** INPUT FILE PATHS ***************************
****
# Get current working directory
current_working_dir = os.getcwd()
# original counts table file path
feature_tbl_file_path = os.path.join(current_working_dir, 'feature-table_balfo
ur.txt')
# dictionary for two sample groupings
dict_metadata = {'129.IL10KO_1':['F1-1', 'F1-2', 'F1-3', 'F1-4', 'F1-5', 'F1-
6'],
                 '129.IL10KO_2':['F2-1' ,'F2-2', 'F2-3', 'F2-4', 'F2-5'],
                'hFMT.1.2.3.gtKO_1':['F1-7', 'F1-8', 'F1-9', 'F1-10', 'F1-11',
 'F1-12', 'F1-13', 'F1-14', 'F1-15'],
                 'hFMT.1.2.3.gtKO_2':['F3-7', 'F3-8', 'F3-9', 'F3-10', 'F3-11'
, 'F3-12'],
                '1gKOgtKO_1':['F3-1', 'F3-2', 'F3-4', 'F3-5', 'F3-6'],
                 '1gKOgtKO_2':['F4-1', 'F4-2', 'F4-3', 'F4-4', 'F4-5'],
                '1gKOgtKO_3':['F8-1', 'F8-2', 'F8-3', 'F8-4', 'F8-5', 'F8-6',
'F8-7', 'F8-8']}
# cross reference for sample grouping names
cross_ref_dict = {'129.IL10KO_1':'HM2toKO_F1',
                  '129.IL10KO_2':'HM2toKO_F2',
                  'hFMT.1.2.3.gtKO_1':'HM1toKO_F1',
                  'hFMT.1.2.3.gtKO_2':'HM1toKO_F3',
                 '1gKOgtKO_1':'IMMg1toKO_F3',
                  '1gKOgtKO_2':'IMMg1toKO_F4',
                  '1gKOgtKO_3':'IMMg1toKO_F8'}
# stacked bar plot colors
colors = ["red","blue","lightgreen","orange","turquoise","deeppink","black","r
oyalblue","darkgreen"]
# Set parameter for top N most abundant taxa across all sample groups
top_most_abundant_count = 8
```

Data Processing Steps

Step 1: Retrieve source feature counts table

In [2]:
```
df_feature_counts = read_csv_file(feature_tbl_file_path, 1)
df_feature_counts = df_feature_counts.astype({col:'int32' for col in df_featur
e_counts.columns[1:]})
df_feature_counts
```

Out[2]:

	#OTU ID	1gKO.1	1gKO.2	1gKO.3	1gWT.1	1gWT.2	1gWT.[illegible]
**0**	d__Bacteria;p__Verrucomicrobiota;c__Verrucomic...	11370	14120	14648	16632	19817	2170[illegible]
**1**	d__Bacteria;p__Firmicutes;c__Clostridia;o__Lac...	22545	25836	17048	7547	9272	599[illegible]
**2**	d__Bacteria;p__Proteobacteria;c__Gammaproteoba...	5919	6481	6714	31	31	3[illegible]
**3**	d__Bacteria;p__Firmicutes;c__Clostridia;o__Lac...	8134	9827	7090	8264	10100	632[illegible]
**4**	d__Bacteria;p__Firmicutes;c__Clostridia;o__Lac...	6280	7649	5719	9715	11851	804[illegible]
**...**	...	...	...	...	...	...	[illegible]
**191**	d__Bacteria;p__Firmicutes;c__Clostridia;o__Pep...	0	0	0	0	0	[illegible]
**192**	d__Bacteria;p__Firmicutes;c__Clostridia;o__Clo...	0	0	0	0	0	[illegible]
**193**	d__Bacteria;p__Firmicutes;c__Clostridia;o__Chr...	0	0	0	0	0	[illegible]
**194**	d__Bacteria;p__Firmicutes;c__Incertae_Sedis;o_...	0	0	0	0	0	[illegible]
**195**	d__Bacteria;p__Firmicutes;c__Bacilli;o__Lactob...	0	0	0	0	0	[illegible]

196 rows × 111 columns

Step 2: Filter feature counts table with seven target sample group columns

In [3]:
```
target_samples = [ sample for sample_group in list(dict_metadata.values()) for
 sample in sample_group ]
df_feature_counts_filtered = df_feature_counts[target_samples]
df_feature_counts_filtered
```

Out[3]:

	F1-1	F1-2	F1-3	F1-4	F1-5	F1-6	F2-1	F2-2	F2-3	F2-4	...	F4-4	F4-5	F8-1	[illegible]
**0**	10401	2753	7914	2398	6595	2313	8957	10961	344	460	...	22240	22298	2627	1[illegible]
**1**	7133	7051	3523	26054	524	2105	10749	5732	14621	319	...	8085	4026	10750	
**2**	2132	5867	4116	54	87	284	2194	1425	3296	1071	...	19808	23320	30563	2[illegible]
**3**	1965	394	779	828	929	1973	4084	39	2827	17547	...	3631	7010	1440	
**4**	4785	1982	3119	2274	41521	49604	3306	3350	3278	2247	...	1346	1347	56	
**...**	...	...	...	...	...	...	...	...	...	...	...	...	...	...	
**191**	0	0	0	0	0	0	0	0	0	0	...	0	0	0	
**192**	0	0	0	0	0	0	0	0	0	0	...	0	0	0	
**193**	0	0	0	0	0	0	0	0	0	0	...	0	0	0	
**194**	0	0	0	0	0	0	0	0	0	0	...	0	0	0	
**195**	0	0	0	0	0	0	0	0	0	0	...	0	0	0	

196 rows × 44 columns

Step 3: Calculate total read counts per sample

In [4]:
```
sample_count_sums = df_feature_counts_filtered.sum(axis=0)
sample_count_sums
```

Out[4]:
```
F1-1     120620
F1-2      89533
F1-3     103243
F1-4      92643
F1-5     120934
F1-6      98057
F2-1      98214
F2-2      90013
F2-3      95932
F2-4     111659
F2-5      87600
F1-7     103534
F1-8     106286
F1-9      95787
F1-10    106356
F1-11     61083
F1-12    102329
F1-13    106433
F1-14    101965
F1-15     87272
F3-7      99046
F3-8     112057
F3-9     121801
F3-10     98907
F3-11     93879
F3-12    100855
F3-1      89907
F3-2      95182
F3-4     110663
F3-5     122538
F3-6     107733
F4-1      93069
F4-2     102268
F4-3     106452
F4-4     102778
F4-5     108196
F8-1     101846
F8-2     103946
F8-3      99383
F8-4     108840
F8-5      19323
F8-6      96062
F8-7     102664
F8-8      84853
dtype: int64
```

Step 4: Generate normalized counts feature table (without log10)

In [5]:
```
df_feature_counts_filtered_norm = (df_feature_counts_filtered/sample_count_sum
s)*sample_count_sums.mean()
```

```
df_feature_counts_filtered_norm
```

Out[5]:

	F1-1	F1-2	F1-3	F1-4	F1-5	F1-6	F2-1
**0**	8547.969608	3048.107446	7598.758242	2565.923864	5405.969352	2338.321527	9040.581637 1
**1**	5862.192790	7806.830948	3382.666829	27878.473871	429.526602	2128.044450	10849.303564
**2**	1752.165292	6495.912235	3952.045606	57.781438	71.314531	287.109085	2214.473162
**3**	1614.917823	436.234774	747.969759	885.982051	761.508041	1994.599383	4122.109569
**4**	3932.509814	2194.460210	2994.759534	2433.240561	34035.064968	50147.038912	3336.849715
**...**	...	...	...	...	...	...	...
**191**	0.000000	0.000000	0.000000	0.000000	0.000000	0.000000	0.000000
**192**	0.000000	0.000000	0.000000	0.000000	0.000000	0.000000	0.000000
**193**	0.000000	0.000000	0.000000	0.000000	0.000000	0.000000	0.000000
**194**	0.000000	0.000000	0.000000	0.000000	0.000000	0.000000	0.000000
**195**	0.000000	0.000000	0.000000	0.000000	0.000000	0.000000	0.000000

196 rows × 44 columns

Step 5: Identify top eight most abundant taxa

In [6]:

```
df_feature_counts_filtered_norm.index = df_feature_counts['#OTU ID']
df_top_eight_genera = pd.DataFrame(df_feature_counts_filtered_norm.mean(axis=1
).\
                                    sort_values(ascending=False)[:top_most_abun
dant_count])
df_top_eight_genera.columns = ['norm_mean_abundance']
df_top_eight_genera
```

Out[6]:

#(
**d__Bacteria;p__Firmicutes;c__Clostridia;o__Lachnospirales;f__Lachnospiraceae;g__[Ruminococcus]_gnavus_**
**d__Bacteria;p__Proteobacteria;c__Gammaproteobacteria;o__Enterobacterales;f__Enterobacteriaceae;g__Esche S**
**d__Bacteria;p__Verrucomicrobiota;c__Verrucomicrobiae;o__Verrucomicrobiales;f__Akkermansiaceae;g__Akkerı**
**d__Bacteria;p__Bacteroidota;c__Bacteroidia;o__Bacteroidales;f__Bacteroidaceae;g__Bacte**
**d__Bacteria;p__Firmicutes;c__Clostridia;o__Clostridiales;f__Clostridiaceae;g__Clostridium_sensu_st**
**d__Bacteria;p__Firmicutes;c__Clostridia;o__Lachnospirales;f__Lachnospiraceae;g__Hun**
**d__Bacteria;p__Firmicutes;c__Clostridia;o__Lachnospirales;f__Lachnospiraceae;g__Lachnoclost**
**d__Bacteria;p__Firmicutes;c__Clostridia;o__Lachnospirales;f__Lachnospiraceae;g__l**

Step 6: Calculate proportional taxa abundance for each sample group

In [7]:

```
# Initialize dictionary to store proportional abundances for each sample group
taxa_dict = {}
```

```
for taxa in df_top_eight_genera.index:
    taxa_dict[taxa] = []
taxa_dict['Other'] = []
sample_group_list = []

# Populate dictionary for sample groups
for sample_group in dict_metadata:
    cross_ref_dict[sample_group]
    sample_group_list.append(cross_ref_dict[sample_group])
    # For a given sample group, calculate the sum of the normalized counts for
 each taxa
    ser = df_feature_counts_filtered_norm[dict_metadata[sample_group]].sum(axi
s=1)
    # For a given sample group, calculate the proportion for each taxa
    ser_prop = ser/ser.sum()
    # For a given sample group, verify that the sum of all taxa proportions is
 equal to zero
    assert np.isclose(ser_prop.sum(), 1.0)
    # Filter out top eight most abundant taxa previously identified in step 5
    ser_prop_top_eight =  ser_prop[df_top_eight_genera.index]
    # Calculate and store the sum of proportions for all other taxa
    # that are not in the set of the top eight taxa
    other = 1.0 - ser_prop_top_eight.sum()
    taxa_dict['Other'].append(other)
    # Store the proportions for the top eight most abundant taxa
    for item in ser_prop_top_eight.items():
        taxa_dict[item[0]].append(item[1])

# Save data to a dataframe to plot during the following step
df_taxa = pd.DataFrame(taxa_dict)
df_taxa.index = sample_group_list
# Multiply by 100 to convert to percentage
df_taxa = df_taxa * 100
df_taxa
```

Out[7]:

	d__Bacteria;p__Firmicutes;c__Clostridia;o__Lachnospirales;f__Lachnospiraceae;g__[Ruminoc
**HM2toKO_F1**	
**HM2toKO_F2**	
**HM1toKO_F1**	
**HM1toKO_F3**	
**IMMg1toKO_F3**	
**IMMg1toKO_F4**	
**IMMg1toKO_F8**	

Step 7: Plot stacked bar plot results for each sample group

In [8]:

```
df_taxa.plot(kind='bar', stacked=True, color=colors, width=0.75, figsize=(8,8
))
plt.title('Regenerated Figure', fontsize=18, fontweight='bold')
plt.xticks(rotation=45, ha='right', fontsize=14)
plt.legend(loc=(1.05, 0.3), prop={'size': 10}, reverse=True)
```

```
plt.ylabel('Percentage (%)')
plt.show()
```

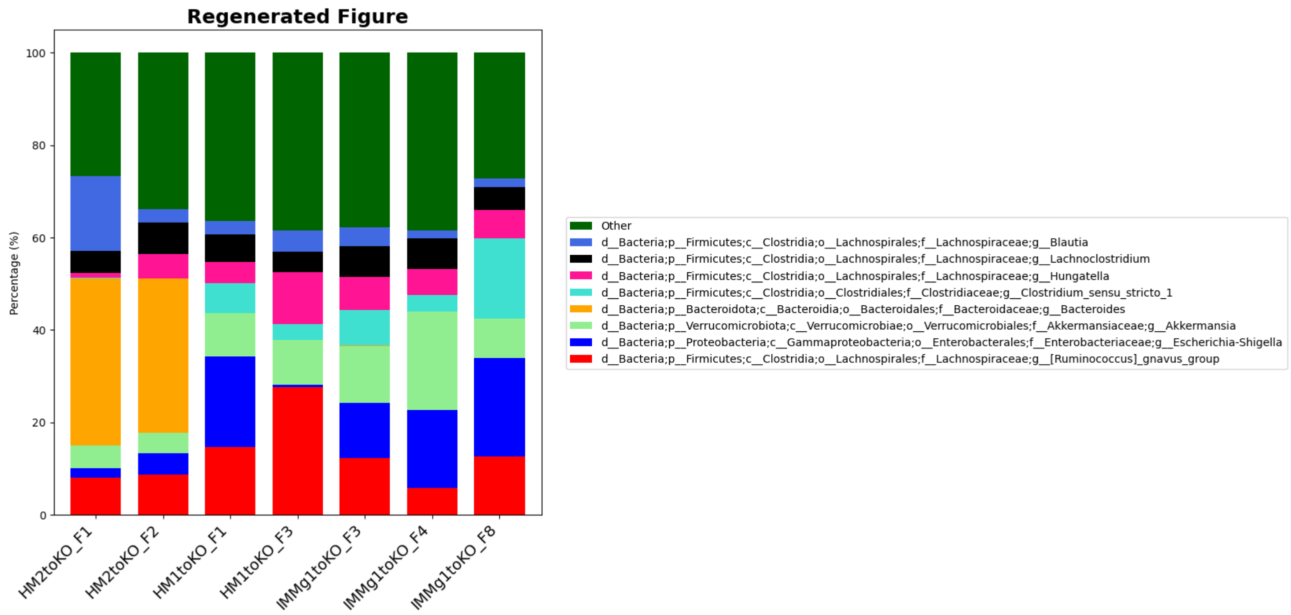

Step 8: Compare original and regenerated figures

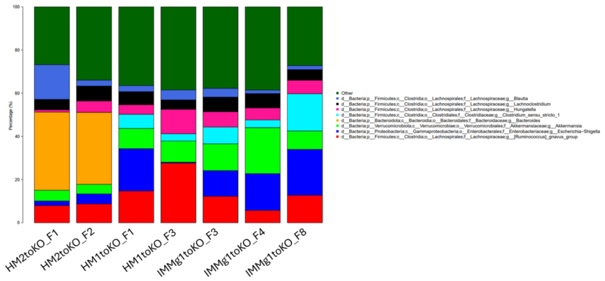

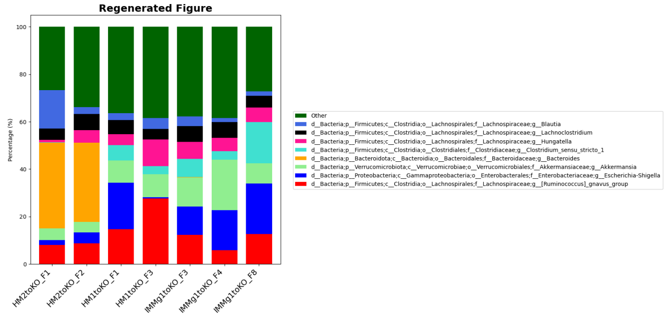

Contents

- **Aim**
- **Main Python Methods**
- **Data Processing Steps**
    - Step 1: Retrieve source feature counts table
    - Step 2: Filter feature counts table with 30 target sample group columns
    - Step 3: Calculate total read counts per sample
    - Step 4: Generate normalized counts feature table (without log10)
    - Step 5: Identify top 30 most abundant taxa
    - Step 6: Calculate proportional taxa abundance for each sample group
    - Step 7: Plot stacked bar plot results for each sample group
    - Step 8: Compare original and regenerated figures

Aim: Replicate figure

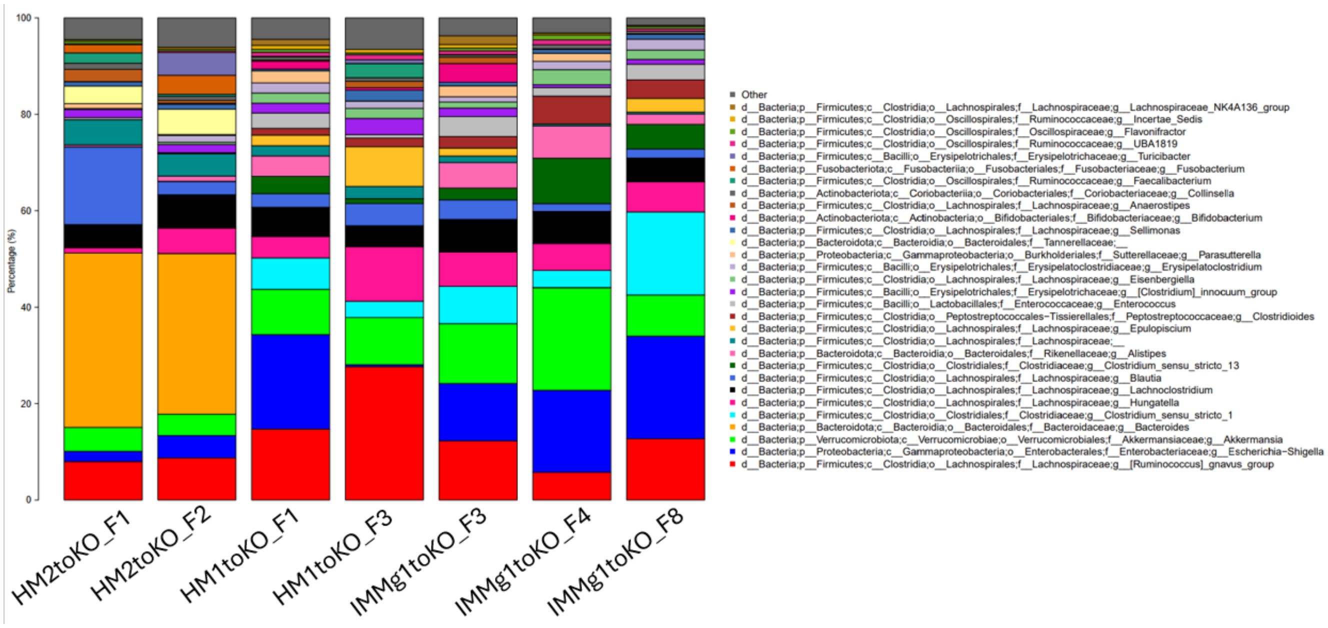

Main Python Methods

In [1]:
```
import numpy as np
import pandas as pd
import matplotlib.pyplot as plt
import os

# ******************************* MAIN METHODS *******************************
# Retrieve tab delimited file
def read_csv_file(file_path, skiprows=None, header = 0, sep = '\t', index_col=
False):
    df = pd.read_csv(file_path, skiprows=skiprows, sep=sep, header=header, ind
ex_col=index_col)

    return df
```

```
# Reference:  Function "get_cov_ellipse" was extracted from the following URL:

# https://scipython.com/book/chapter-7-matplotlib/examples/bmi-data-with-confi
dence-ellipses/
# (Learning Scientific Programming with Python by Christian Hill)
def get_cov_ellipse(cov, centre, nstd, **kwargs):
    """
    Return a matplotlib Ellipse patch representing the covariance matrix
    cov centred at centre and scaled by the factor nstd.
    """
    # Find and sort eigenvalues and eigenvectors into descending order
    eigvals, eigvecs = np.linalg.eigh(cov)
    order = eigvals.argsort()[::-1]
    eigvals, eigvecs = eigvals[order], eigvecs[:, order]

    # The anti-clockwise angle to rotate our ellipse by
    vx, vy = eigvecs[:,0][0], eigvecs[:,0][1]
    theta = np.arctan2(vy, vx)

    # Width and height of ellipse to draw
    width, height = 2 * nstd * np.sqrt(eigvals)
    return Ellipse(xy=centre, width=width, height=height,
                   angle=np.degrees(theta), fill=False, **kwargs)

# ******************************* INPUT FILE PATHS ***************************
****
# Get current working directory
current_working_dir = os.getcwd()
# original counts table file path
feature_tbl_file_path = os.path.join(current_working_dir, 'feature-table_balfo
ur.txt')
# dictionary for two sample groupings
dict_metadata = {'129.IL10KO_1':['F1-1', 'F1-2', 'F1-3', 'F1-4', 'F1-5', 'F1-
6'],
                 '129.IL10KO_2':['F2-1' ,'F2-2', 'F2-3', 'F2-4', 'F2-5'],
                'hFMT.1.2.3.gtKO_1':['F1-7', 'F1-8', 'F1-9', 'F1-10', 'F1-11',
 'F1-12', 'F1-13', 'F1-14', 'F1-15'],
                 'hFMT.1.2.3.gtKO_2':['F3-7', 'F3-8', 'F3-9', 'F3-10', 'F3-11'
, 'F3-12'],
                '1gKOgtKO_1':['F3-1', 'F3-2', 'F3-4', 'F3-5', 'F3-6'],
                 '1gKOgtKO_2':['F4-1', 'F4-2', 'F4-3', 'F4-4', 'F4-5'],
                '1gKOgtKO_3':['F8-1', 'F8-2', 'F8-3', 'F8-4', 'F8-5', 'F8-6',
'F8-7', 'F8-8']}
# cross reference for sample grouping names
cross_ref_dict = {'129.IL10KO_1':'HM2toKO_F1',
                  '129.IL10KO_2':'HM2toKO_F2',
                  'hFMT.1.2.3.gtKO_1':'HM1toKO_F1',
                  'hFMT.1.2.3.gtKO_2':'HM1toKO_F3',
                 '1gKOgtKO_1':'IMMg1toKO_F3',
                  '1gKOgtKO_2':'IMMg1toKO_F4',
                  '1gKOgtKO_3':'IMMg1toKO_F8'}
# stacked bar plot colors
colors = ["red","blue","lightgreen","orange" ,"turquoise",
"deeppink","black","royalblue",  "darkgreen",  "hotpink",
"darkcyan","goldenrod", "brown","grey", "purple" ,
"#7FC97F", "#BEAED4", "#FDC086", "#FFFF99", "#386CB0",
"#F0027F", "#BF5B17","#666666", "#1B9E77", "#D95F02",
"#7570B3","#E7298A", "#66A61E", "#E6AB02", "#A6761D",
"#666666"]
# Set parameter for top N most abundant taxa across all sample groups
top_most_abundant_count = 30
```

Data Processing Steps

Step 1: Retrieve source feature counts table

In [2]:
```
df_feature_counts = read_csv_file(feature_tbl_file_path, 1)
df_feature_counts = df_feature_counts.astype({col:'int32' for col in df_featur
e_counts.columns[1:]})
df_feature_counts
```

Out[2]:

	#OTU ID	1gKO.1	1gKO.2	1gKO.3	1gWT.1	1gWT.2	1gWT.:
0	d__Bacteria;p__Verrucomicrobiota;c__Verrucomic...	11370	14120	14648	16632	19817	2170
1	d__Bacteria;p__Firmicutes;c__Clostridia;o__Lac...	22545	25836	17048	7547	9272	599
2	d__Bacteria;p__Proteobacteria;c__Gammaproteoba...	5919	6481	6714	31	31	3
3	d__Bacteria;p__Firmicutes;c__Clostridia;o__Lac...	8134	9827	7090	8264	10100	632
4	d__Bacteria;p__Firmicutes;c__Clostridia;o__Lac...	6280	7649	5719	9715	11851	804
...	...	...	...	...	...	...	.
191	d__Bacteria;p__Firmicutes;c__Clostridia;o__Pep...	0	0	0	0	0	
192	d__Bacteria;p__Firmicutes;c__Clostridia;o__Clo...	0	0	0	0	0	
193	d__Bacteria;p__Firmicutes;c__Clostridia;o__Chr...	0	0	0	0	0	
194	d__Bacteria;p__Firmicutes;c__Incertae_Sedis;o_...	0	0	0	0	0	
195	d__Bacteria;p__Firmicutes;c__Bacilli;o__Lactob...	0	0	0	0	0	

196 rows × 111 columns

Step 2: Filter feature counts table with 30 target sample group columns

In [3]:
```
target_samples = [ sample for sample_group in list(dict_metadata.values()) for
 sample in sample_group ]
df_feature_counts_filtered = df_feature_counts[target_samples]
df_feature_counts_filtered
```

Out[3]:

	F1-1	F1-2	F1-3	F1-4	F1-5	F1-6	F2-1	F2-2	F2-3	F2-4	...	F4-4	F4-5	F8-1	
0	10401	2753	7914	2398	6595	2313	8957	10961	344	460	...	22240	22298	2627	1
1	7133	7051	3523	26054	524	2105	10749	5732	14621	319	...	8085	4026	10750	
2	2132	5867	4116	54	87	284	2194	1425	3296	1071	...	19808	23320	30563	2
3	1965	394	779	828	929	1973	4084	39	2827	17547	...	3631	7010	1440	
4	4785	1982	3119	2274	41521	49604	3306	3350	3278	2247	...	1346	1347	56	
...	...	...	...	...	...	...	...	...	...	...	...	...	...	...	
191	0	0	0	0	0	0	0	0	0	0	...	0	0	0	
192	0	0	0	0	0	0	0	0	0	0	...	0	0	0	
[illegible]	[illegible]	[illegible]	[illegible]	[illegible]	[illegible]	[illegible]	[illegible]	[illegible]	[illegible]	[illegible]		[illegible]	[illegible]	[illegible]	

**193**	0	0	0	0	0	0	0	0	0	0	...	0	0	0
**194**	0	0	0	0	0	0	0	0	0	0	...	0	0	0
**195**	0	0	0	0	0	0	0	0	0	0	...	0	0	0

196 rows × 44 columns

Step 3: Calculate total read counts per sample

In [4]:

```
sample_count_sums = df_feature_counts_filtered.sum(axis=0)
sample_count_sums
```

Out[4]:

```
F1-1      120620
F1-2       89533
F1-3      103243
F1-4       92643
F1-5      120934
F1-6       98057
F2-1       98214
F2-2       90013
F2-3       95932
F2-4      111659
F2-5       87600
F1-7      103534
F1-8      106286
F1-9       95787
F1-10     106356
F1-11      61083
F1-12     102329
F1-13     106433
F1-14     101965
F1-15      87272
F3-7       99046
F3-8      112057
F3-9      121801
F3-10      98907
F3-11      93879
F3-12     100855
F3-1       89907
F3-2       95182
F3-4      110663
F3-5      122538
F3-6      107733
F4-1       93069
F4-2      102268
F4-3      106452
F4-4      102778
F4-5      108196
F8-1      101846
F8-2      103946
F8-3       99383
F8-4      108840
F8-5       19323
F8-6       96062
F8-7      102664
F8-8       84853
dtype: int64
```

Step 4: Generate normalized counts feature table (without log10)

In [5]:
```
df_feature_counts_filtered_norm = (df_feature_counts_filtered/sample_count_sum
s)*sample_count_sums.mean()
df_feature_counts_filtered_norm
```

Out[5]:

	F1-1	F1-2	F1-3	F1-4	F1-5	F1-6	F2-1	
**0**	8547.969608	3048.107446	7598.758242	2565.923864	5405.969352	2338.321527	9040.581637	1
**1**	5862.192790	7806.830948	3382.666829	27878.473871	429.526602	2128.044450	10849.303564	
**2**	1752.165292	6495.912235	3952.045606	57.781438	71.314531	287.109085	2214.473162	
**3**	1614.917823	436.234774	747.969759	885.982051	761.508041	1994.599383	4122.109569	
**4**	3932.509814	2194.460210	2994.759534	2433.240561	34035.064968	50147.038912	3336.849715	
**...**	...	...	...	...	...	...	...	
**191**	0.000000	0.000000	0.000000	0.000000	0.000000	0.000000	0.000000	
**192**	0.000000	0.000000	0.000000	0.000000	0.000000	0.000000	0.000000	
**193**	0.000000	0.000000	0.000000	0.000000	0.000000	0.000000	0.000000	
**194**	0.000000	0.000000	0.000000	0.000000	0.000000	0.000000	0.000000	
**195**	0.000000	0.000000	0.000000	0.000000	0.000000	0.000000	0.000000	

196 rows × 44 columns

Step 5: Identify top eight most abundant taxa

In [6]:
```
df_feature_counts_filtered_norm.index = df_feature_counts['#OTU ID']
df_top_eight_genera = pd.DataFrame(df_feature_counts_filtered_norm.mean(axis=1
).\
                                   sort_values(ascending=False)[:top_most_abun
dant_count])
df_top_eight_genera.columns = ['norm_mean_abundance']
df_top_eight_genera
```

Out[6]:

#
**d__Bacteria;p__Firmicutes;c__Clostridia;o__Lachnospirales;f__Lachnospiraceae;g__[Ruminococcus]_gnavus**
**d__Bacteria;p__Proteobacteria;c__Gammaproteobacteria;o__Enterobacterales;f__Enterobacteriaceae;g__Esch S**
**d__Bacteria;p__Verrucomicrobiota;c__Verrucomicrobiae;o__Verrucomicrobiales;f__Akkermansiaceae;g__Akker**
**d__Bacteria;p__Bacteroidota;c__Bacteroidia;o__Bacteroidales;f__Bacteroidaceae;g__Bact**
**d__Bacteria;p__Firmicutes;c__Clostridia;o__Clostridiales;f__Clostridiaceae;g__Clostridium_sensu_s**
**d__Bacteria;p__Firmicutes;c__Clostridia;o__Lachnospirales;f__Lachnospiraceae;g__Hu**
**d__Bacteria;p__Firmicutes;c__Clostridia;o__Lachnospirales;f__Lachnospiraceae;g__Lachnoclos**
**d__Bacteria;p__Firmicutes;c__Clostridia;o__Lachnospirales;f__Lachnospiraceae;g__**
**d__Bacteria;p__Firmicutes;c__Clostridia;o__Clostridiales;f__Clostridiaceae;g__Clostridium_sensu_str**
**d__Bacteria;p__Bacteroidota;c__Bacteroidia;o__Bacteroidales;f__Rikenellaceae;g__A**

d__Bacteria;p__Firmicutes;c__Clostridia;o__Lachnospirales;f__Lachnospira
d__Bacteria;p__Firmicutes;c__Clostridia;o__Lachnospirales;f__Lachnospiraceae;g__Epulo
d__Bacteria;p__Firmicutes;c__Clostridia;o__Peptostreptocc Tissierellales;f__Peptostreptococcaceae;g__Clostri
d__Bacteria;p__Firmicutes;c__Bacilli;o__Lactobacillales;f__Enterococcaceae;g__Entero
d__Bacteria;p__Firmicutes;c__Bacilli;o__Erysipelotrichales;f__Erysipelotrichaceae;g__[Clostridium]_innocuum
d__Bacteria;p__Firmicutes;c__Clostridia;o__Lachnospirales;f__Lachnospiraceae;g__Eisenb
d__Bacteria;p__Firmicutes;c__Bacilli;o__Erysipelotrichales;f__Erysipelatoclostridiaceae;g__Erysipelatoclos
d__Bacteria;p__Proteobacteria;c__Gammaproteobacteria;o__Burkholderiales;f__Sutterellaceae;g__Parasu
d__Bacteria;p__Bacteroidota;c__Bacteroidia;o__Bacteroidales;f__Tannerella
d__Bacteria;p__Firmicutes;c__Clostridia;o__Lachnospirales;f__Lachnospiraceae;g__Sell
d__Bacteria;p__Actinobacteriota;c__Actinobacteria;o__Bifidobacteriales;f__Bifidobacteriaceae;g__Bifidoba
d__Bacteria;p__Firmicutes;c__Clostridia;o__Lachnospirales;f__Lachnospiraceae;g__Anaer
d__Bacteria;p__Actinobacteriota;c__Coriobacteriia;o__Coriobacteriales;f__Coriobacteriaceae;g__Co
d__Bacteria;p__Firmicutes;c__Clostridia;o__Oscillospirales;f__Ruminococcaceae;g__Faecaliba
d__Bacteria;p__Fusobacteriota;c__Fusobacteriia;o__Fusobacteriales;f__Fusobacteriaceae;g__Fusoba
d__Bacteria;p__Firmicutes;c__Bacilli;o__Erysipelotrichales;f__Erysipelotrichaceae;g__Turi
d__Bacteria;p__Firmicutes;c__Clostridia;o__Oscillospirales;f__Ruminococcaceae;g__U
d__Bacteria;p__Firmicutes;c__Clostridia;o__Oscillospirales;f__Oscillospiraceae;g__Flavon
d__Bacteria;p__Firmicutes;c__Clostridia;o__Oscillospirales;f__Ruminococcaceae;g__Incerta
d__Bacteria;p__Firmicutes;c__Clostridia;o__Lachnospirales;f__Lachnospiraceae;g__Lachnospiraceae_NK4A136

Step 6: Calculate proportional taxa abundance for each sample group

In [7]:

```
# Initialize dictionary to store proportional abundances for each sample group
taxa_dict = {}
for taxa in df_top_eight_genera.index:
    taxa_dict[taxa] = []
taxa_dict['Other'] = []
sample_group_list = []

# Populate dictionary for sample groups
for sample_group in dict_metadata:
    cross_ref_dict[sample_group]
    sample_group_list.append(cross_ref_dict[sample_group])
    # For a given sample group, calculate the sum of the normalized counts for
 each taxa
    ser = df_feature_counts_filtered_norm[dict_metadata[sample_group]].sum(axi
s=1)
    # For a given sample group, calculate the proportion for each taxa
    ser_prop = ser/ser.sum()
    # For a given sample group, verify that the sum of all taxa proportions is
 equal to zero
    assert np.isclose(ser_prop.sum(), 1.0)
    # Filter out top eight most abundant taxa previously identified in step 5
    ser_prop_top_eight =  ser_prop[df_top_eight_genera.index]
    # Calculate and store the sum of proportions for all other taxa
```

```python
    # that are not in the set of top eight taxa
    other = 1.0 - ser_prop_top_eight.sum()
    taxa_dict['Other'].append(other)
    # Store the proportions for the top eight most abundant taxa
    for item in ser_prop_top_eight.items():
        taxa_dict[item[0]].append(item[1])

# Save data to a dataframe to plot during the following step
df_taxa = pd.DataFrame(taxa_dict)
df_taxa.index = sample_group_list
# Multiply by 100 to convert to percentage
df_taxa = df_taxa * 100
df_taxa
```

Out[7]:

	d__Bacteria;p__Firmicutes;c__Clostridia;o__Lachnospirales;f__Lachnospiraceae;g__[Ruminoc
**HM2toKO_F1**	
**HM2toKO_F2**	
**HM1toKO_F1**	
**HM1toKO_F3**	
**IMMg1toKO_F3**	
**IMMg1toKO_F4**	
**IMMg1toKO_F8**	

7 rows × 31 columns

Step 7: Plot stacked bar plot results for each sample group

In [8]:
```python
df_taxa.plot(kind='bar', stacked=True, color=colors, width=0.75, figsize=(8,8
))
plt.title('Regenerated Figure', fontsize=18, fontweight='bold')
plt.xticks(rotation=45, ha='right', fontsize=14)
plt.legend(loc=(1.05, 0.01), prop={'size': 9}, reverse=True)
plt.ylabel('Percentage (%)')
plt.show()
```

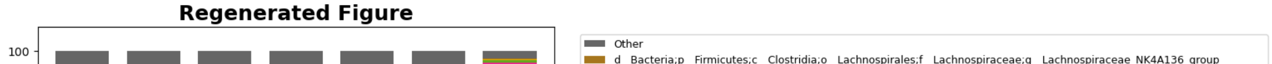

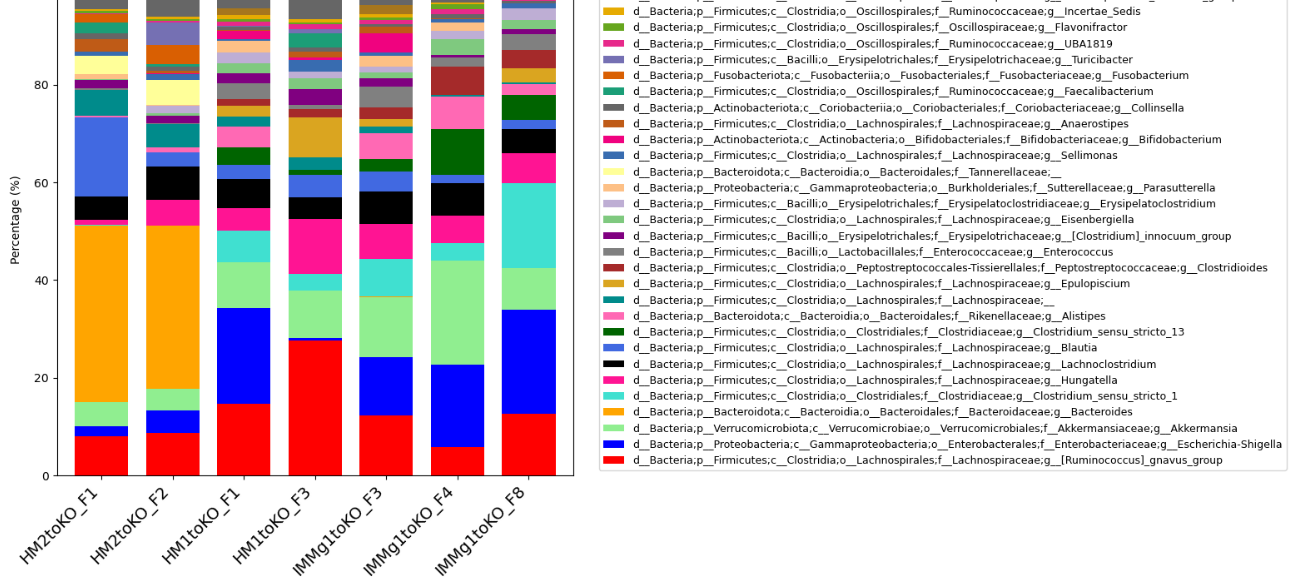

Step 8: Compare original and regenerated figures

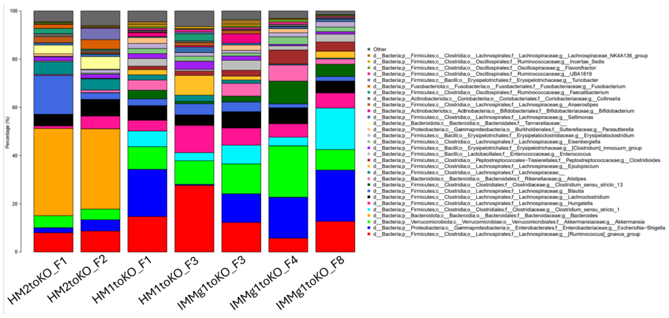

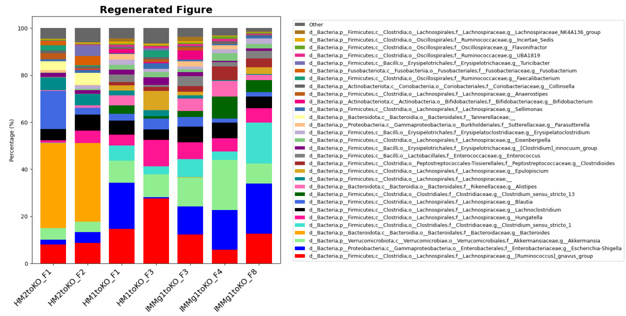

In [ ]:

Sartor Lab Figure Validation Summary

Figure Comparision 1

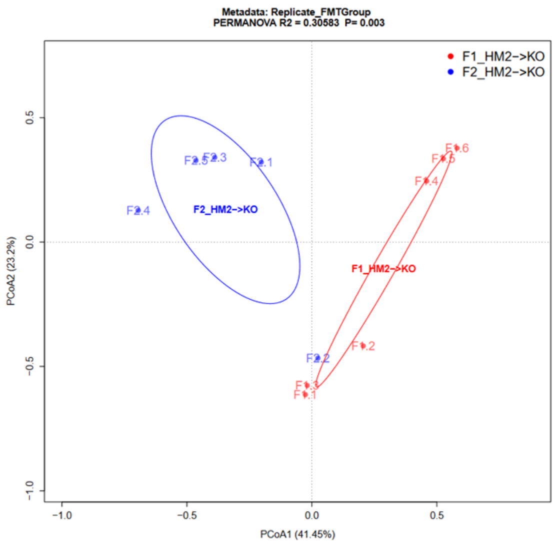

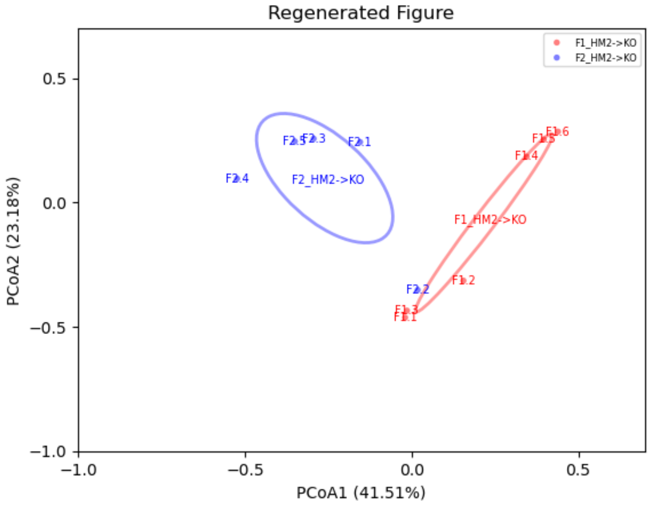

Figure Comparision 2

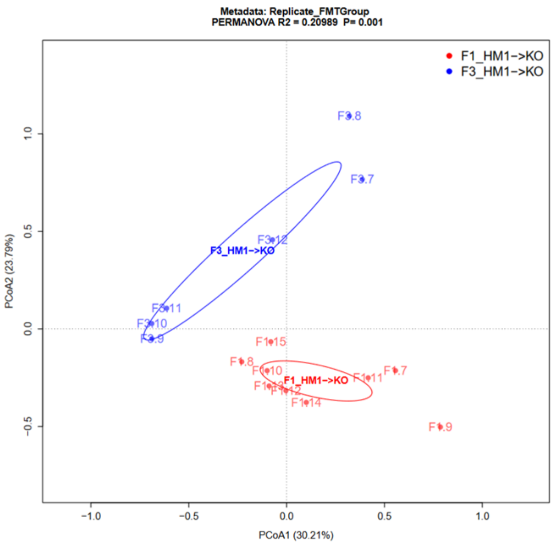

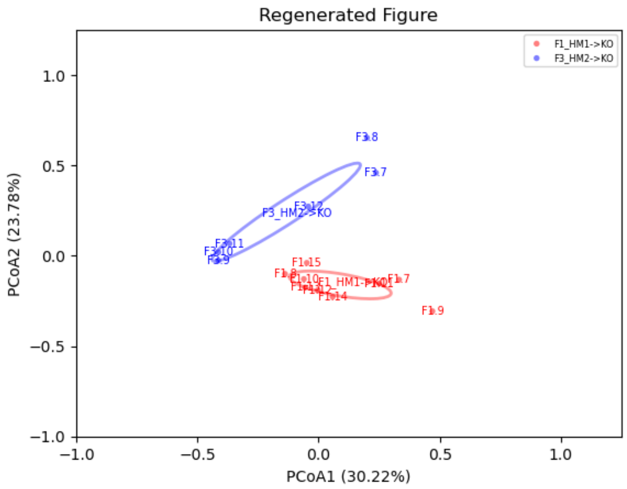

Figure Comparision 3

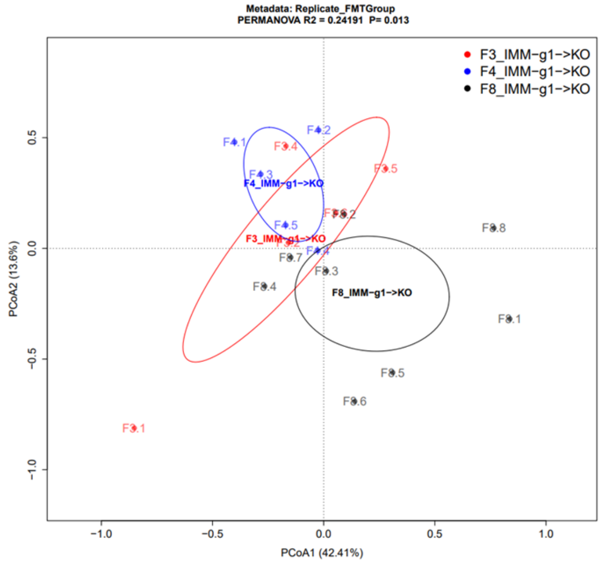

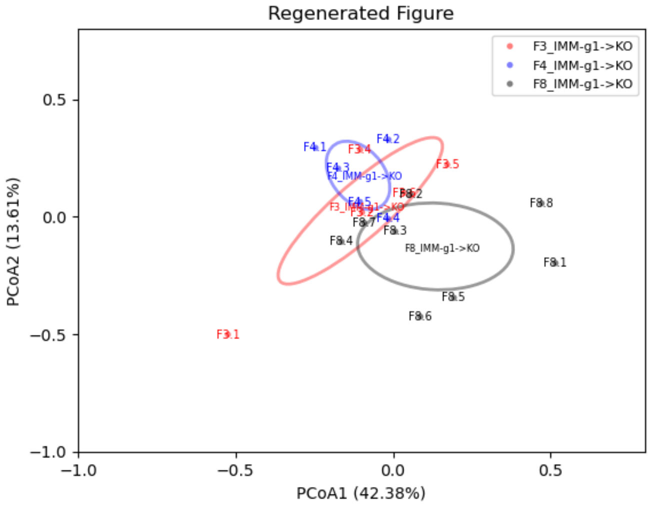

Figure Comparision 4

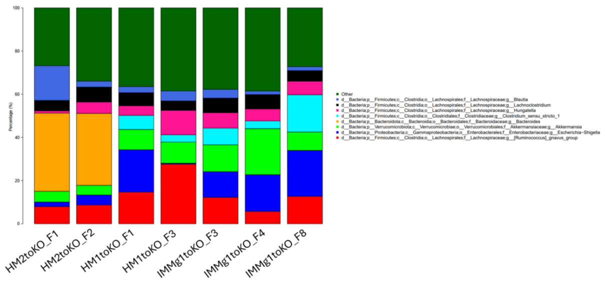

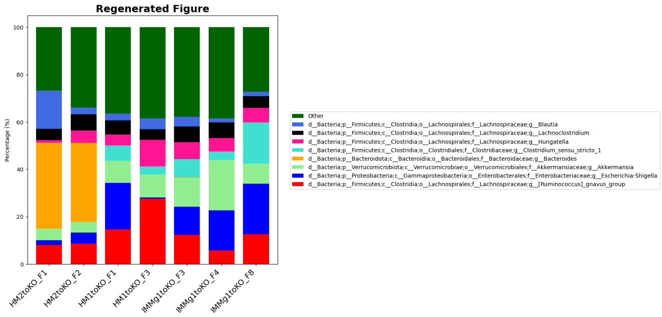

Figure Comparision 5

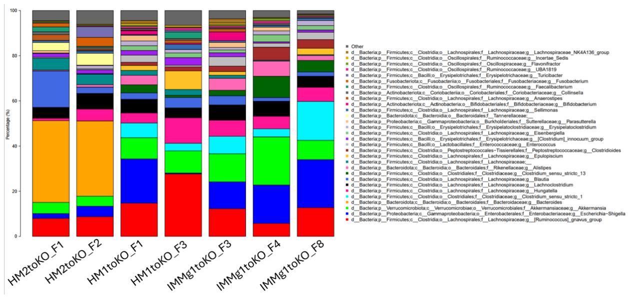

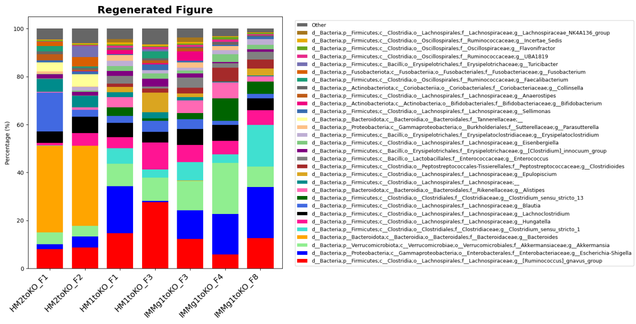

Supplement: Supplementary file 2 — Supplementary file 1 contains R and Python code used in our analyses. Supplementary Table S1 contains Differential abundance analysis between groups excluding genera present in less than 10% of the samples. The Supplemental Figures and Experimental Methods file contains supplemental figures with figure legends and supplemental experimental procedures. [file Supplemental_File_1_ESM.pdf › Supplemental_File_1_ESM.pdf]
